# Supplementary figures and images for: Uncertainty-aware mixed-variable machine learning for materials design (part 2 of 2)
Source: Sci Rep. 2022 Nov 17;12:19760. doi: 10.1038/s41598-022-23431-2 (PMC9672324; doi:10.1038/s41598-022-23431-2)

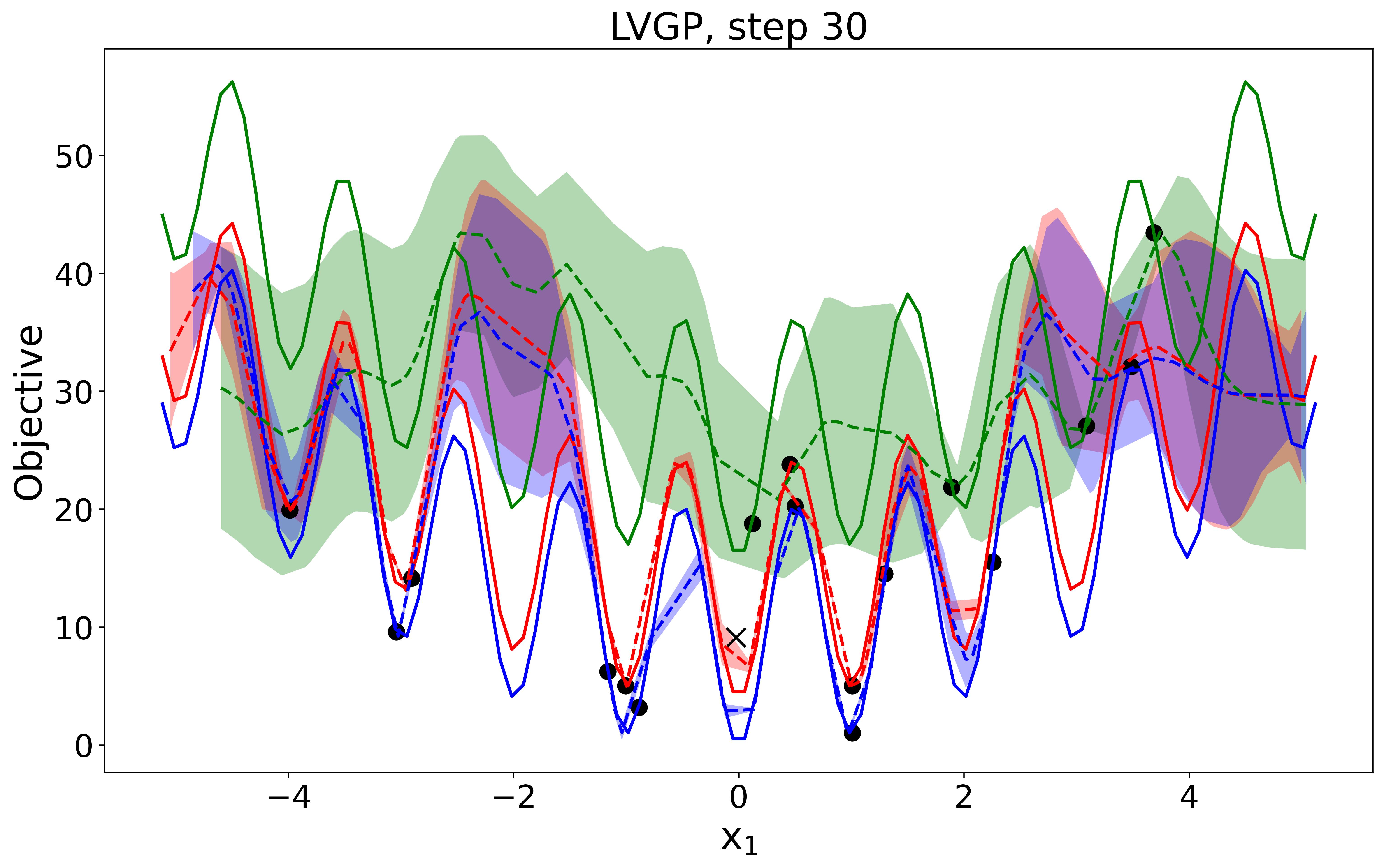

Supplement: Supplementary file 1 — Supplementary Information 1. [file 41598_2022_23431_MOESM1_ESM.zip › Sampling_Sequence_Figures/Rastrigin_Function/rastrigin2_LVGP_30.jpg]

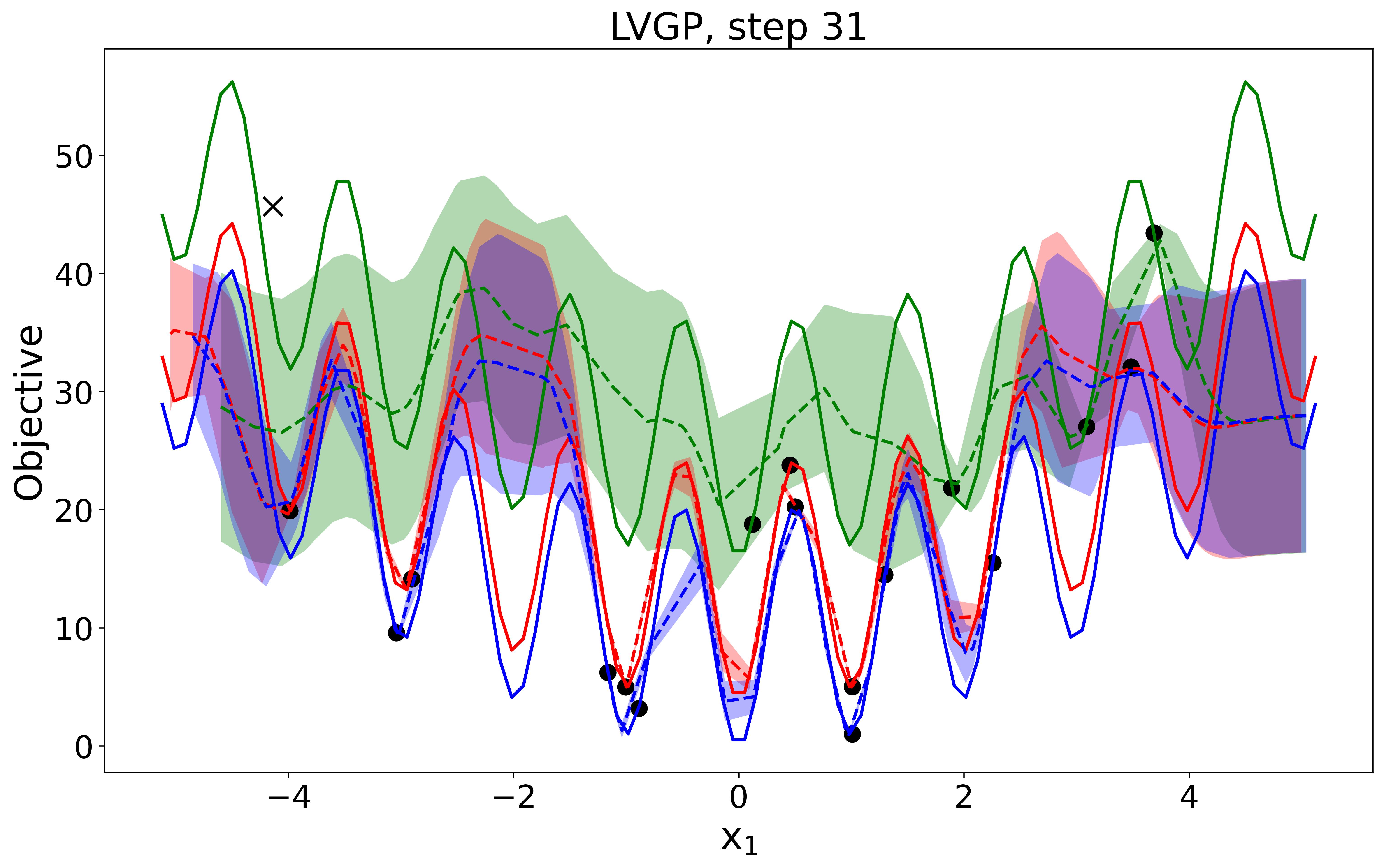

Supplement: Supplementary file 1 — Supplementary Information 1. [file 41598_2022_23431_MOESM1_ESM.zip › Sampling_Sequence_Figures/Rastrigin_Function/rastrigin2_LVGP_31.jpg]

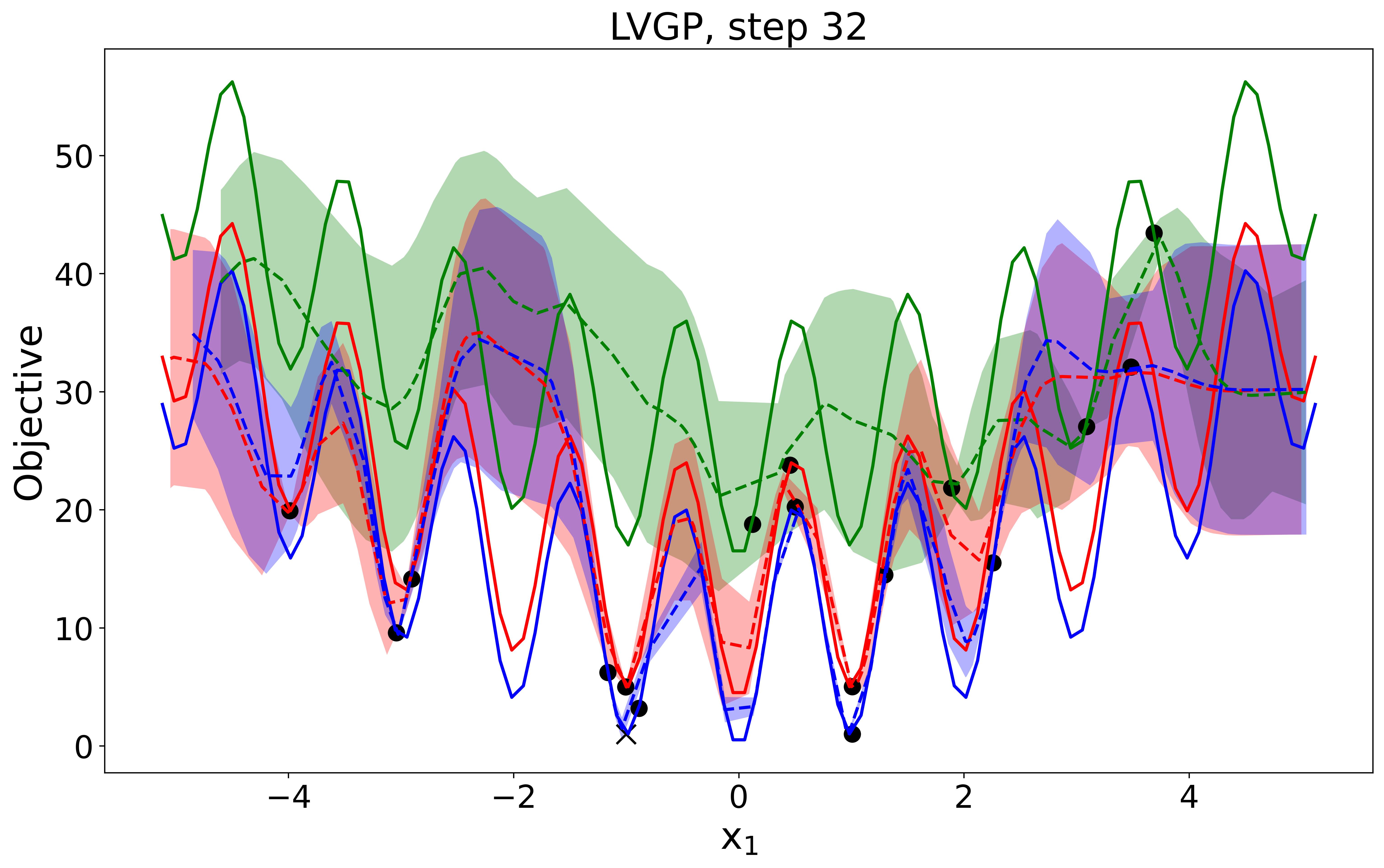

Supplement: Supplementary file 1 — Supplementary Information 1. [file 41598_2022_23431_MOESM1_ESM.zip › Sampling_Sequence_Figures/Rastrigin_Function/rastrigin2_LVGP_32.jpg]

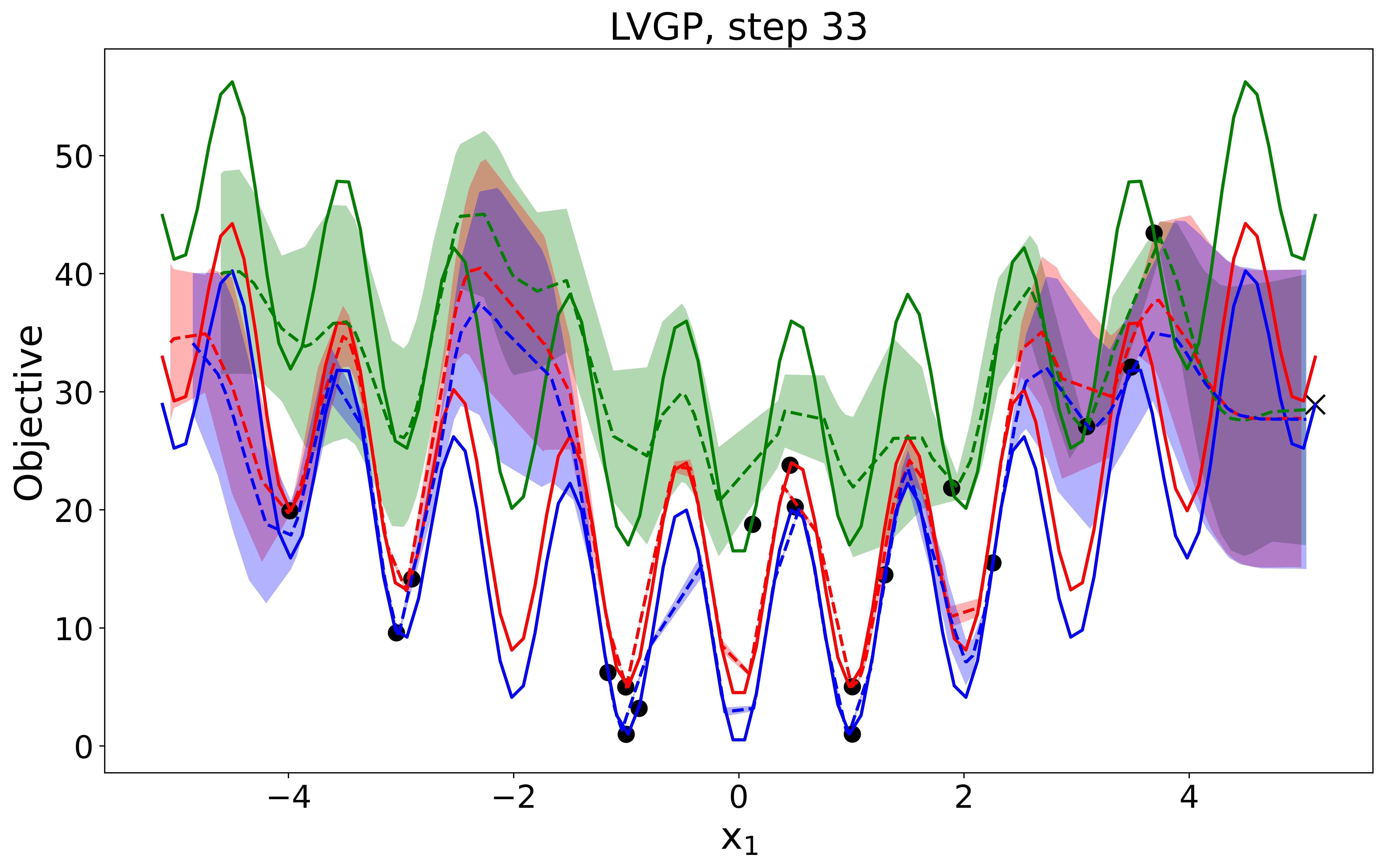

Supplement: Supplementary file 1 — Supplementary Information 1. [file 41598_2022_23431_MOESM1_ESM.zip › Sampling_Sequence_Figures/Rastrigin_Function/rastrigin2_LVGP_33.jpg]

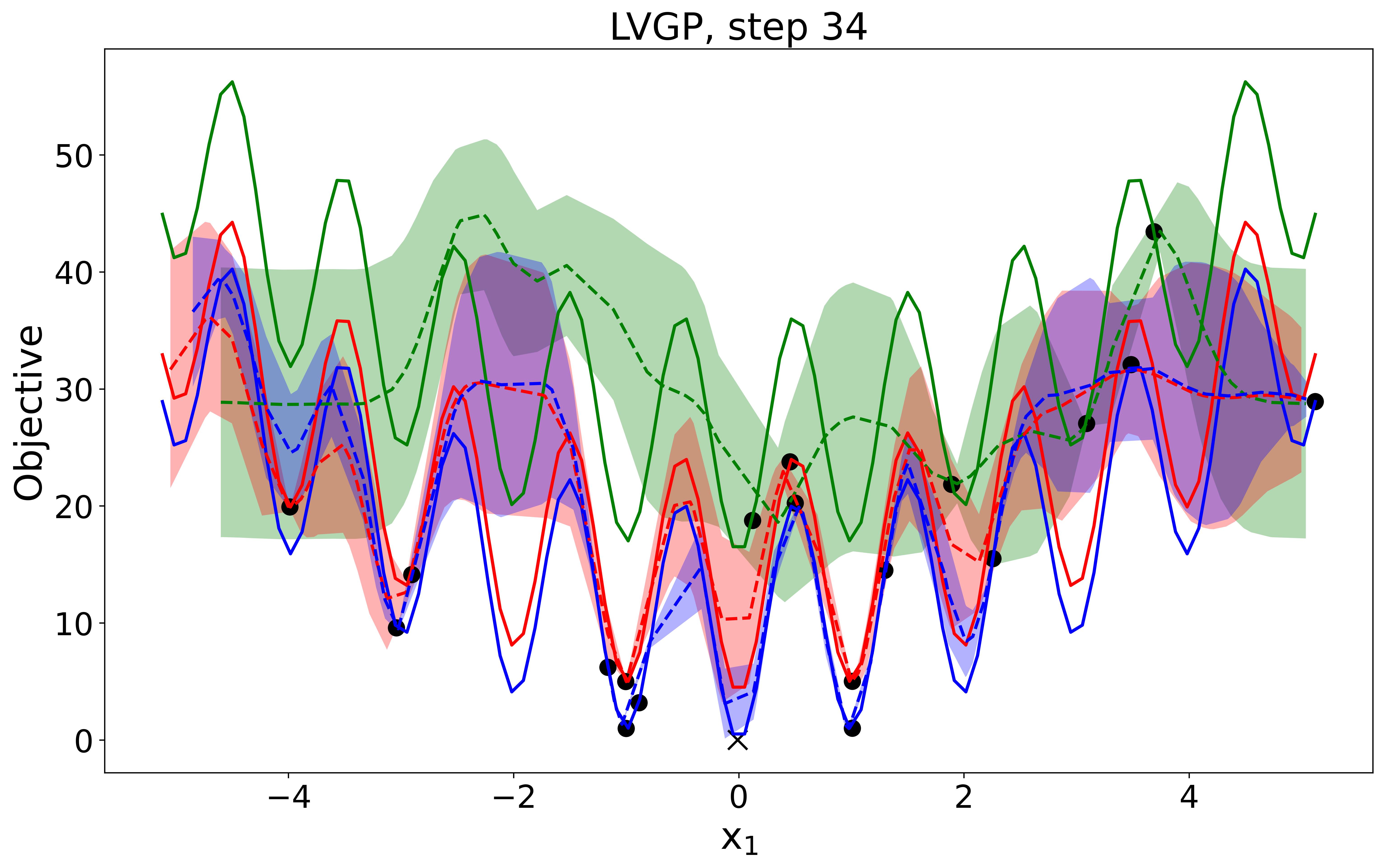

Supplement: Supplementary file 1 — Supplementary Information 1. [file 41598_2022_23431_MOESM1_ESM.zip › Sampling_Sequence_Figures/Rastrigin_Function/rastrigin2_LVGP_34.jpg]

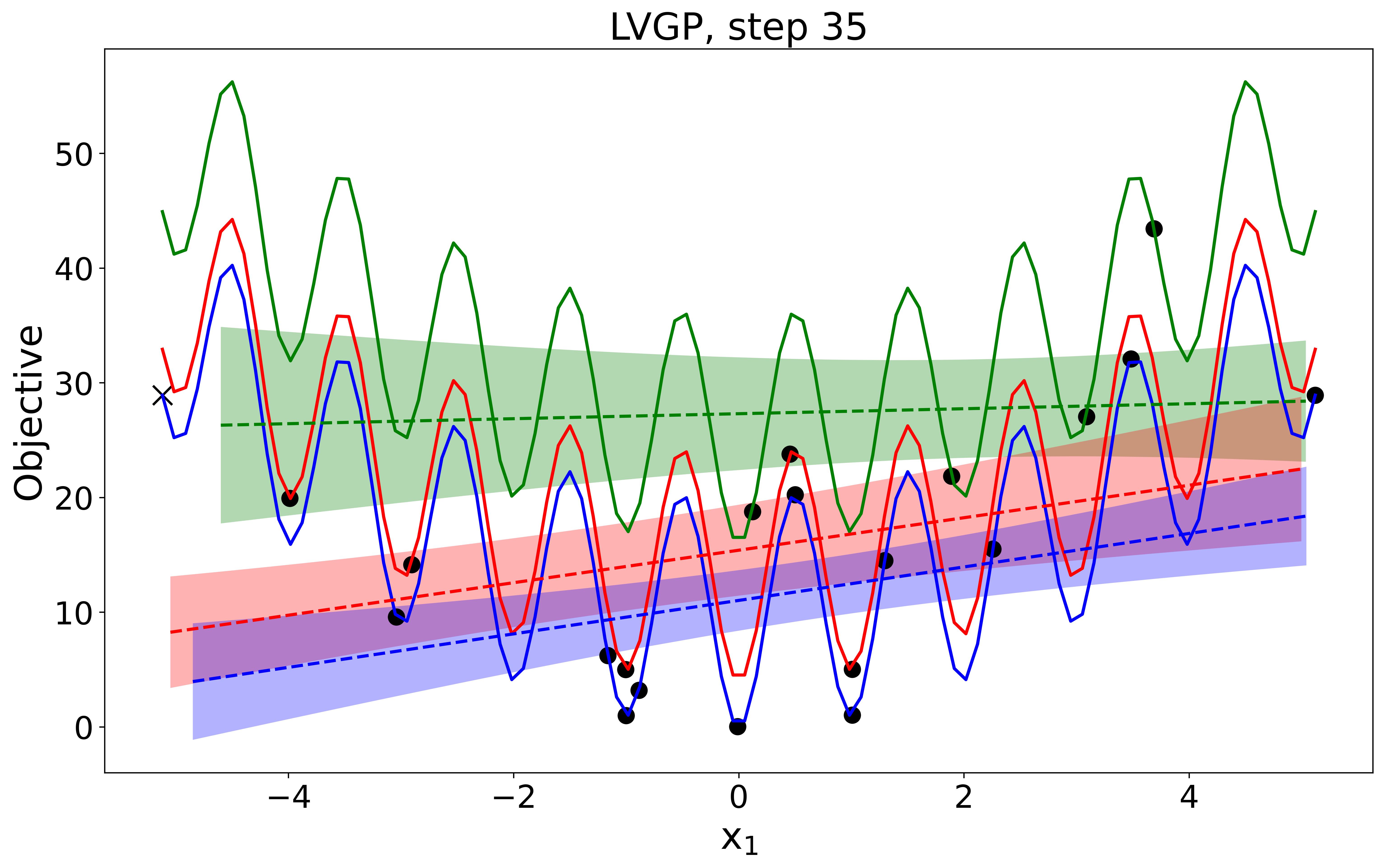

Supplement: Supplementary file 1 — Supplementary Information 1. [file 41598_2022_23431_MOESM1_ESM.zip › Sampling_Sequence_Figures/Rastrigin_Function/rastrigin2_LVGP_35.jpg]

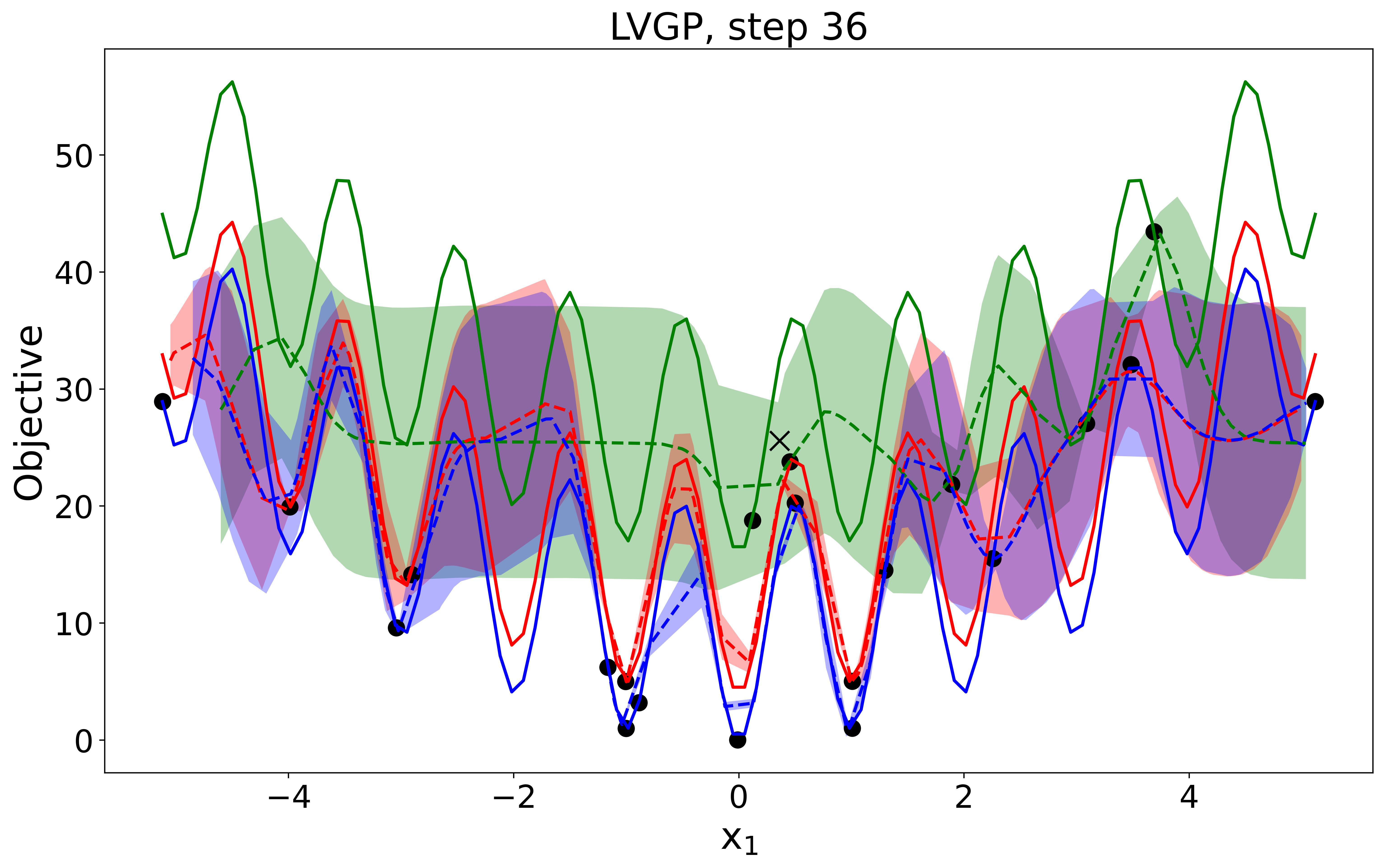

Supplement: Supplementary file 1 — Supplementary Information 1. [file 41598_2022_23431_MOESM1_ESM.zip › Sampling_Sequence_Figures/Rastrigin_Function/rastrigin2_LVGP_36.jpg]

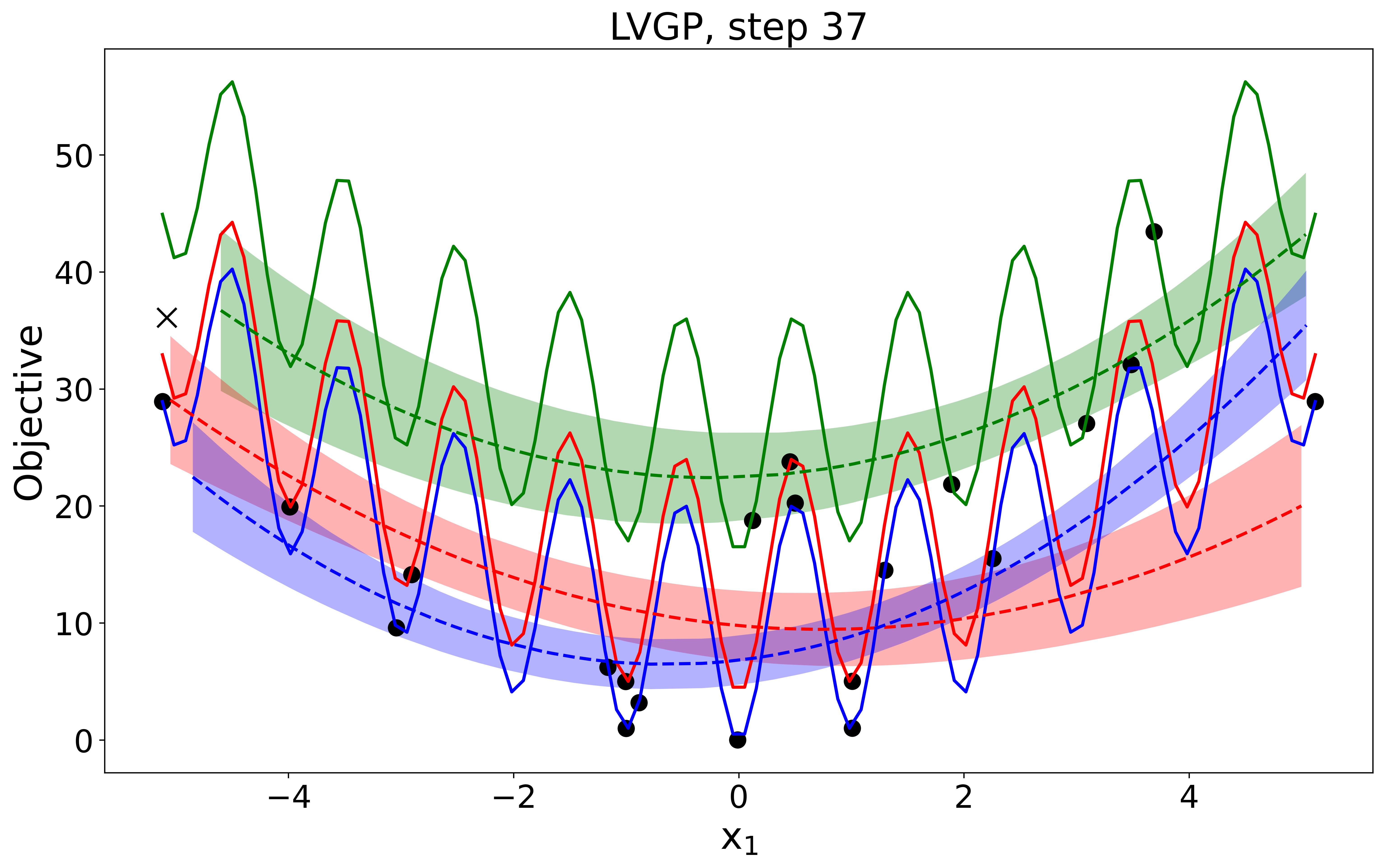

Supplement: Supplementary file 1 — Supplementary Information 1. [file 41598_2022_23431_MOESM1_ESM.zip › Sampling_Sequence_Figures/Rastrigin_Function/rastrigin2_LVGP_37.jpg]

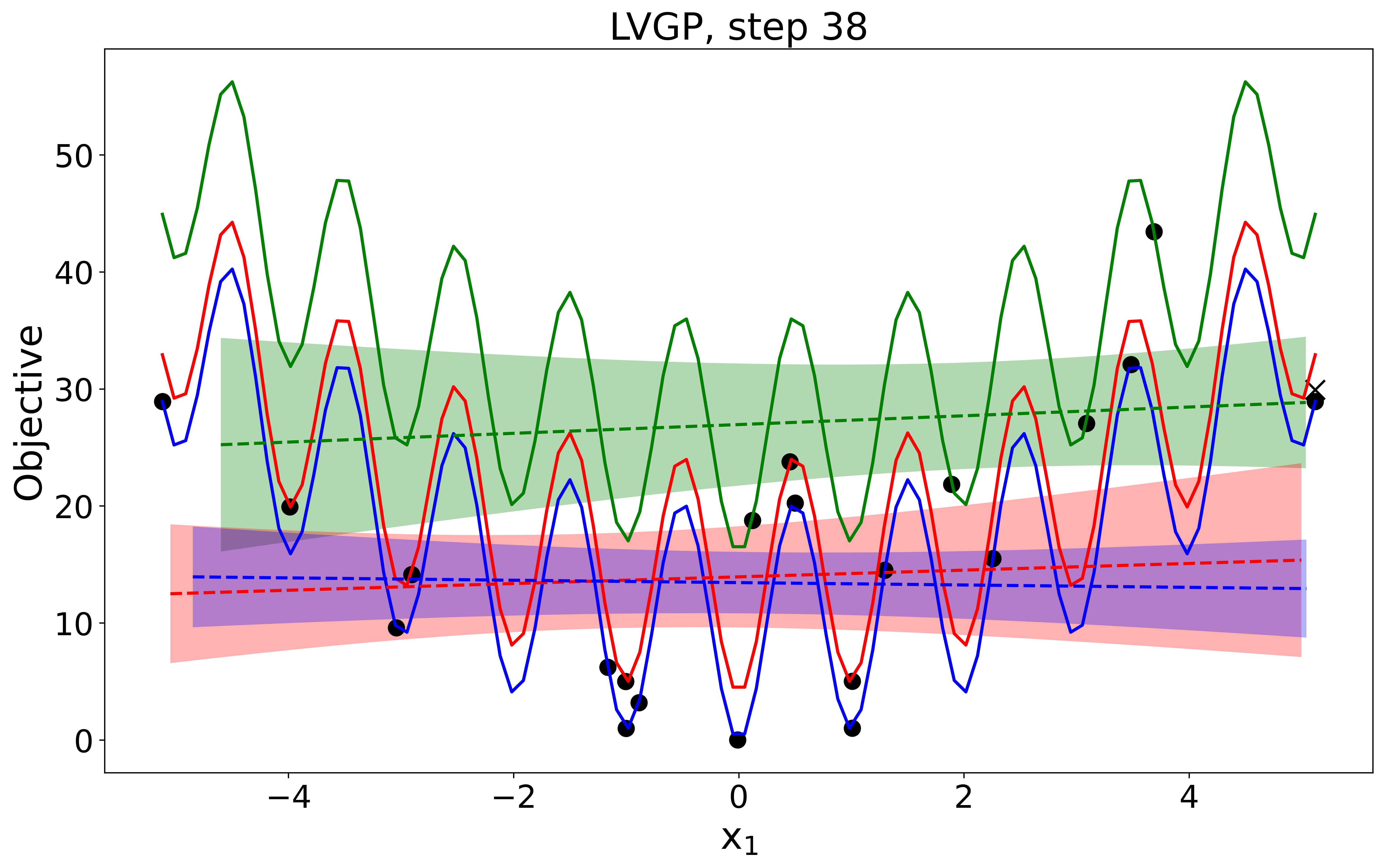

Supplement: Supplementary file 1 — Supplementary Information 1. [file 41598_2022_23431_MOESM1_ESM.zip › Sampling_Sequence_Figures/Rastrigin_Function/rastrigin2_LVGP_38.jpg]

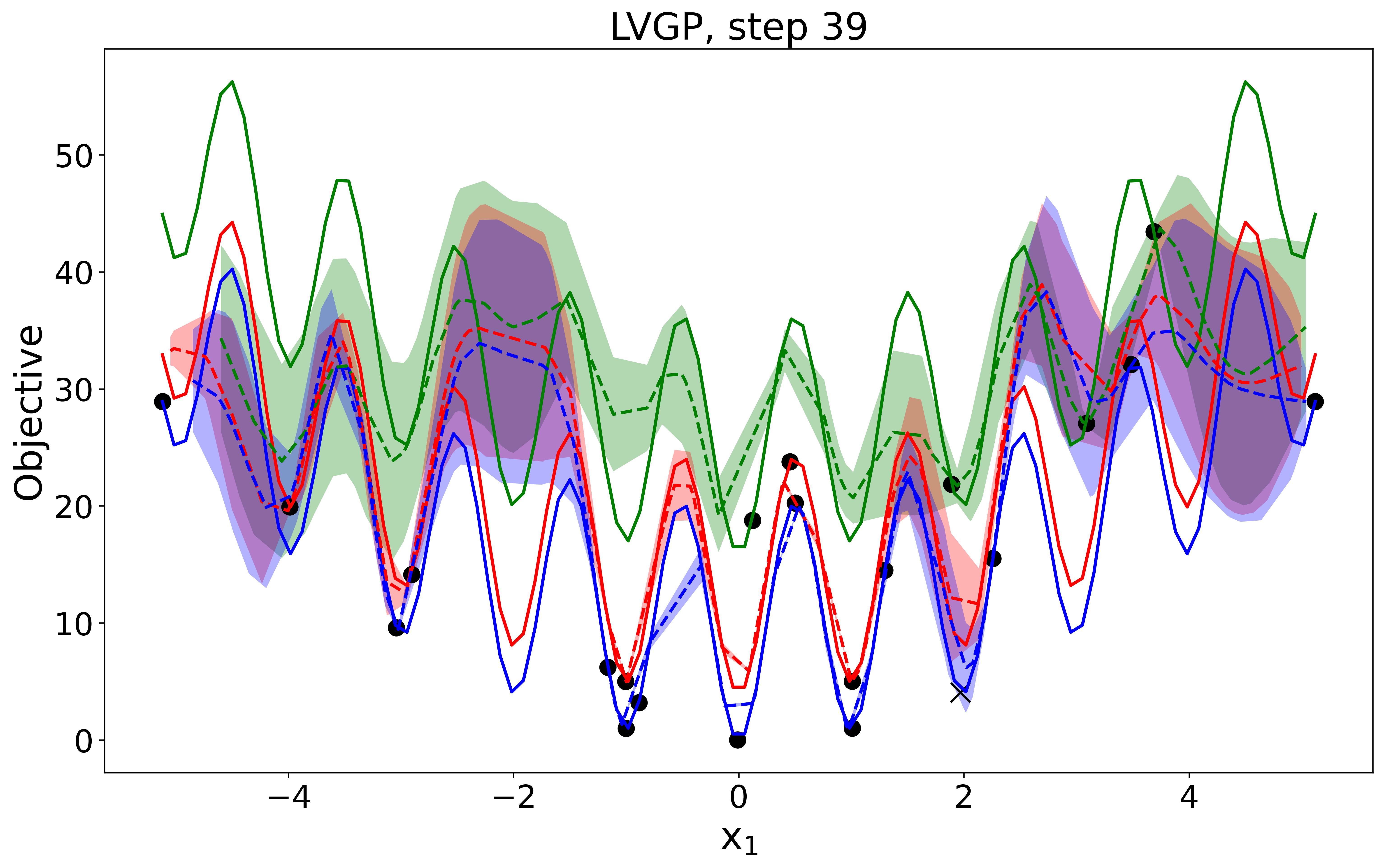

Supplement: Supplementary file 1 — Supplementary Information 1. [file 41598_2022_23431_MOESM1_ESM.zip › Sampling_Sequence_Figures/Rastrigin_Function/rastrigin2_LVGP_39.jpg]

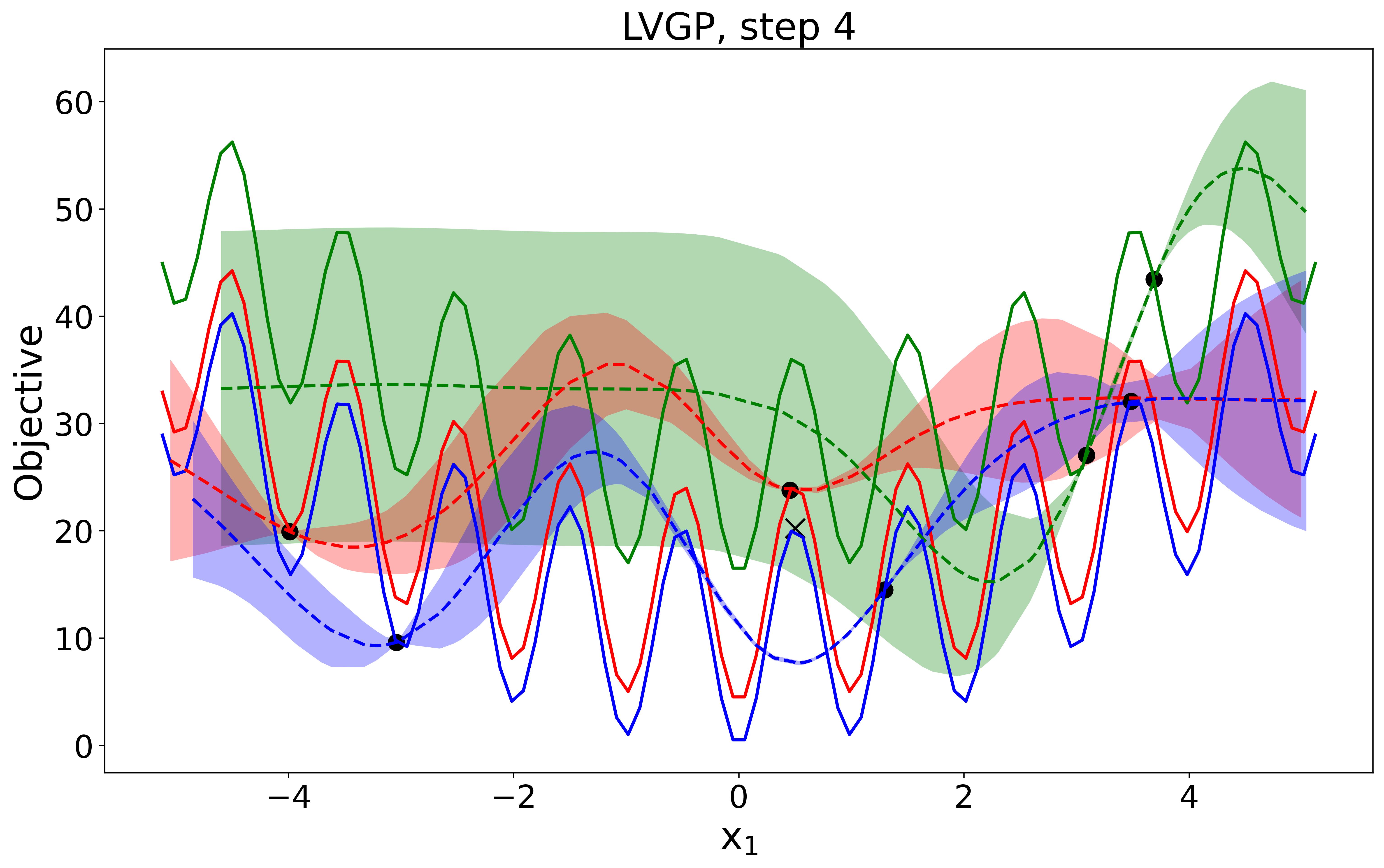

Supplement: Supplementary file 1 — Supplementary Information 1. [file 41598_2022_23431_MOESM1_ESM.zip › Sampling_Sequence_Figures/Rastrigin_Function/rastrigin2_LVGP_4.jpg]

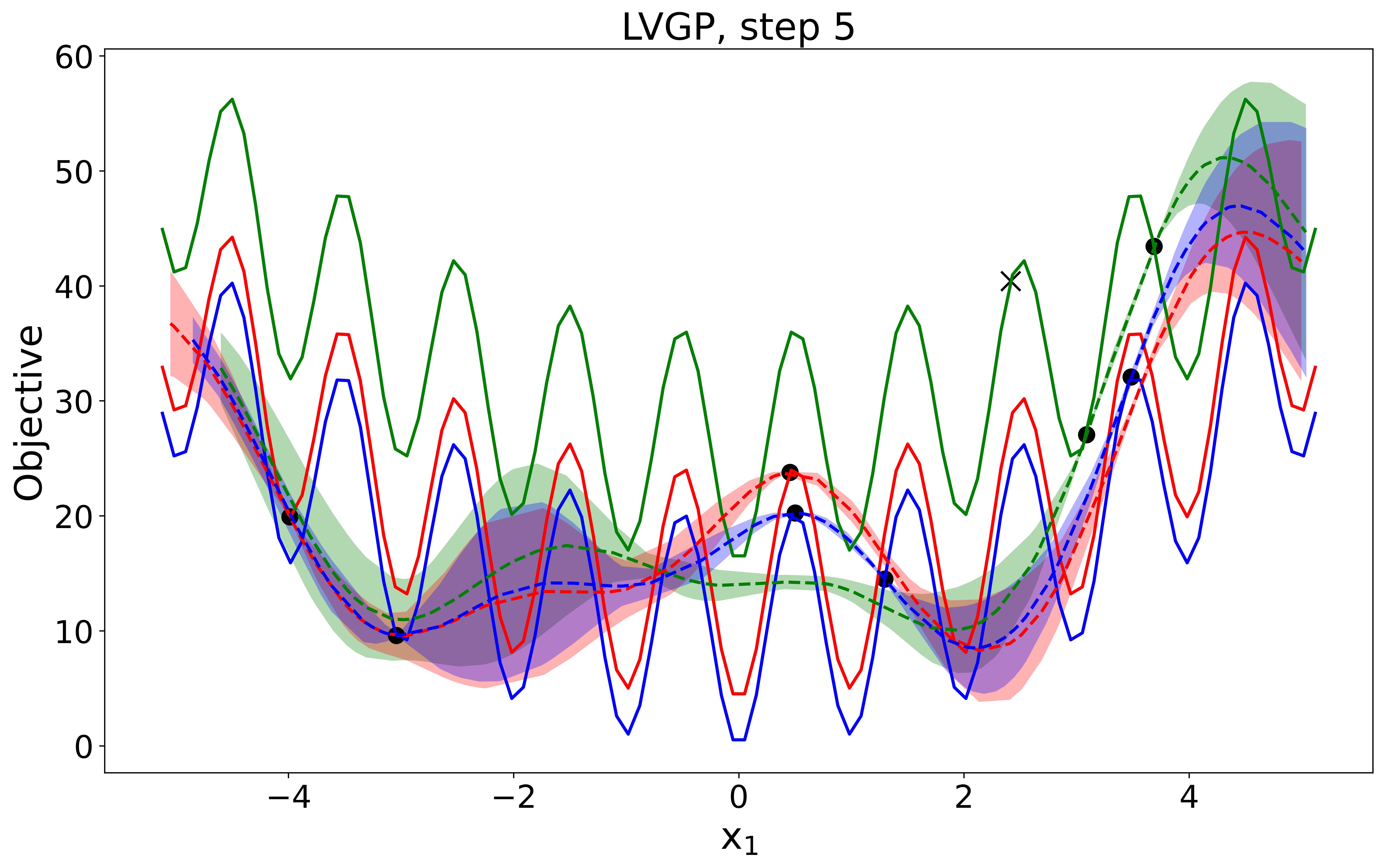

Supplement: Supplementary file 1 — Supplementary Information 1. [file 41598_2022_23431_MOESM1_ESM.zip › Sampling_Sequence_Figures/Rastrigin_Function/rastrigin2_LVGP_5.jpg]

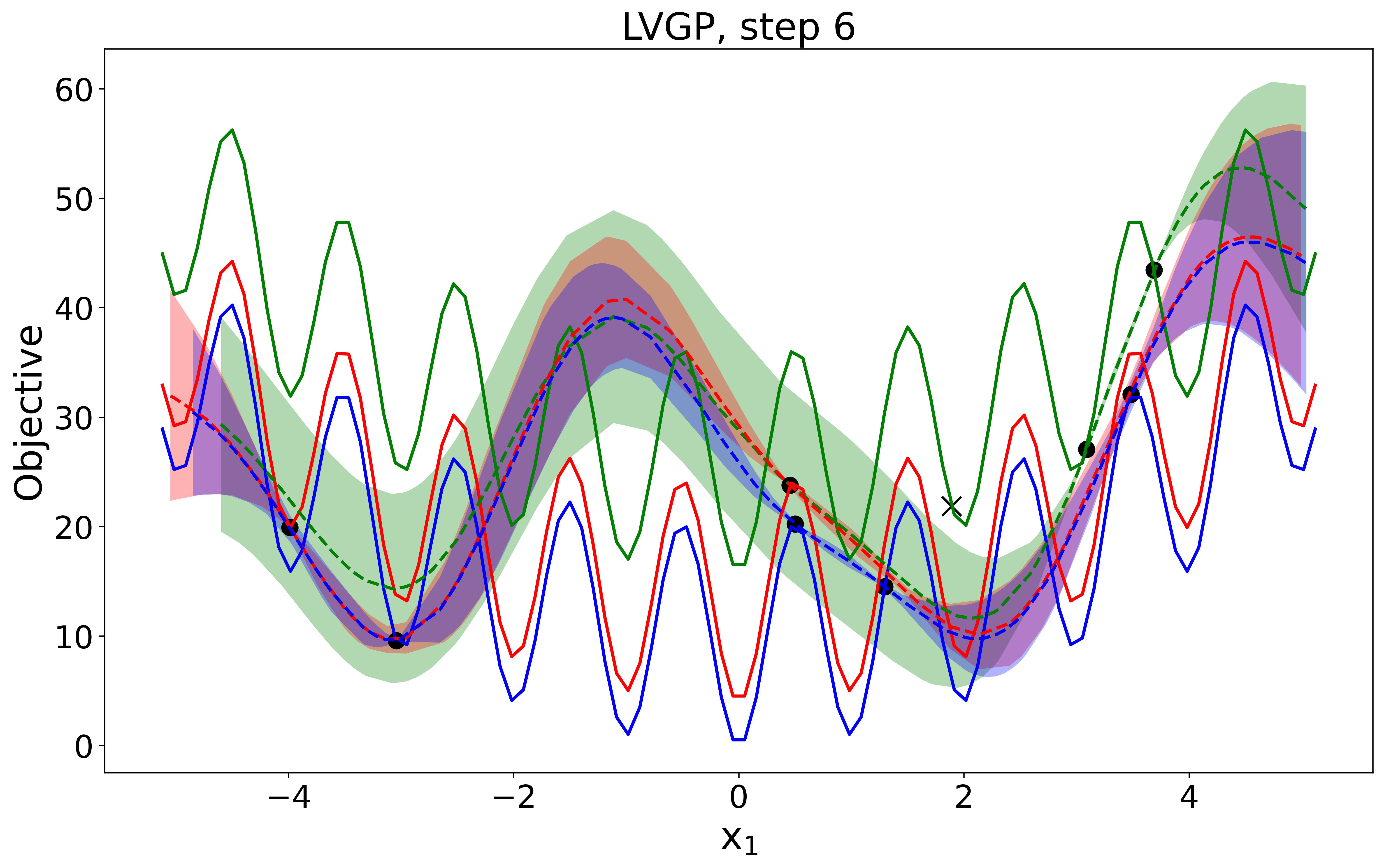

Supplement: Supplementary file 1 — Supplementary Information 1. [file 41598_2022_23431_MOESM1_ESM.zip › Sampling_Sequence_Figures/Rastrigin_Function/rastrigin2_LVGP_6.jpg]

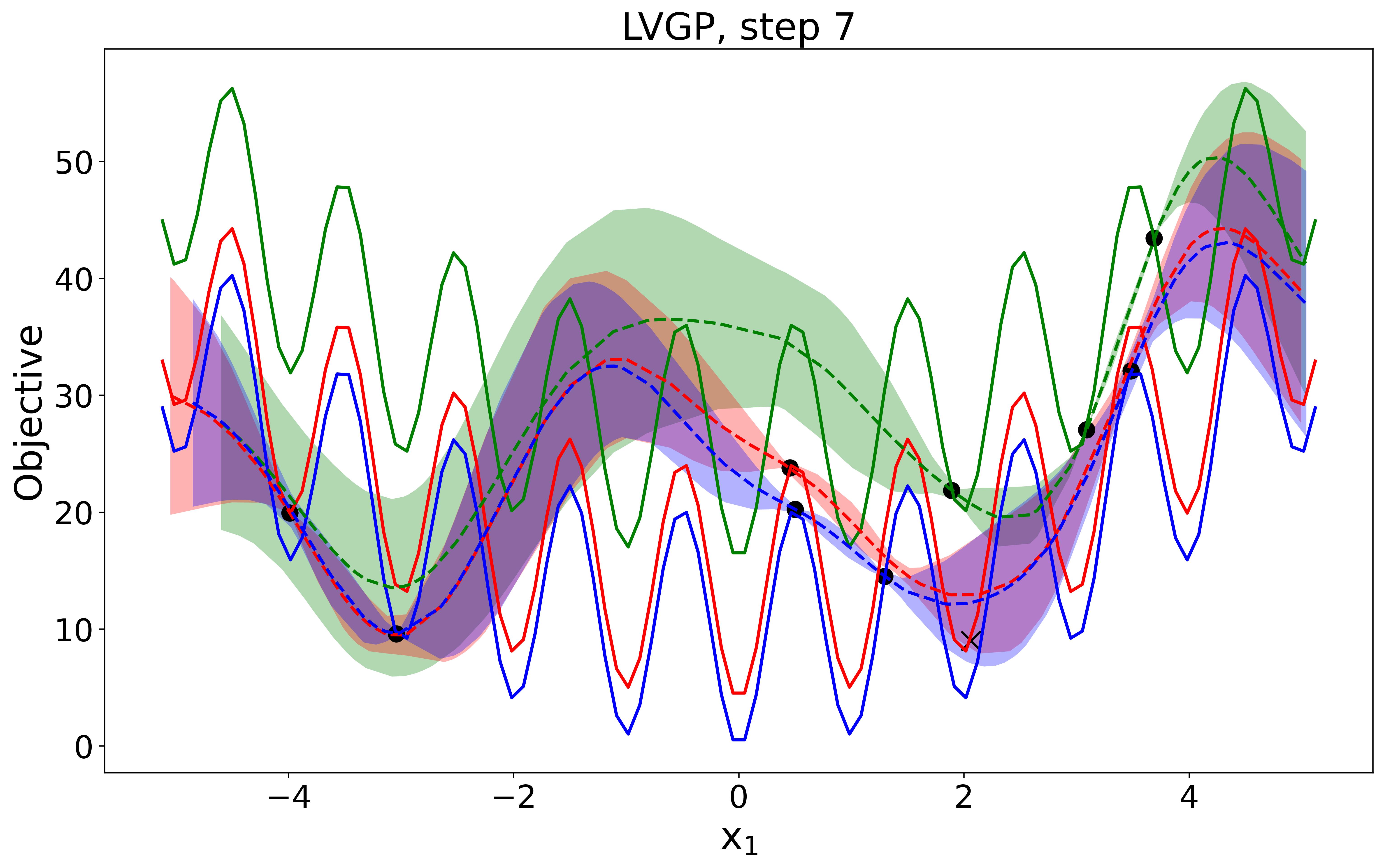

Supplement: Supplementary file 1 — Supplementary Information 1. [file 41598_2022_23431_MOESM1_ESM.zip › Sampling_Sequence_Figures/Rastrigin_Function/rastrigin2_LVGP_7.jpg]

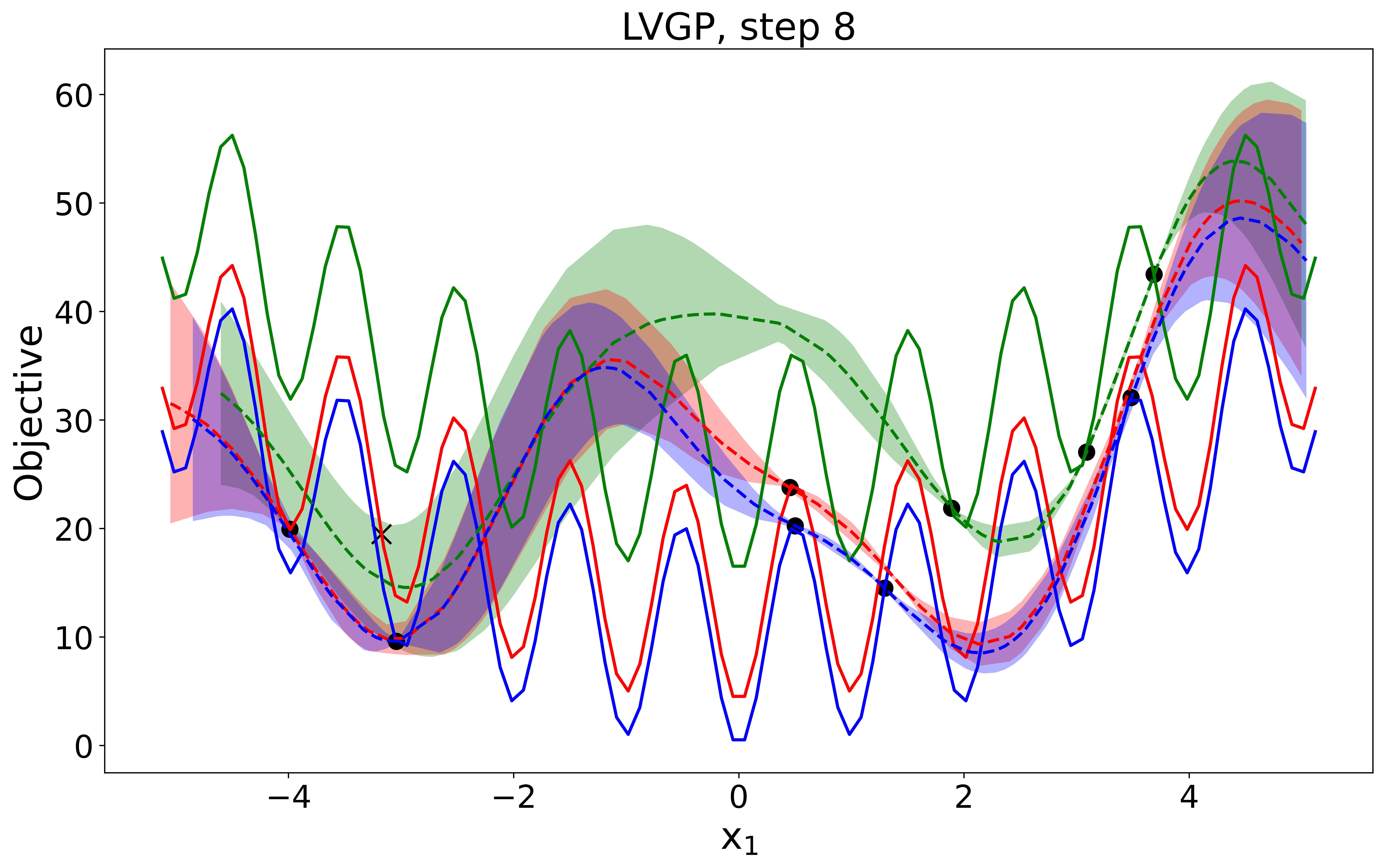

Supplement: Supplementary file 1 — Supplementary Information 1. [file 41598_2022_23431_MOESM1_ESM.zip › Sampling_Sequence_Figures/Rastrigin_Function/rastrigin2_LVGP_8.jpg]

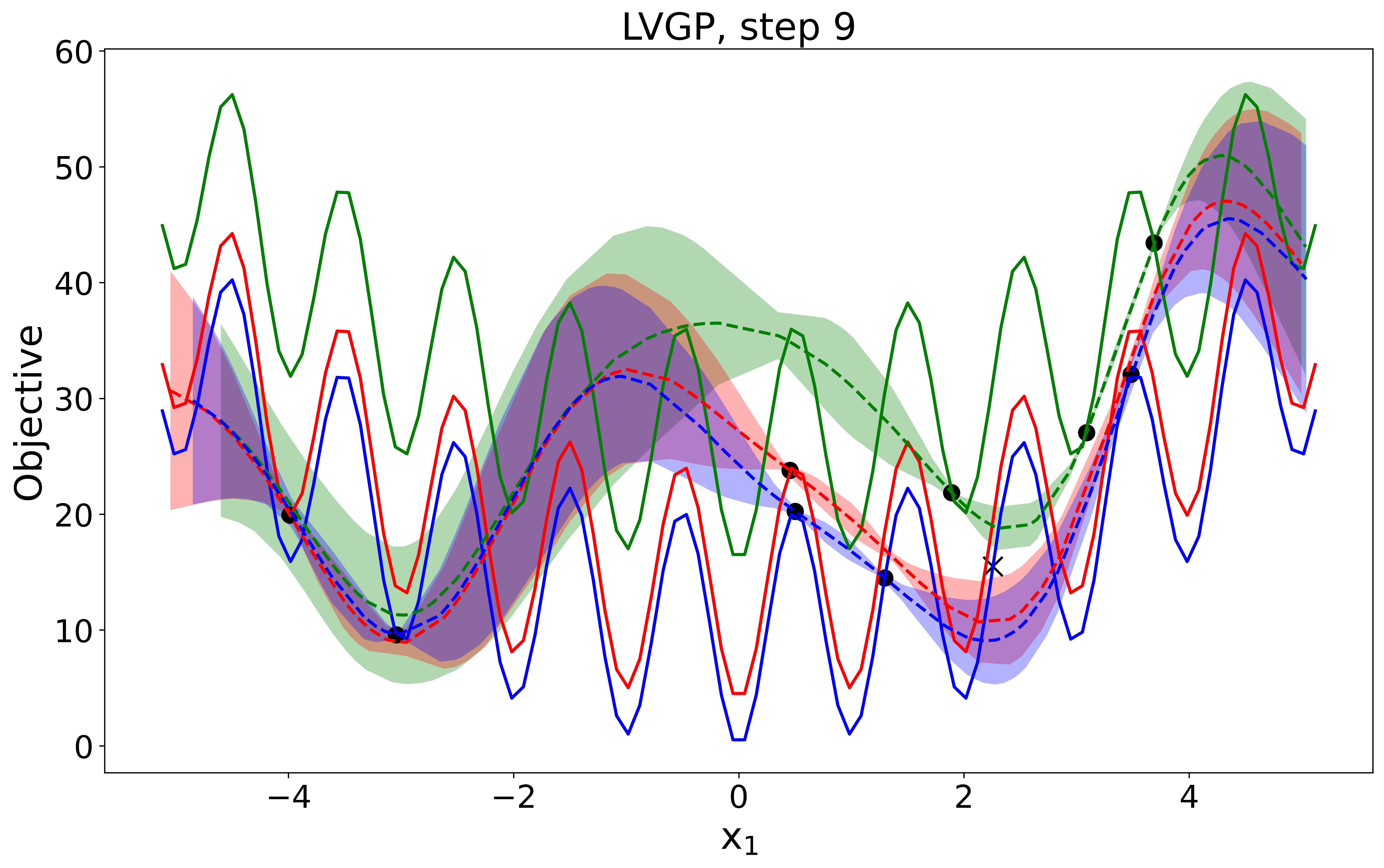

Supplement: Supplementary file 1 — Supplementary Information 1. [file 41598_2022_23431_MOESM1_ESM.zip › Sampling_Sequence_Figures/Rastrigin_Function/rastrigin2_LVGP_9.jpg]
