# Supplementary figures and images for: Uncertainty-aware mixed-variable machine learning for materials design (part 1 of 2)
Source: Sci Rep. 2022 Nov 17;12:19760. doi: 10.1038/s41598-022-23431-2 (PMC9672324; doi:10.1038/s41598-022-23431-2)

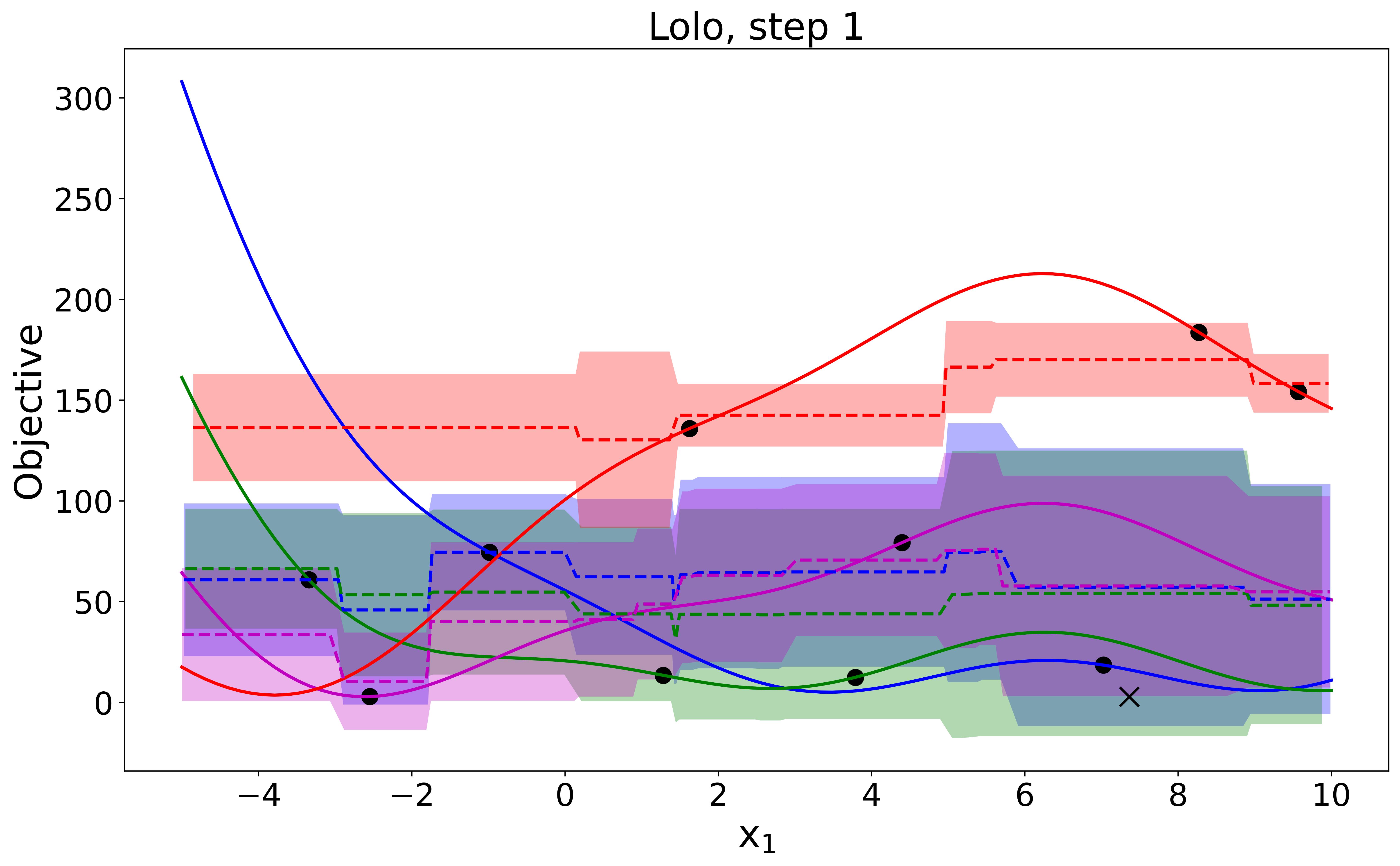

Supplement: Supplementary file 1 — Supplementary Information 1. [file 41598_2022_23431_MOESM1_ESM.zip › Sampling_Sequence_Figures/Branin_Function/branin_Lolo_1.jpg]

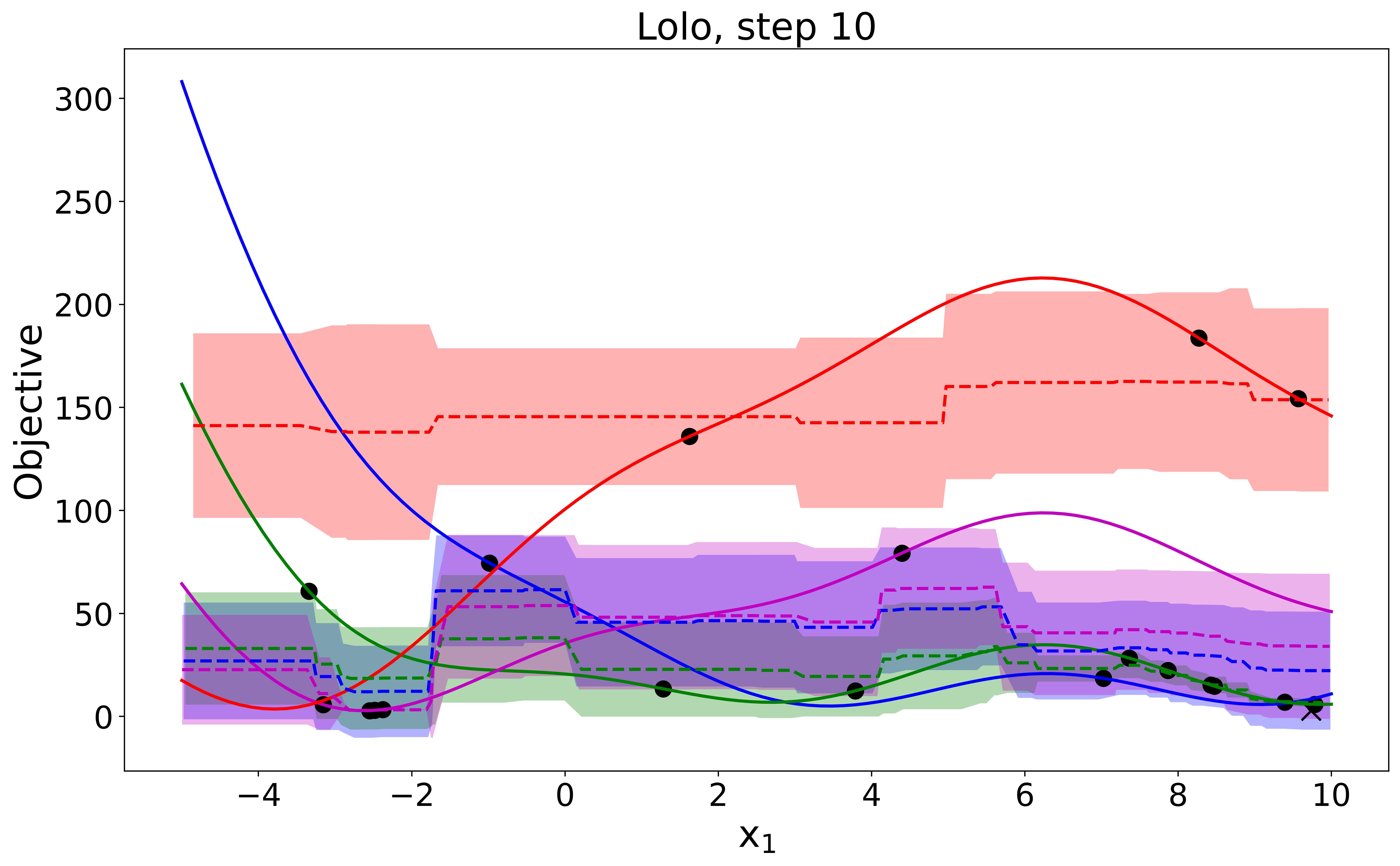

Supplement: Supplementary file 1 — Supplementary Information 1. [file 41598_2022_23431_MOESM1_ESM.zip › Sampling_Sequence_Figures/Branin_Function/branin_Lolo_10.jpg]

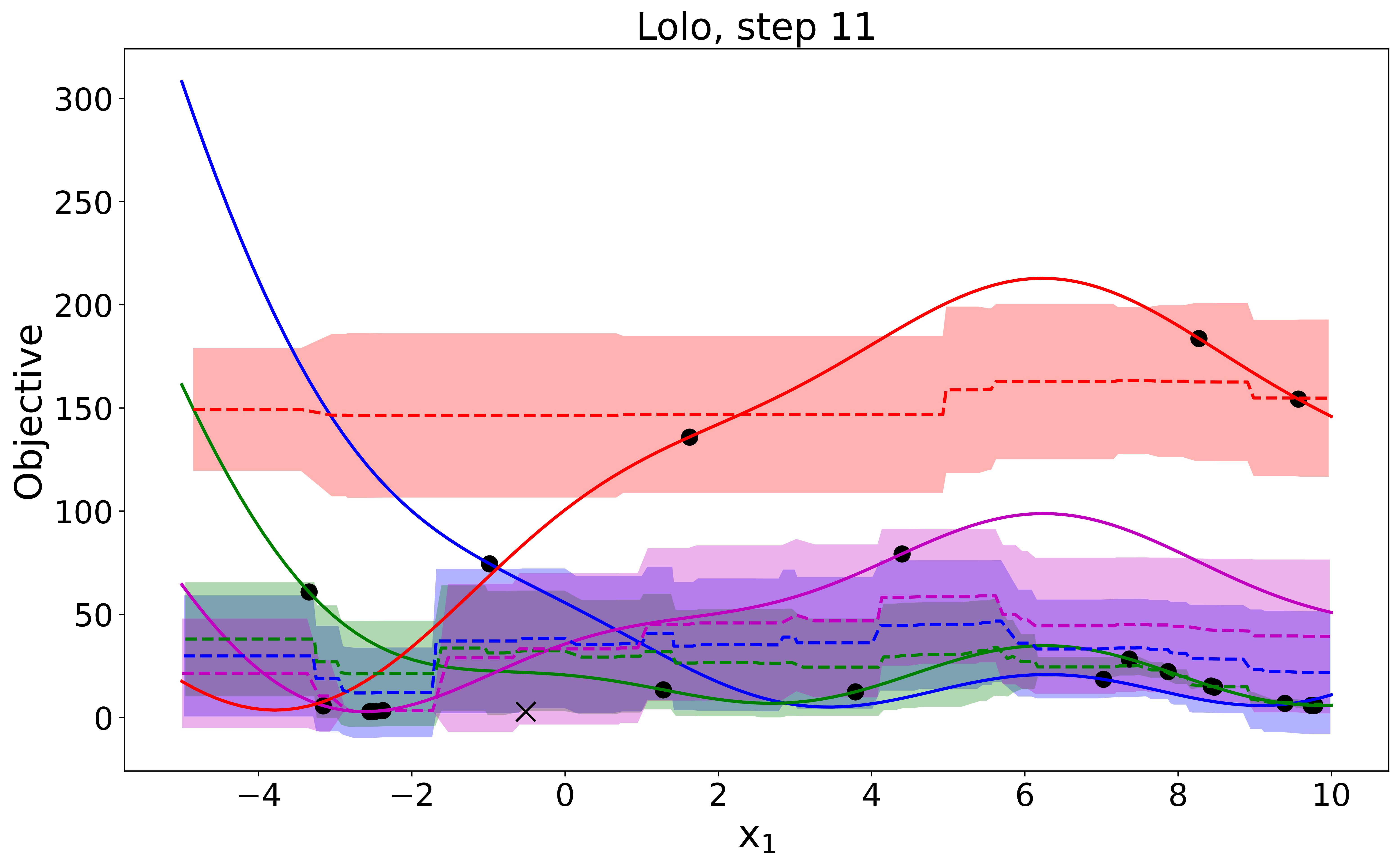

Supplement: Supplementary file 1 — Supplementary Information 1. [file 41598_2022_23431_MOESM1_ESM.zip › Sampling_Sequence_Figures/Branin_Function/branin_Lolo_11.jpg]

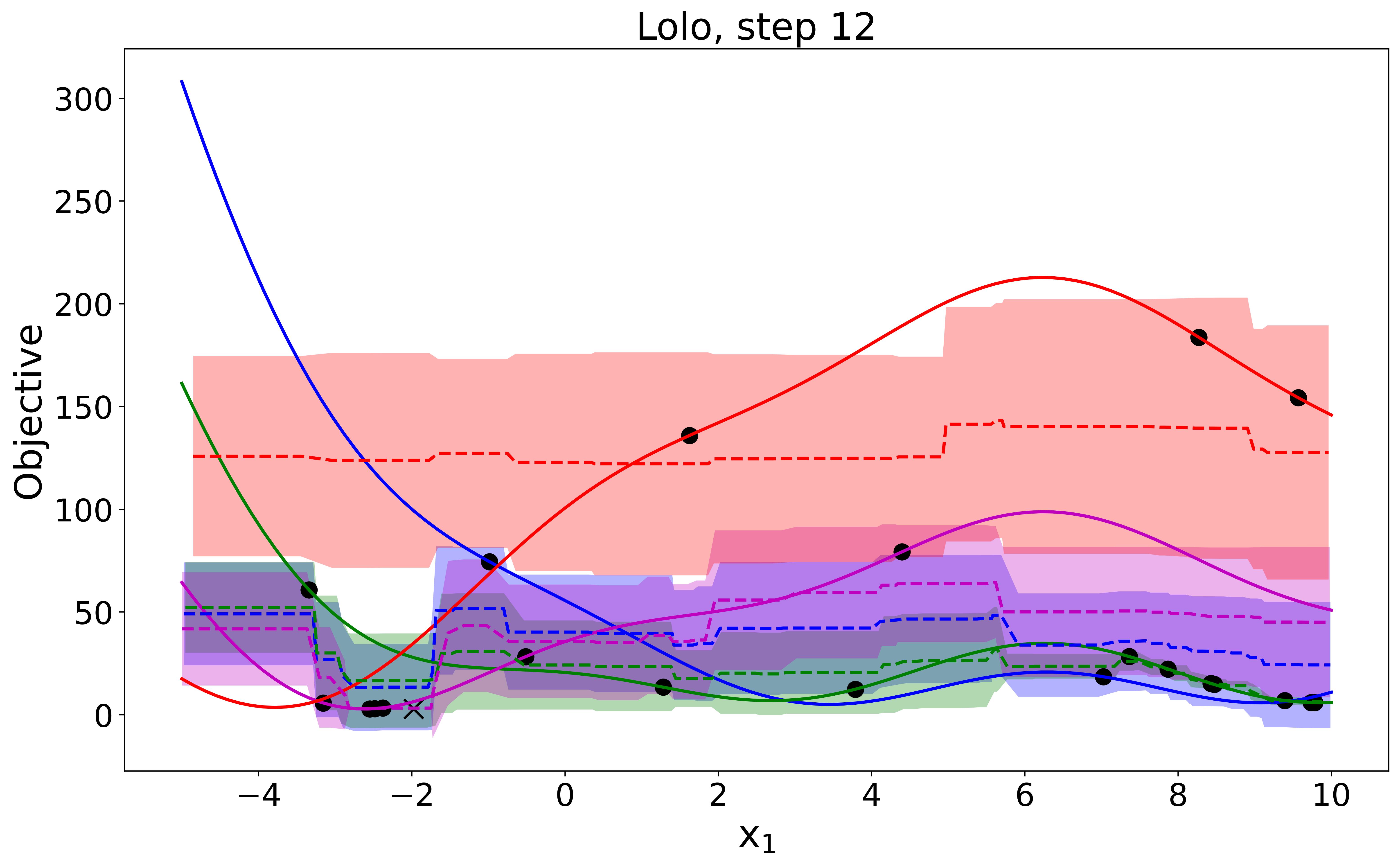

Supplement: Supplementary file 1 — Supplementary Information 1. [file 41598_2022_23431_MOESM1_ESM.zip › Sampling_Sequence_Figures/Branin_Function/branin_Lolo_12.jpg]

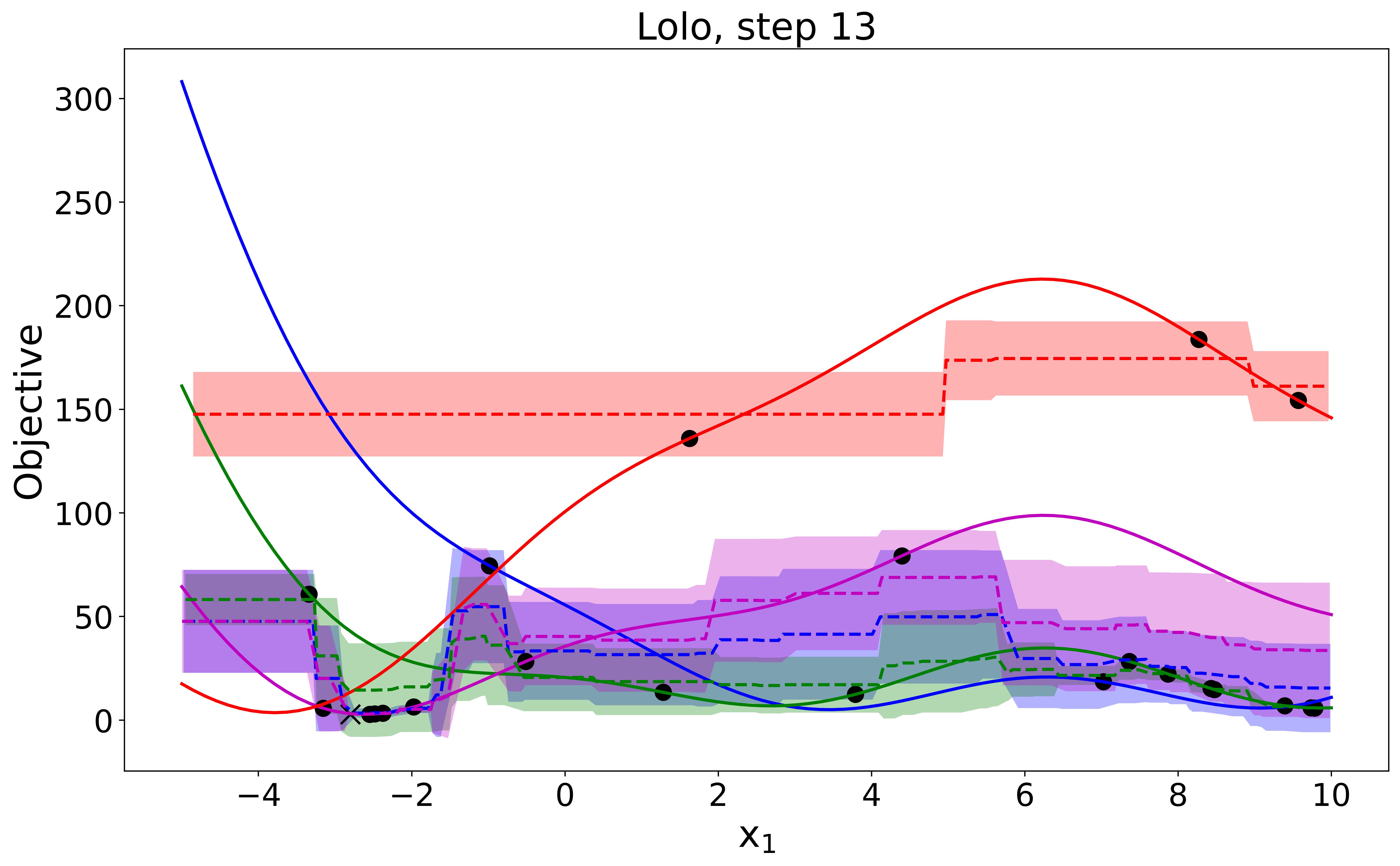

Supplement: Supplementary file 1 — Supplementary Information 1. [file 41598_2022_23431_MOESM1_ESM.zip › Sampling_Sequence_Figures/Branin_Function/branin_Lolo_13.jpg]

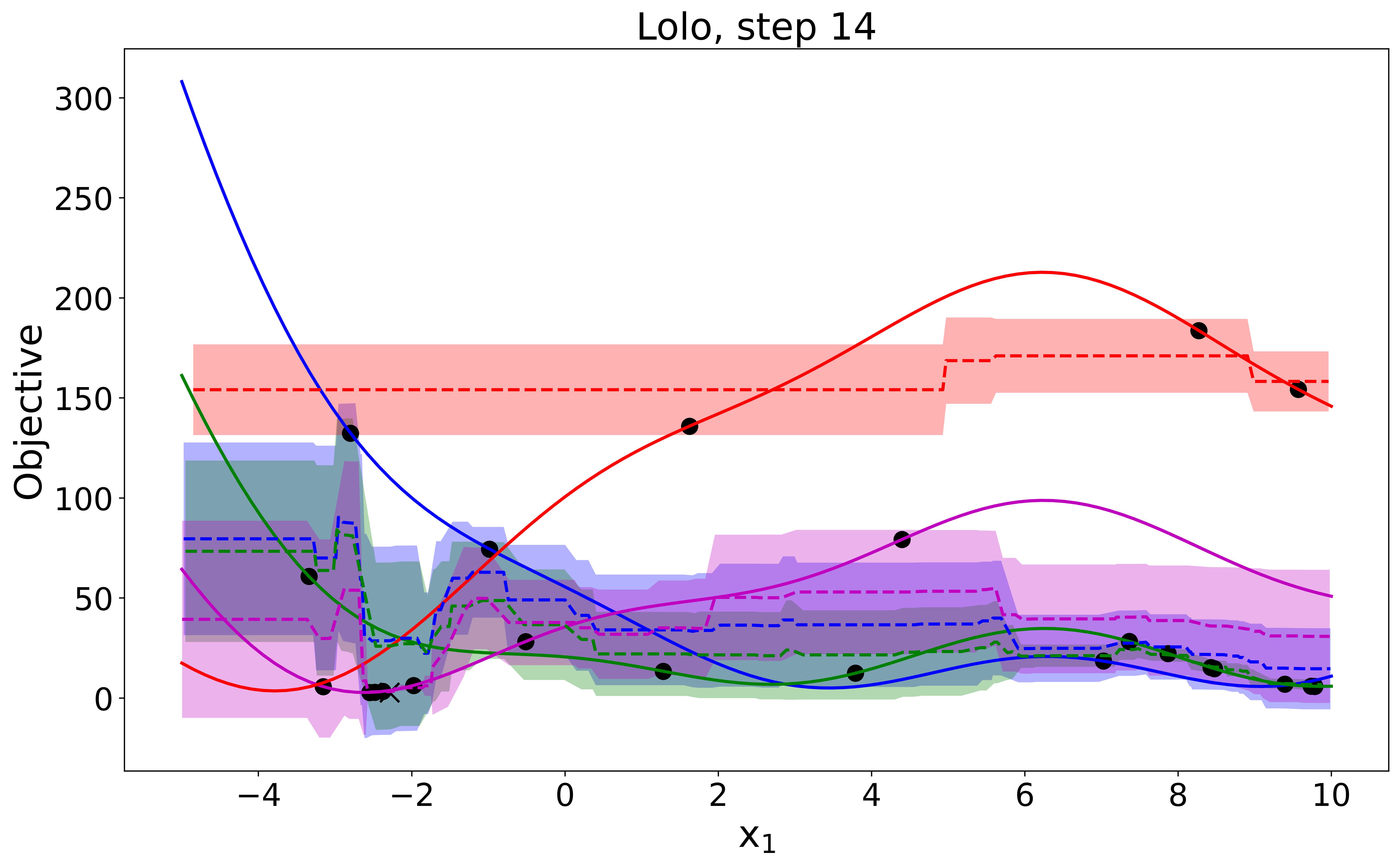

Supplement: Supplementary file 1 — Supplementary Information 1. [file 41598_2022_23431_MOESM1_ESM.zip › Sampling_Sequence_Figures/Branin_Function/branin_Lolo_14.jpg]

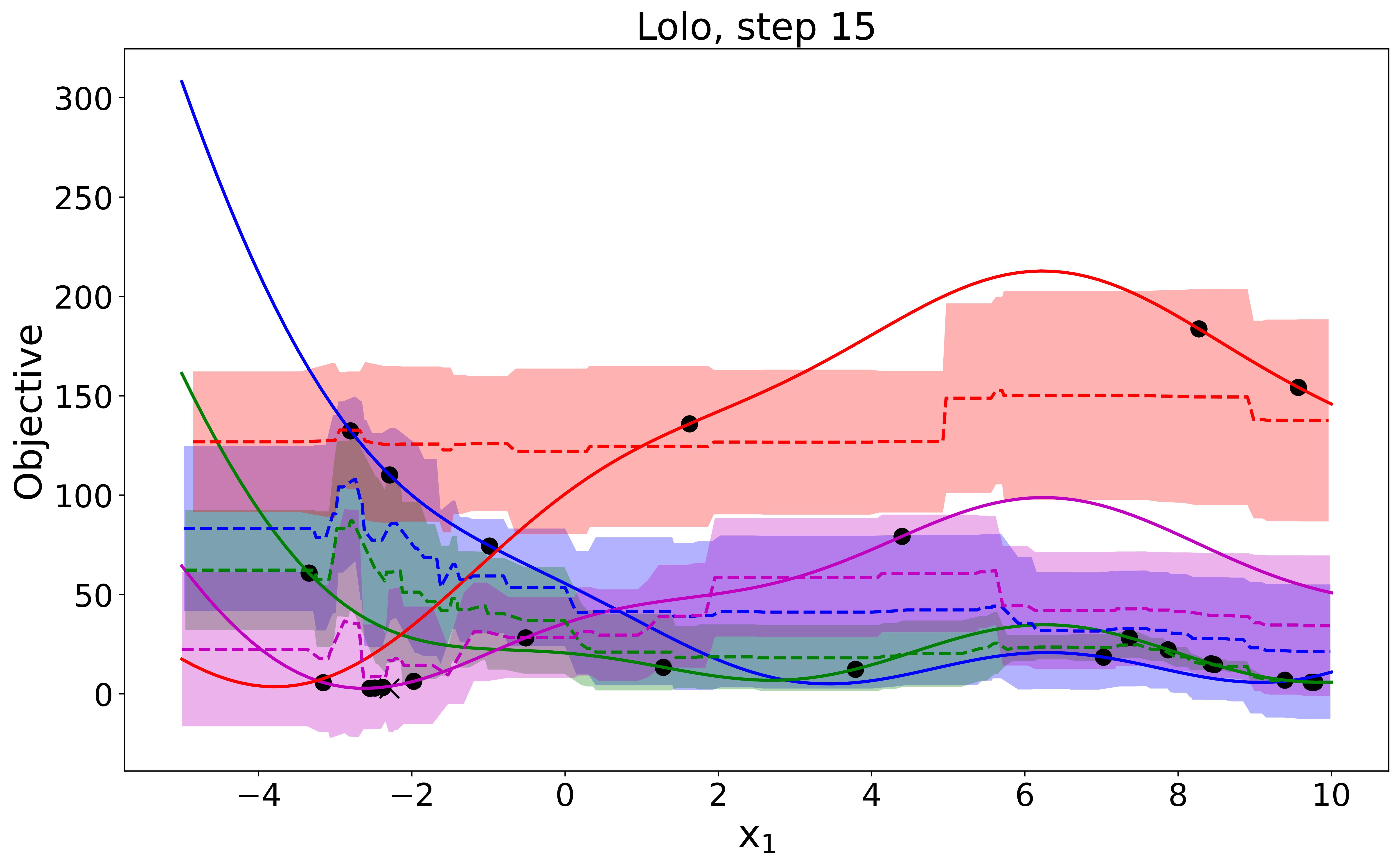

Supplement: Supplementary file 1 — Supplementary Information 1. [file 41598_2022_23431_MOESM1_ESM.zip › Sampling_Sequence_Figures/Branin_Function/branin_Lolo_15.jpg]

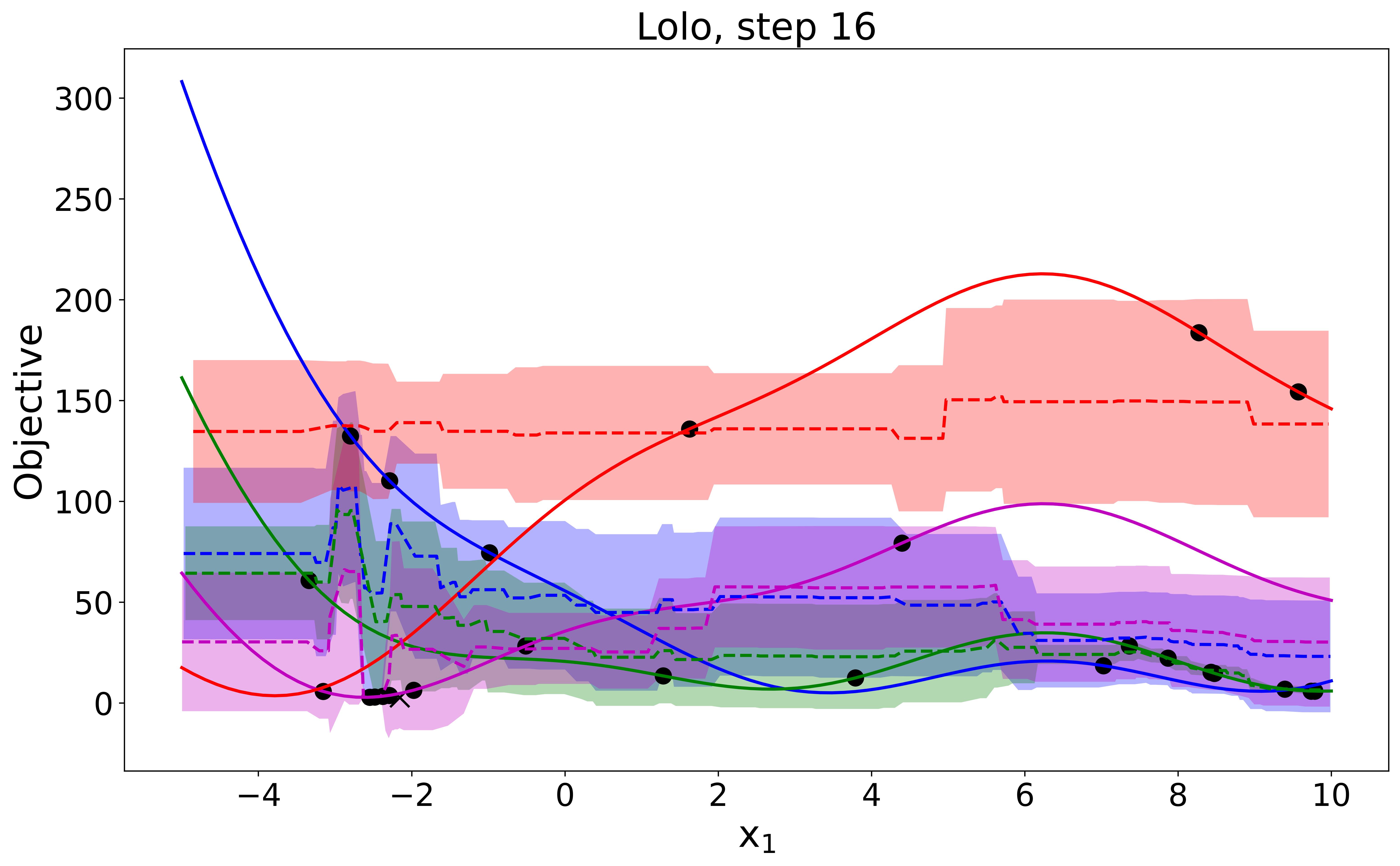

Supplement: Supplementary file 1 — Supplementary Information 1. [file 41598_2022_23431_MOESM1_ESM.zip › Sampling_Sequence_Figures/Branin_Function/branin_Lolo_16.jpg]

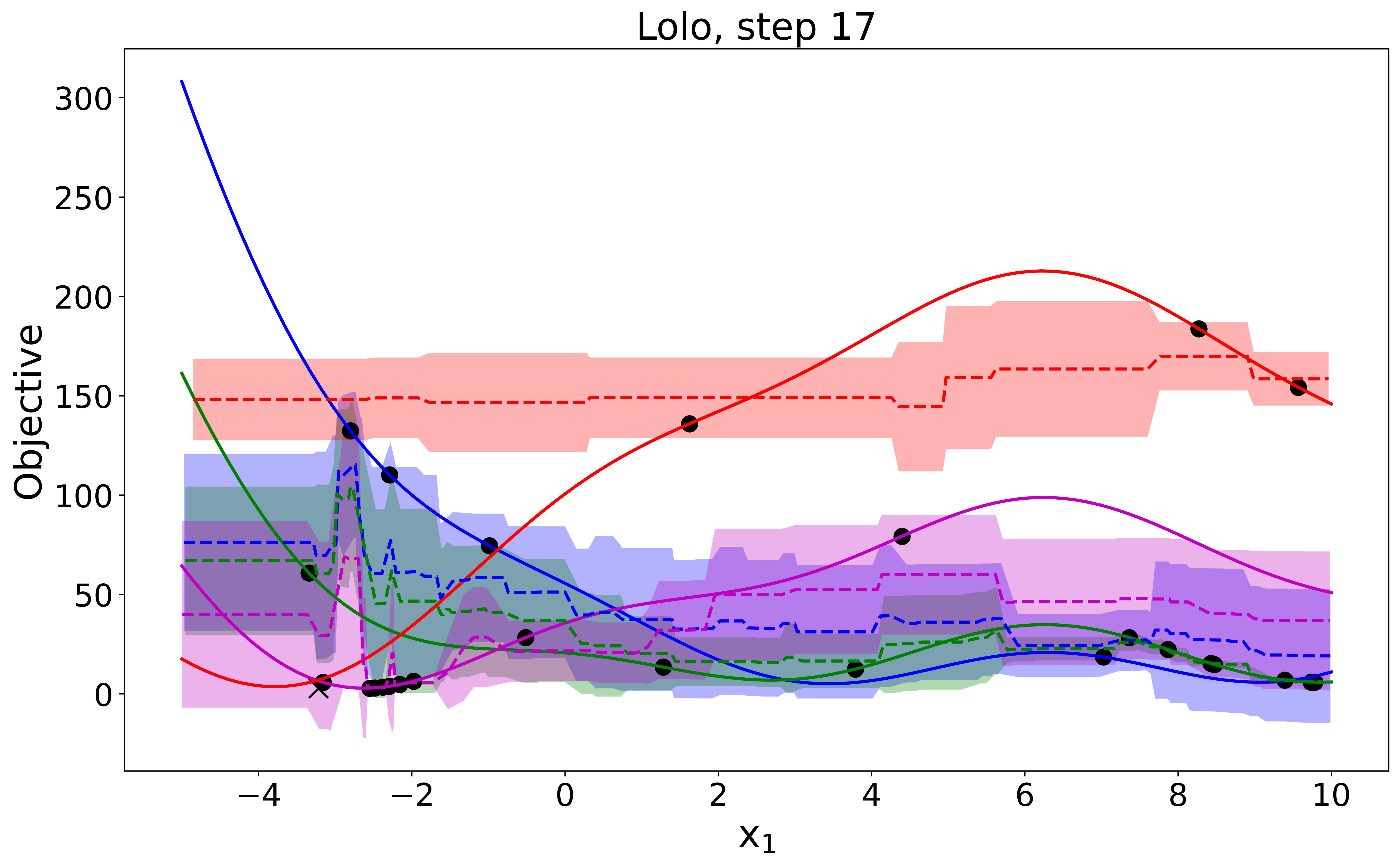

Supplement: Supplementary file 1 — Supplementary Information 1. [file 41598_2022_23431_MOESM1_ESM.zip › Sampling_Sequence_Figures/Branin_Function/branin_Lolo_17.jpg]

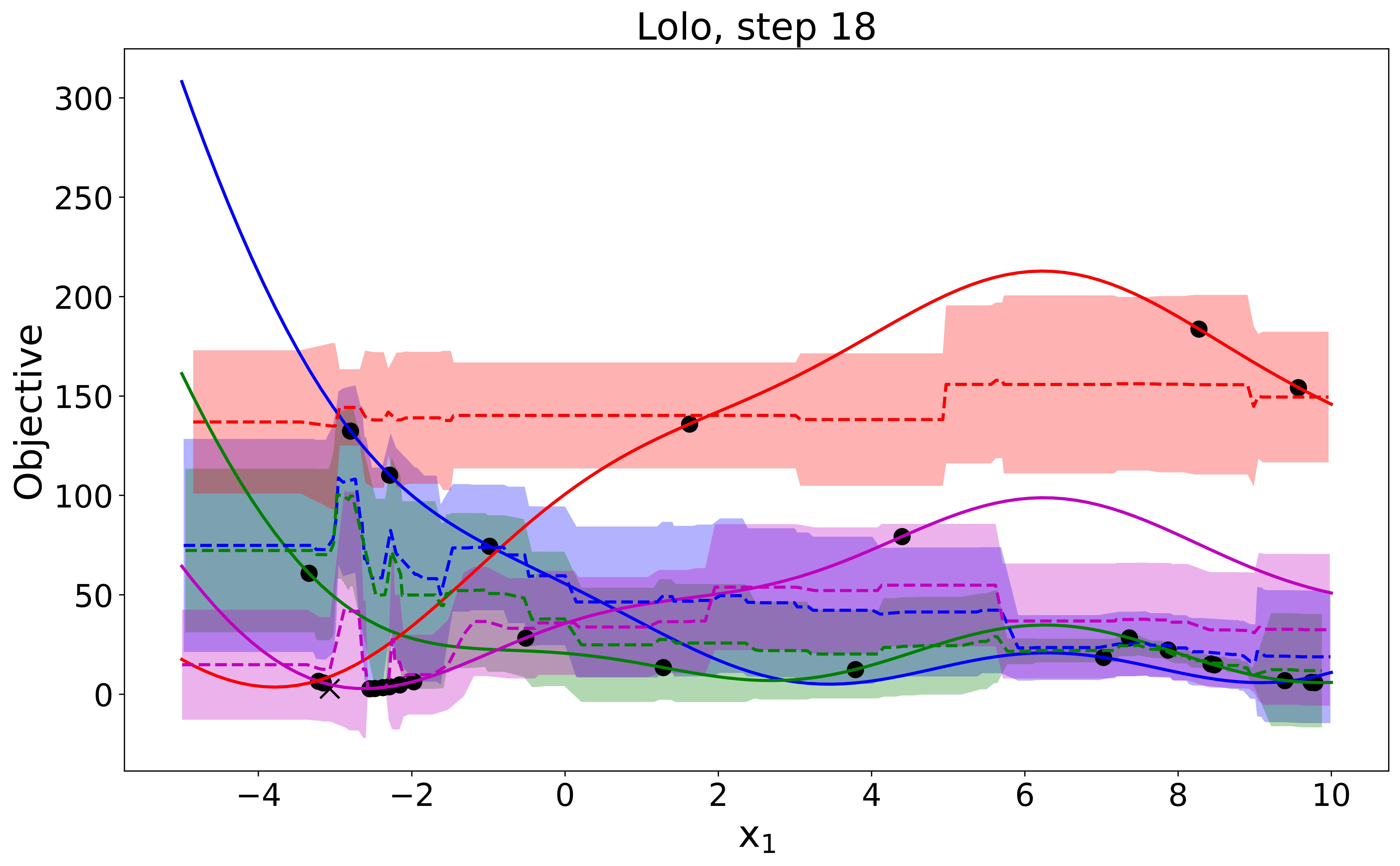

Supplement: Supplementary file 1 — Supplementary Information 1. [file 41598_2022_23431_MOESM1_ESM.zip › Sampling_Sequence_Figures/Branin_Function/branin_Lolo_18.jpg]

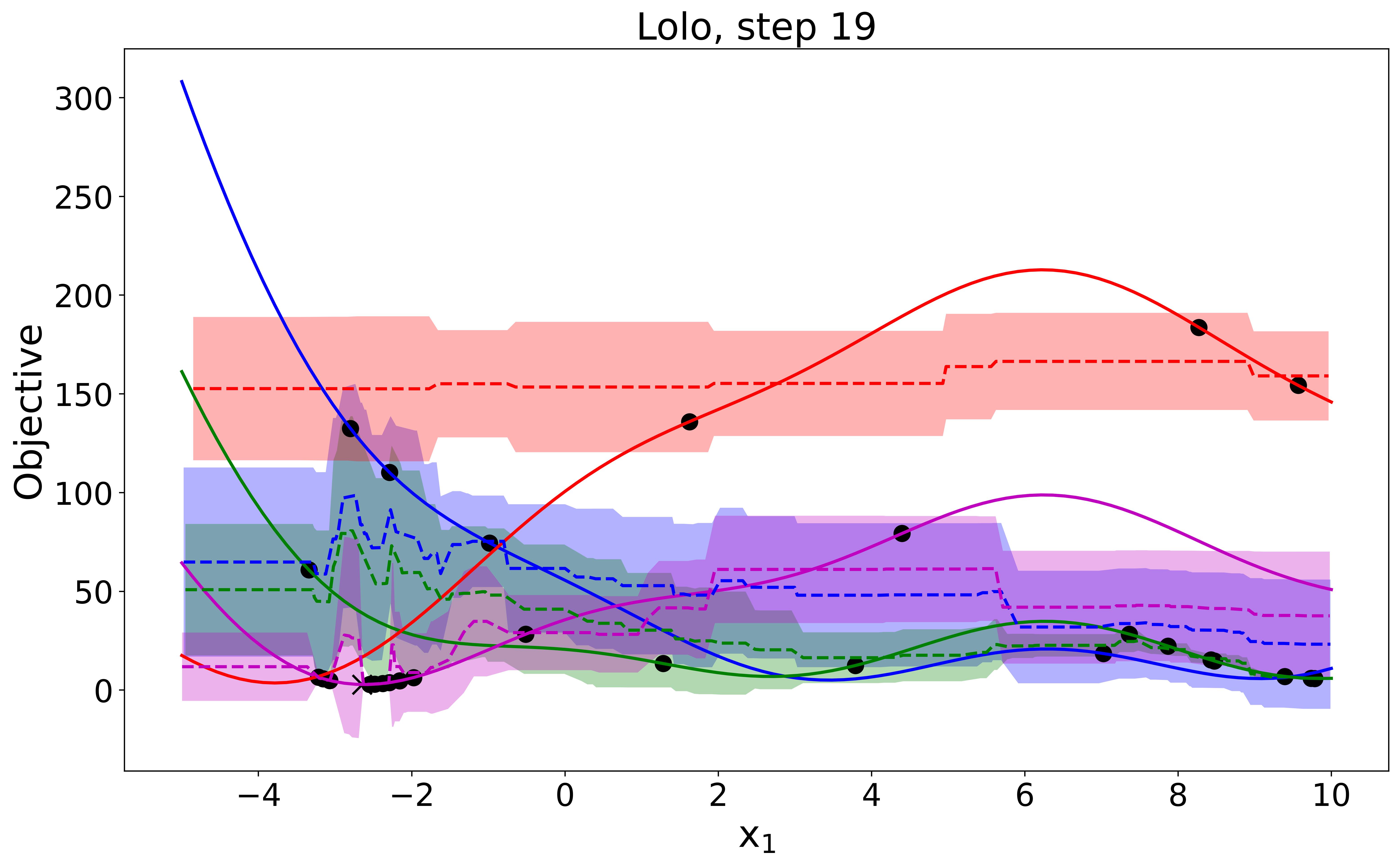

Supplement: Supplementary file 1 — Supplementary Information 1. [file 41598_2022_23431_MOESM1_ESM.zip › Sampling_Sequence_Figures/Branin_Function/branin_Lolo_19.jpg]

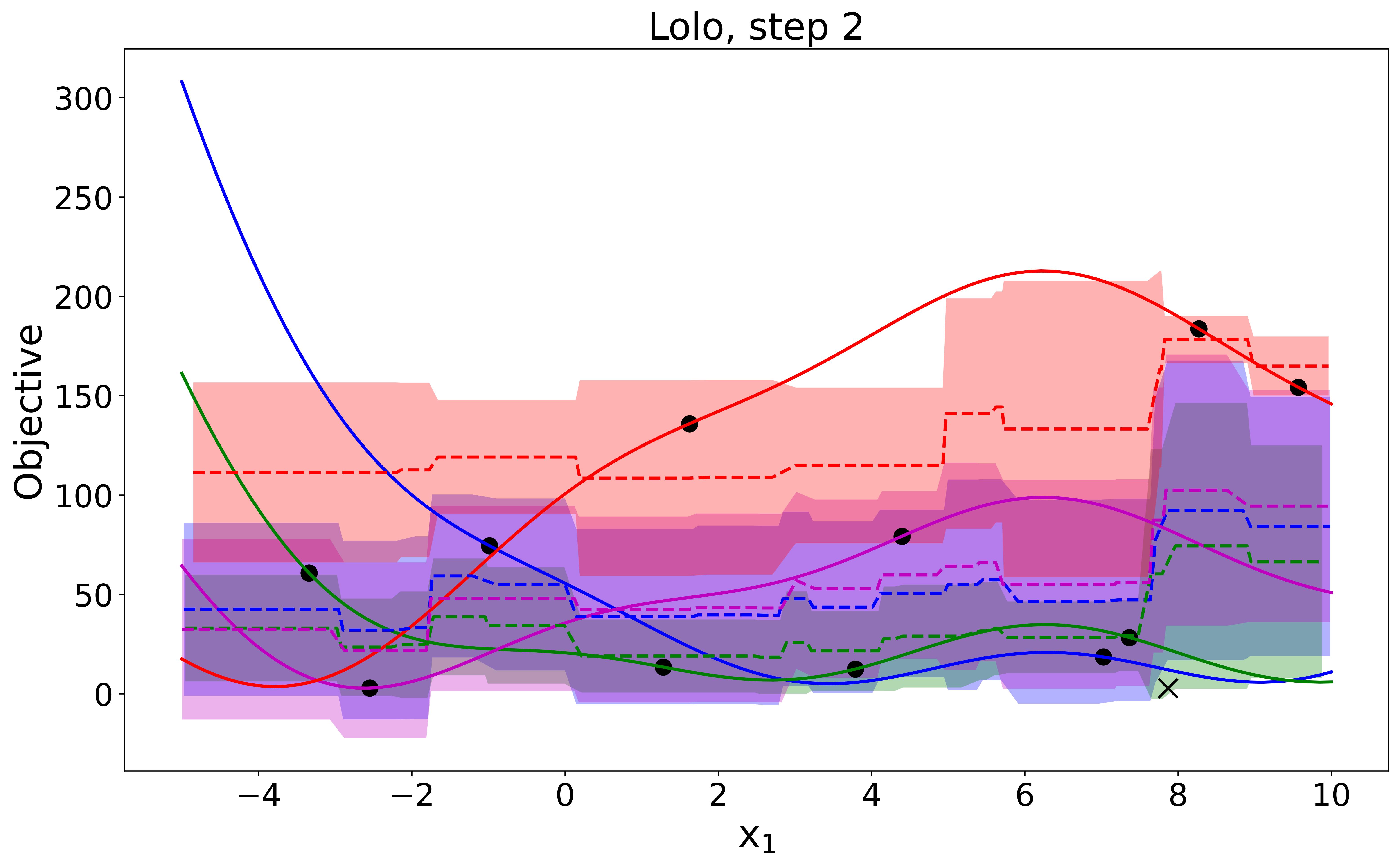

Supplement: Supplementary file 1 — Supplementary Information 1. [file 41598_2022_23431_MOESM1_ESM.zip › Sampling_Sequence_Figures/Branin_Function/branin_Lolo_2.jpg]

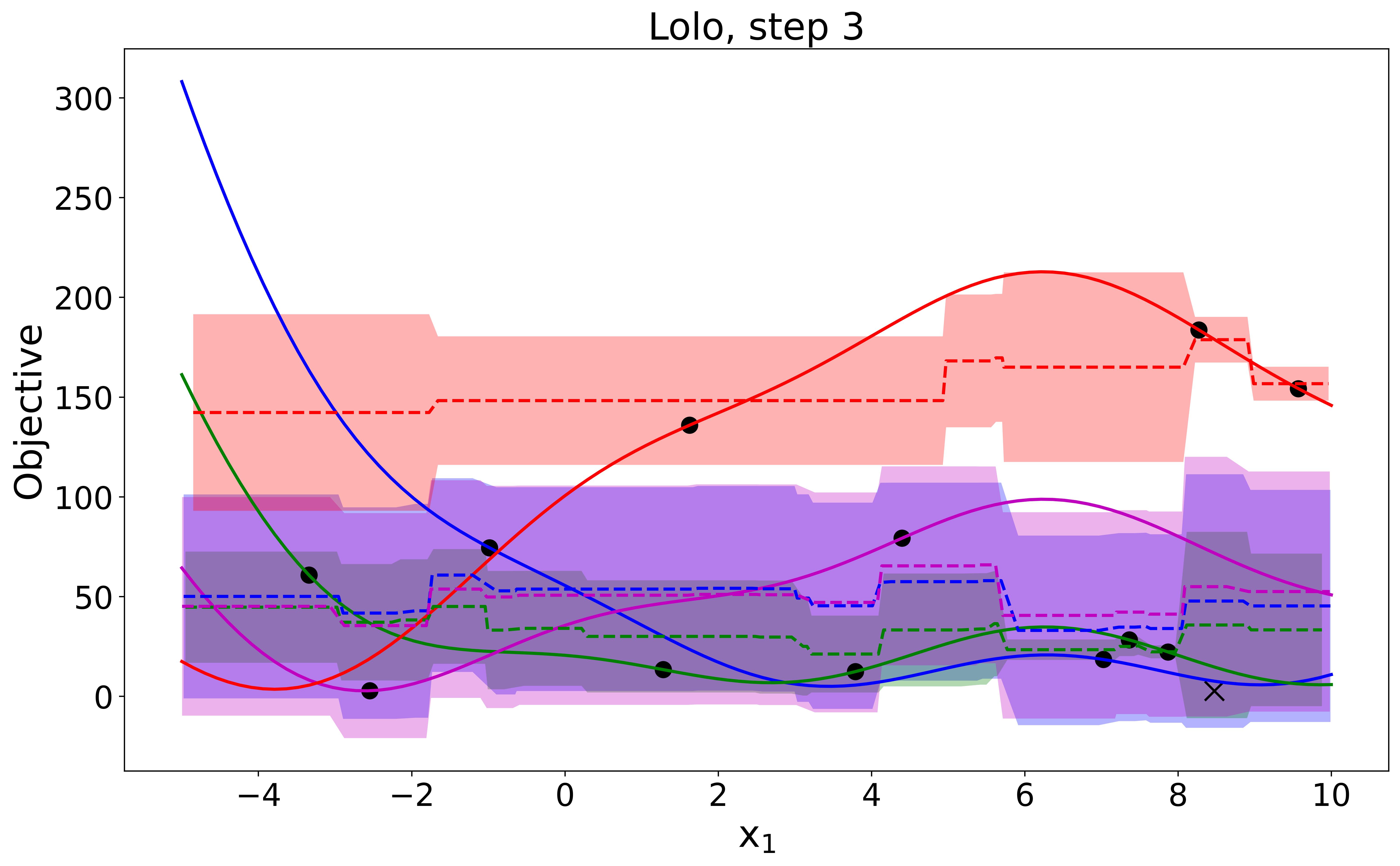

Supplement: Supplementary file 1 — Supplementary Information 1. [file 41598_2022_23431_MOESM1_ESM.zip › Sampling_Sequence_Figures/Branin_Function/branin_Lolo_3.jpg]

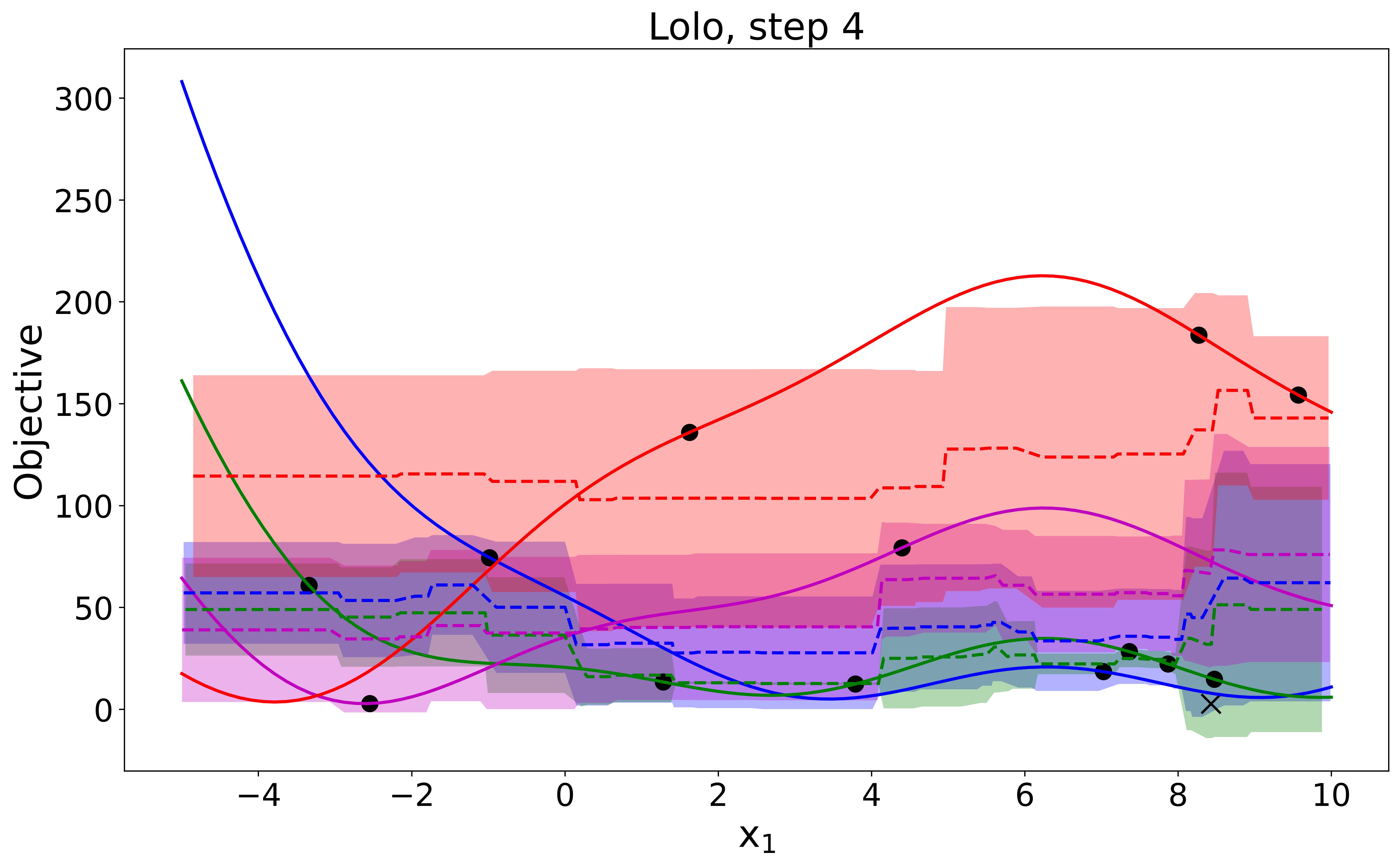

Supplement: Supplementary file 1 — Supplementary Information 1. [file 41598_2022_23431_MOESM1_ESM.zip › Sampling_Sequence_Figures/Branin_Function/branin_Lolo_4.jpg]

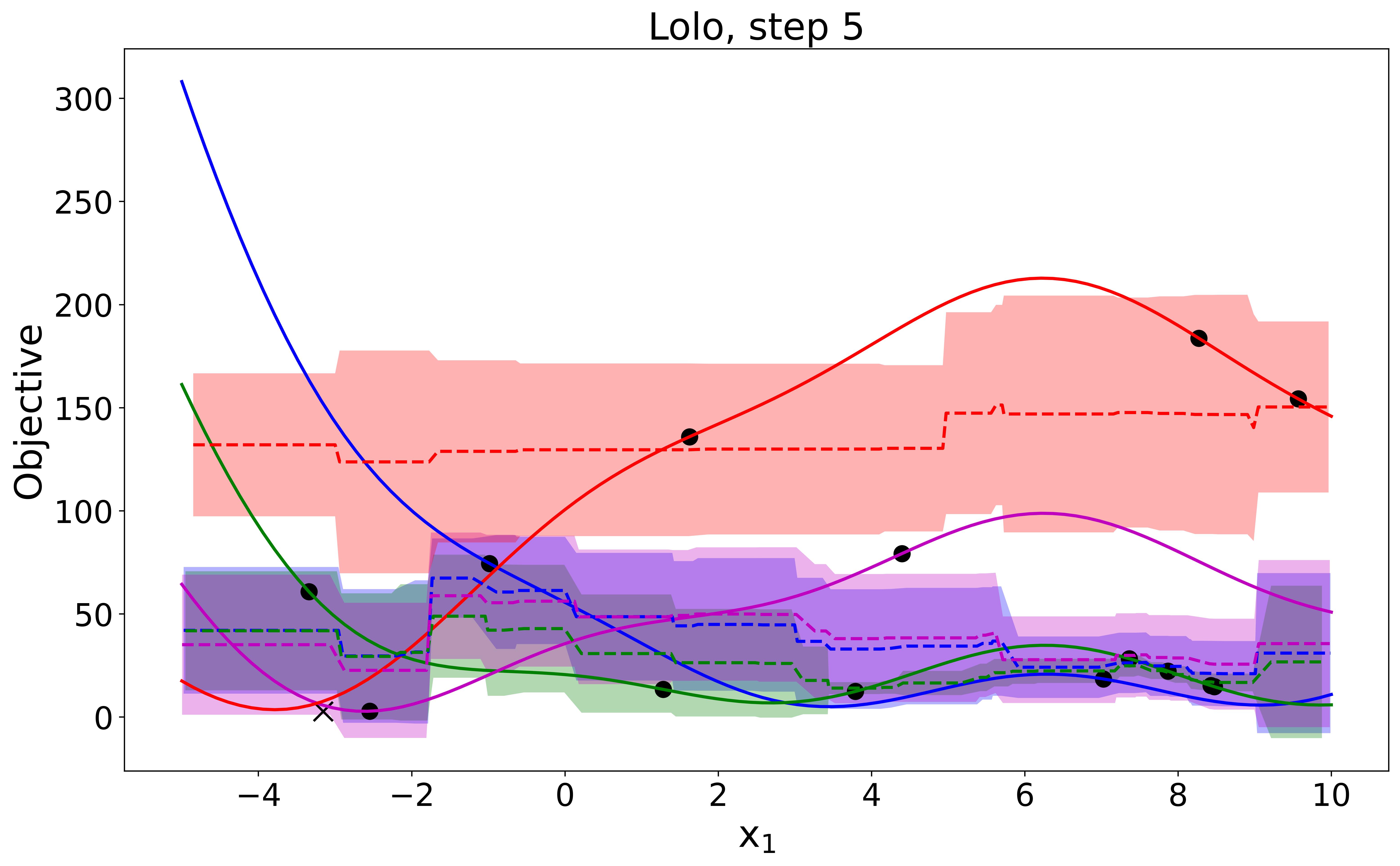

Supplement: Supplementary file 1 — Supplementary Information 1. [file 41598_2022_23431_MOESM1_ESM.zip › Sampling_Sequence_Figures/Branin_Function/branin_Lolo_5.jpg]

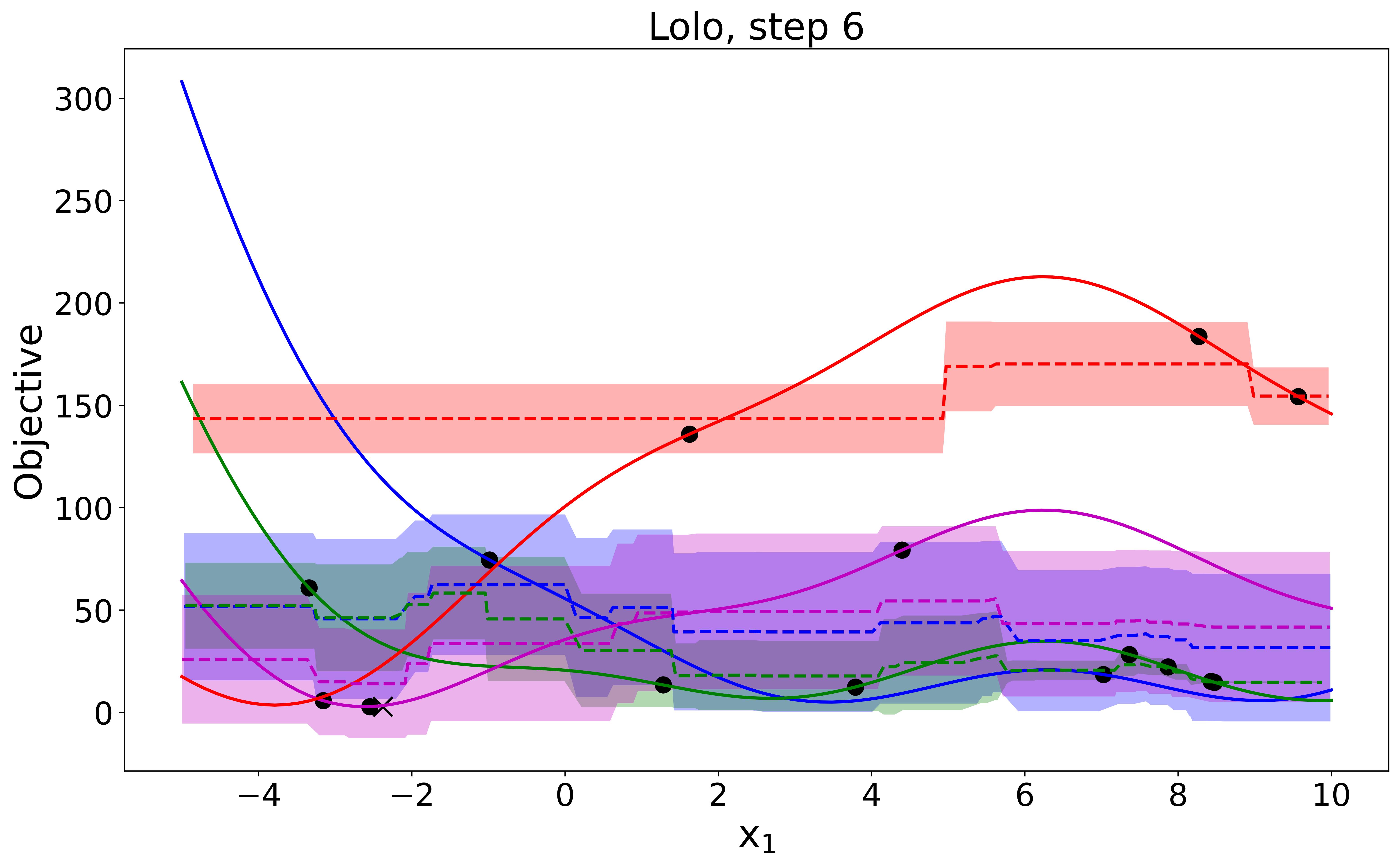

Supplement: Supplementary file 1 — Supplementary Information 1. [file 41598_2022_23431_MOESM1_ESM.zip › Sampling_Sequence_Figures/Branin_Function/branin_Lolo_6.jpg]

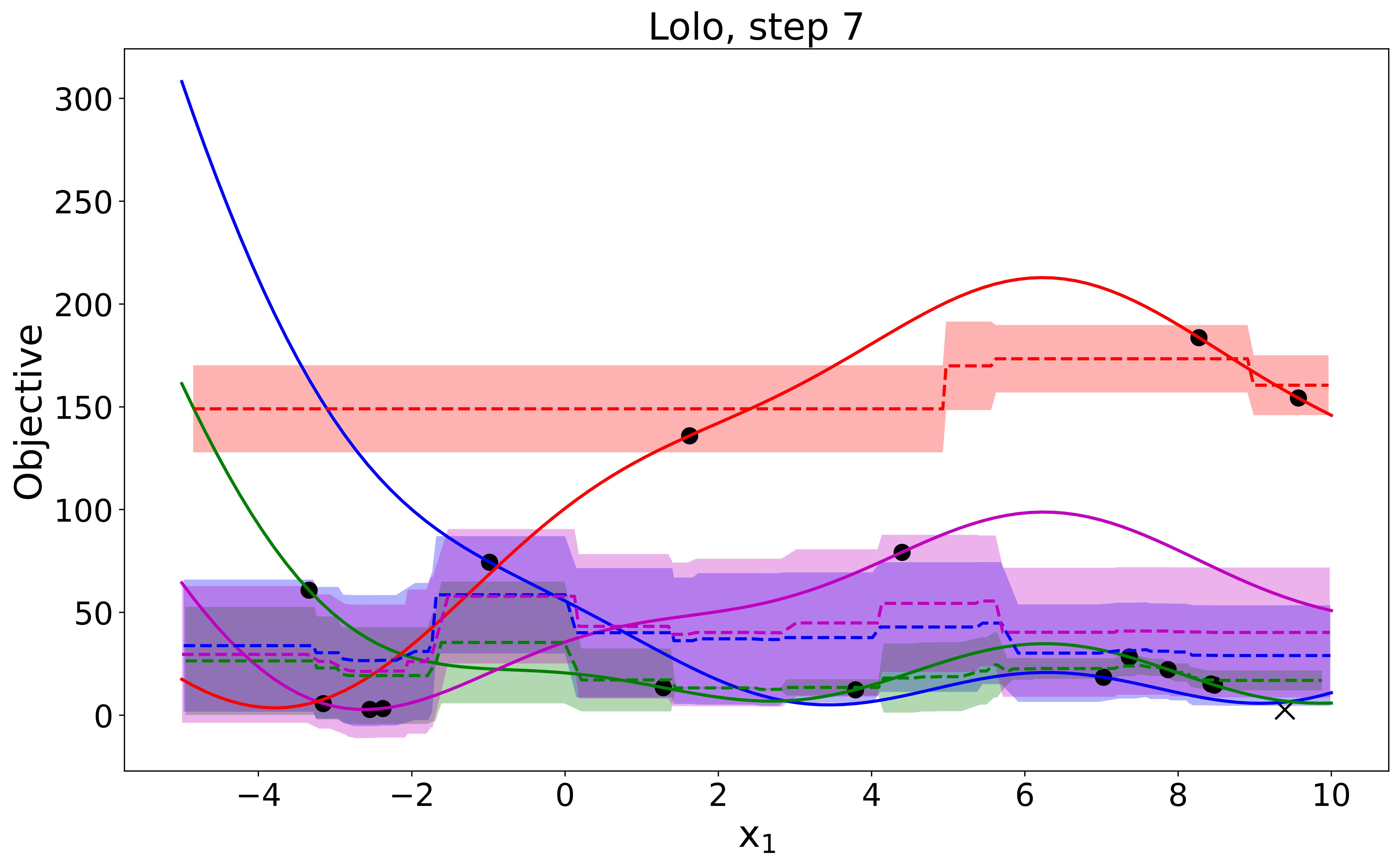

Supplement: Supplementary file 1 — Supplementary Information 1. [file 41598_2022_23431_MOESM1_ESM.zip › Sampling_Sequence_Figures/Branin_Function/branin_Lolo_7.jpg]

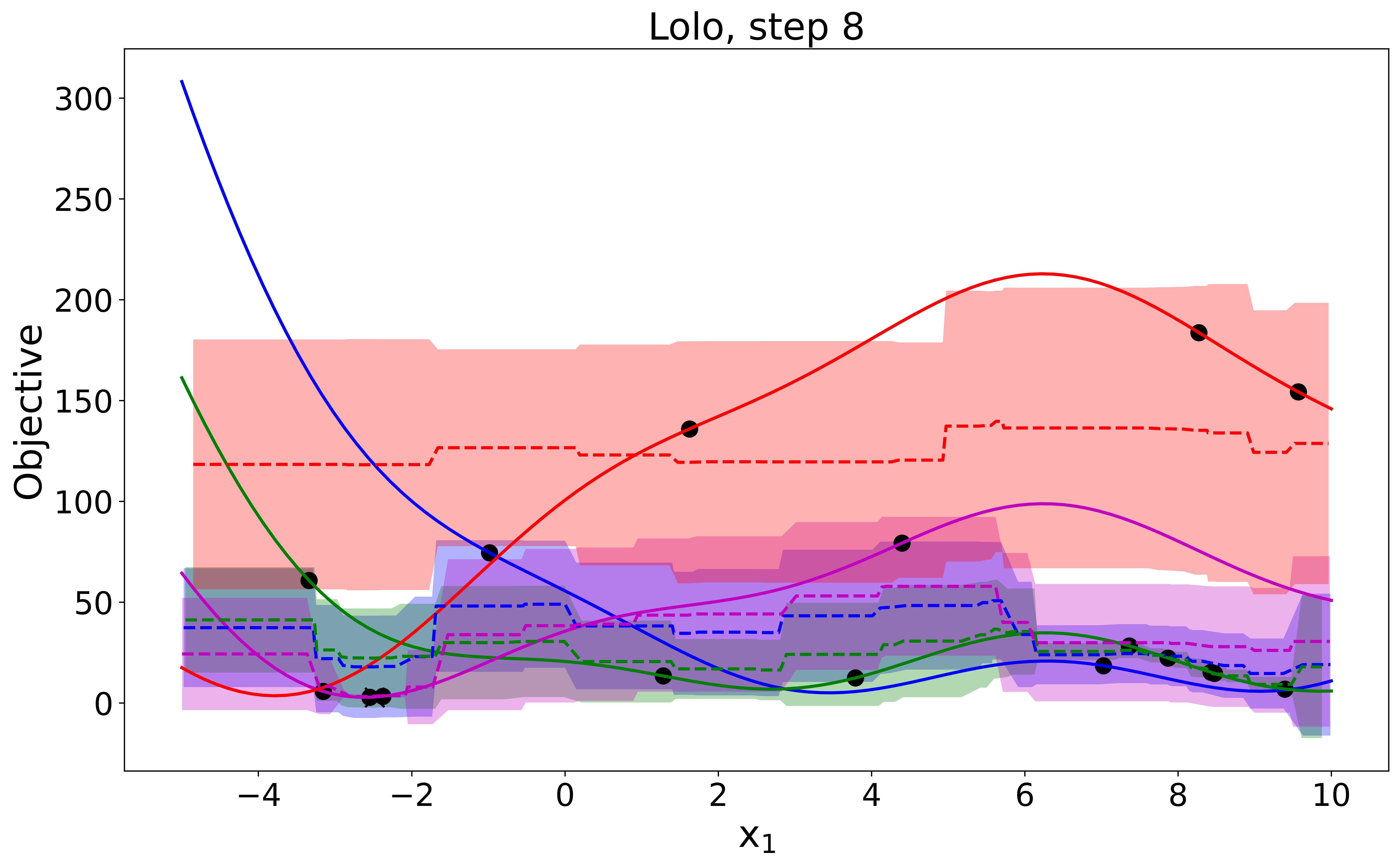

Supplement: Supplementary file 1 — Supplementary Information 1. [file 41598_2022_23431_MOESM1_ESM.zip › Sampling_Sequence_Figures/Branin_Function/branin_Lolo_8.jpg]

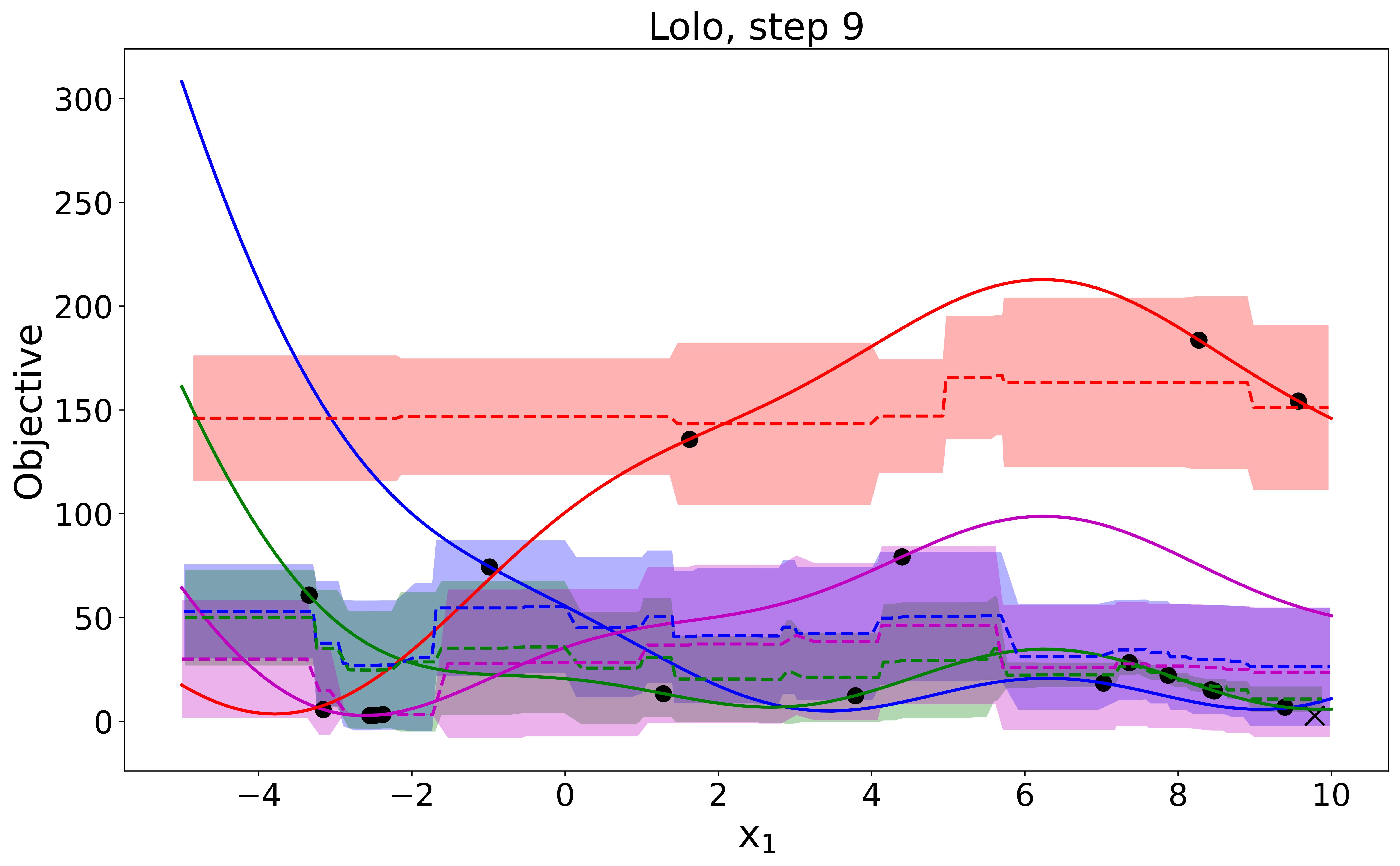

Supplement: Supplementary file 1 — Supplementary Information 1. [file 41598_2022_23431_MOESM1_ESM.zip › Sampling_Sequence_Figures/Branin_Function/branin_Lolo_9.jpg]

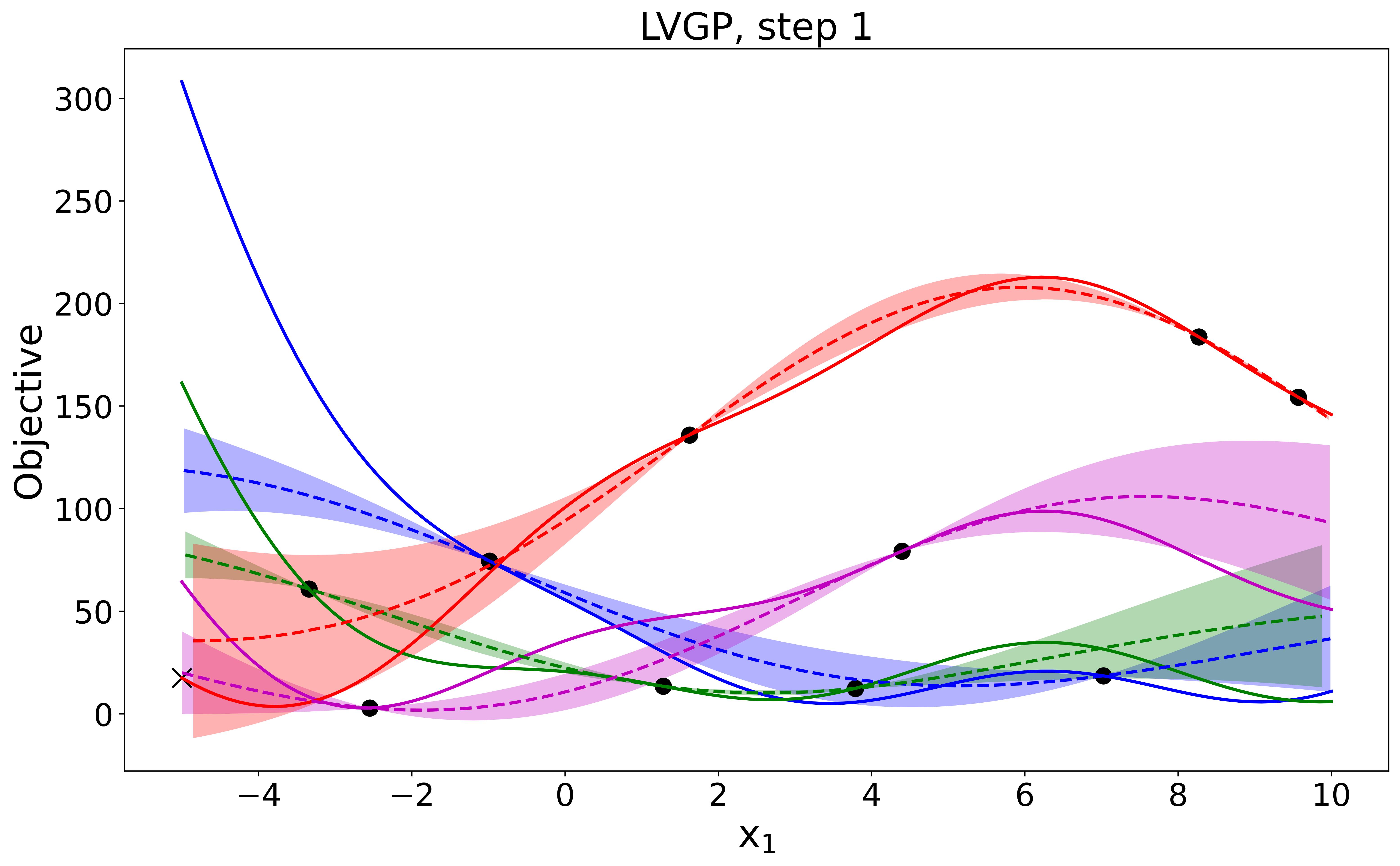

Supplement: Supplementary file 1 — Supplementary Information 1. [file 41598_2022_23431_MOESM1_ESM.zip › Sampling_Sequence_Figures/Branin_Function/branin_LVGP_1.jpg]

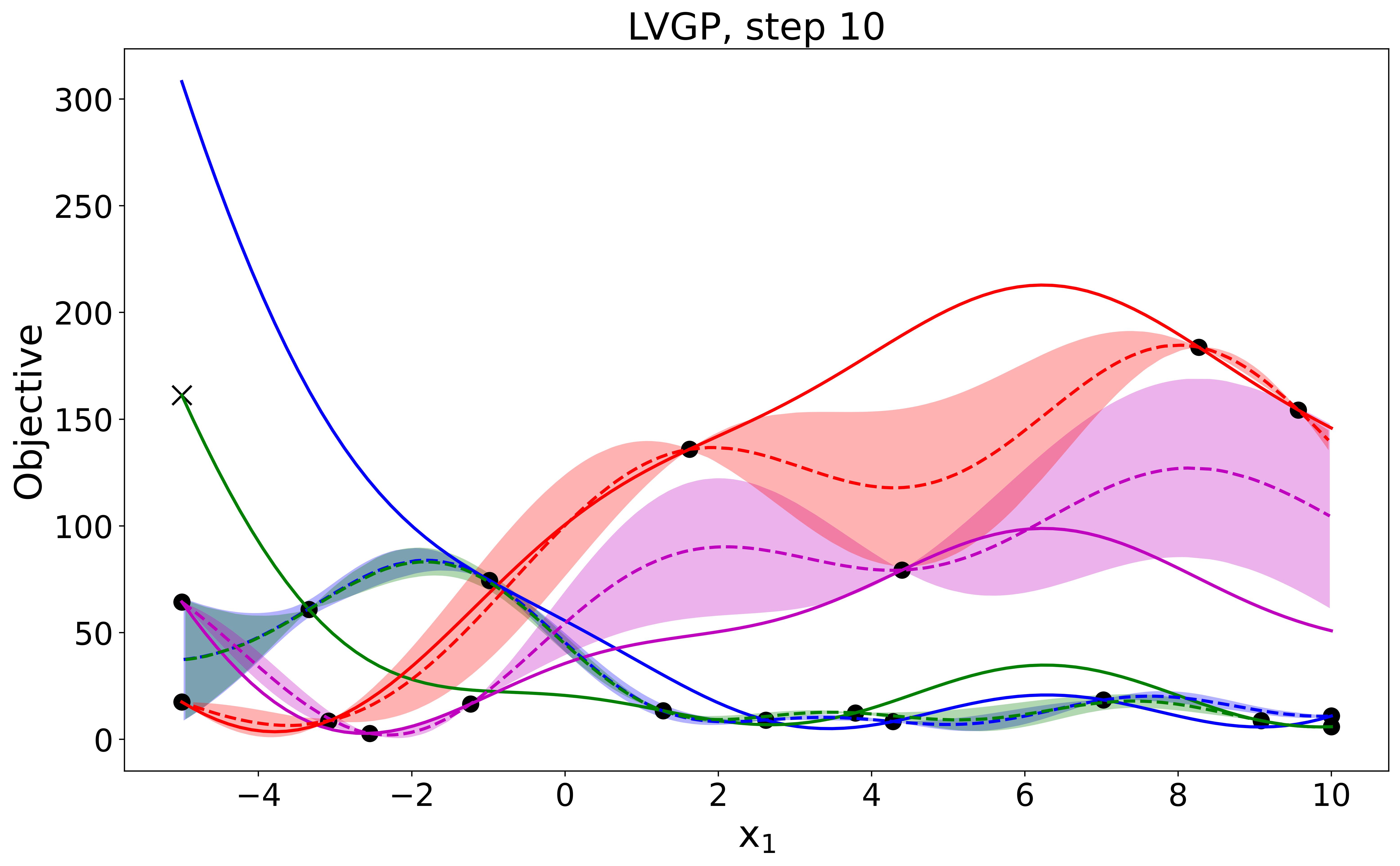

Supplement: Supplementary file 1 — Supplementary Information 1. [file 41598_2022_23431_MOESM1_ESM.zip › Sampling_Sequence_Figures/Branin_Function/branin_LVGP_10.jpg]

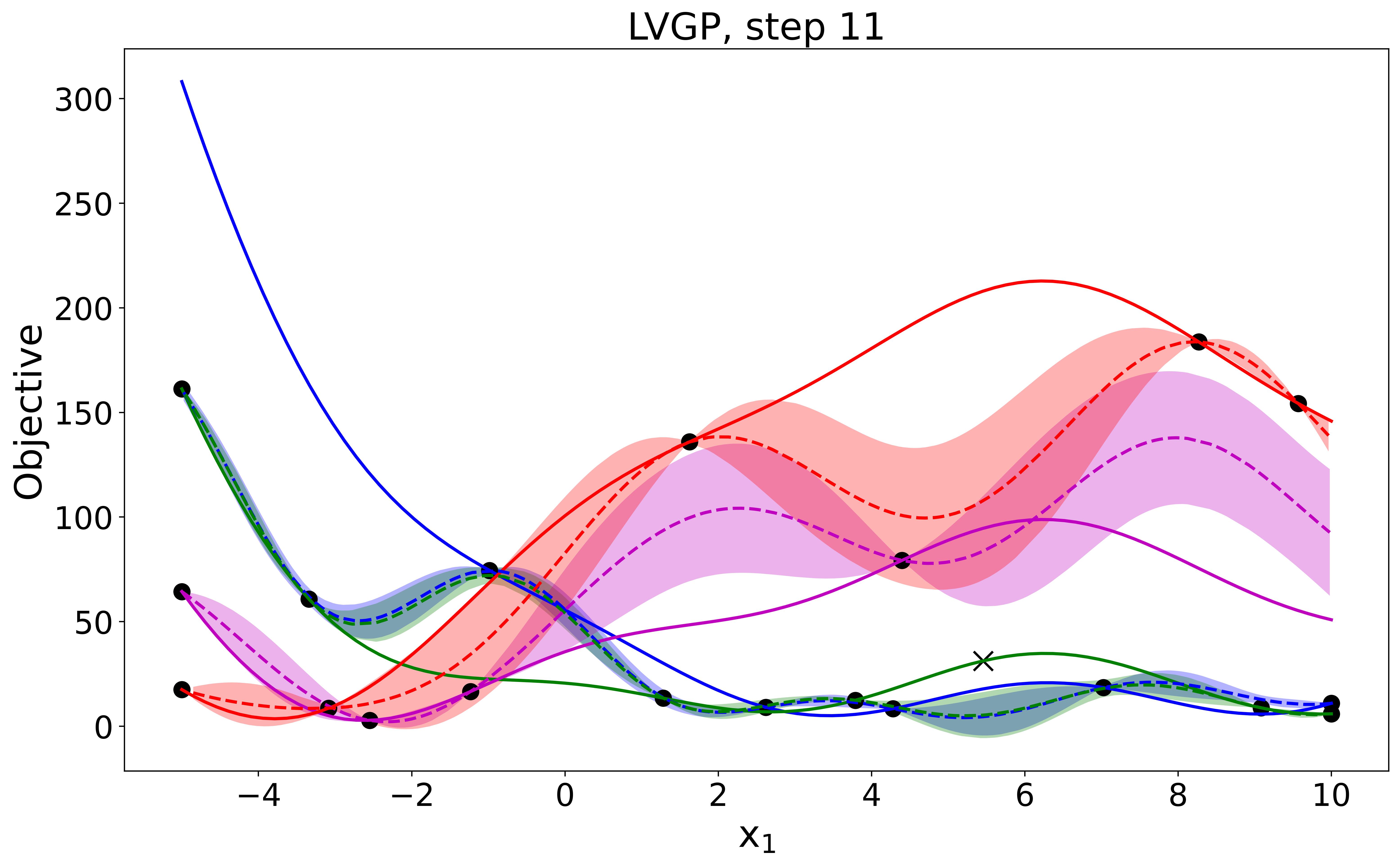

Supplement: Supplementary file 1 — Supplementary Information 1. [file 41598_2022_23431_MOESM1_ESM.zip › Sampling_Sequence_Figures/Branin_Function/branin_LVGP_11.jpg]

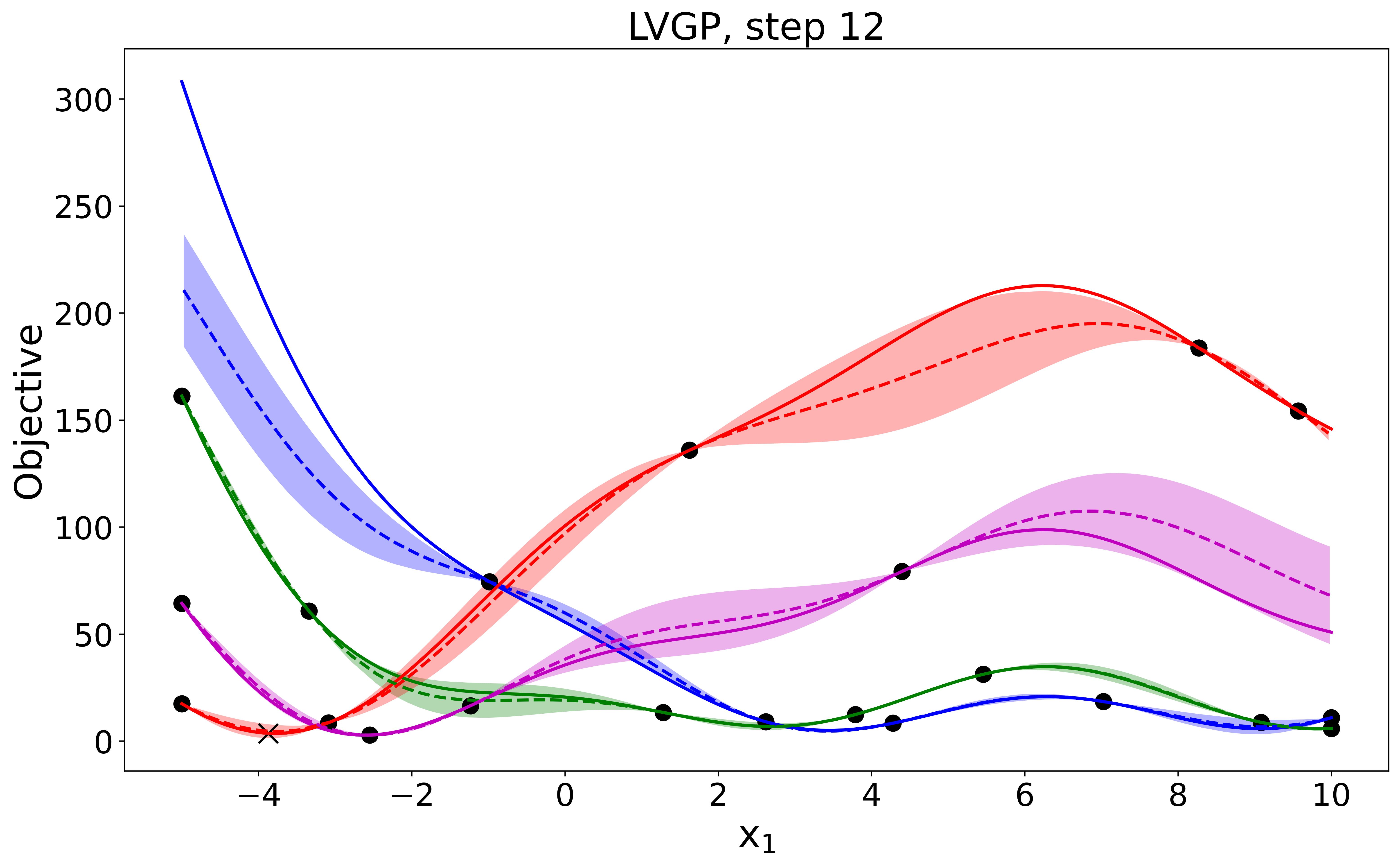

Supplement: Supplementary file 1 — Supplementary Information 1. [file 41598_2022_23431_MOESM1_ESM.zip › Sampling_Sequence_Figures/Branin_Function/branin_LVGP_12.jpg]

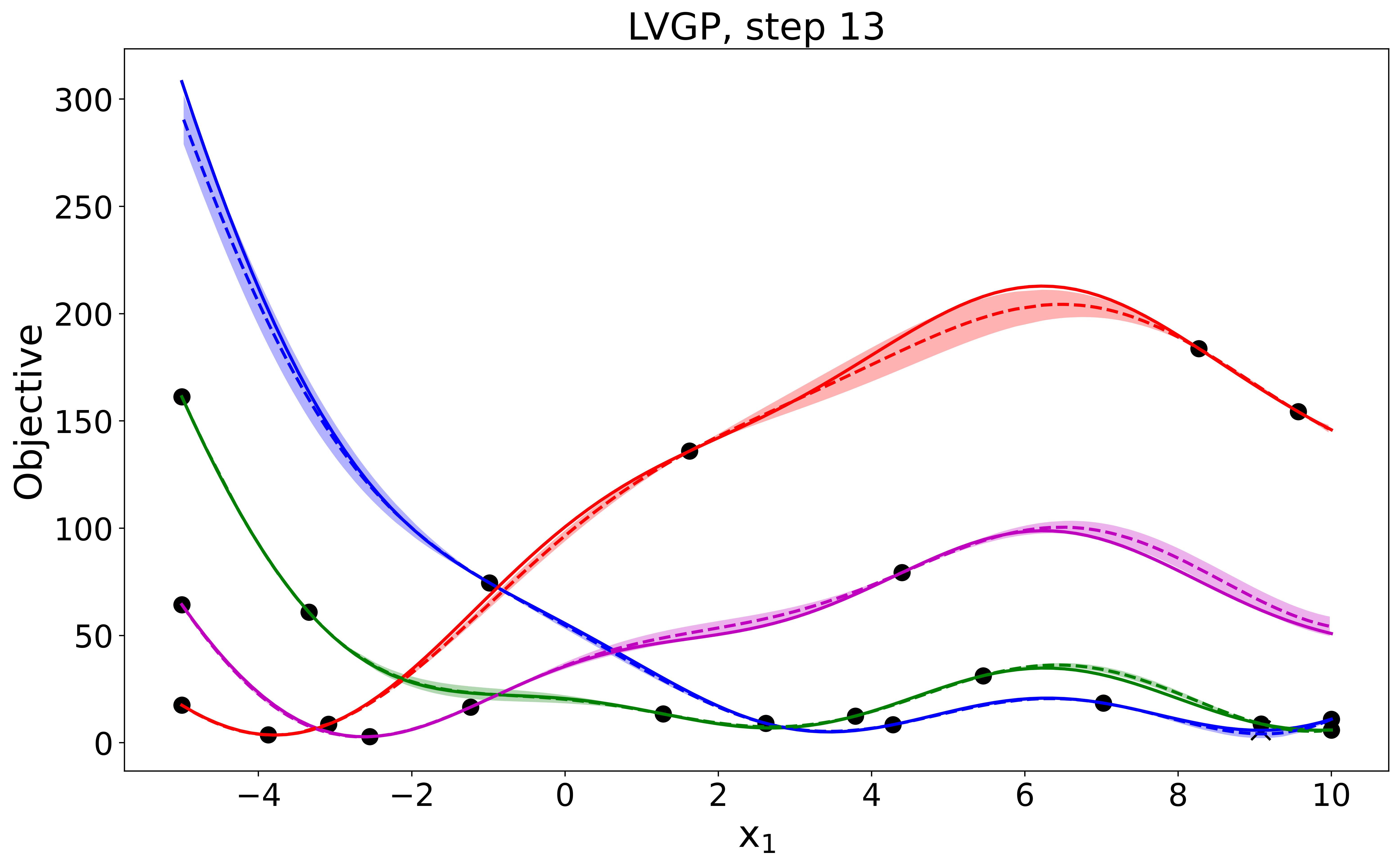

Supplement: Supplementary file 1 — Supplementary Information 1. [file 41598_2022_23431_MOESM1_ESM.zip › Sampling_Sequence_Figures/Branin_Function/branin_LVGP_13.jpg]

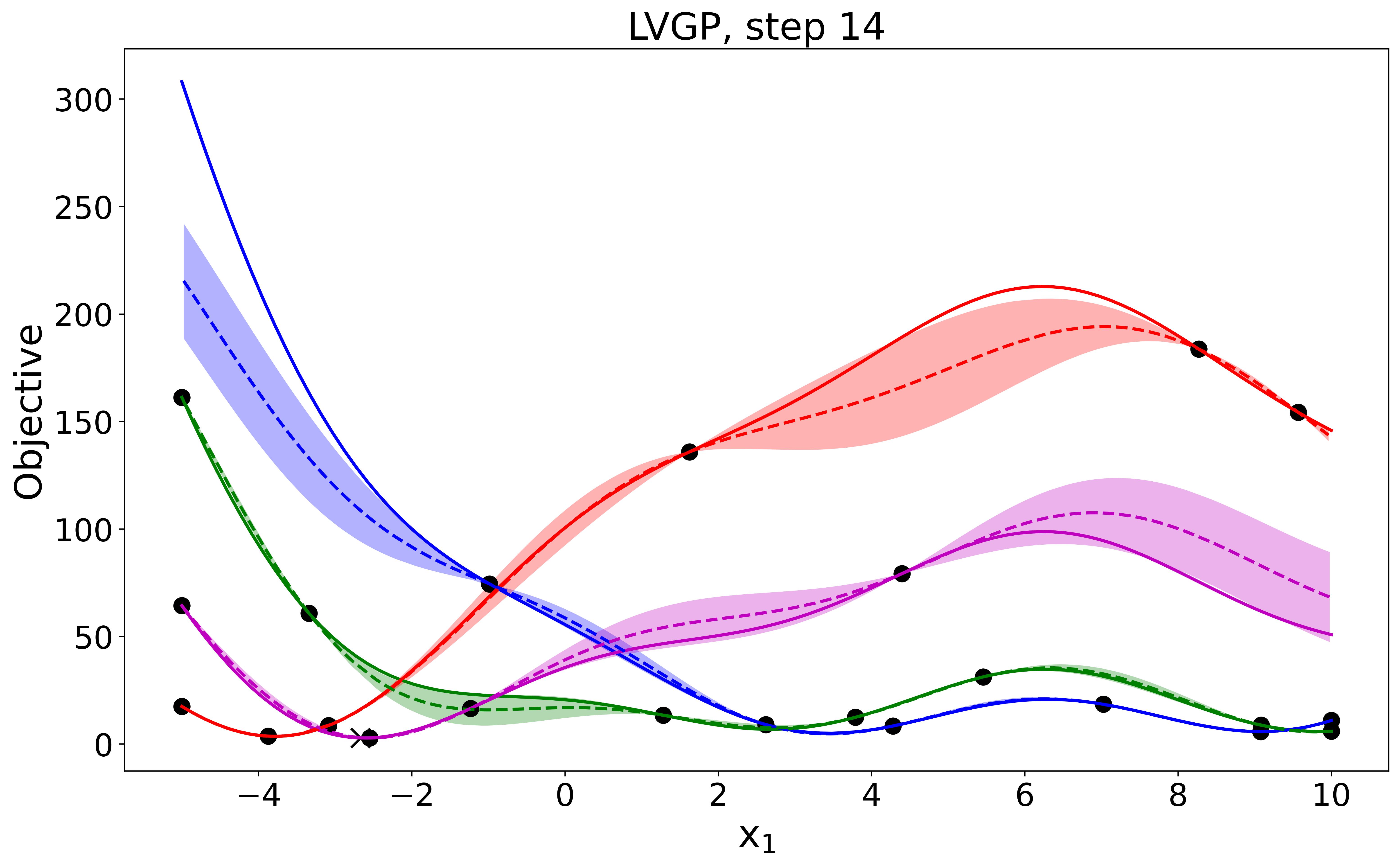

Supplement: Supplementary file 1 — Supplementary Information 1. [file 41598_2022_23431_MOESM1_ESM.zip › Sampling_Sequence_Figures/Branin_Function/branin_LVGP_14.jpg]

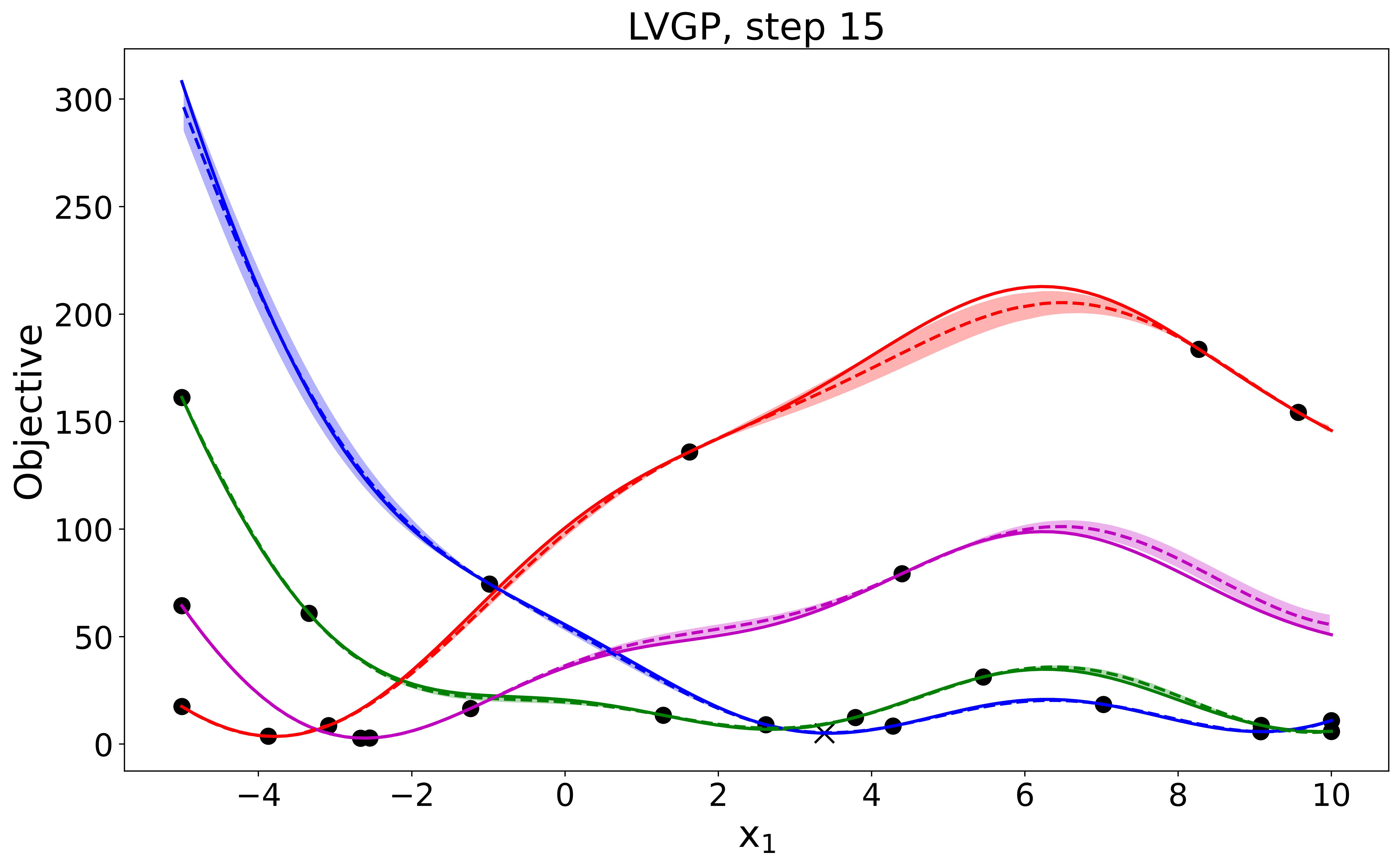

Supplement: Supplementary file 1 — Supplementary Information 1. [file 41598_2022_23431_MOESM1_ESM.zip › Sampling_Sequence_Figures/Branin_Function/branin_LVGP_15.jpg]

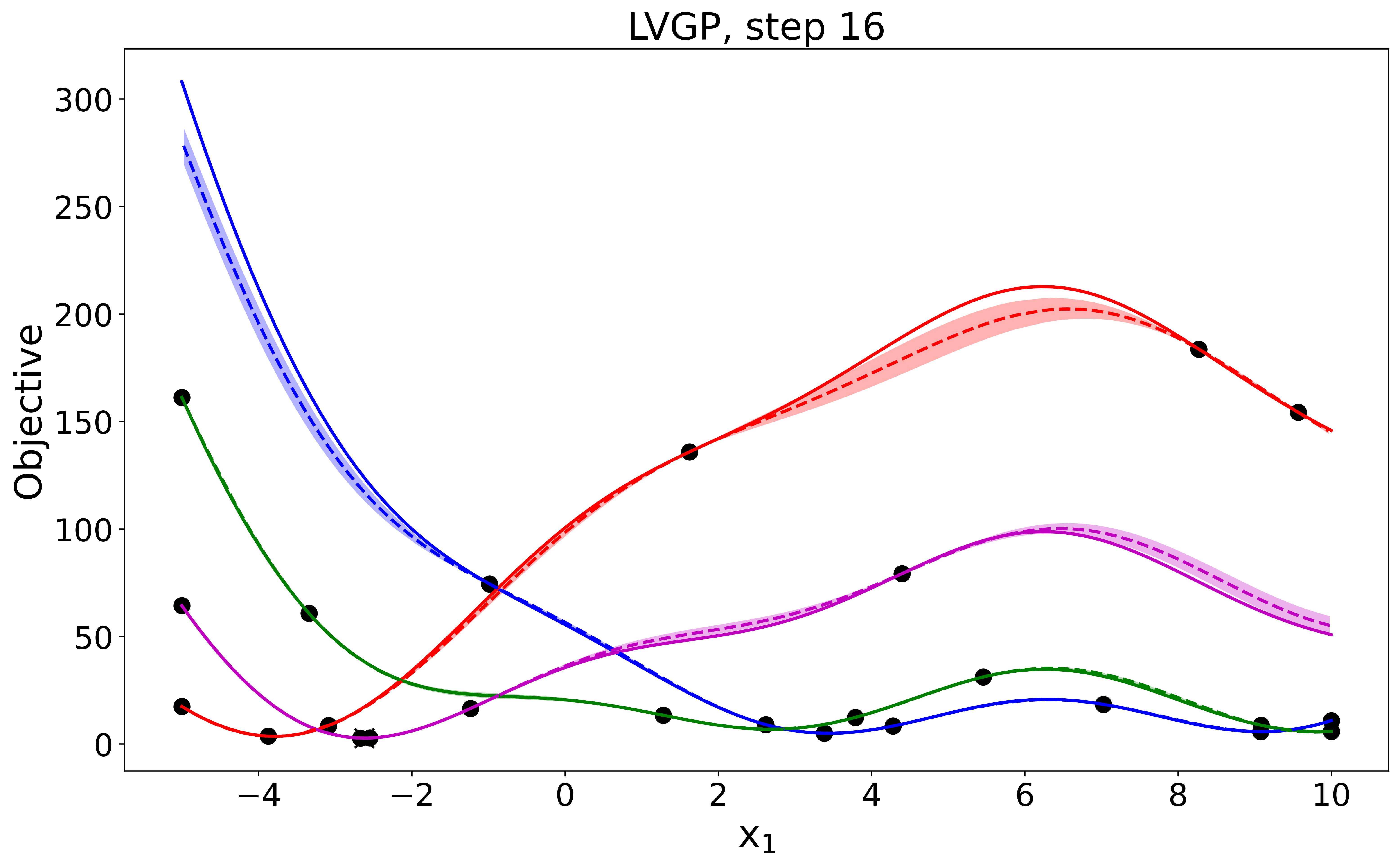

Supplement: Supplementary file 1 — Supplementary Information 1. [file 41598_2022_23431_MOESM1_ESM.zip › Sampling_Sequence_Figures/Branin_Function/branin_LVGP_16.jpg]

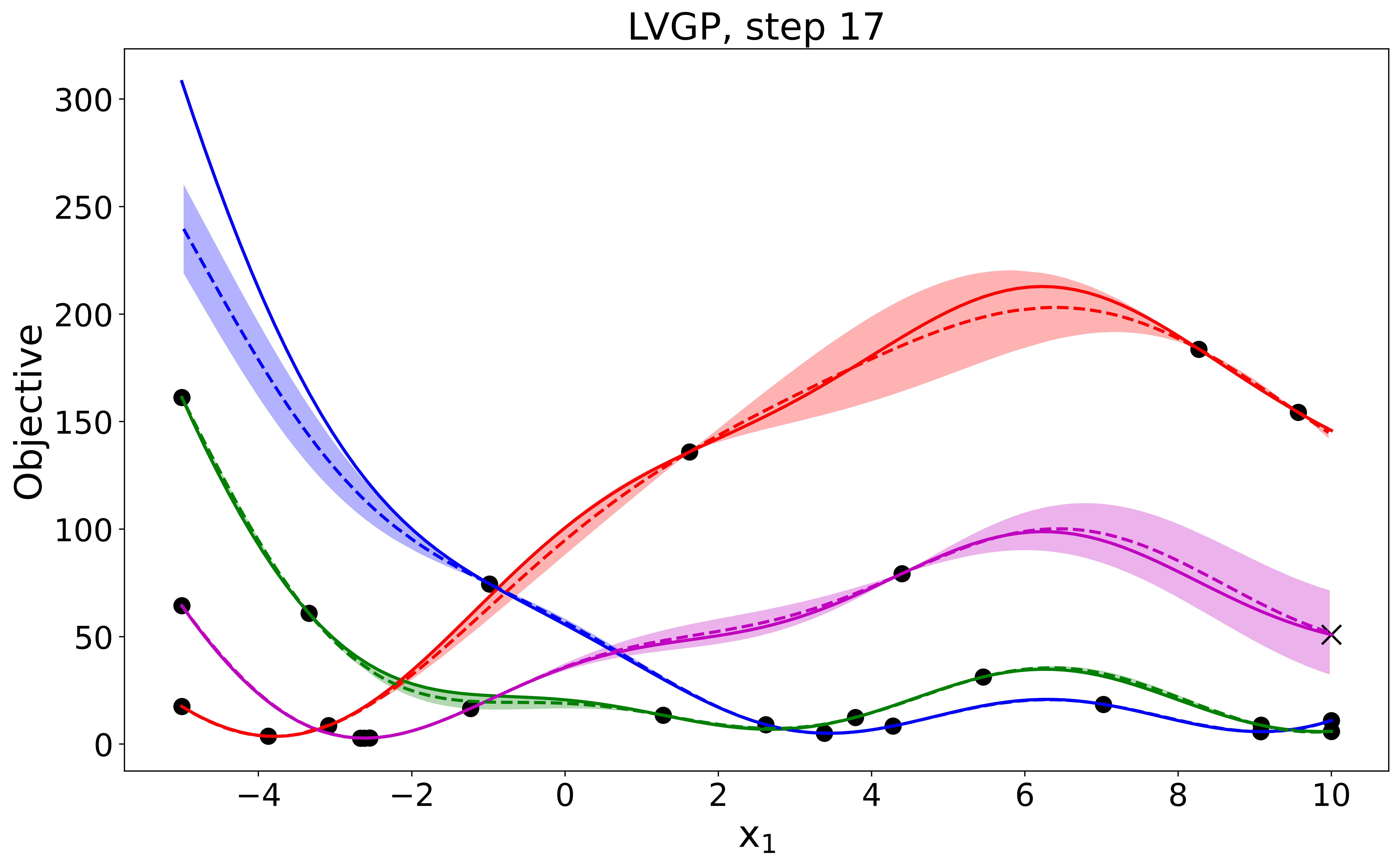

Supplement: Supplementary file 1 — Supplementary Information 1. [file 41598_2022_23431_MOESM1_ESM.zip › Sampling_Sequence_Figures/Branin_Function/branin_LVGP_17.jpg]

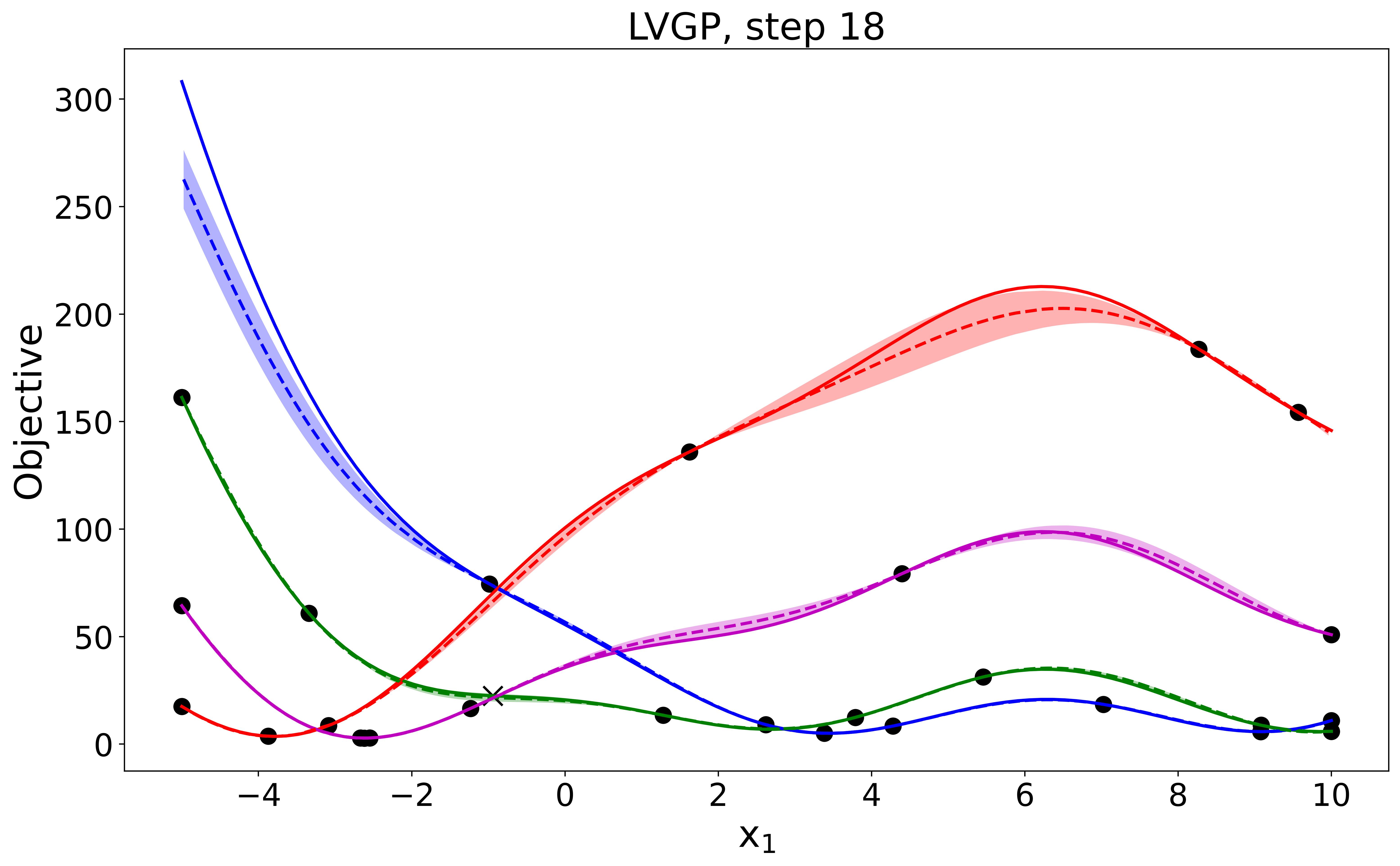

Supplement: Supplementary file 1 — Supplementary Information 1. [file 41598_2022_23431_MOESM1_ESM.zip › Sampling_Sequence_Figures/Branin_Function/branin_LVGP_18.jpg]

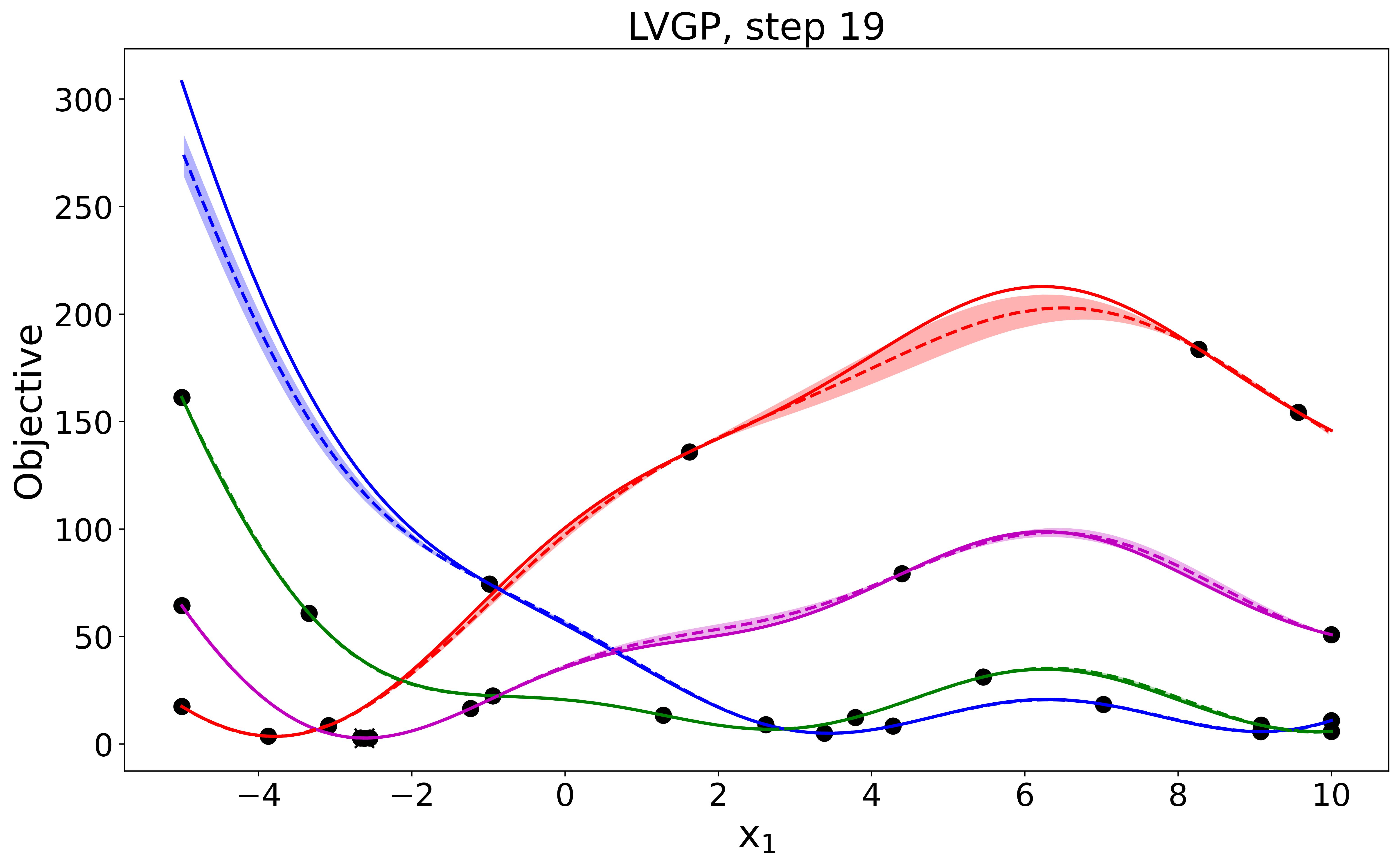

Supplement: Supplementary file 1 — Supplementary Information 1. [file 41598_2022_23431_MOESM1_ESM.zip › Sampling_Sequence_Figures/Branin_Function/branin_LVGP_19.jpg]

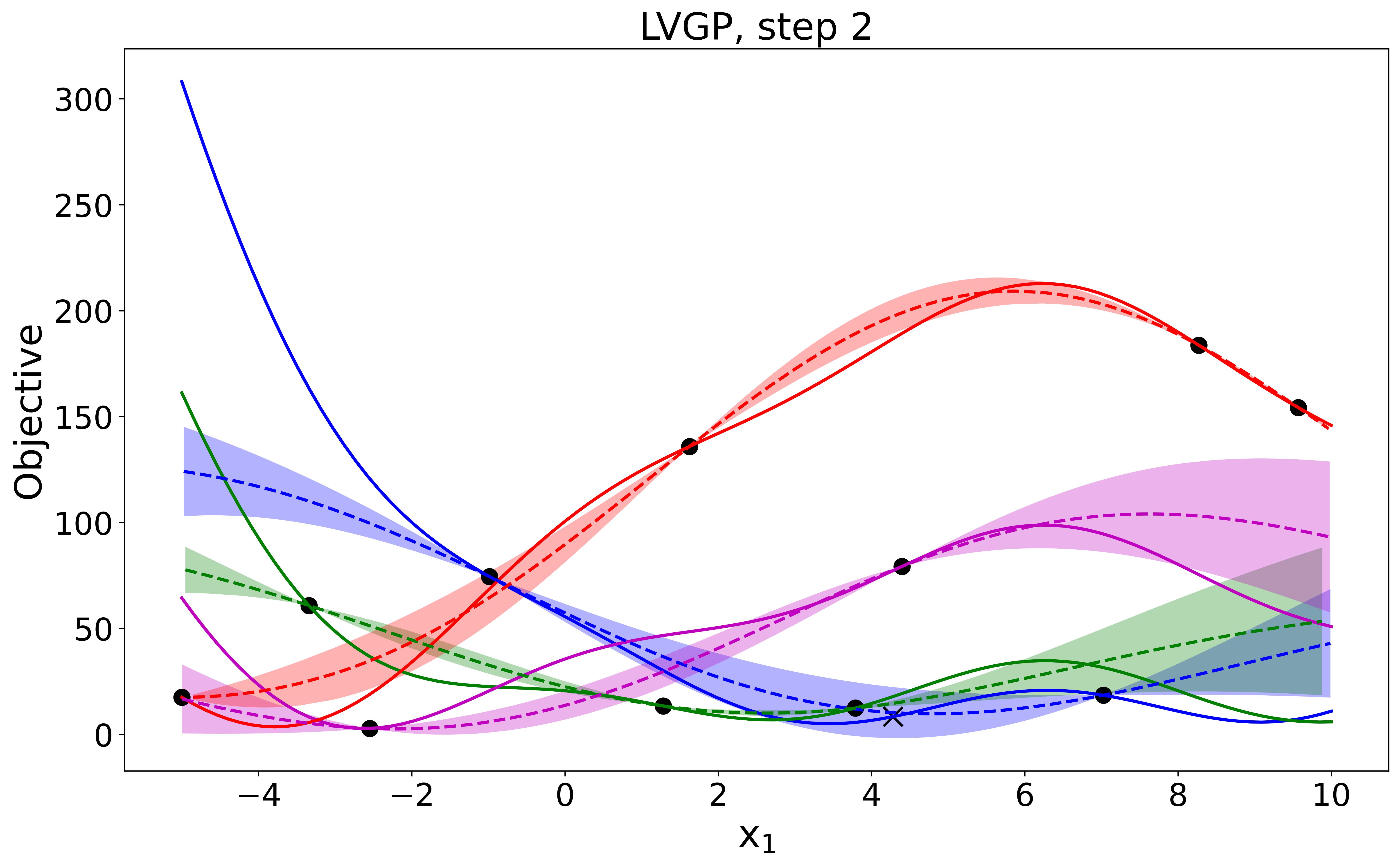

Supplement: Supplementary file 1 — Supplementary Information 1. [file 41598_2022_23431_MOESM1_ESM.zip › Sampling_Sequence_Figures/Branin_Function/branin_LVGP_2.jpg]

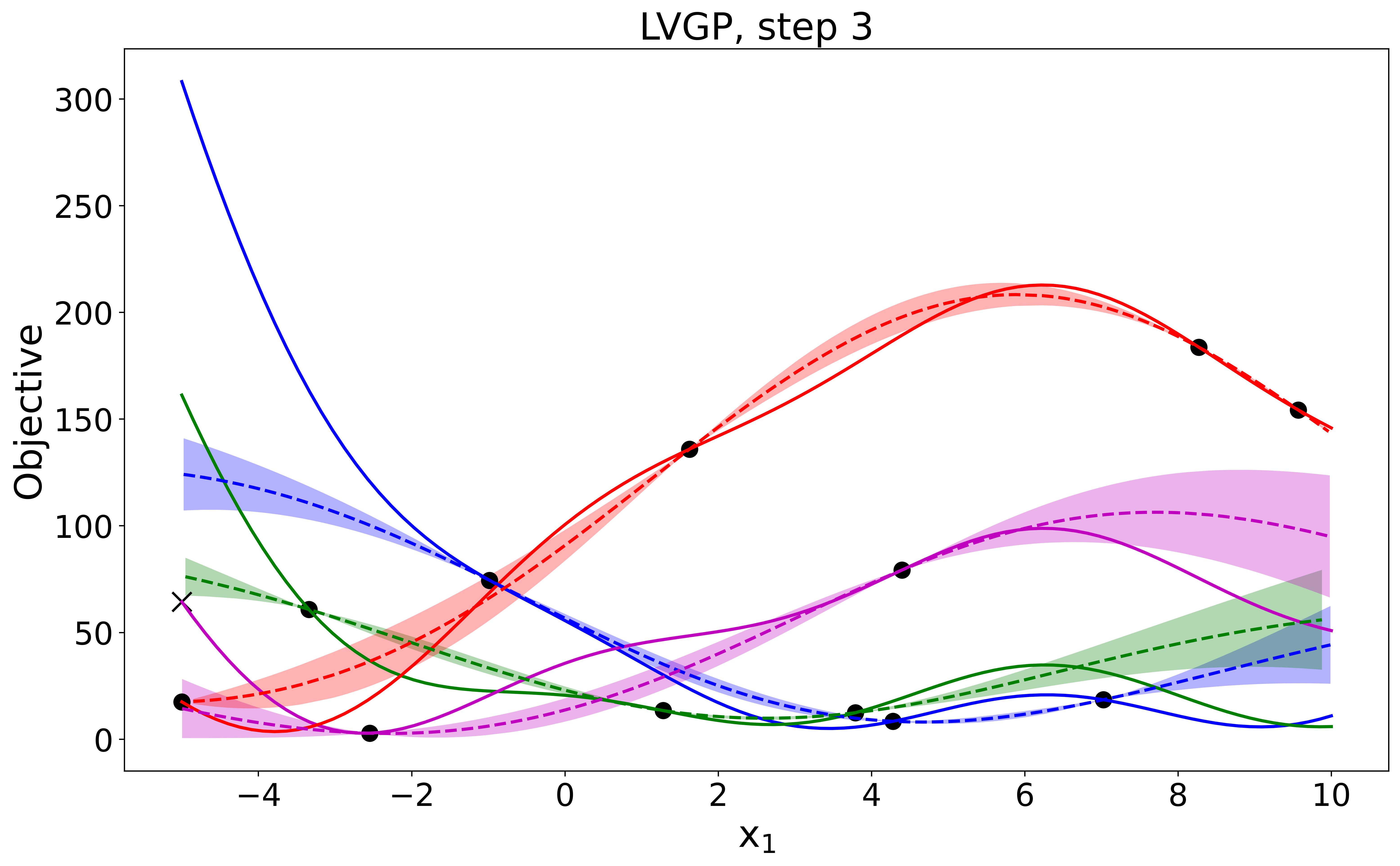

Supplement: Supplementary file 1 — Supplementary Information 1. [file 41598_2022_23431_MOESM1_ESM.zip › Sampling_Sequence_Figures/Branin_Function/branin_LVGP_3.jpg]

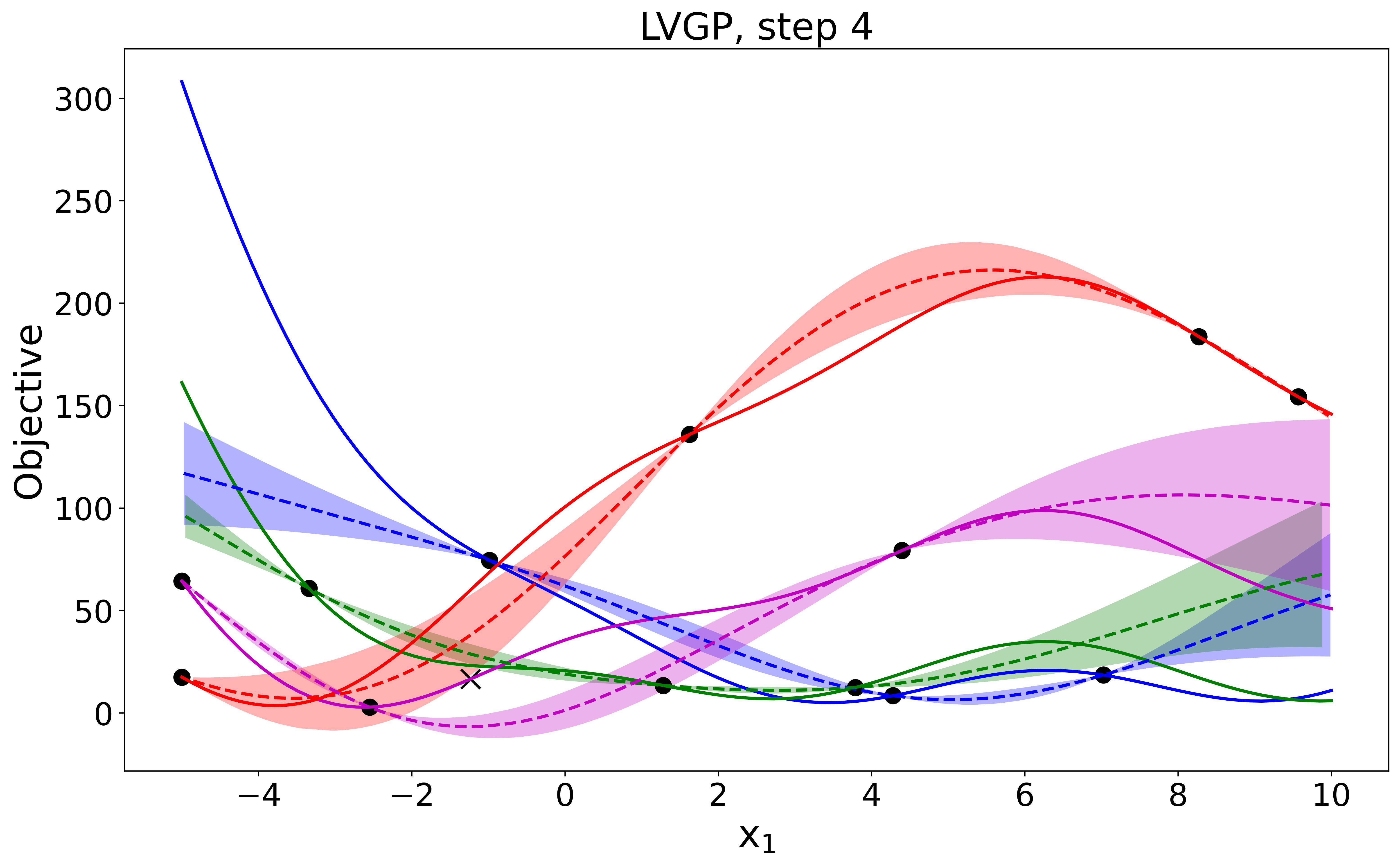

Supplement: Supplementary file 1 — Supplementary Information 1. [file 41598_2022_23431_MOESM1_ESM.zip › Sampling_Sequence_Figures/Branin_Function/branin_LVGP_4.jpg]

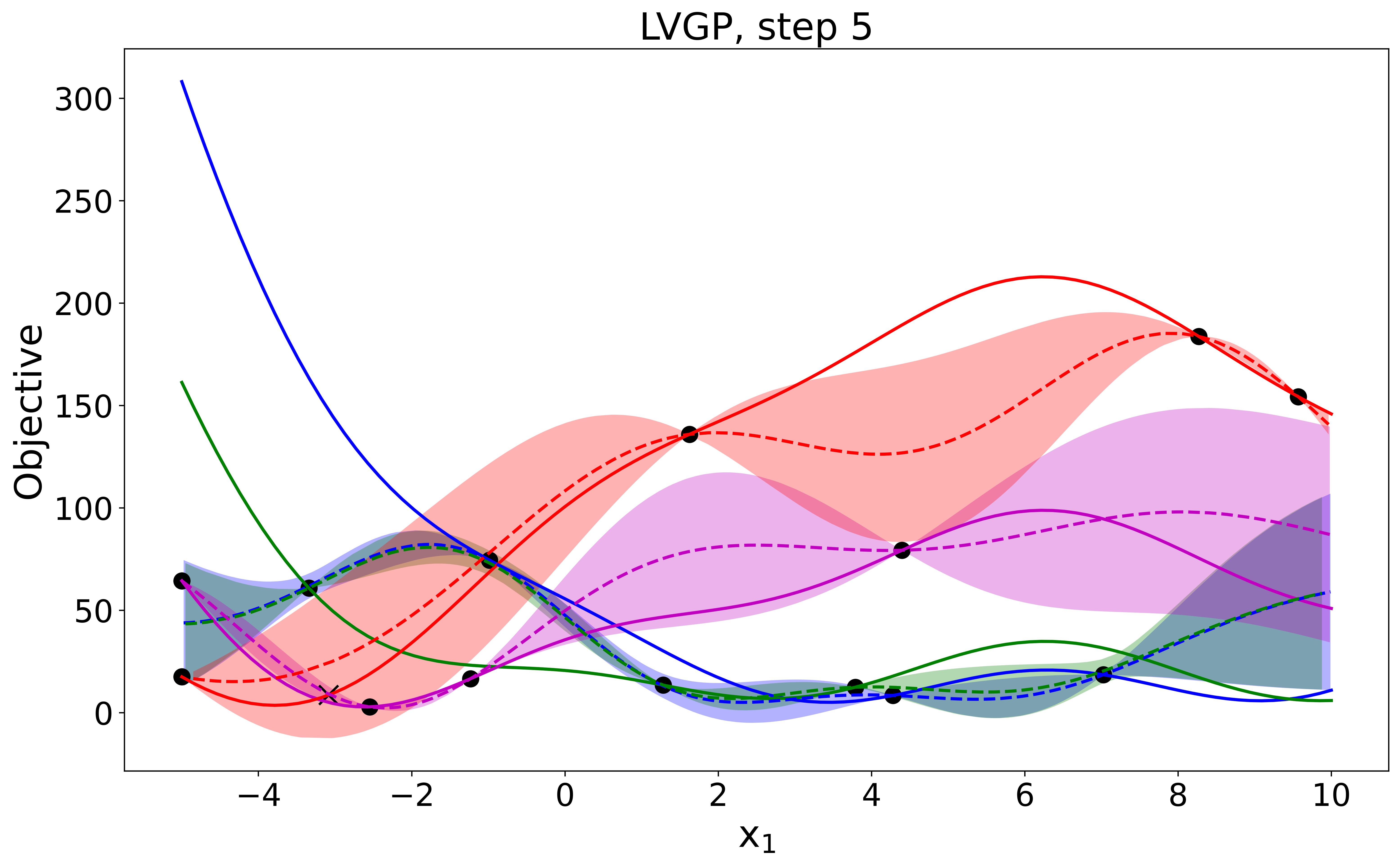

Supplement: Supplementary file 1 — Supplementary Information 1. [file 41598_2022_23431_MOESM1_ESM.zip › Sampling_Sequence_Figures/Branin_Function/branin_LVGP_5.jpg]

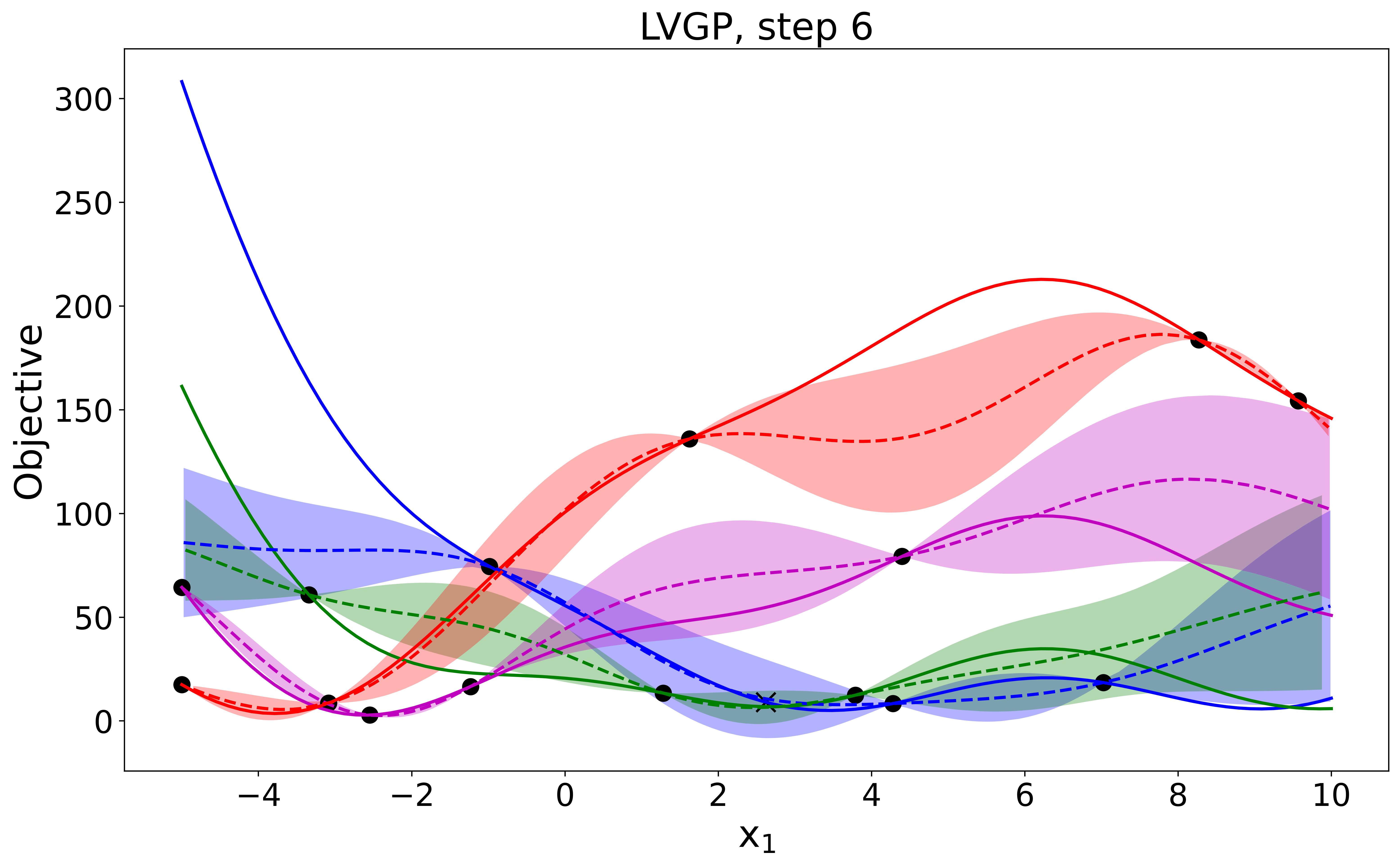

Supplement: Supplementary file 1 — Supplementary Information 1. [file 41598_2022_23431_MOESM1_ESM.zip › Sampling_Sequence_Figures/Branin_Function/branin_LVGP_6.jpg]

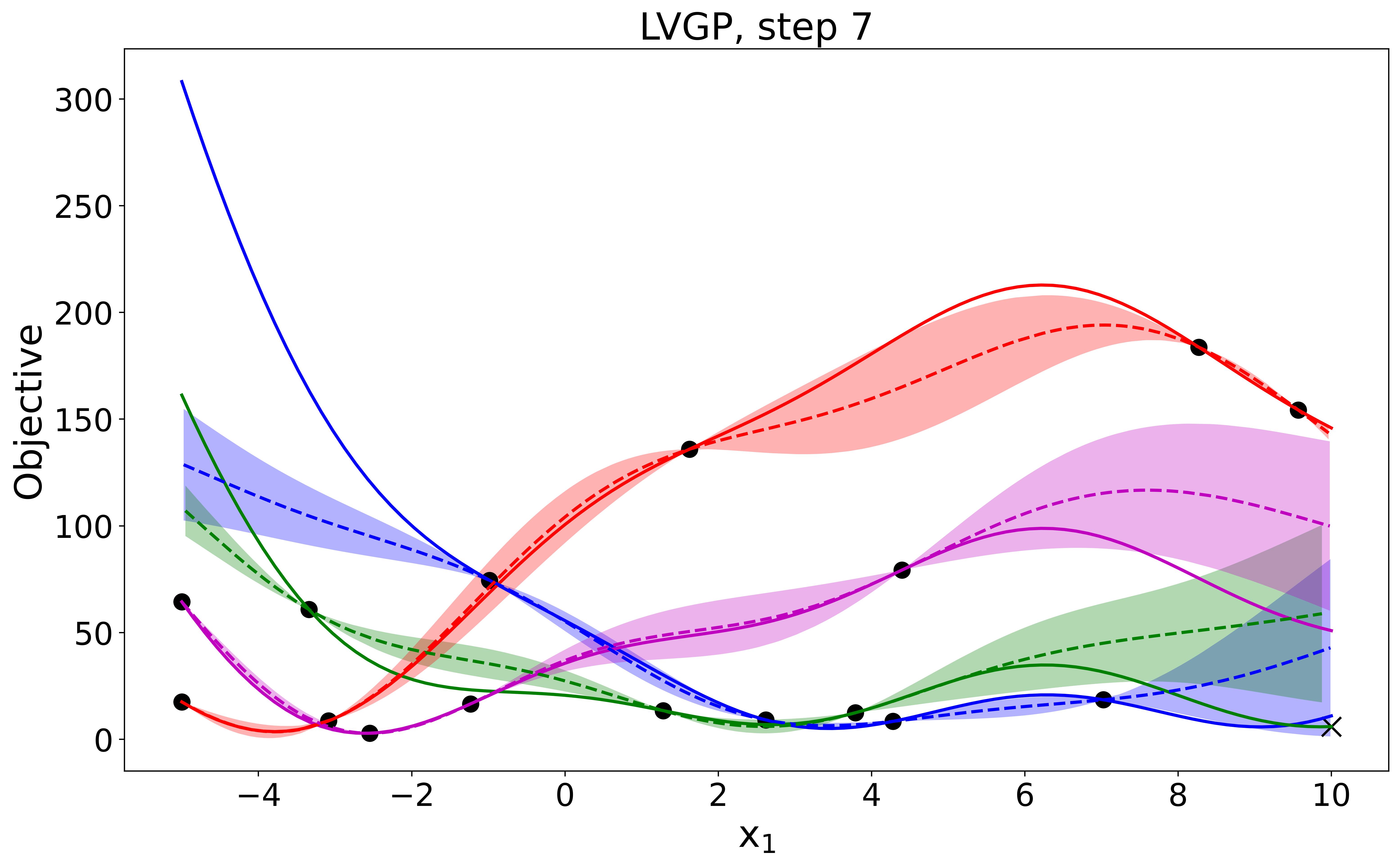

Supplement: Supplementary file 1 — Supplementary Information 1. [file 41598_2022_23431_MOESM1_ESM.zip › Sampling_Sequence_Figures/Branin_Function/branin_LVGP_7.jpg]

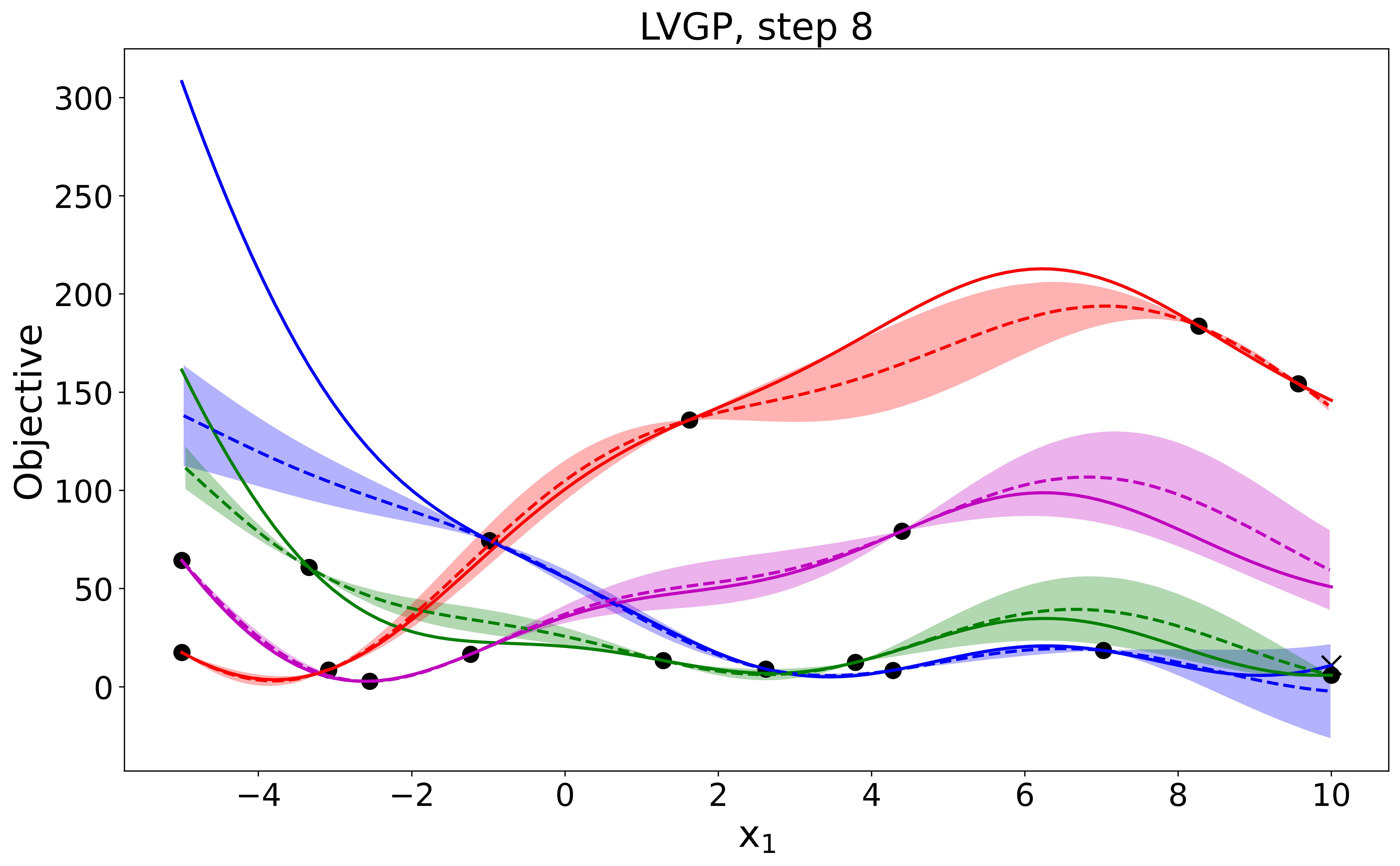

Supplement: Supplementary file 1 — Supplementary Information 1. [file 41598_2022_23431_MOESM1_ESM.zip › Sampling_Sequence_Figures/Branin_Function/branin_LVGP_8.jpg]

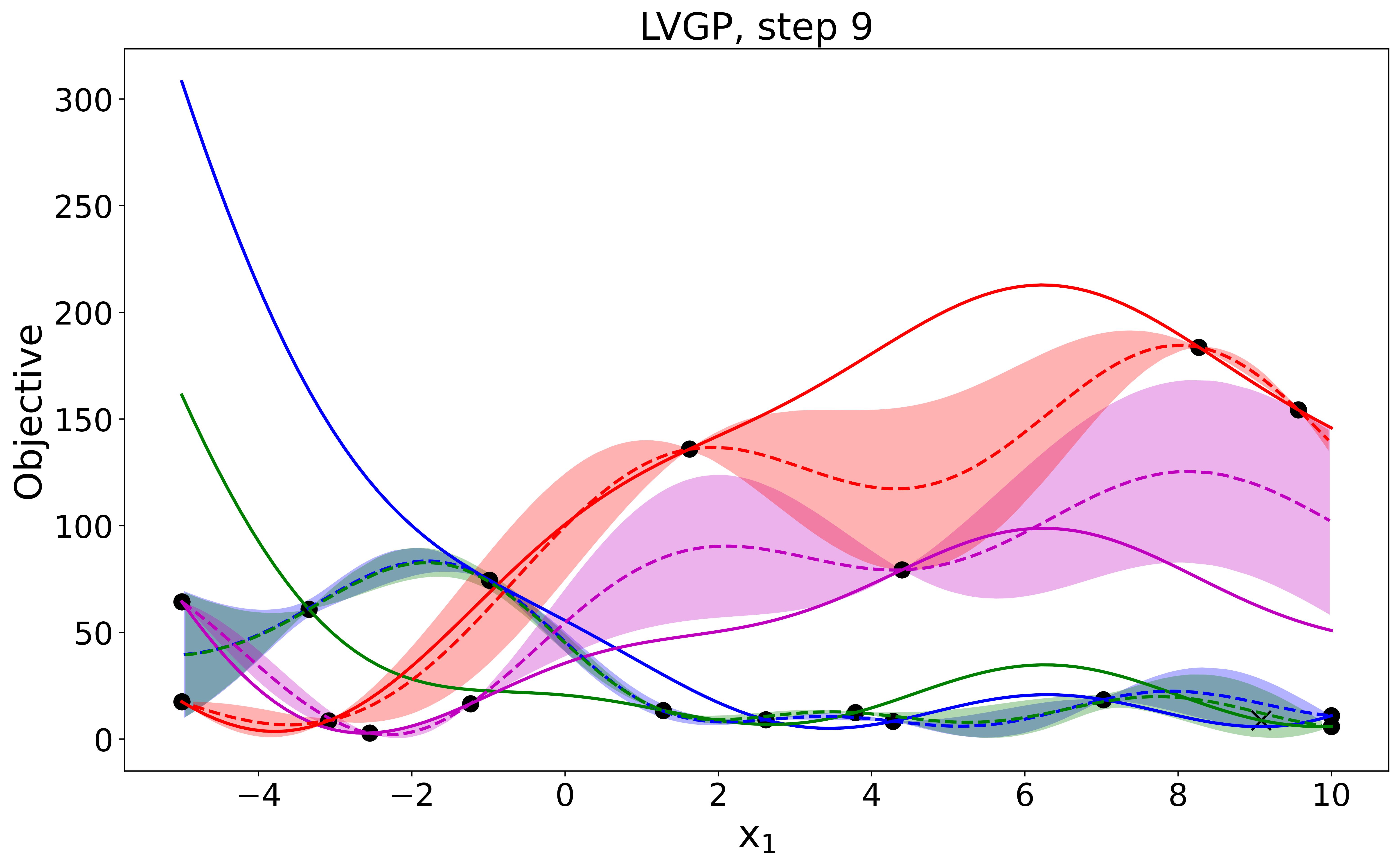

Supplement: Supplementary file 1 — Supplementary Information 1. [file 41598_2022_23431_MOESM1_ESM.zip › Sampling_Sequence_Figures/Branin_Function/branin_LVGP_9.jpg]

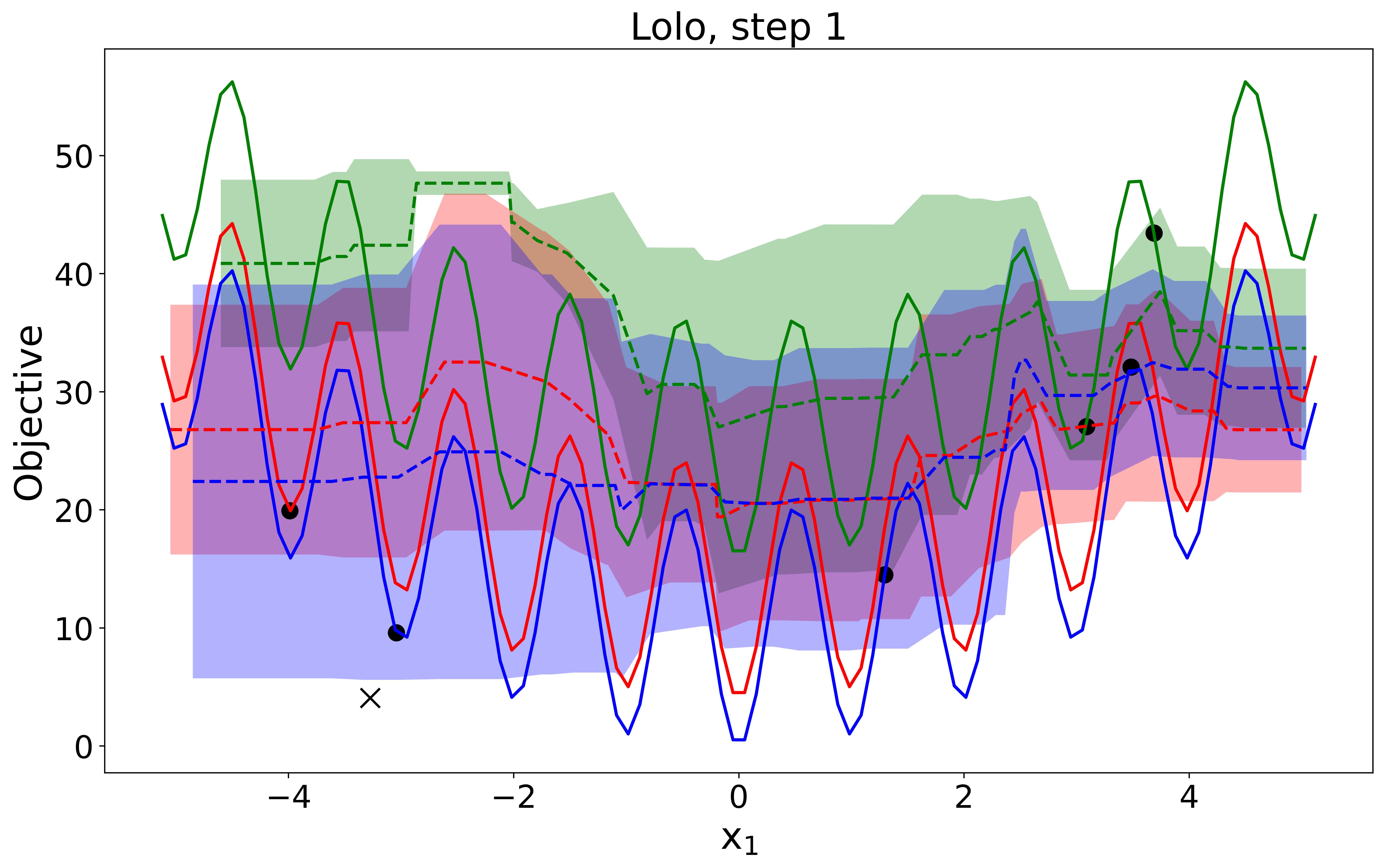

Supplement: Supplementary file 1 — Supplementary Information 1. [file 41598_2022_23431_MOESM1_ESM.zip › Sampling_Sequence_Figures/Rastrigin_Function/rastrigin2_Lolo_1.jpg]

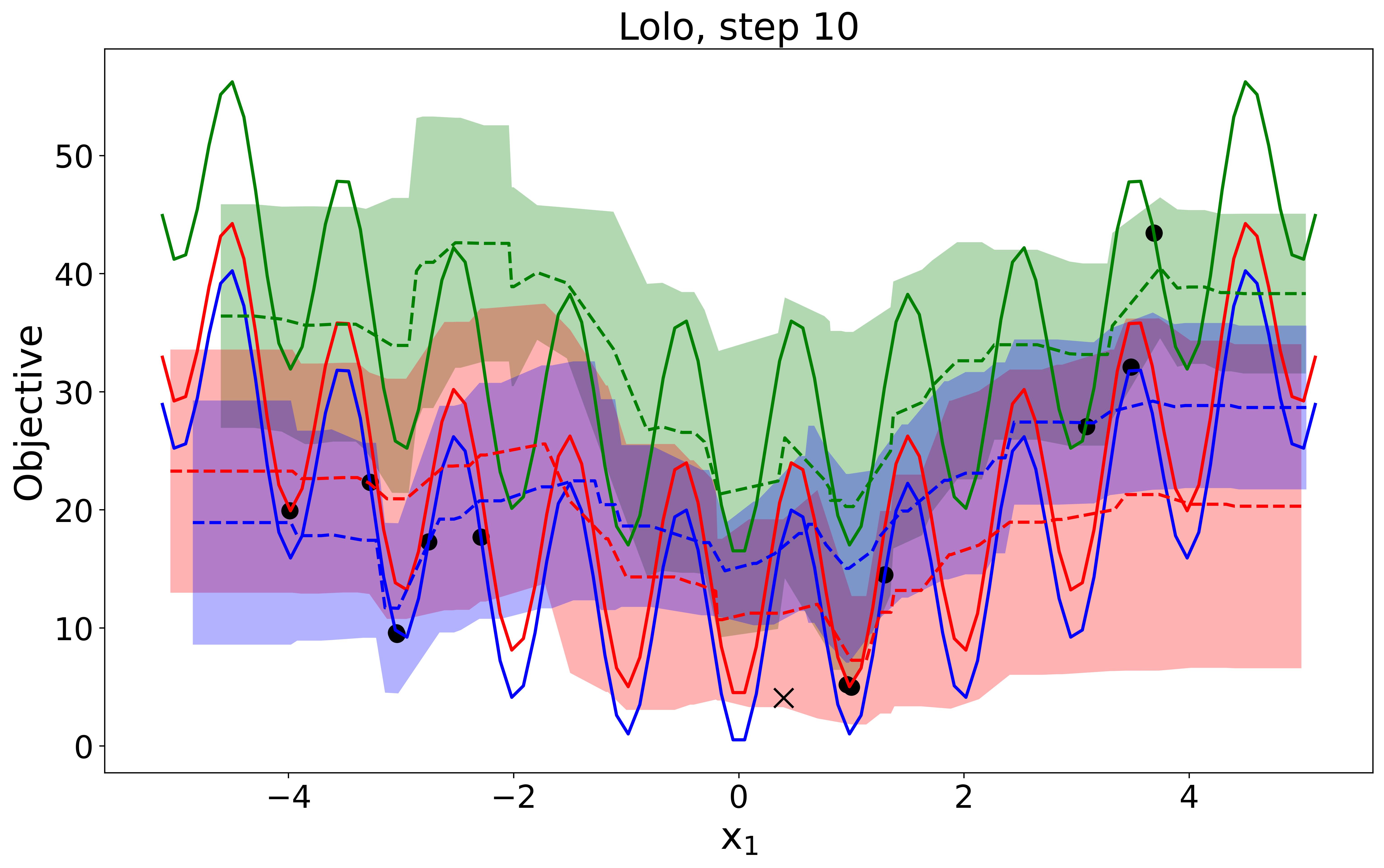

Supplement: Supplementary file 1 — Supplementary Information 1. [file 41598_2022_23431_MOESM1_ESM.zip › Sampling_Sequence_Figures/Rastrigin_Function/rastrigin2_Lolo_10.jpg]

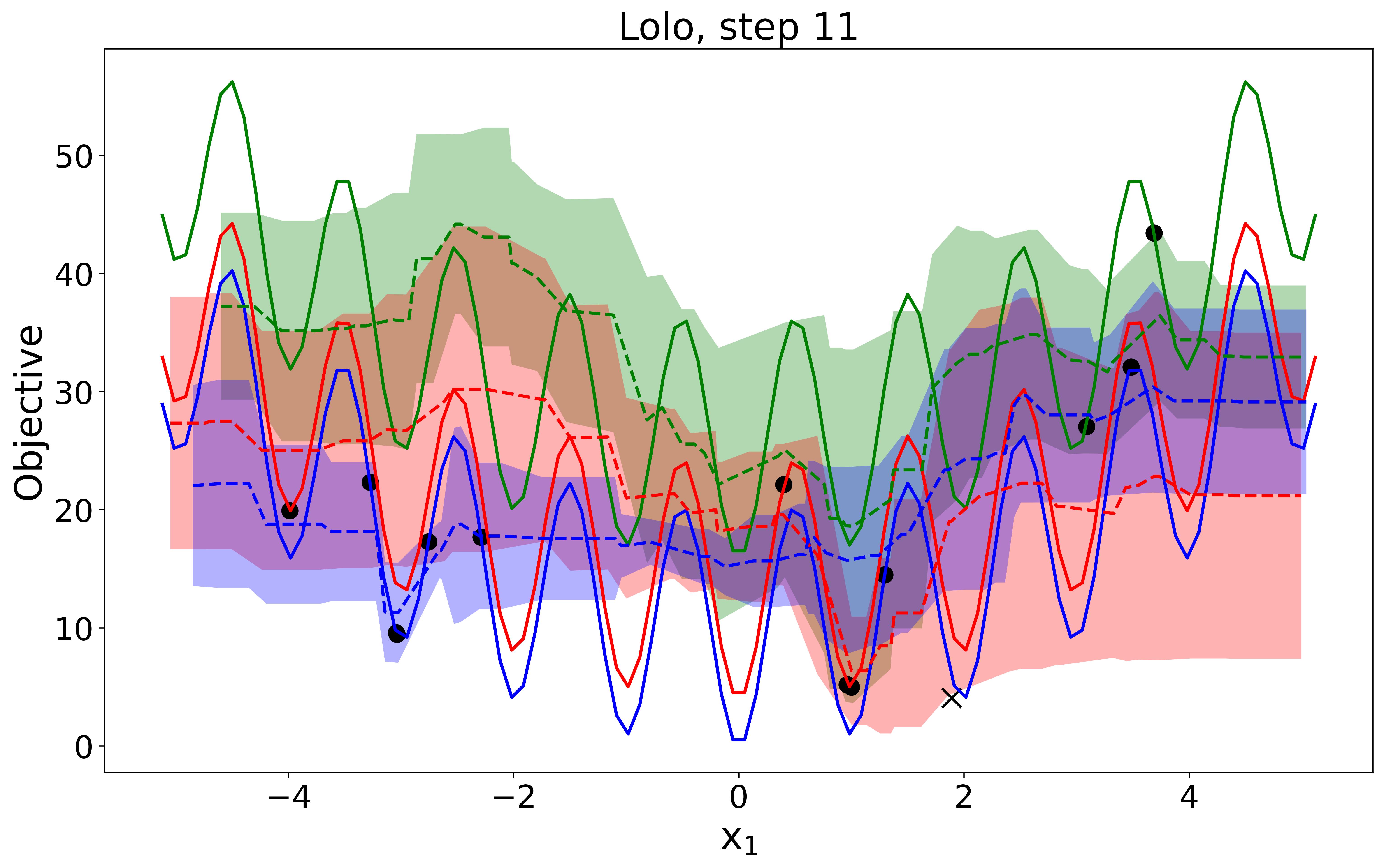

Supplement: Supplementary file 1 — Supplementary Information 1. [file 41598_2022_23431_MOESM1_ESM.zip › Sampling_Sequence_Figures/Rastrigin_Function/rastrigin2_Lolo_11.jpg]

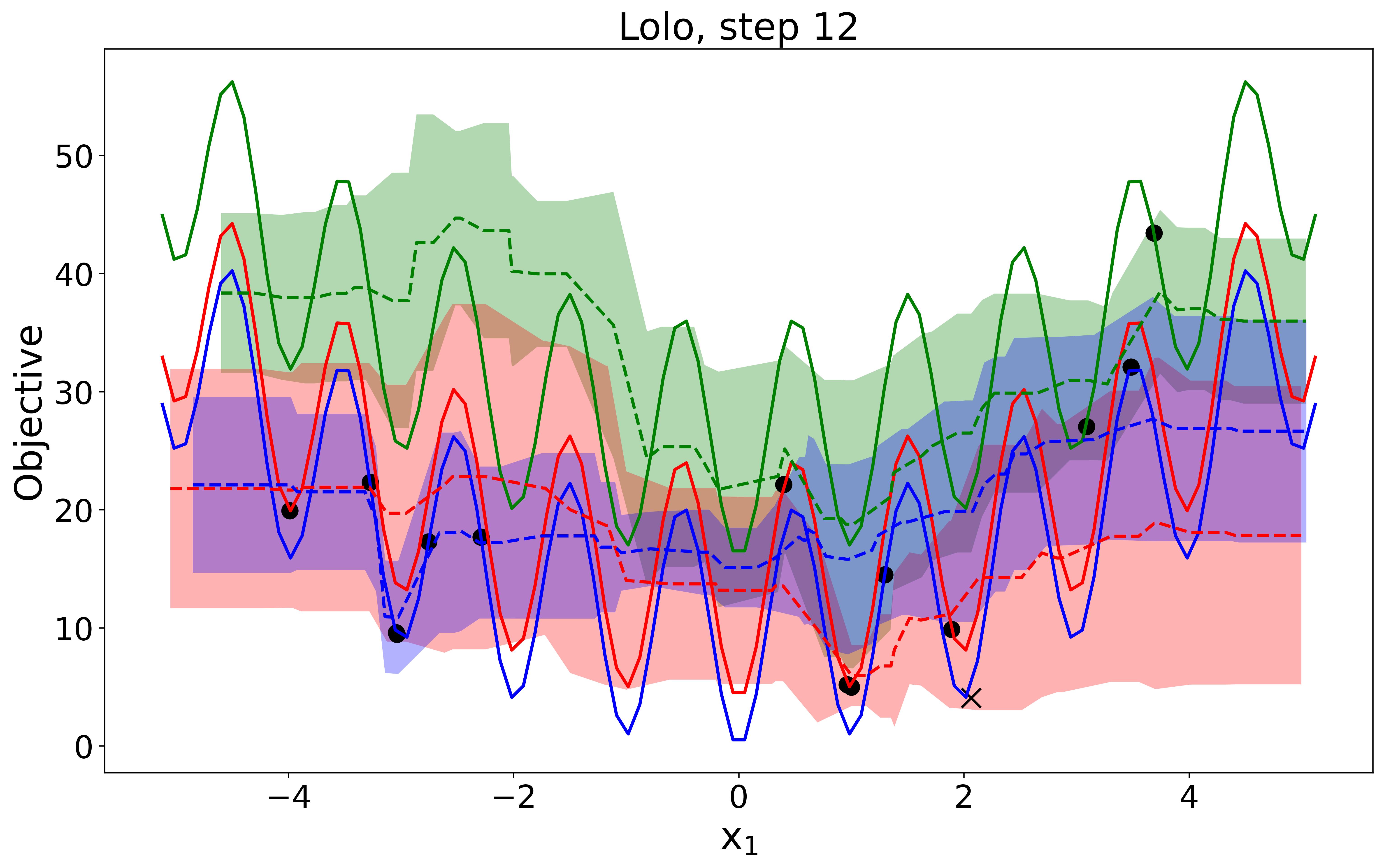

Supplement: Supplementary file 1 — Supplementary Information 1. [file 41598_2022_23431_MOESM1_ESM.zip › Sampling_Sequence_Figures/Rastrigin_Function/rastrigin2_Lolo_12.jpg]

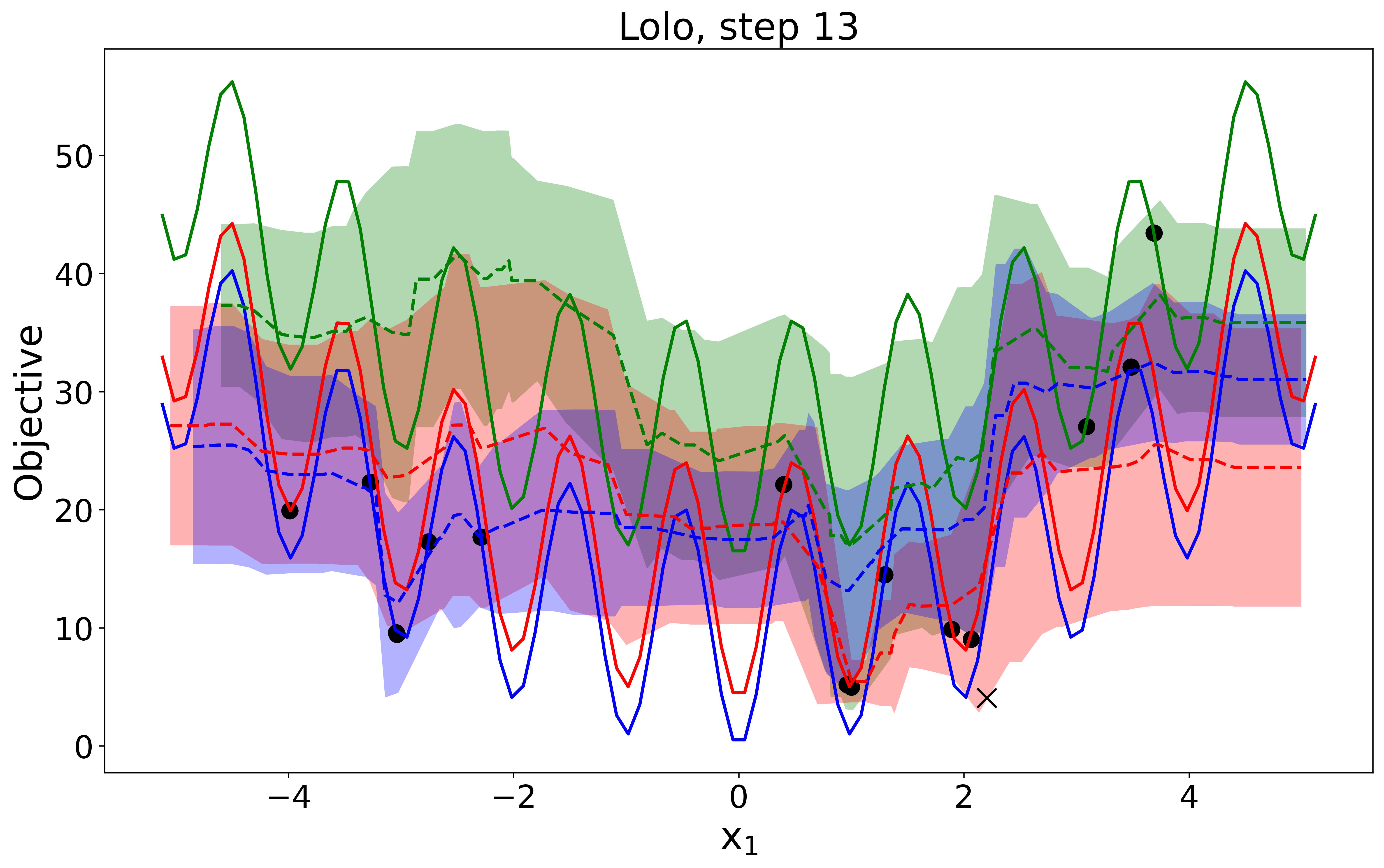

Supplement: Supplementary file 1 — Supplementary Information 1. [file 41598_2022_23431_MOESM1_ESM.zip › Sampling_Sequence_Figures/Rastrigin_Function/rastrigin2_Lolo_13.jpg]

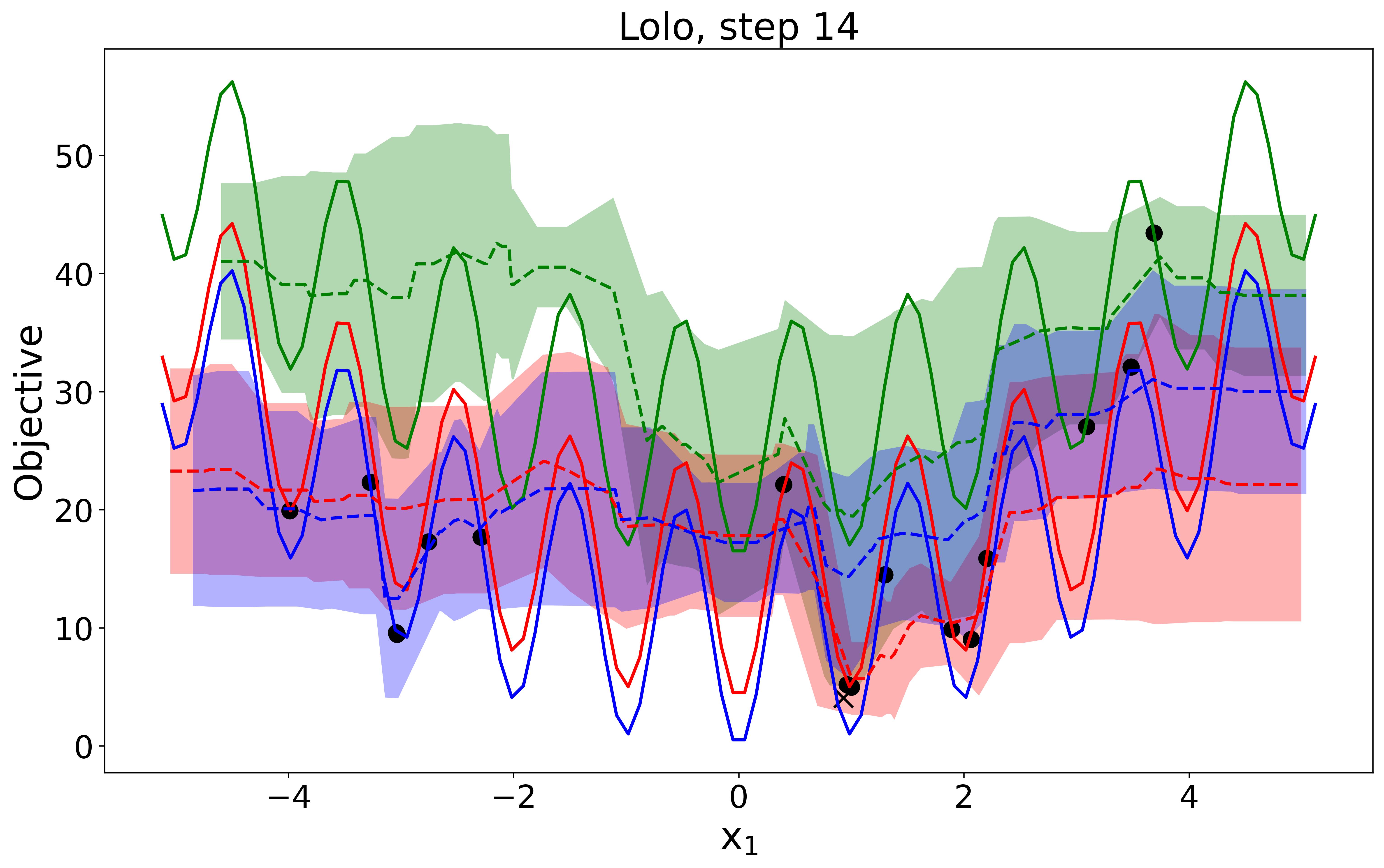

Supplement: Supplementary file 1 — Supplementary Information 1. [file 41598_2022_23431_MOESM1_ESM.zip › Sampling_Sequence_Figures/Rastrigin_Function/rastrigin2_Lolo_14.jpg]

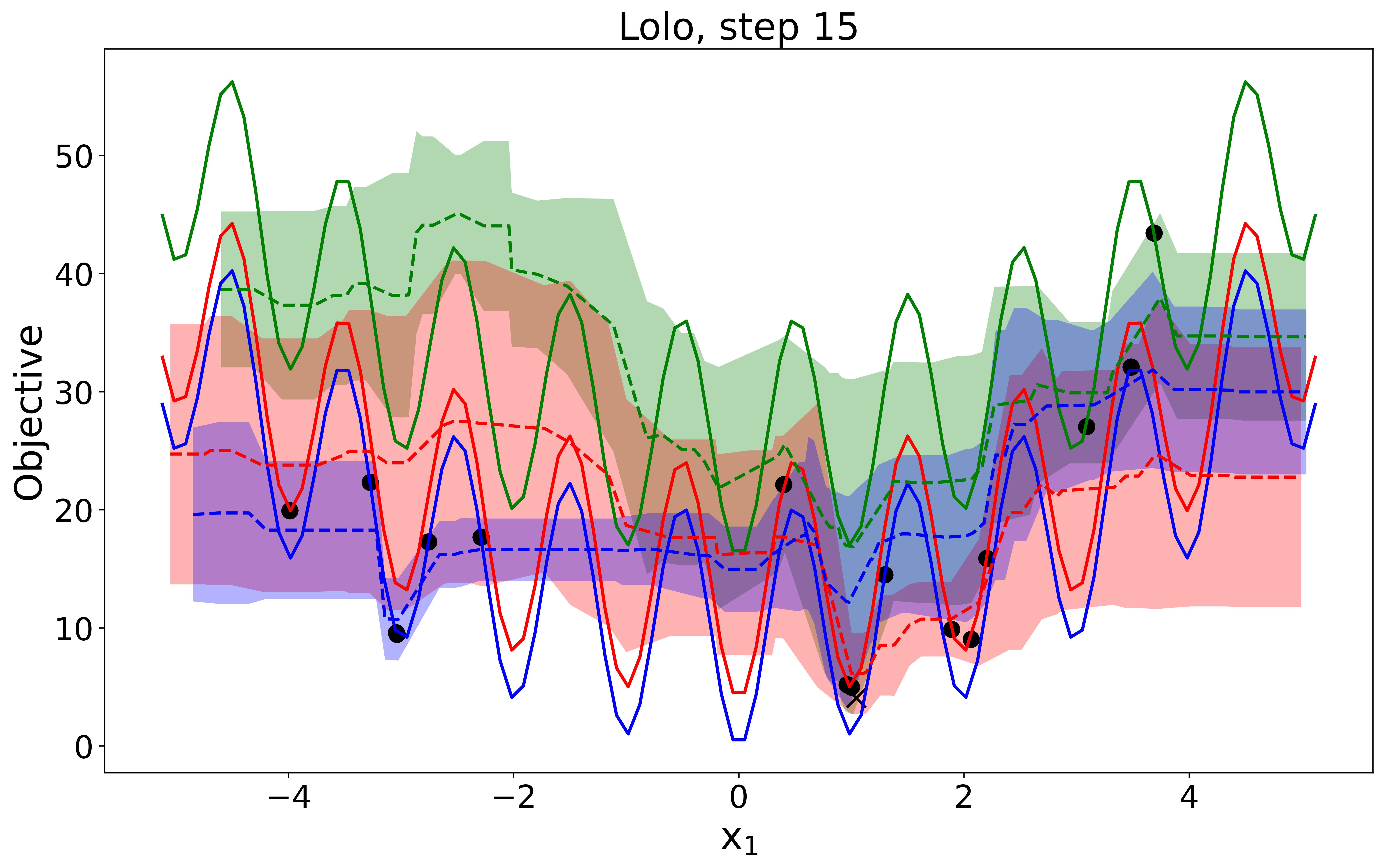

Supplement: Supplementary file 1 — Supplementary Information 1. [file 41598_2022_23431_MOESM1_ESM.zip › Sampling_Sequence_Figures/Rastrigin_Function/rastrigin2_Lolo_15.jpg]

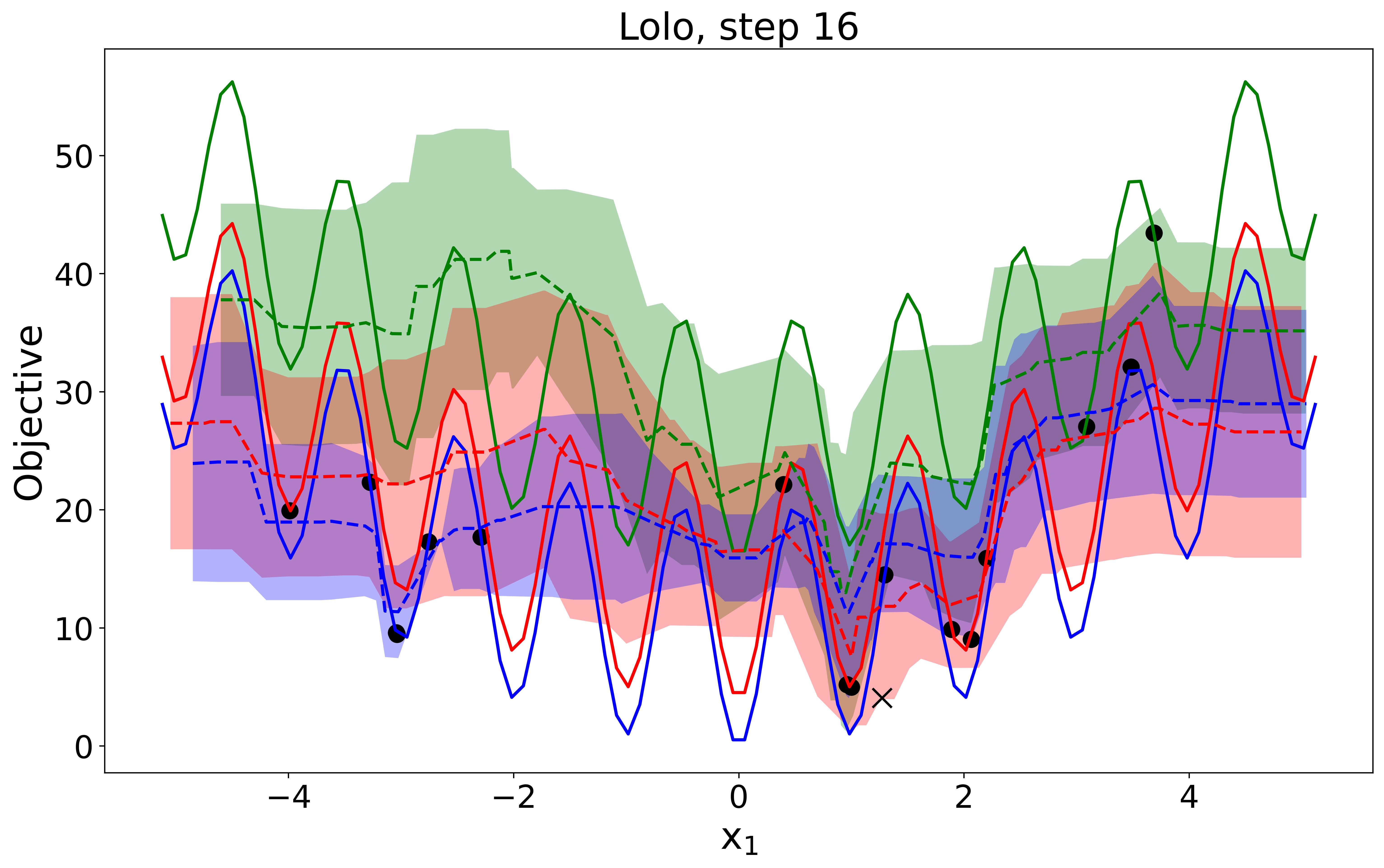

Supplement: Supplementary file 1 — Supplementary Information 1. [file 41598_2022_23431_MOESM1_ESM.zip › Sampling_Sequence_Figures/Rastrigin_Function/rastrigin2_Lolo_16.jpg]

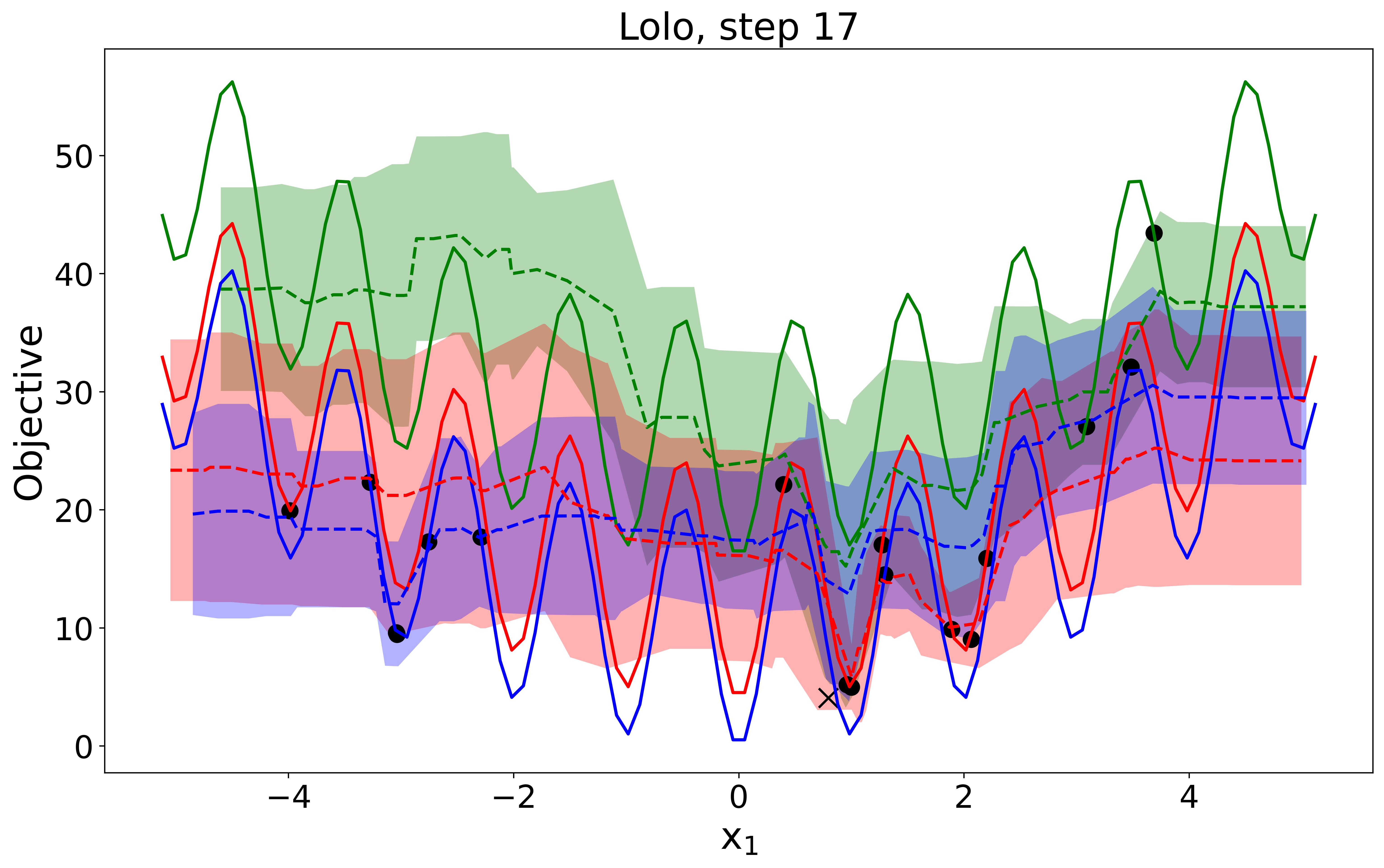

Supplement: Supplementary file 1 — Supplementary Information 1. [file 41598_2022_23431_MOESM1_ESM.zip › Sampling_Sequence_Figures/Rastrigin_Function/rastrigin2_Lolo_17.jpg]

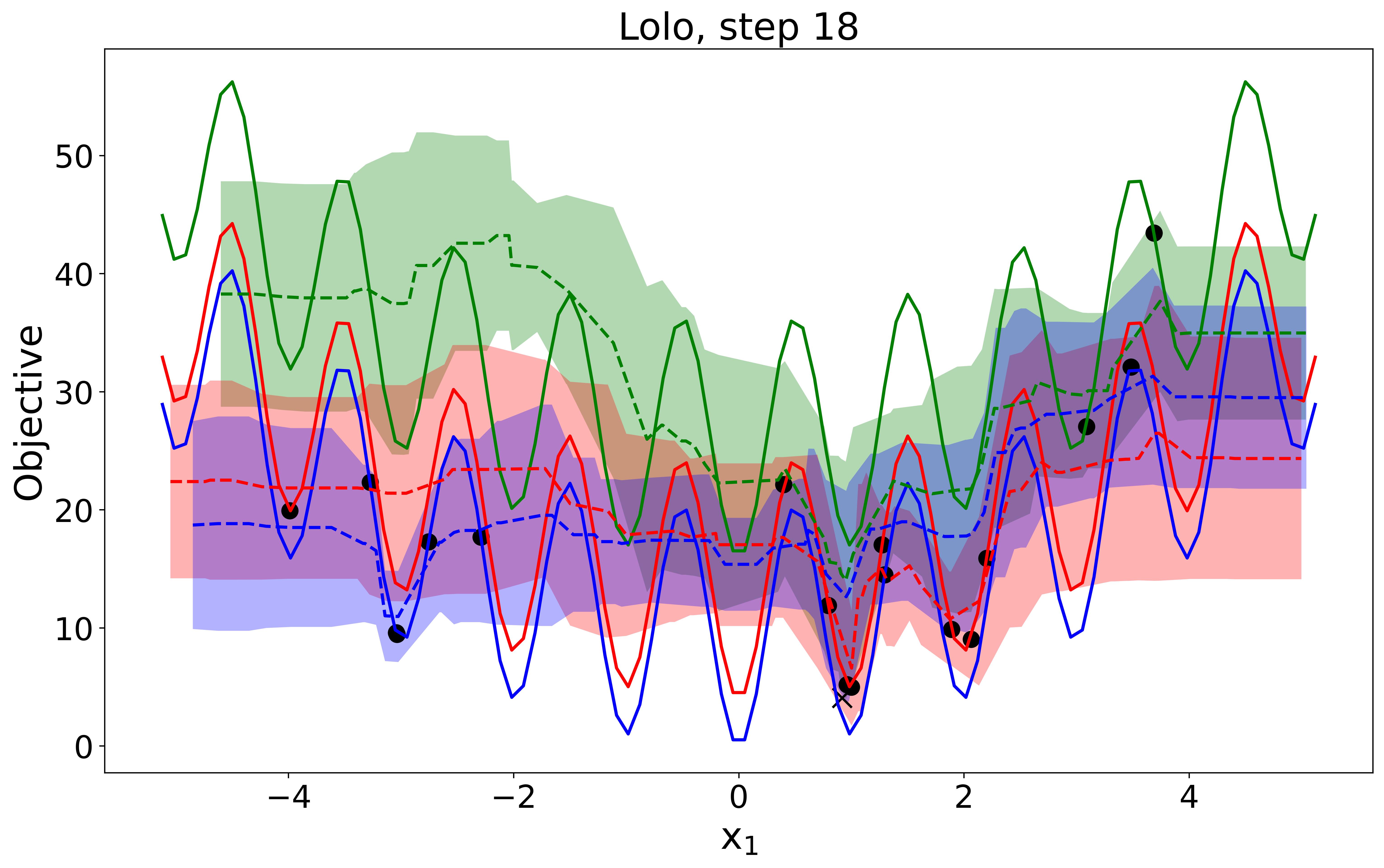

Supplement: Supplementary file 1 — Supplementary Information 1. [file 41598_2022_23431_MOESM1_ESM.zip › Sampling_Sequence_Figures/Rastrigin_Function/rastrigin2_Lolo_18.jpg]

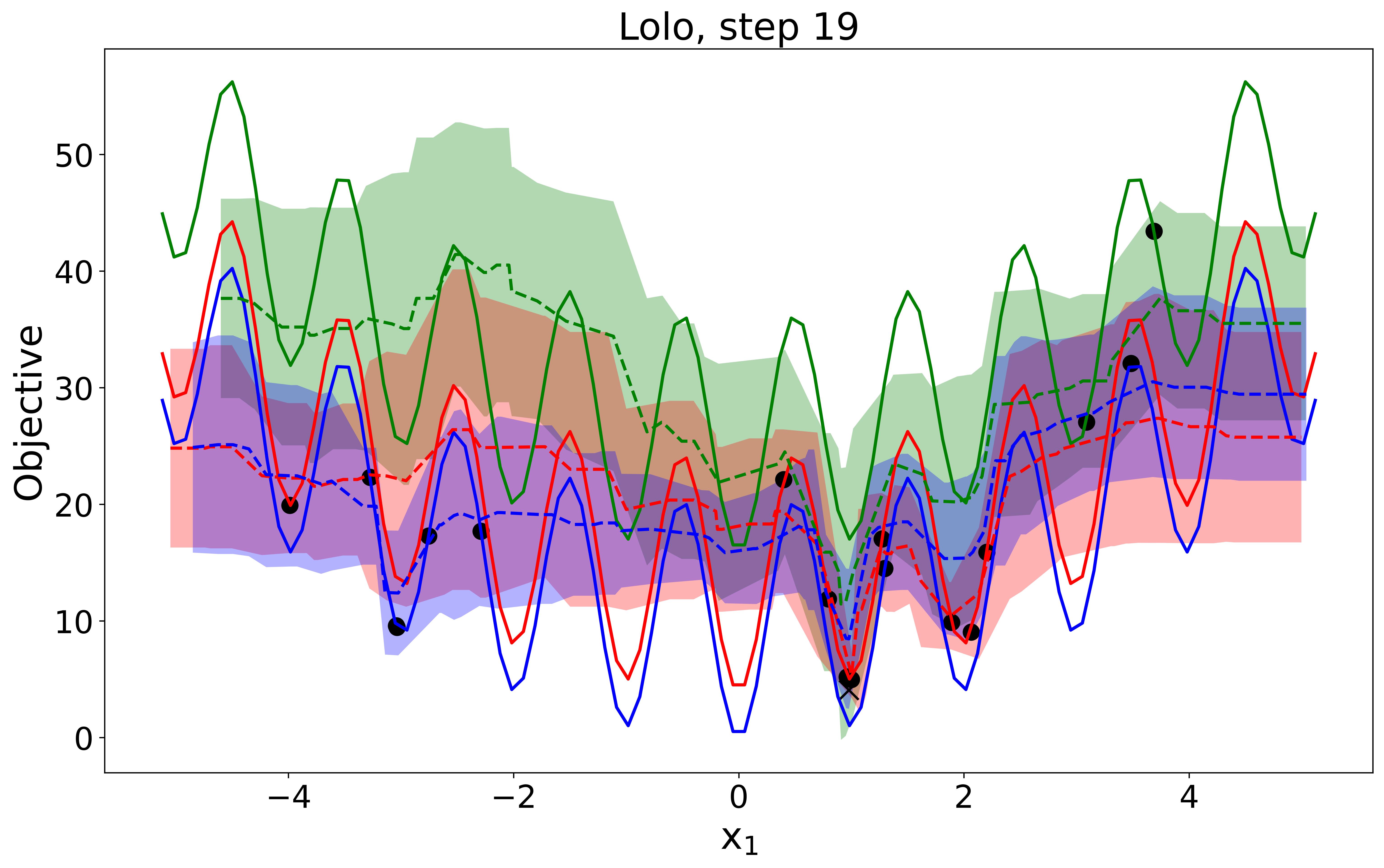

Supplement: Supplementary file 1 — Supplementary Information 1. [file 41598_2022_23431_MOESM1_ESM.zip › Sampling_Sequence_Figures/Rastrigin_Function/rastrigin2_Lolo_19.jpg]

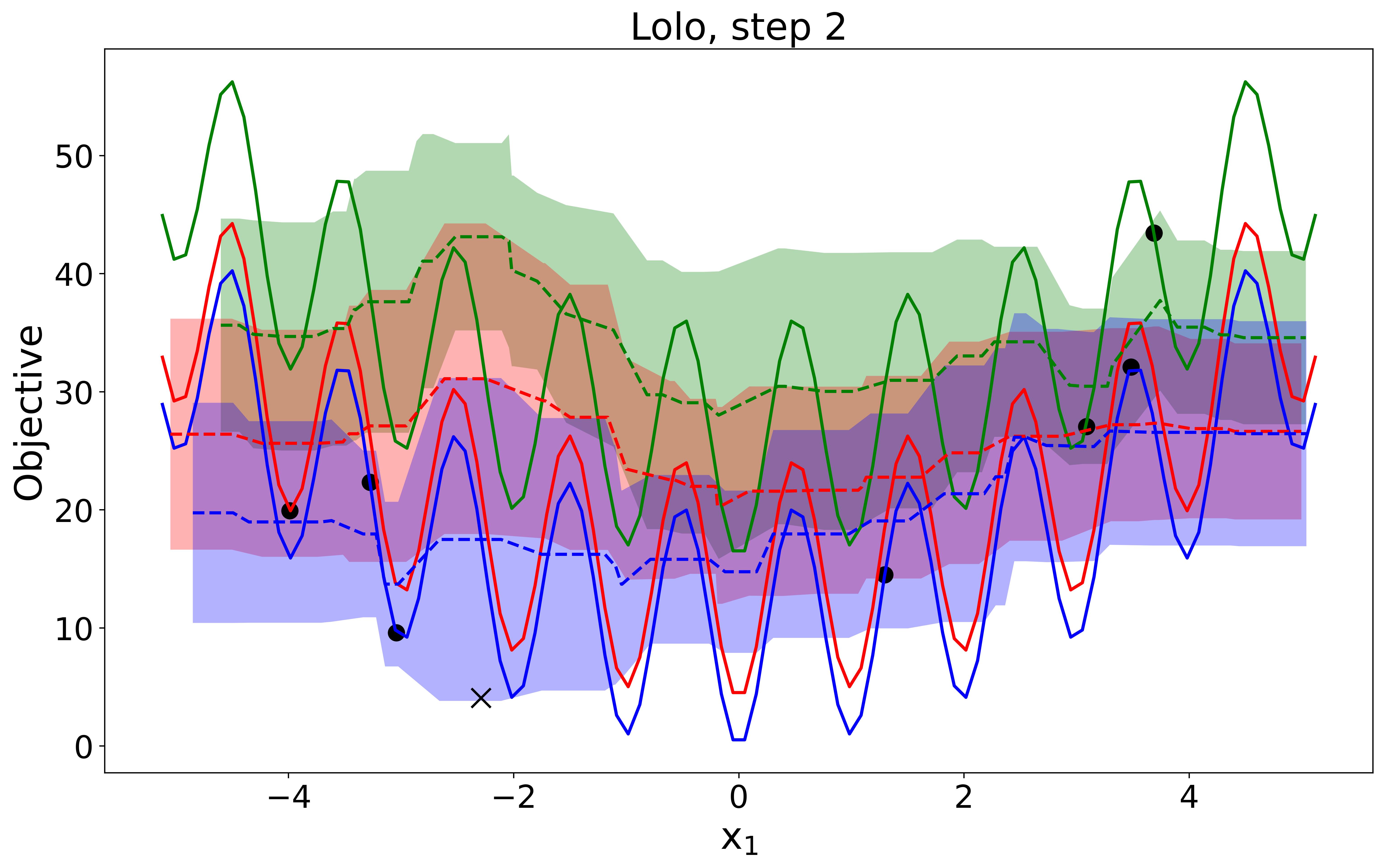

Supplement: Supplementary file 1 — Supplementary Information 1. [file 41598_2022_23431_MOESM1_ESM.zip › Sampling_Sequence_Figures/Rastrigin_Function/rastrigin2_Lolo_2.jpg]

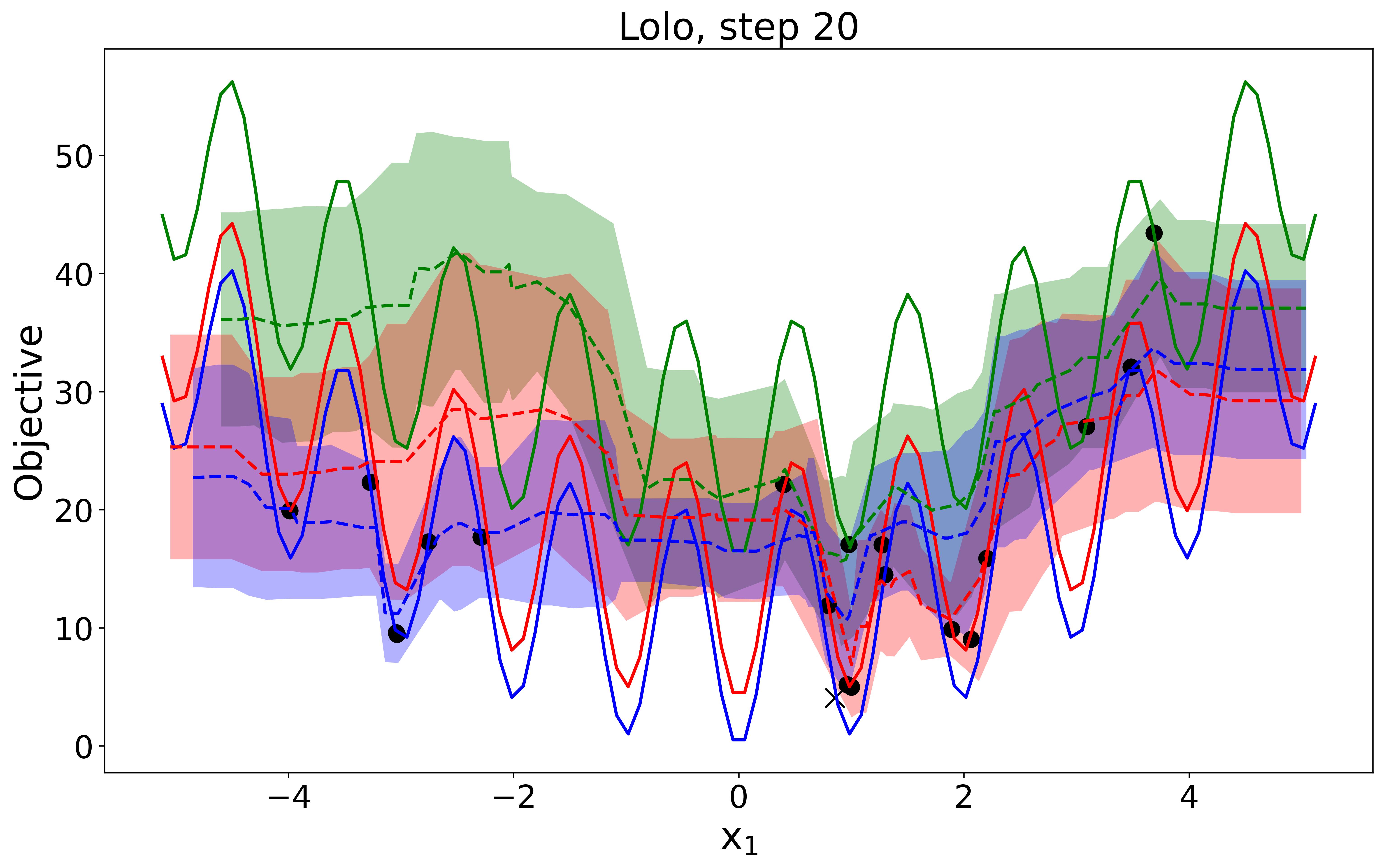

Supplement: Supplementary file 1 — Supplementary Information 1. [file 41598_2022_23431_MOESM1_ESM.zip › Sampling_Sequence_Figures/Rastrigin_Function/rastrigin2_Lolo_20.jpg]

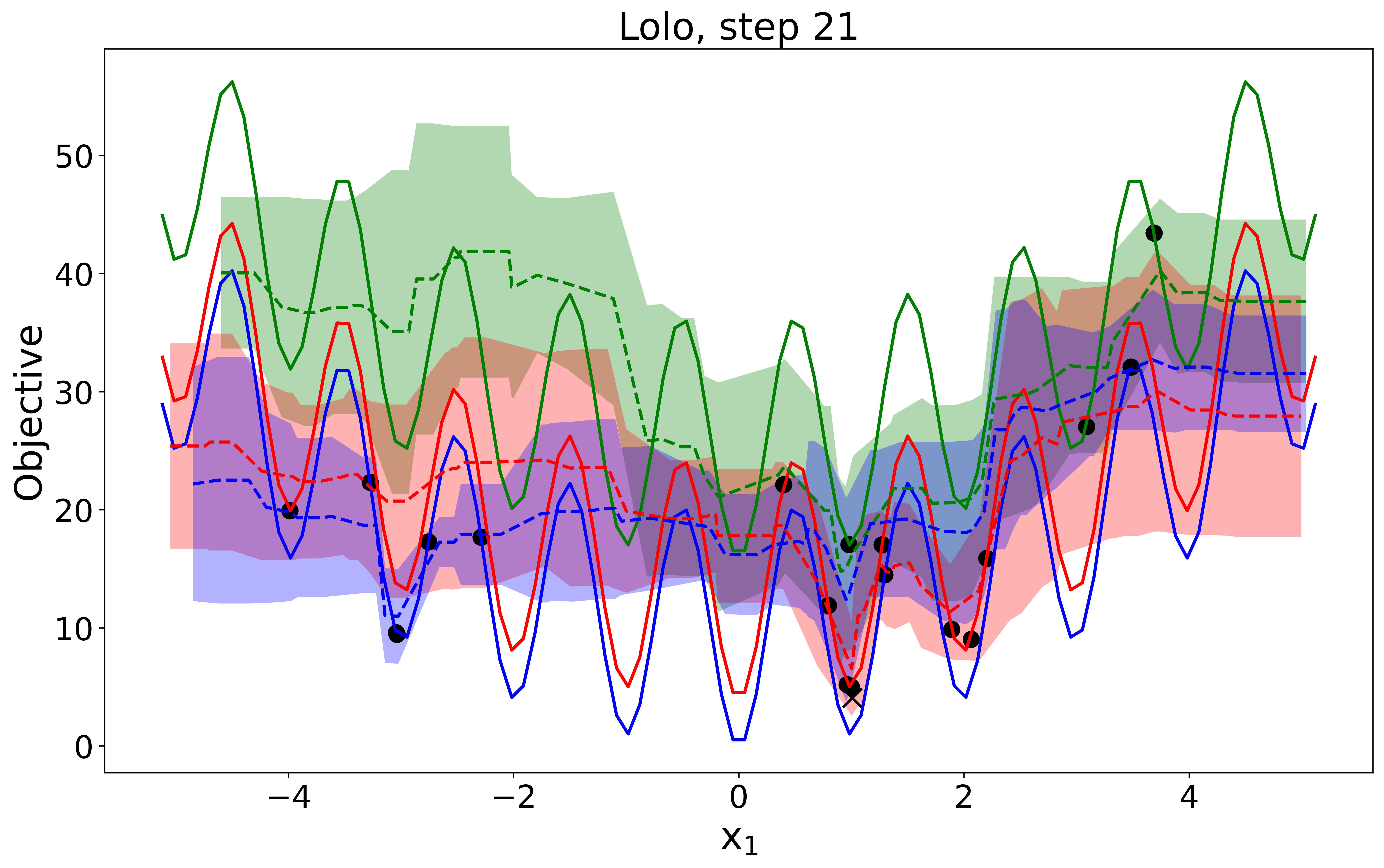

Supplement: Supplementary file 1 — Supplementary Information 1. [file 41598_2022_23431_MOESM1_ESM.zip › Sampling_Sequence_Figures/Rastrigin_Function/rastrigin2_Lolo_21.jpg]

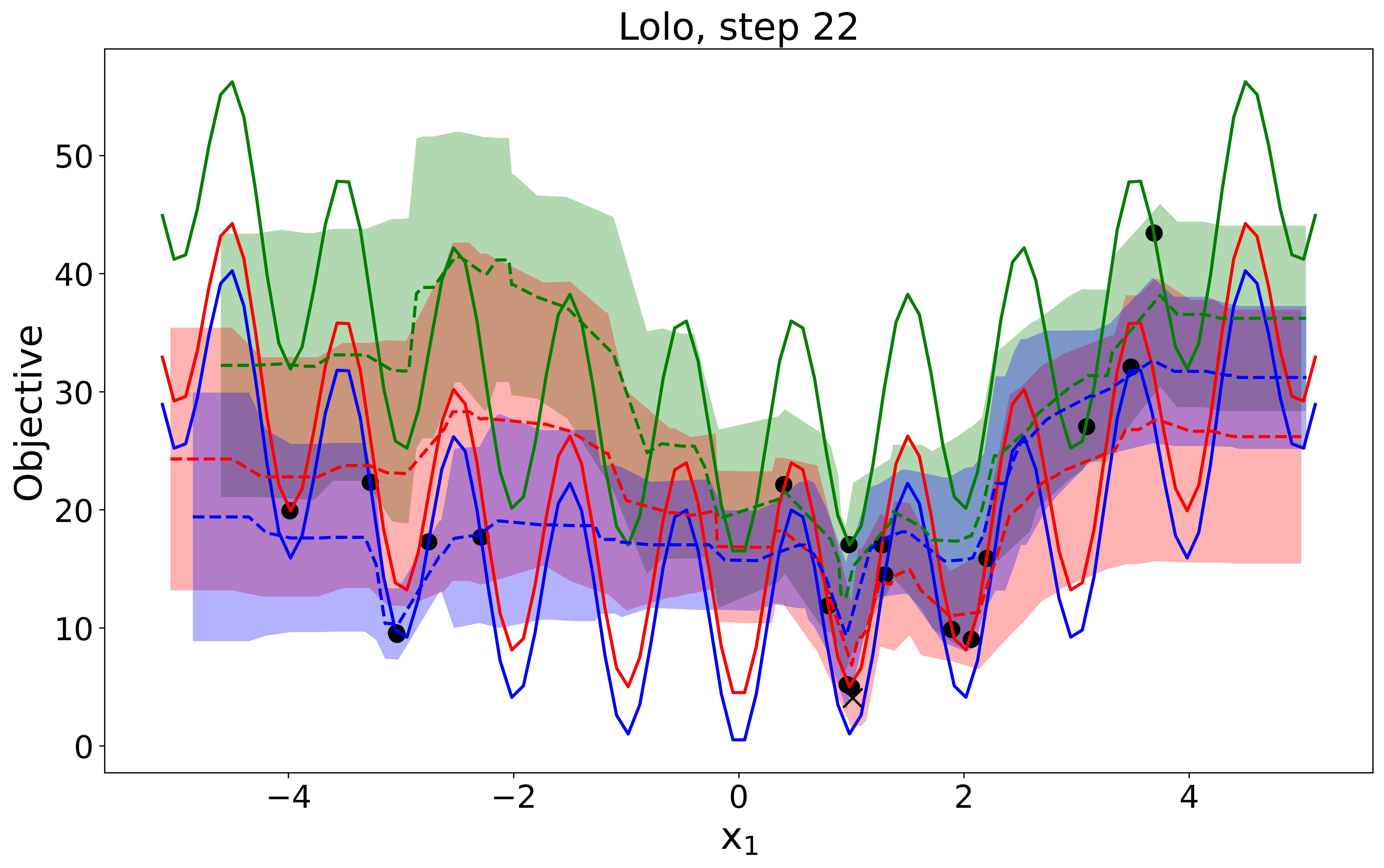

Supplement: Supplementary file 1 — Supplementary Information 1. [file 41598_2022_23431_MOESM1_ESM.zip › Sampling_Sequence_Figures/Rastrigin_Function/rastrigin2_Lolo_22.jpg]

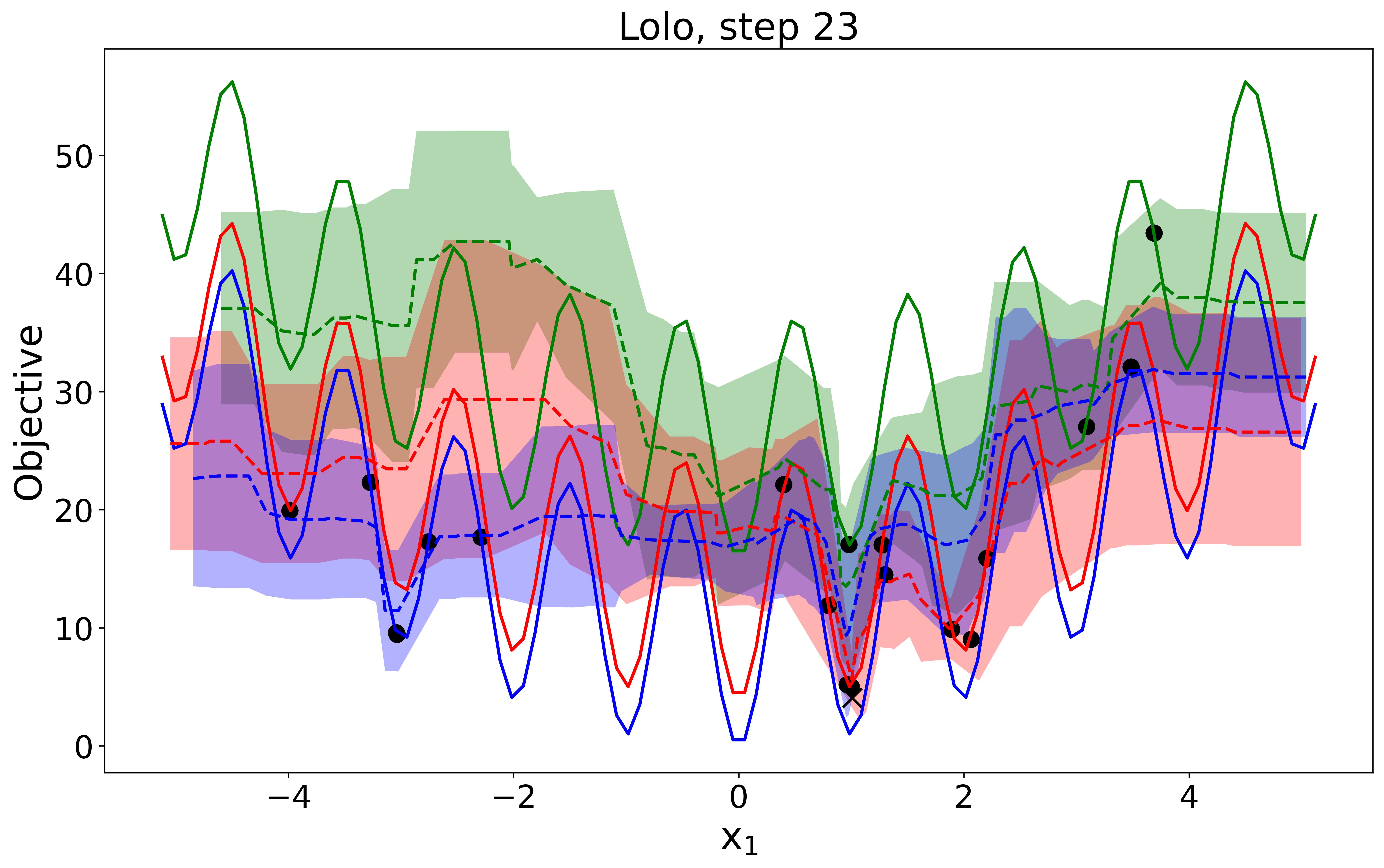

Supplement: Supplementary file 1 — Supplementary Information 1. [file 41598_2022_23431_MOESM1_ESM.zip › Sampling_Sequence_Figures/Rastrigin_Function/rastrigin2_Lolo_23.jpg]

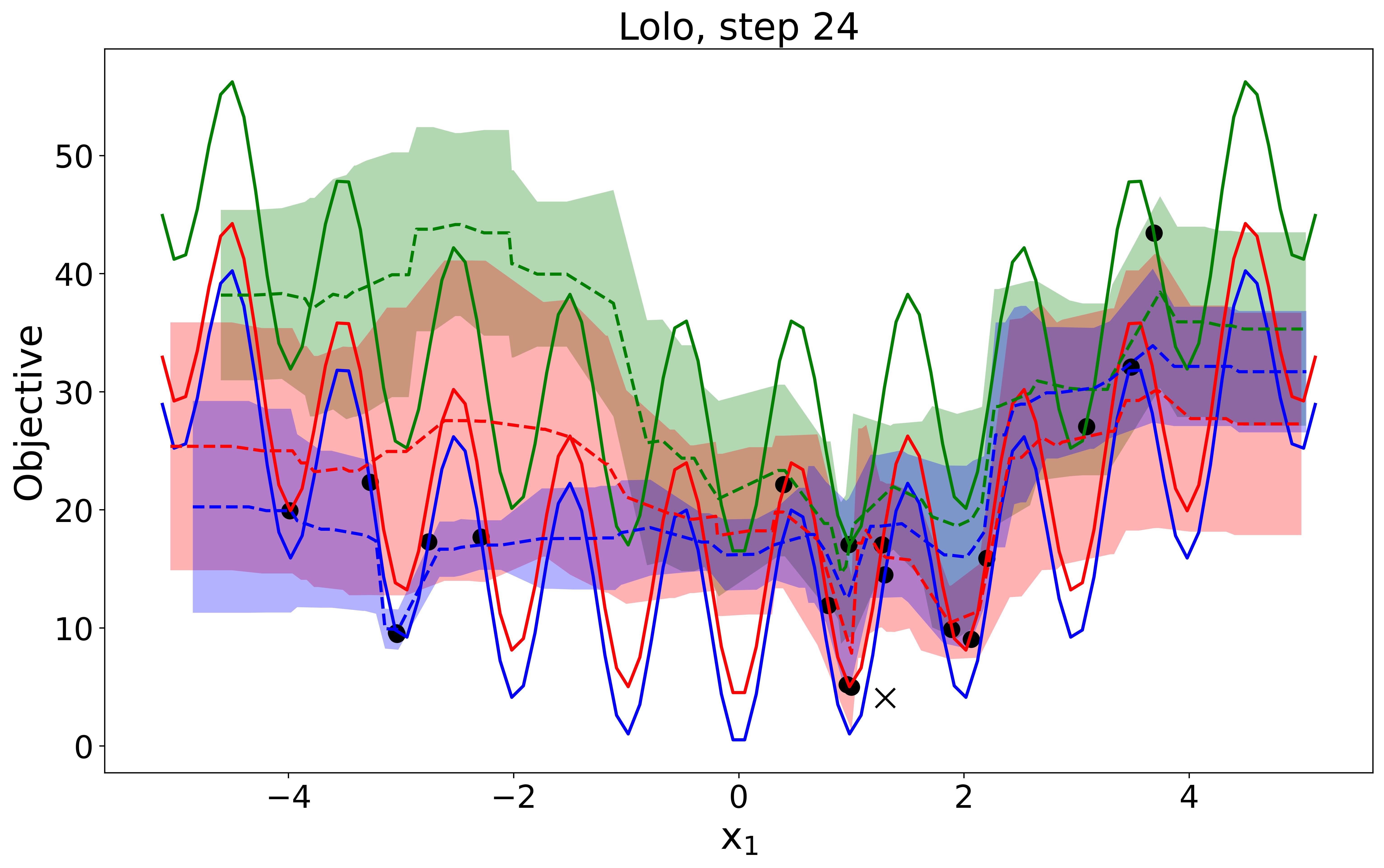

Supplement: Supplementary file 1 — Supplementary Information 1. [file 41598_2022_23431_MOESM1_ESM.zip › Sampling_Sequence_Figures/Rastrigin_Function/rastrigin2_Lolo_24.jpg]

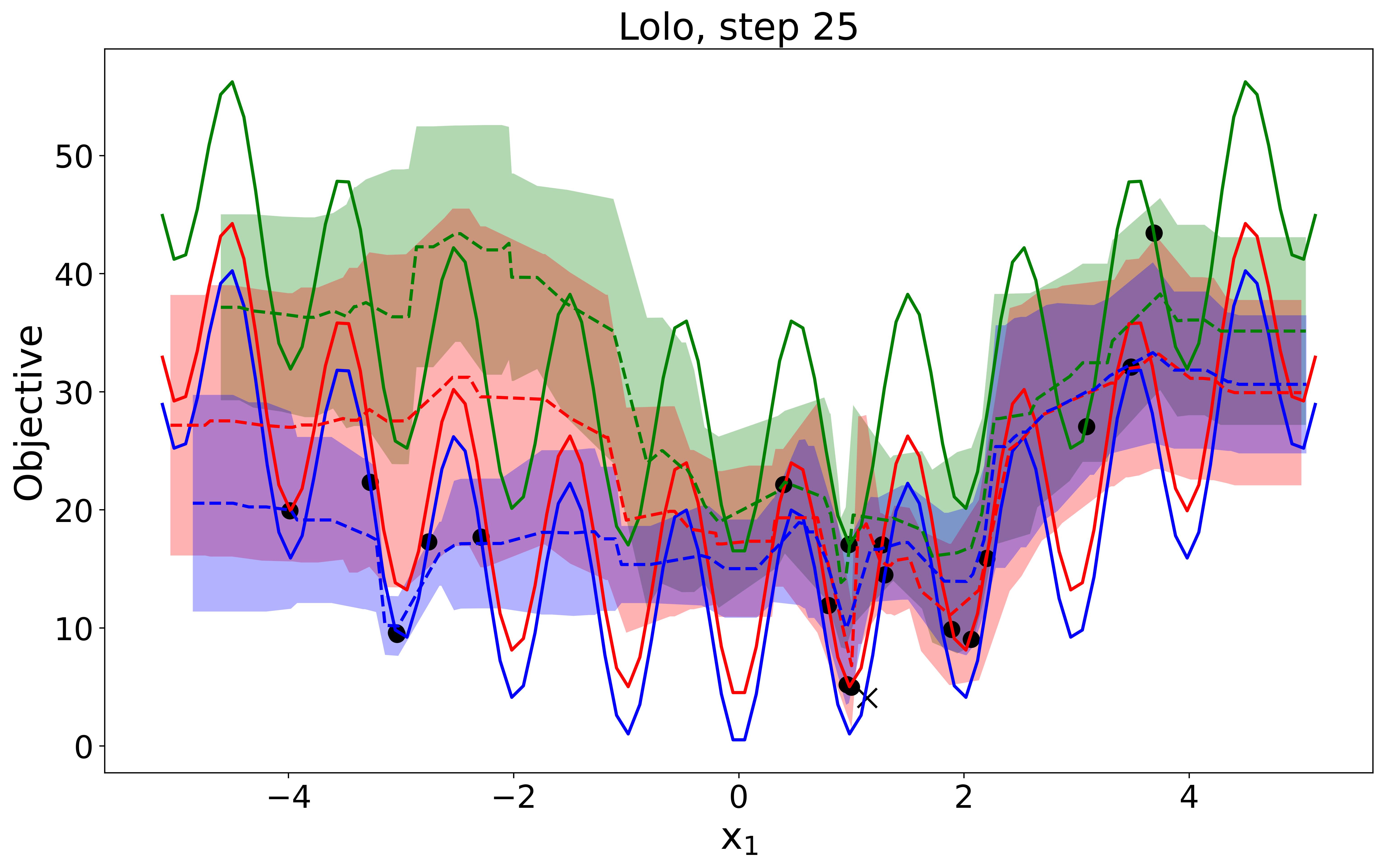

Supplement: Supplementary file 1 — Supplementary Information 1. [file 41598_2022_23431_MOESM1_ESM.zip › Sampling_Sequence_Figures/Rastrigin_Function/rastrigin2_Lolo_25.jpg]

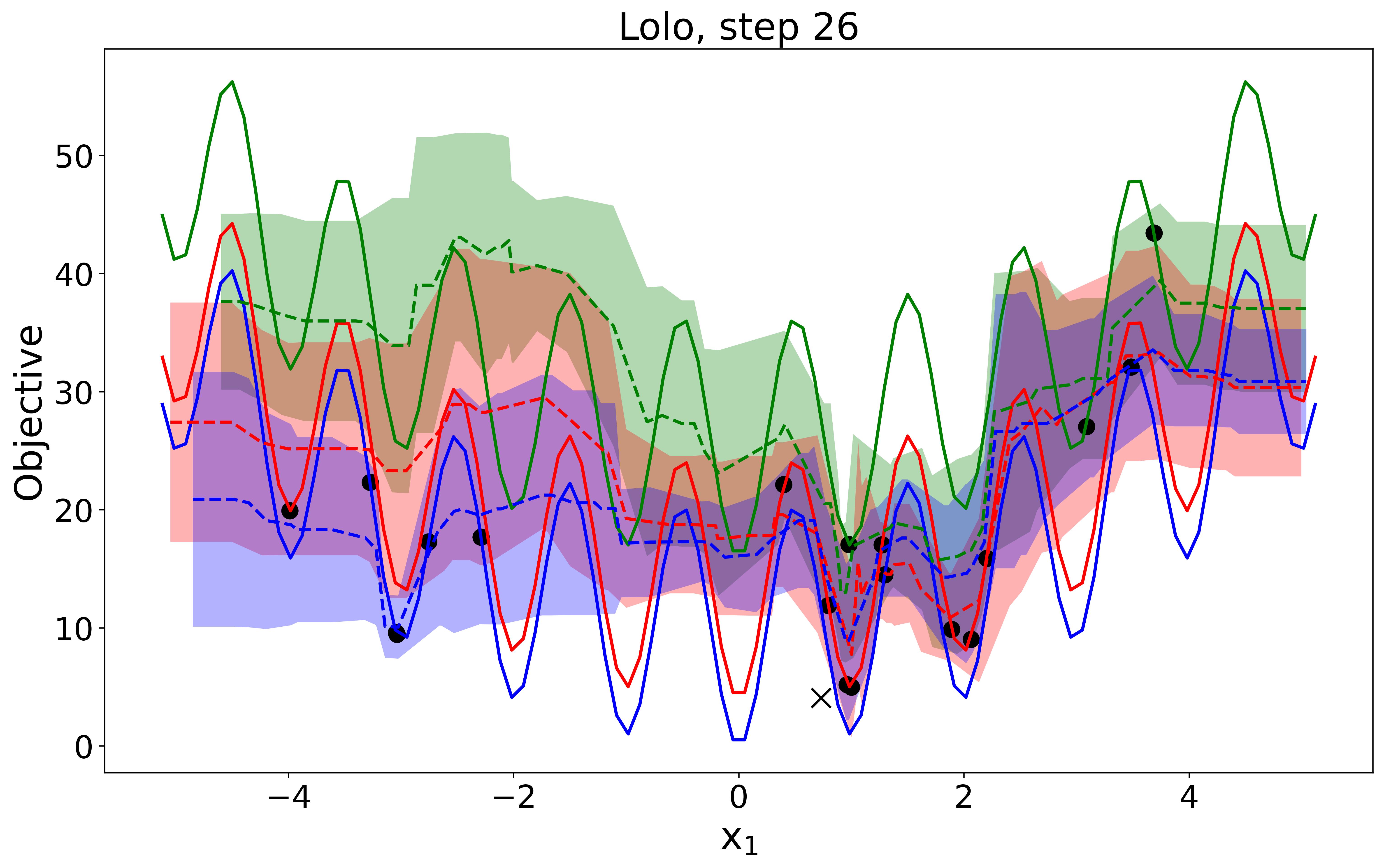

Supplement: Supplementary file 1 — Supplementary Information 1. [file 41598_2022_23431_MOESM1_ESM.zip › Sampling_Sequence_Figures/Rastrigin_Function/rastrigin2_Lolo_26.jpg]

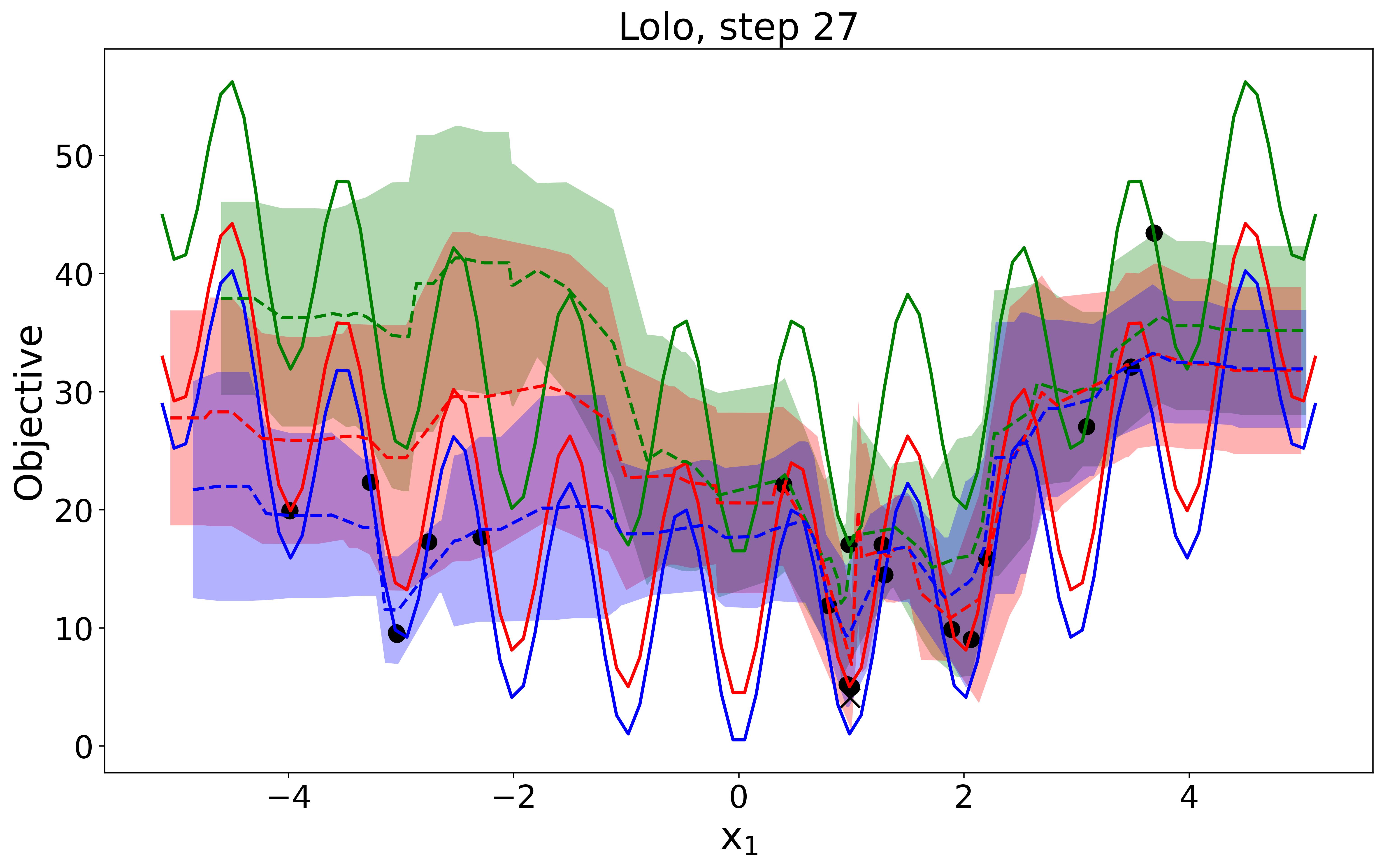

Supplement: Supplementary file 1 — Supplementary Information 1. [file 41598_2022_23431_MOESM1_ESM.zip › Sampling_Sequence_Figures/Rastrigin_Function/rastrigin2_Lolo_27.jpg]

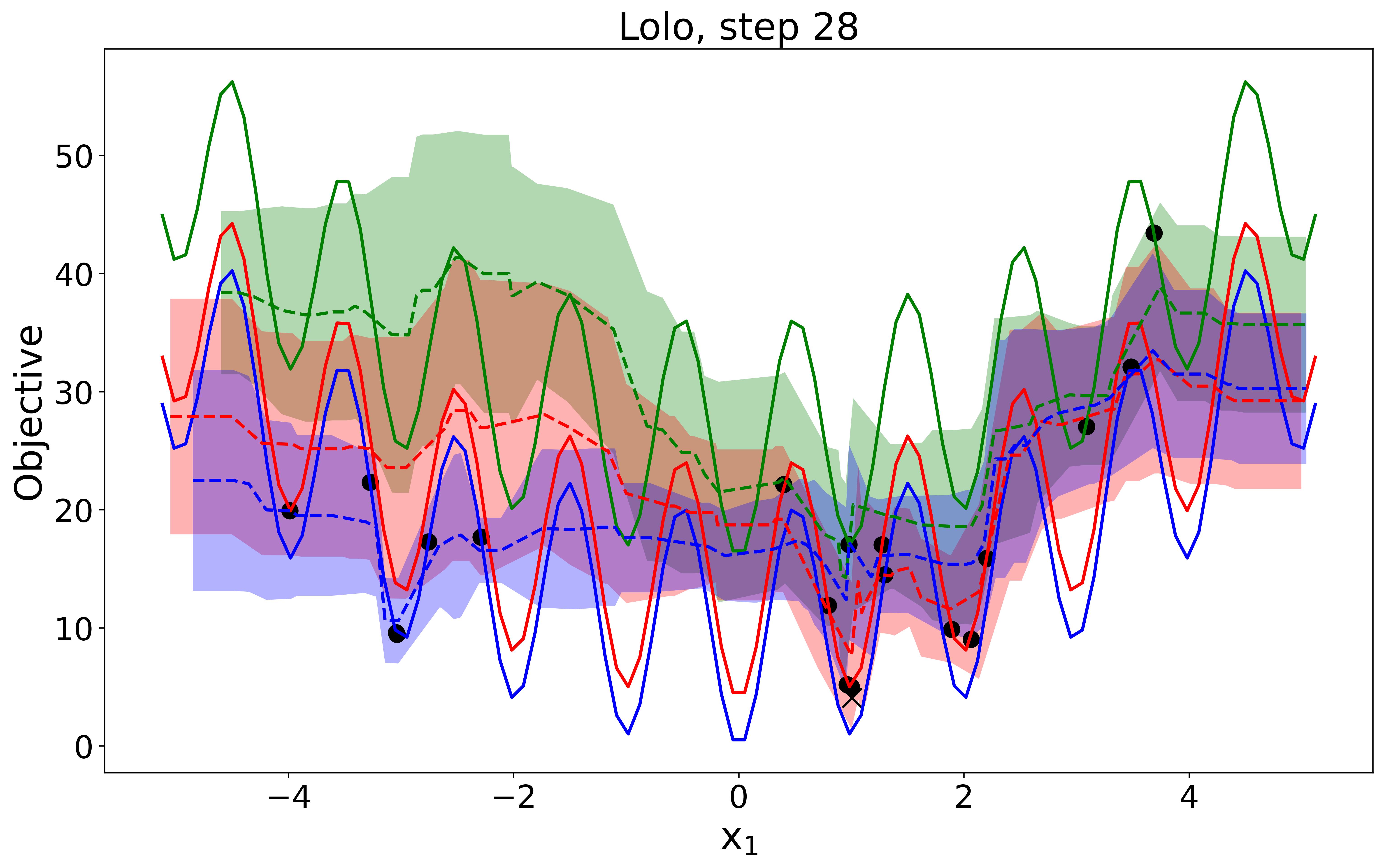

Supplement: Supplementary file 1 — Supplementary Information 1. [file 41598_2022_23431_MOESM1_ESM.zip › Sampling_Sequence_Figures/Rastrigin_Function/rastrigin2_Lolo_28.jpg]

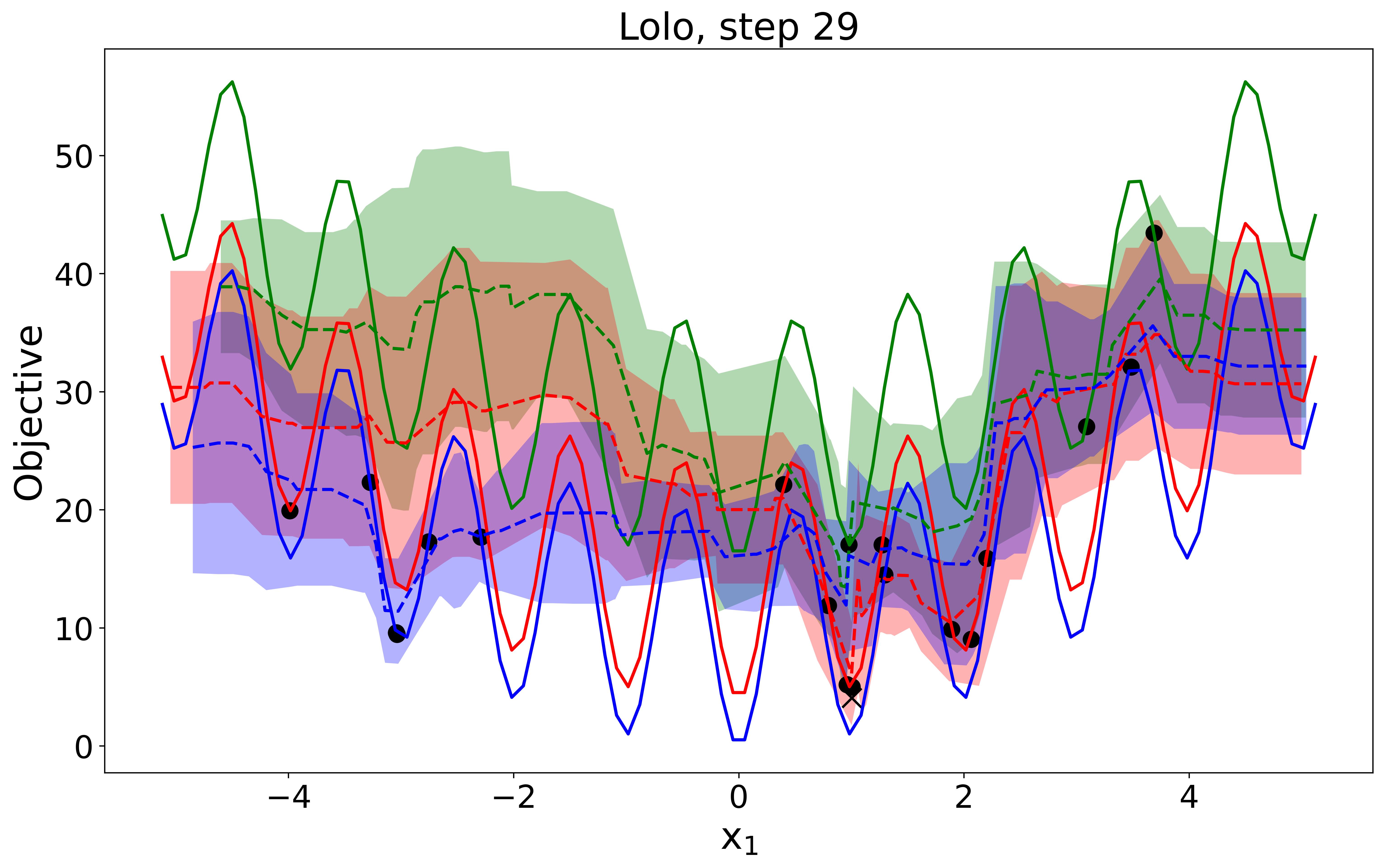

Supplement: Supplementary file 1 — Supplementary Information 1. [file 41598_2022_23431_MOESM1_ESM.zip › Sampling_Sequence_Figures/Rastrigin_Function/rastrigin2_Lolo_29.jpg]

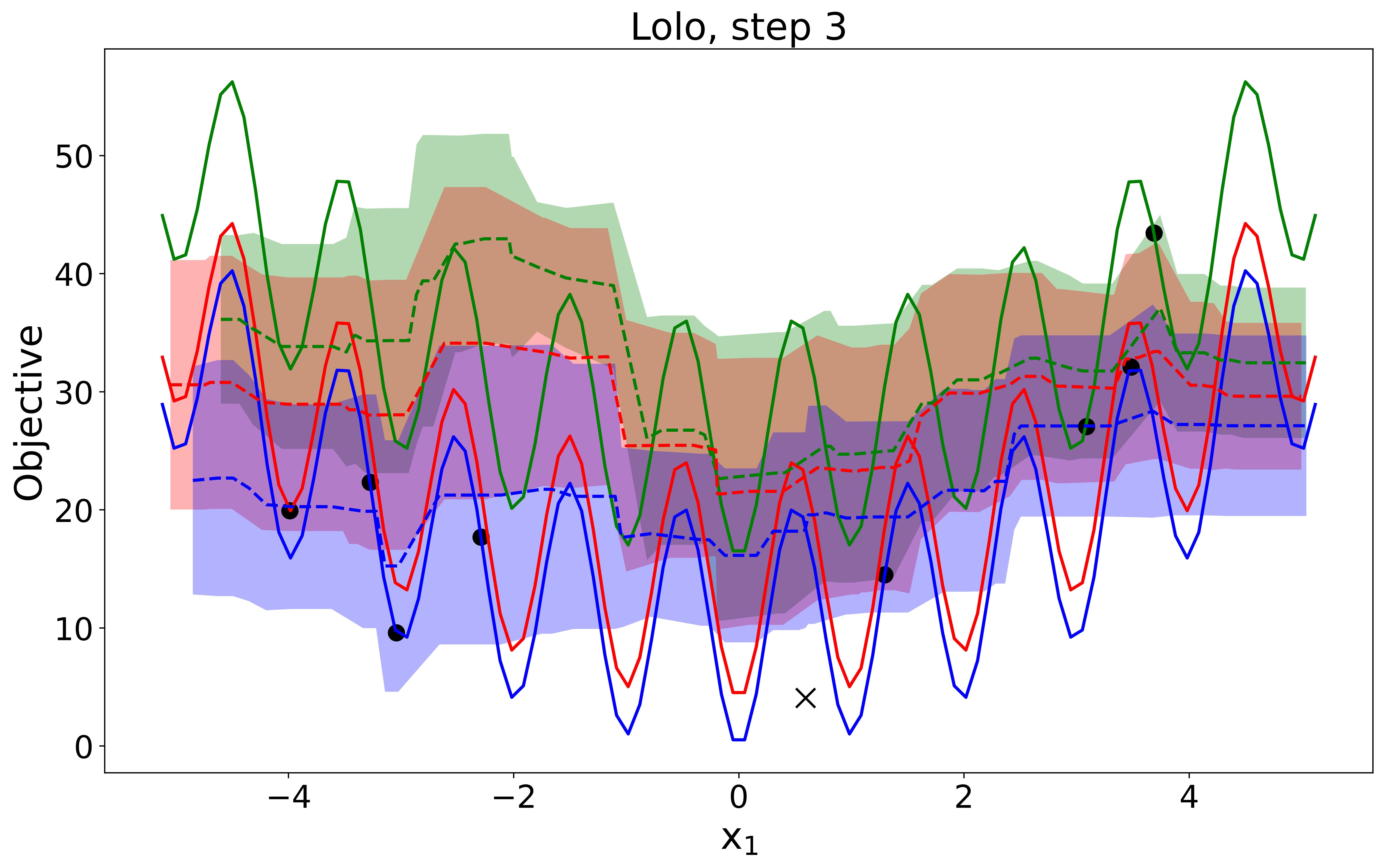

Supplement: Supplementary file 1 — Supplementary Information 1. [file 41598_2022_23431_MOESM1_ESM.zip › Sampling_Sequence_Figures/Rastrigin_Function/rastrigin2_Lolo_3.jpg]

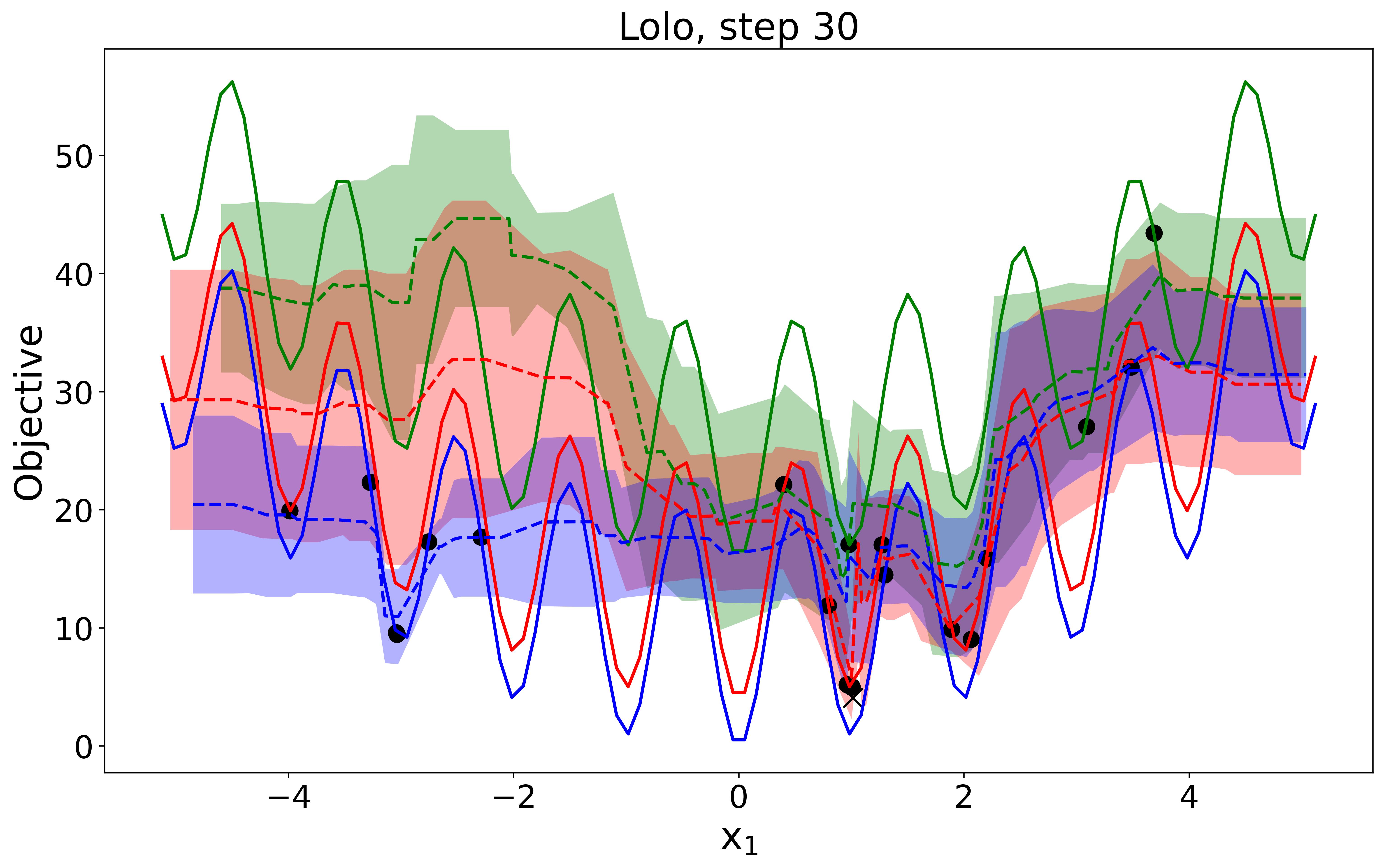

Supplement: Supplementary file 1 — Supplementary Information 1. [file 41598_2022_23431_MOESM1_ESM.zip › Sampling_Sequence_Figures/Rastrigin_Function/rastrigin2_Lolo_30.jpg]

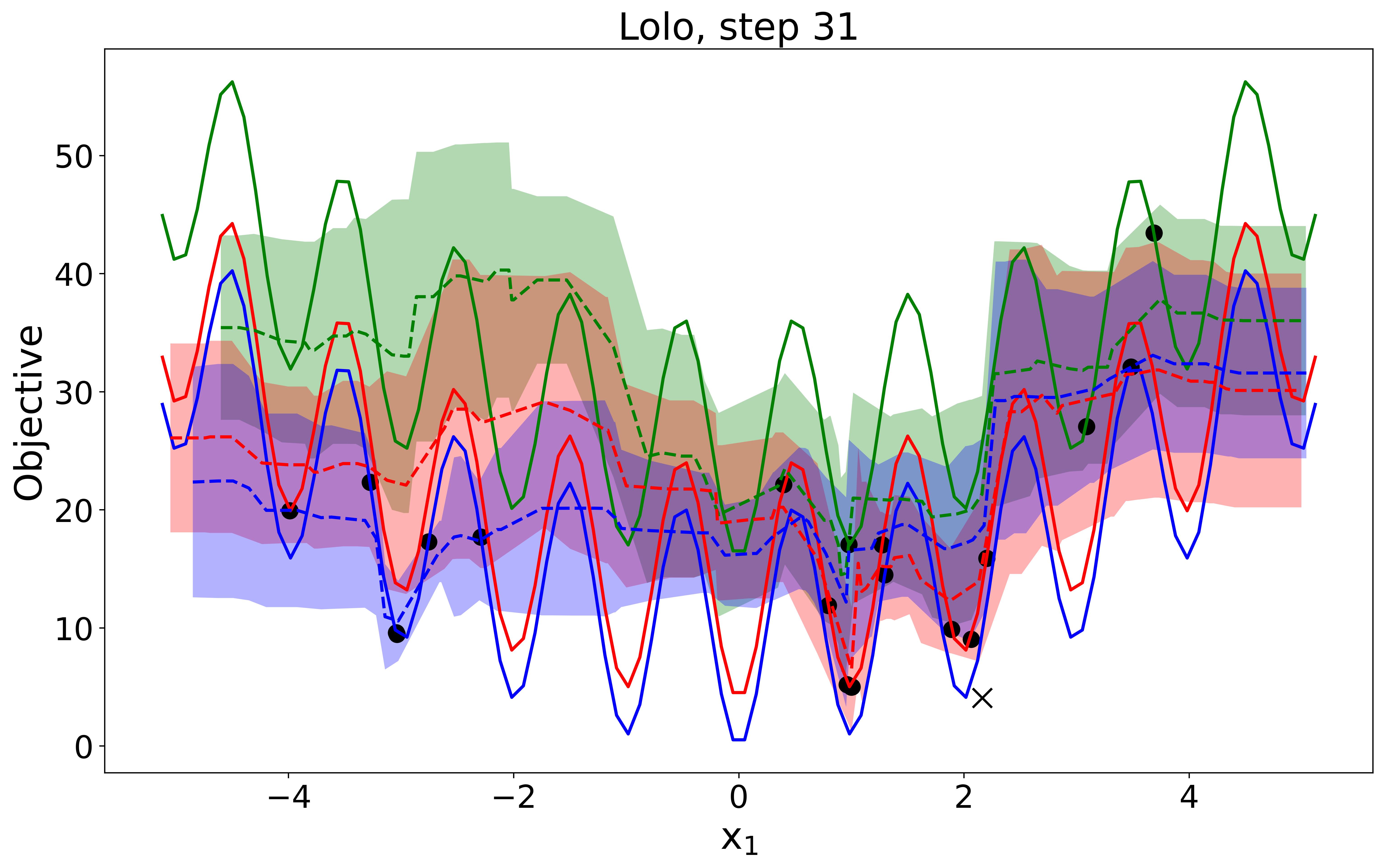

Supplement: Supplementary file 1 — Supplementary Information 1. [file 41598_2022_23431_MOESM1_ESM.zip › Sampling_Sequence_Figures/Rastrigin_Function/rastrigin2_Lolo_31.jpg]

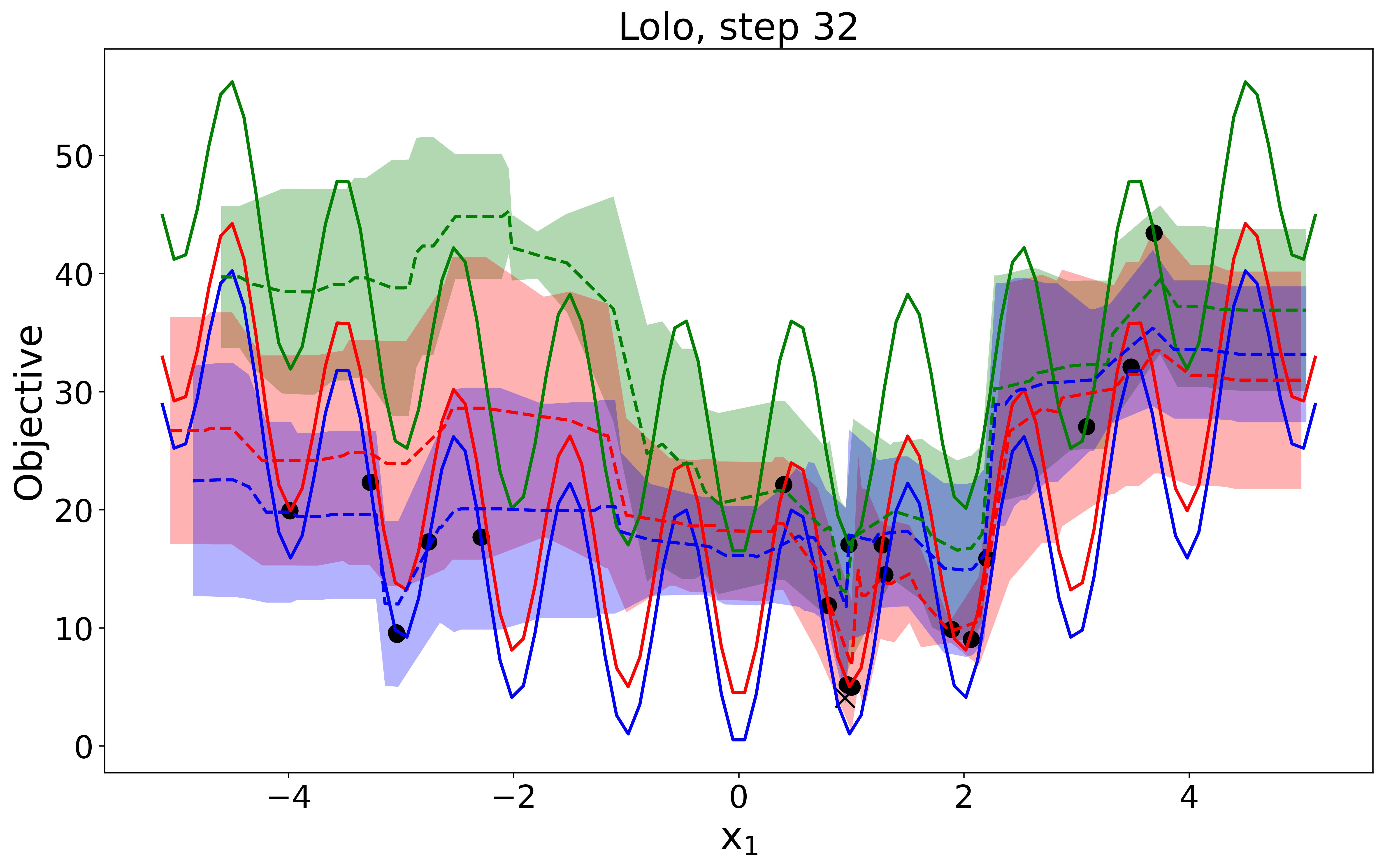

Supplement: Supplementary file 1 — Supplementary Information 1. [file 41598_2022_23431_MOESM1_ESM.zip › Sampling_Sequence_Figures/Rastrigin_Function/rastrigin2_Lolo_32.jpg]

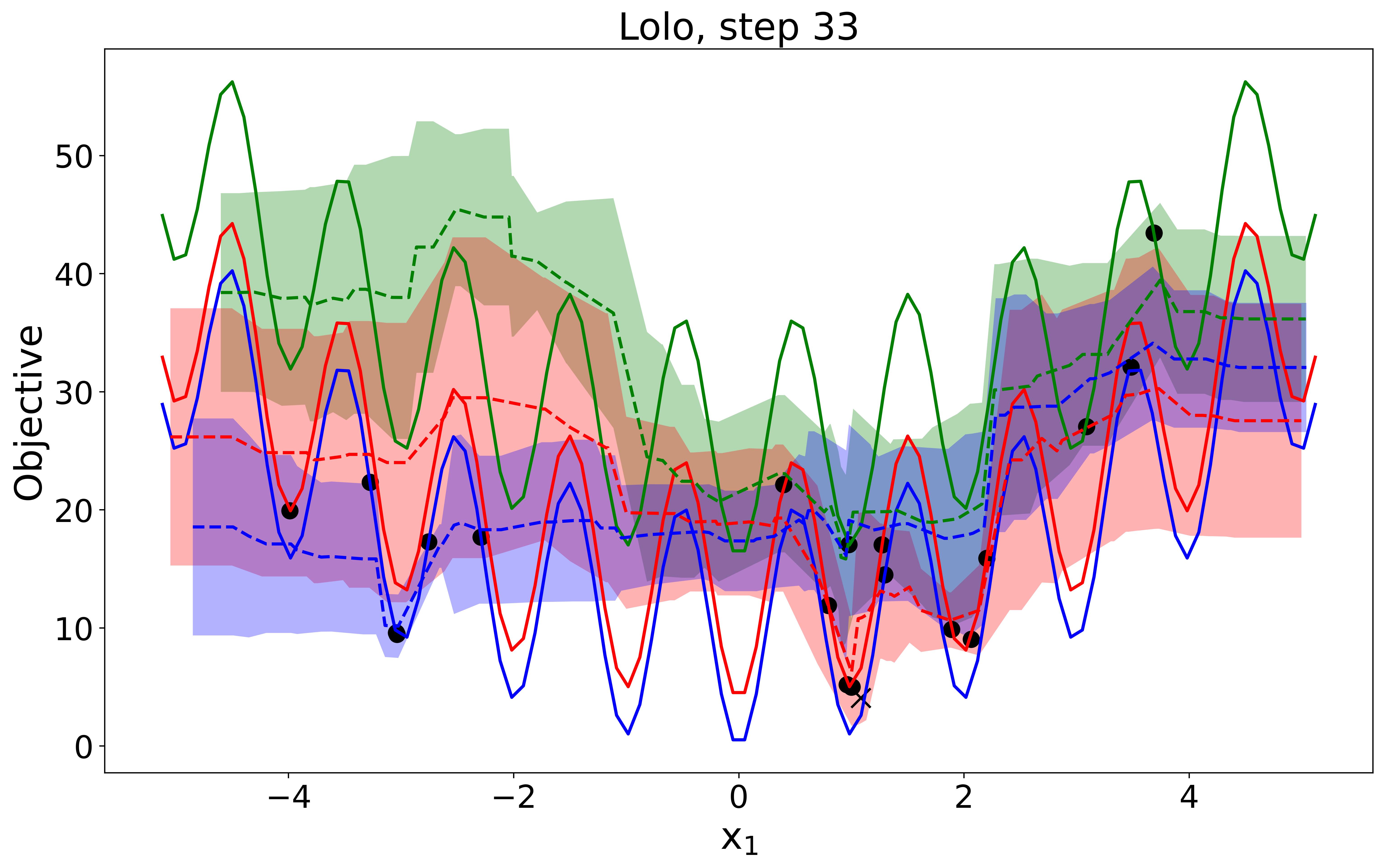

Supplement: Supplementary file 1 — Supplementary Information 1. [file 41598_2022_23431_MOESM1_ESM.zip › Sampling_Sequence_Figures/Rastrigin_Function/rastrigin2_Lolo_33.jpg]

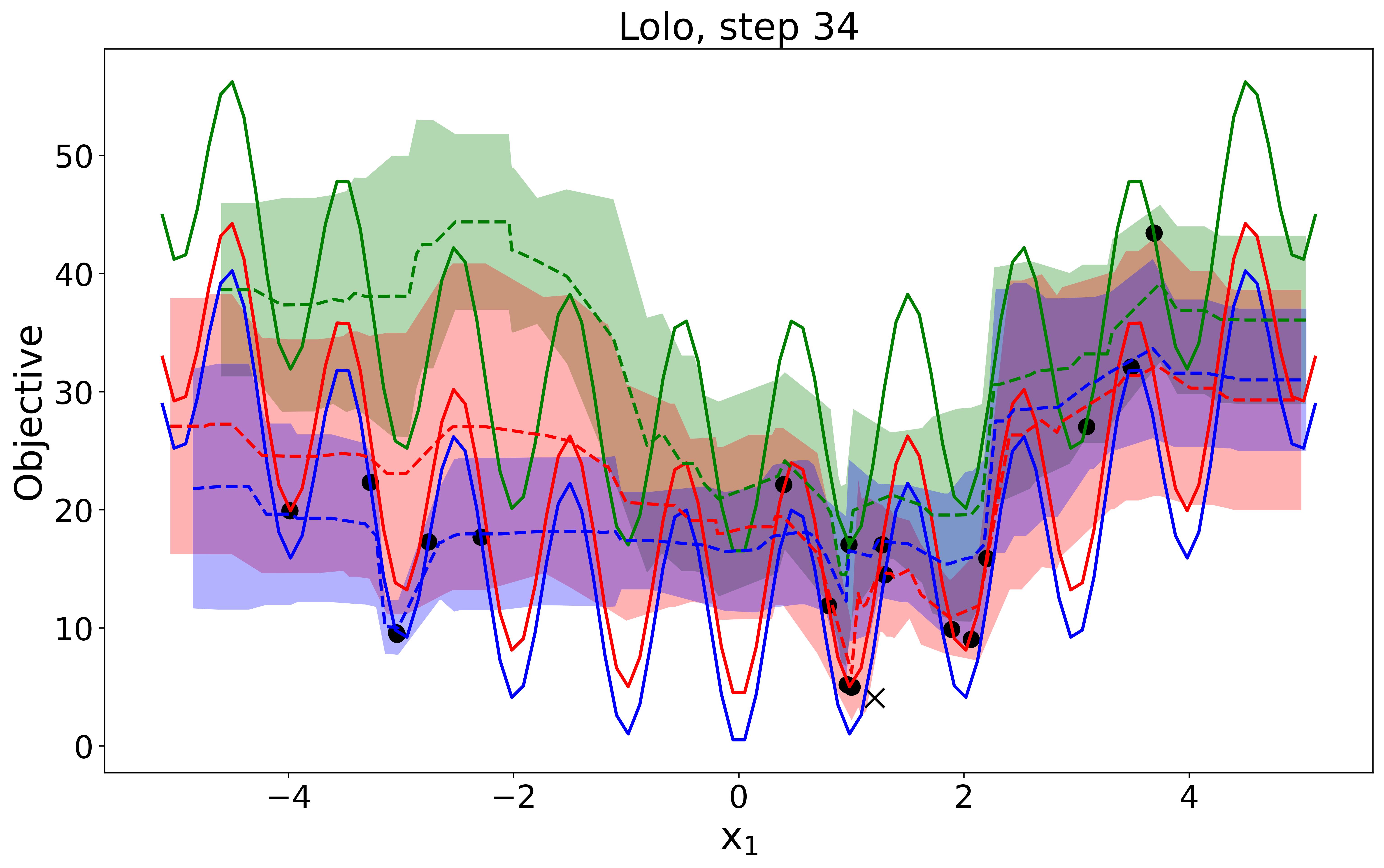

Supplement: Supplementary file 1 — Supplementary Information 1. [file 41598_2022_23431_MOESM1_ESM.zip › Sampling_Sequence_Figures/Rastrigin_Function/rastrigin2_Lolo_34.jpg]

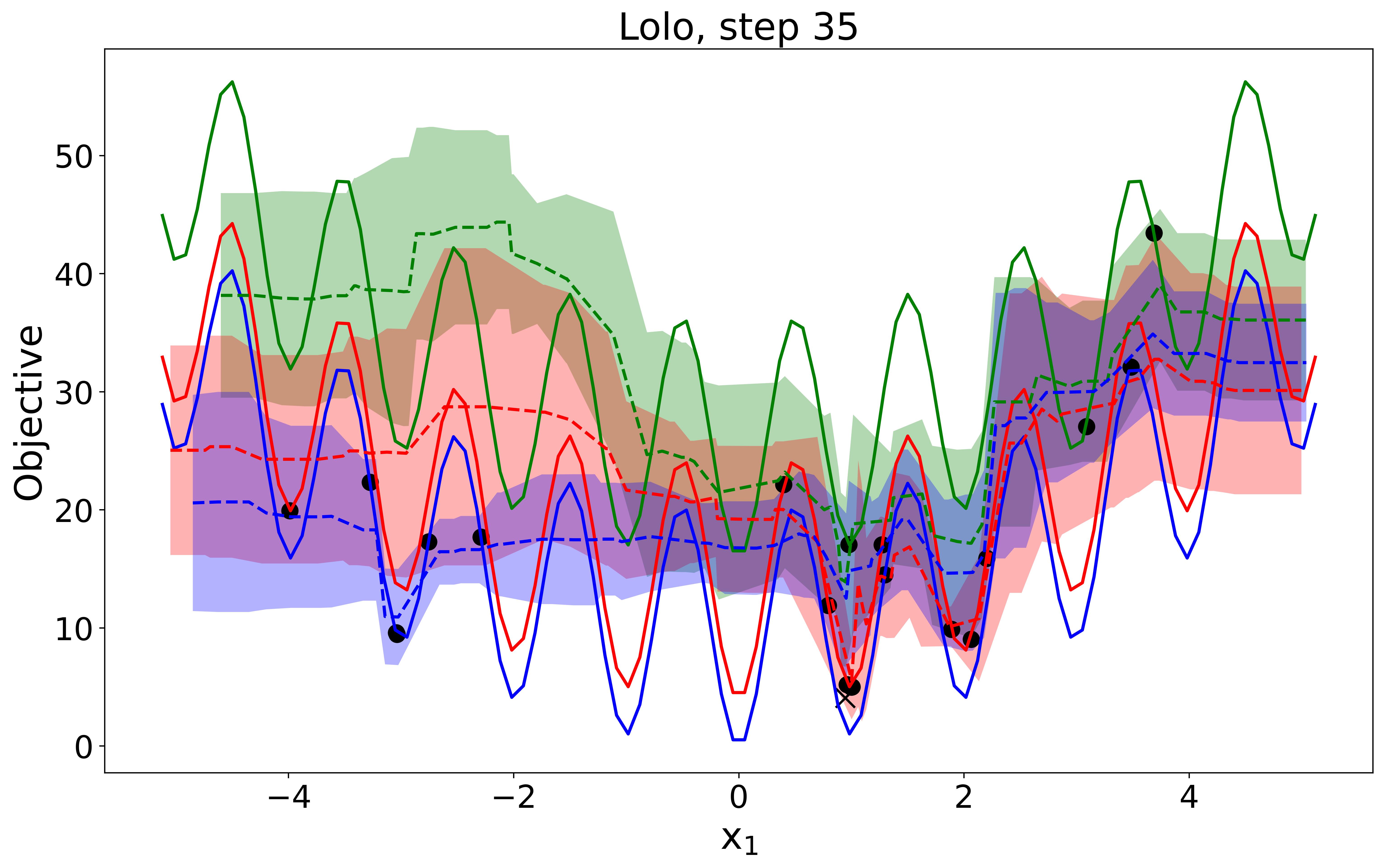

Supplement: Supplementary file 1 — Supplementary Information 1. [file 41598_2022_23431_MOESM1_ESM.zip › Sampling_Sequence_Figures/Rastrigin_Function/rastrigin2_Lolo_35.jpg]

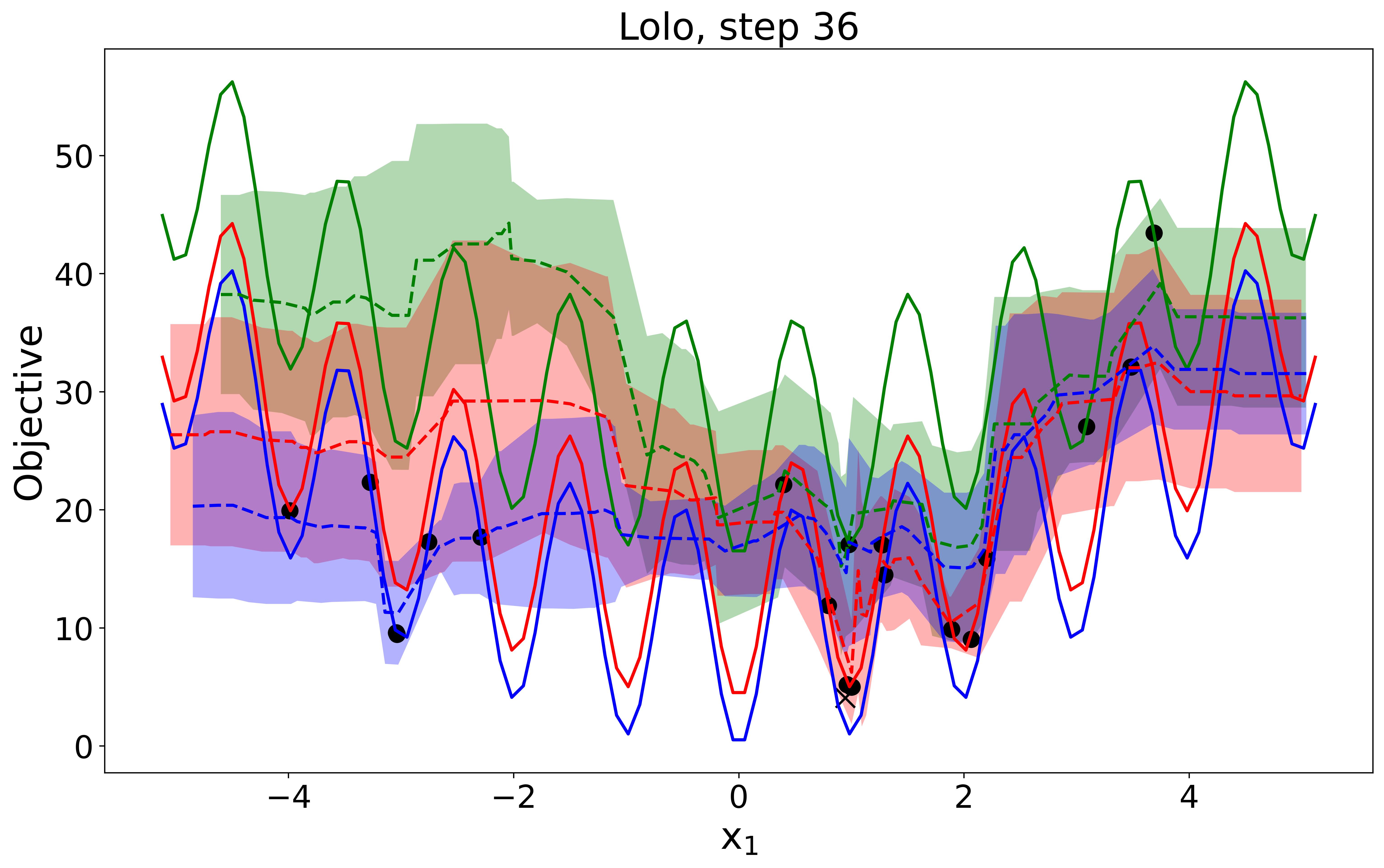

Supplement: Supplementary file 1 — Supplementary Information 1. [file 41598_2022_23431_MOESM1_ESM.zip › Sampling_Sequence_Figures/Rastrigin_Function/rastrigin2_Lolo_36.jpg]

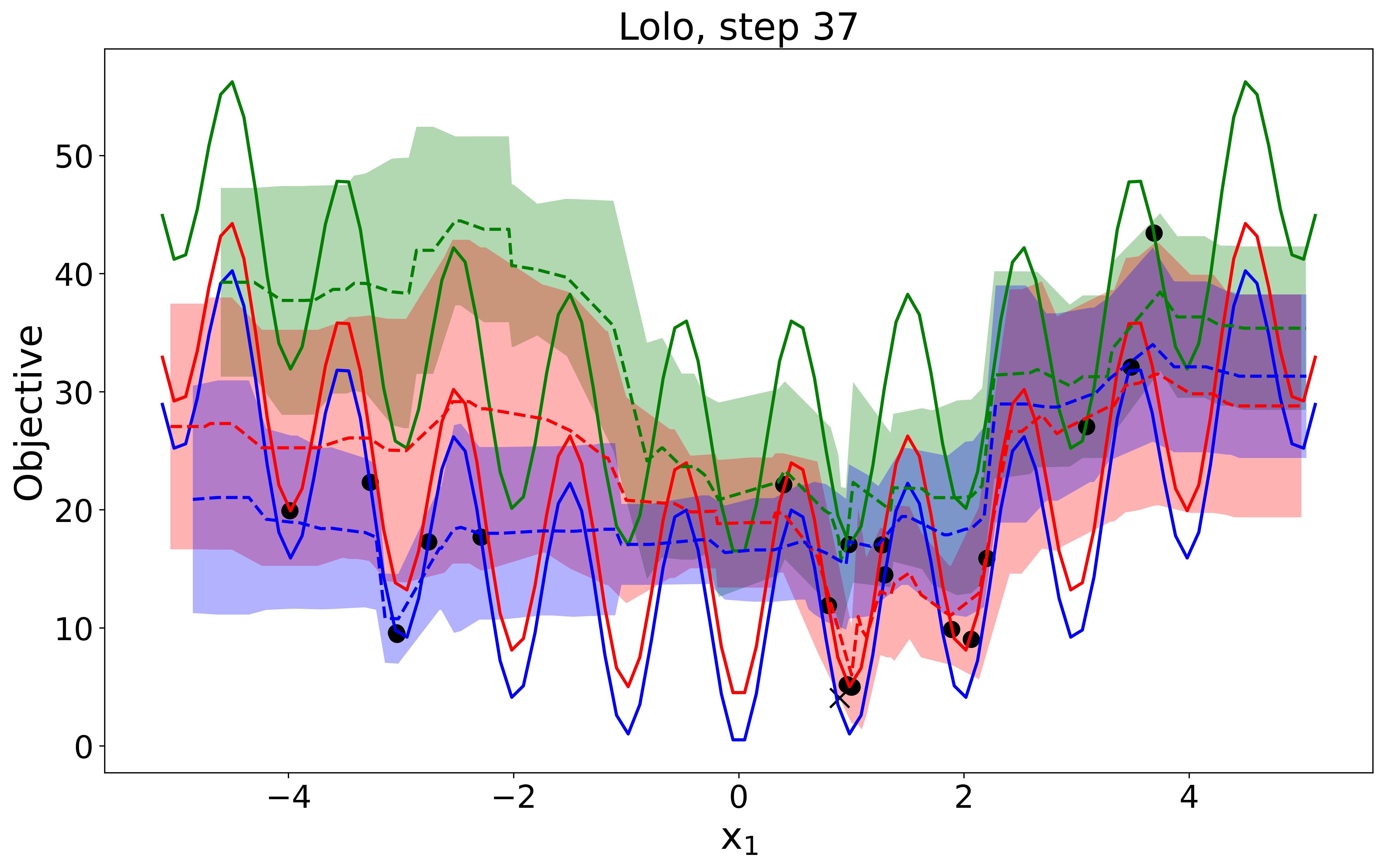

Supplement: Supplementary file 1 — Supplementary Information 1. [file 41598_2022_23431_MOESM1_ESM.zip › Sampling_Sequence_Figures/Rastrigin_Function/rastrigin2_Lolo_37.jpg]

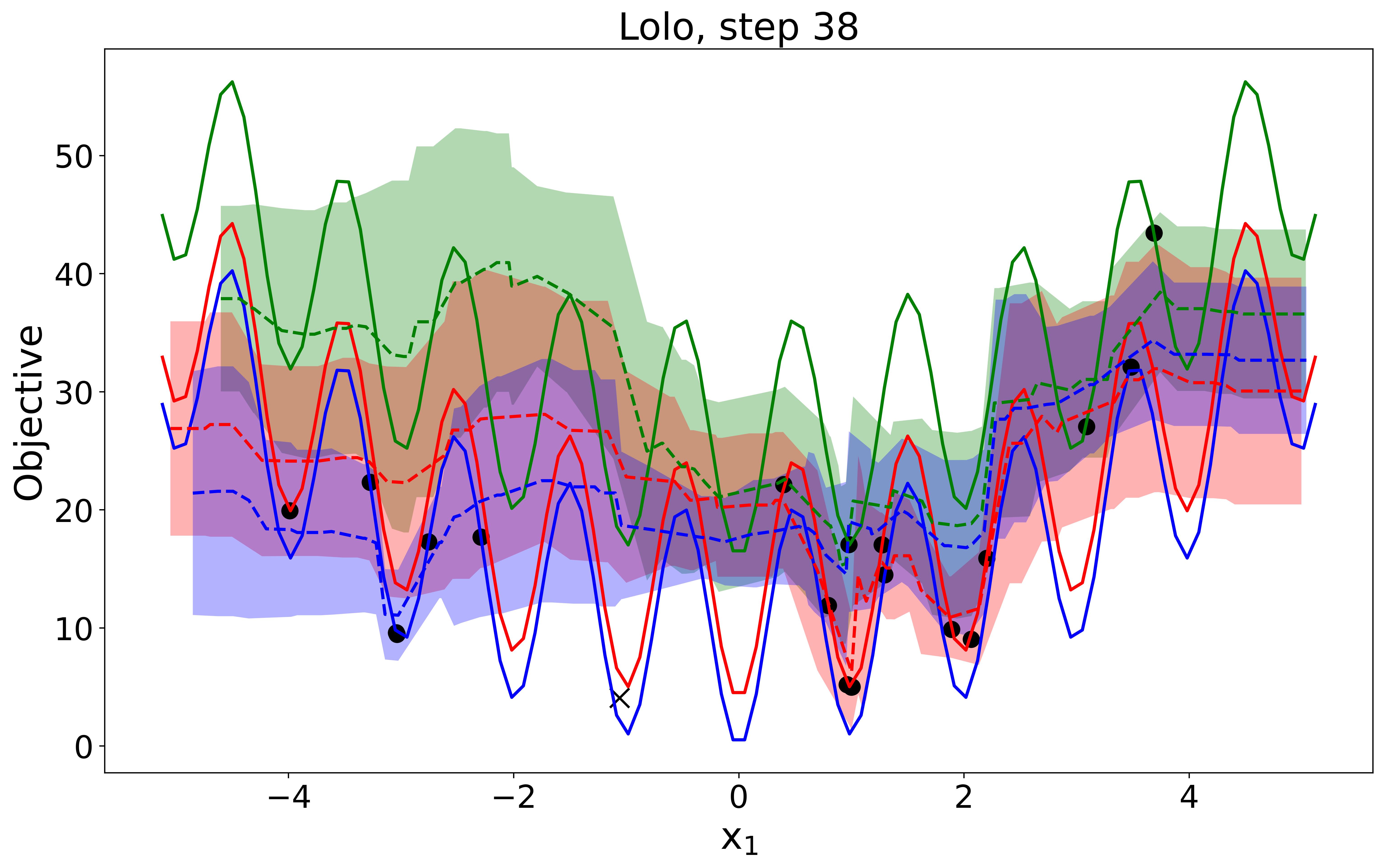

Supplement: Supplementary file 1 — Supplementary Information 1. [file 41598_2022_23431_MOESM1_ESM.zip › Sampling_Sequence_Figures/Rastrigin_Function/rastrigin2_Lolo_38.jpg]

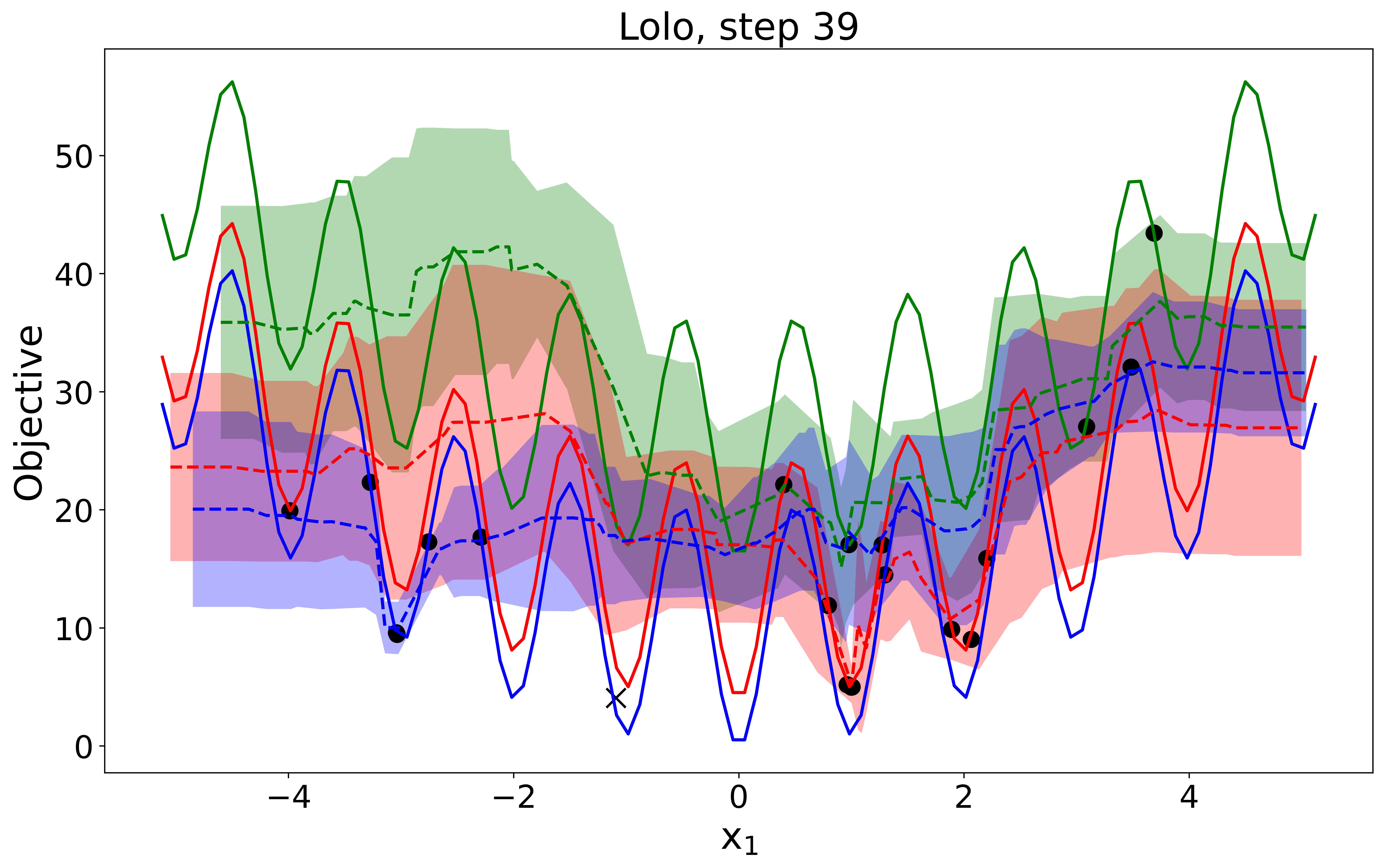

Supplement: Supplementary file 1 — Supplementary Information 1. [file 41598_2022_23431_MOESM1_ESM.zip › Sampling_Sequence_Figures/Rastrigin_Function/rastrigin2_Lolo_39.jpg]

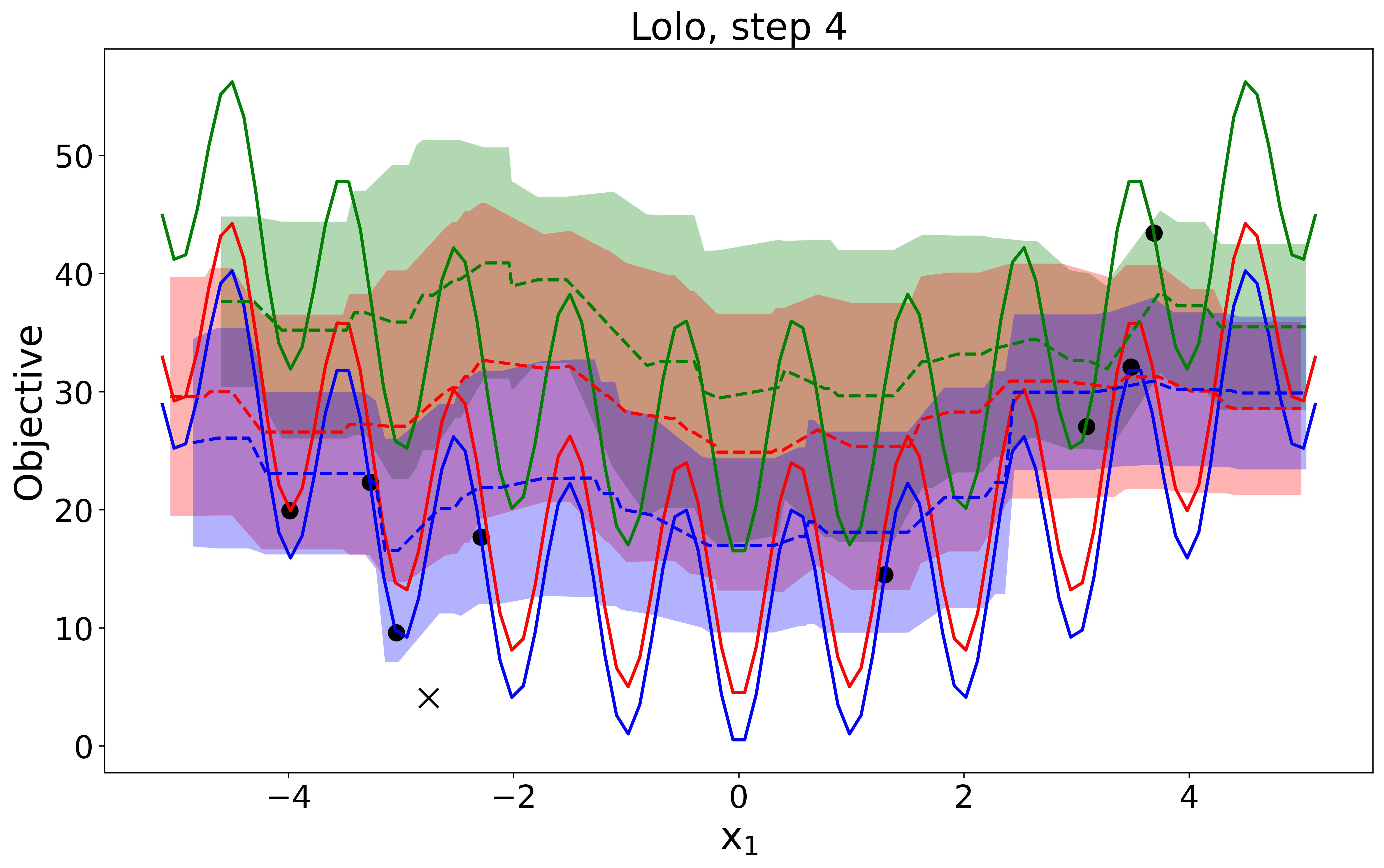

Supplement: Supplementary file 1 — Supplementary Information 1. [file 41598_2022_23431_MOESM1_ESM.zip › Sampling_Sequence_Figures/Rastrigin_Function/rastrigin2_Lolo_4.jpg]

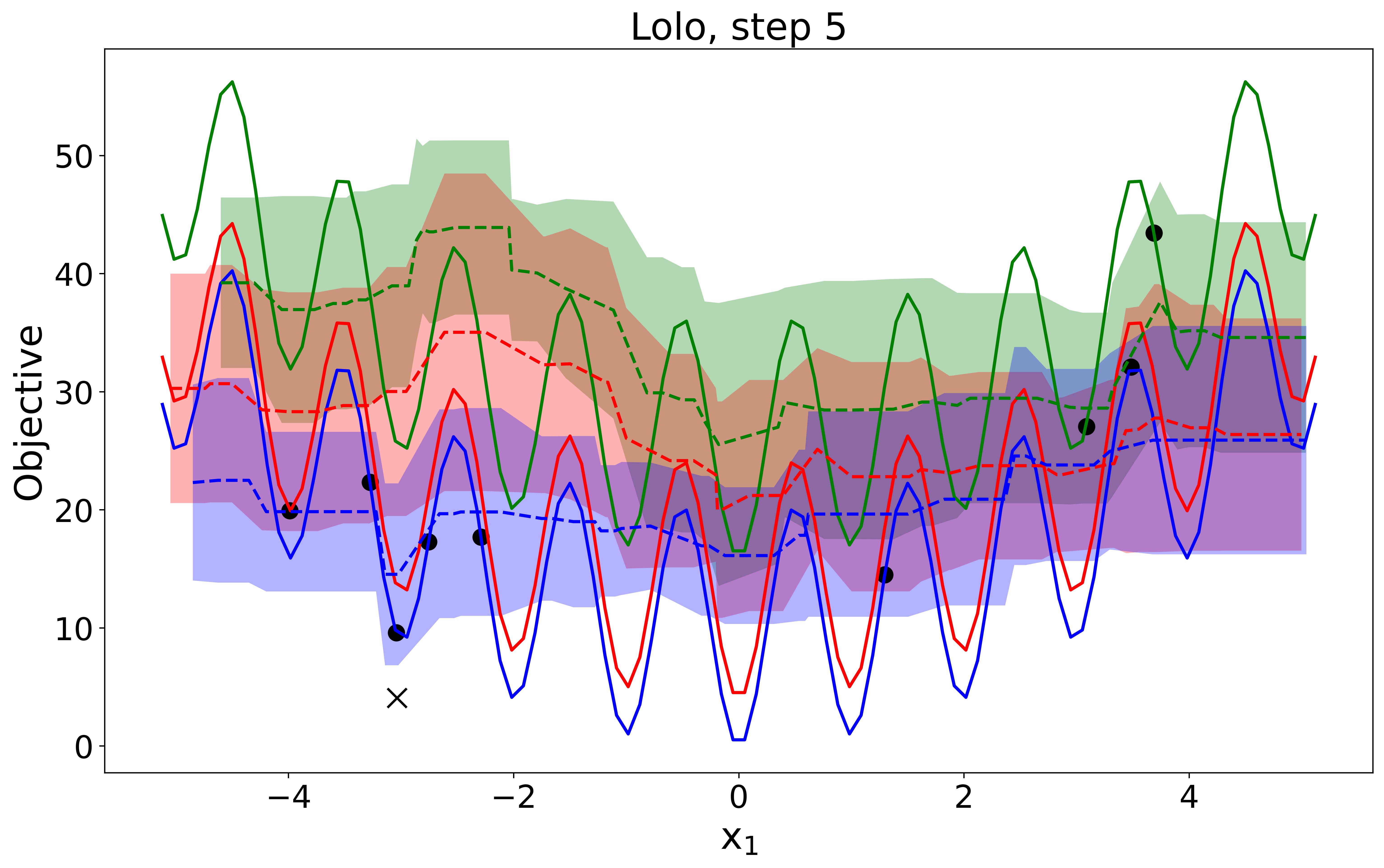

Supplement: Supplementary file 1 — Supplementary Information 1. [file 41598_2022_23431_MOESM1_ESM.zip › Sampling_Sequence_Figures/Rastrigin_Function/rastrigin2_Lolo_5.jpg]

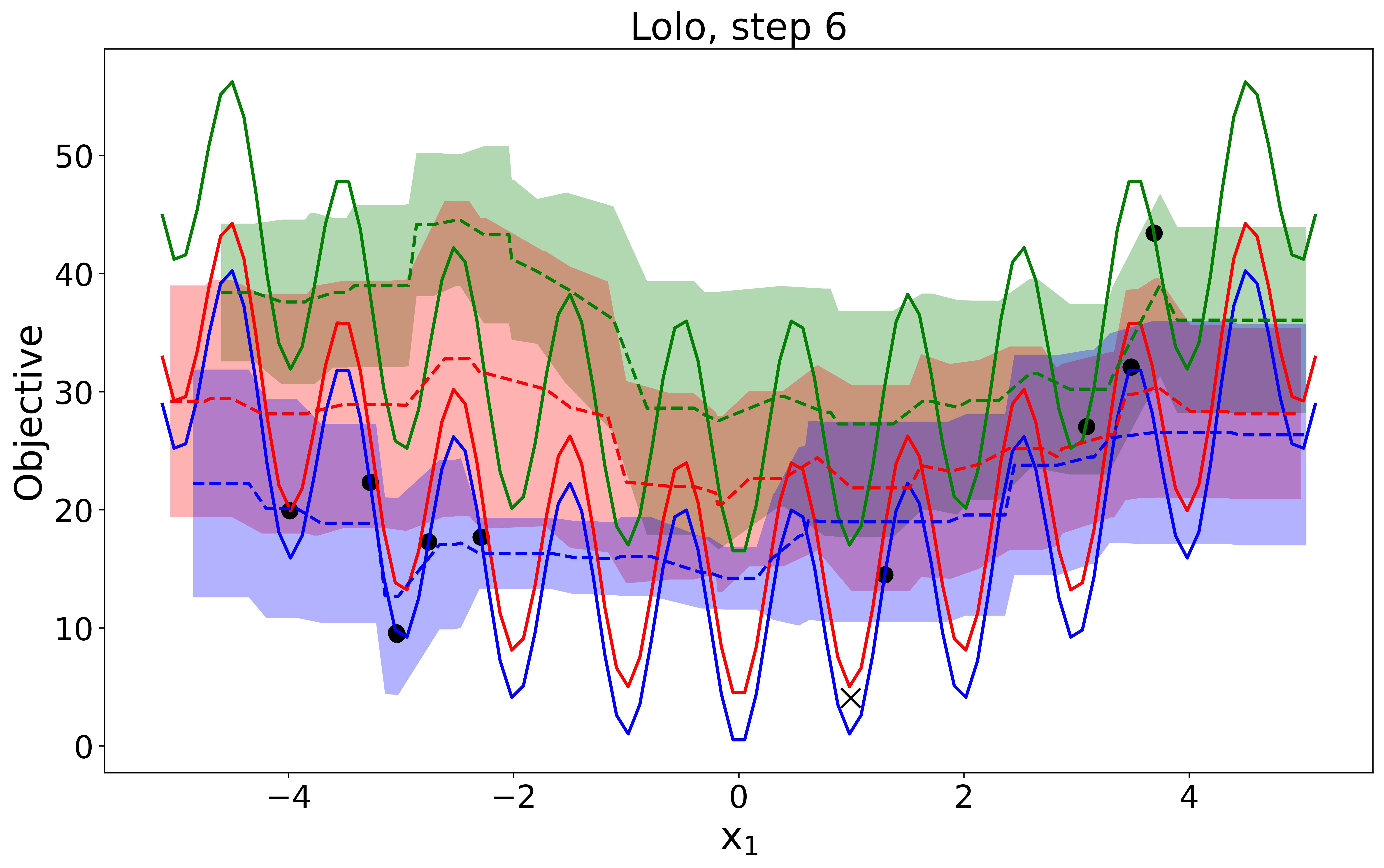

Supplement: Supplementary file 1 — Supplementary Information 1. [file 41598_2022_23431_MOESM1_ESM.zip › Sampling_Sequence_Figures/Rastrigin_Function/rastrigin2_Lolo_6.jpg]

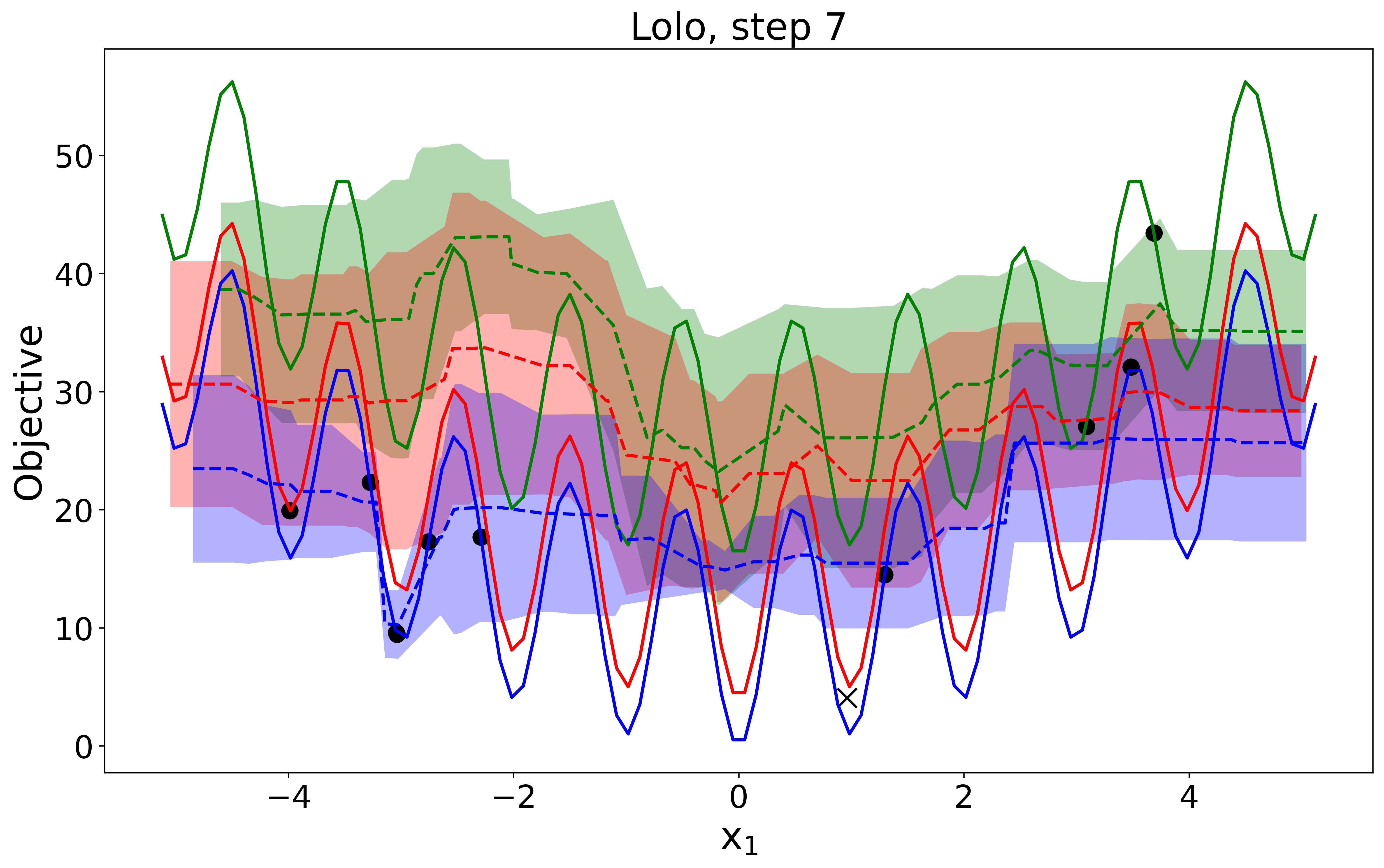

Supplement: Supplementary file 1 — Supplementary Information 1. [file 41598_2022_23431_MOESM1_ESM.zip › Sampling_Sequence_Figures/Rastrigin_Function/rastrigin2_Lolo_7.jpg]

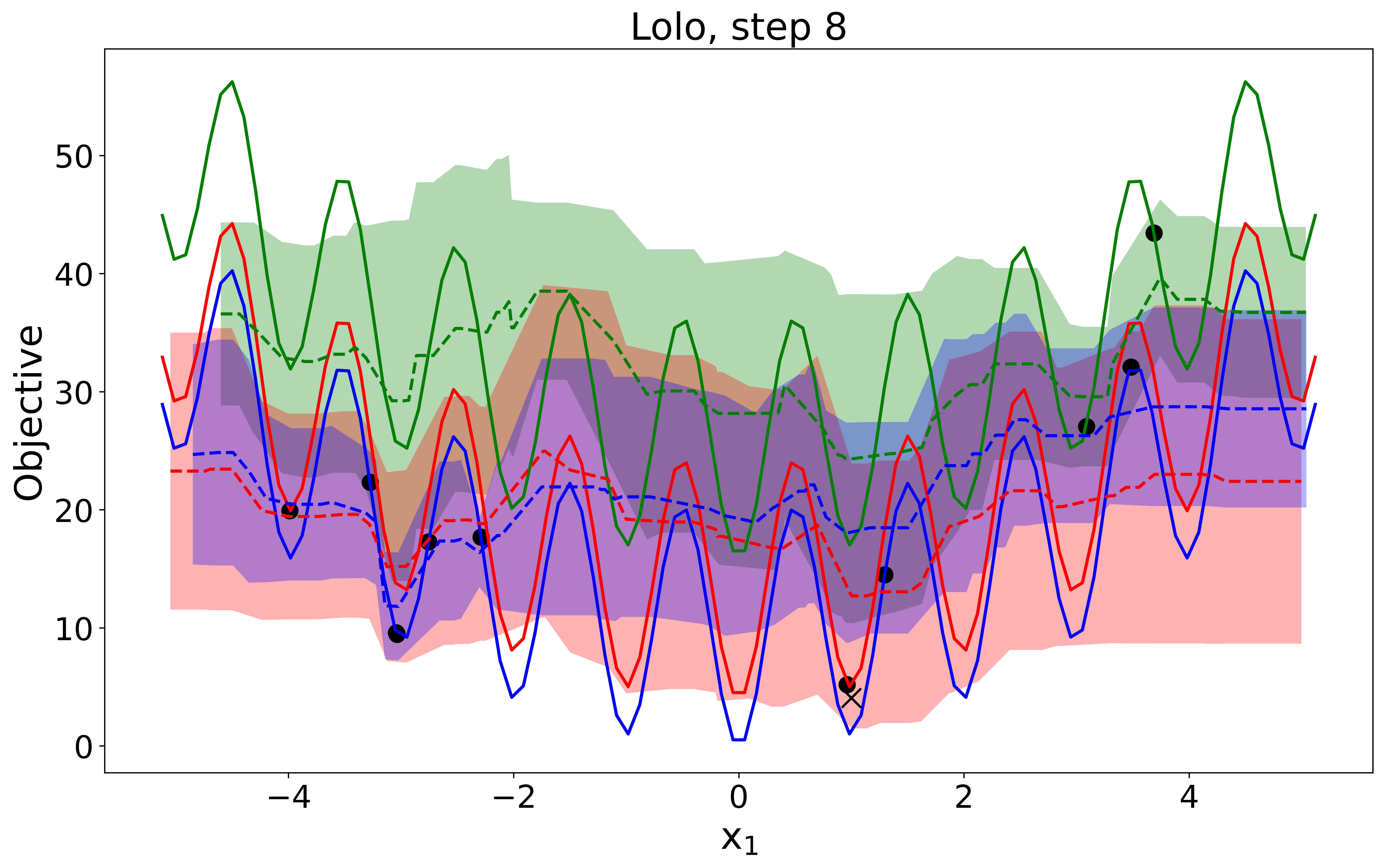

Supplement: Supplementary file 1 — Supplementary Information 1. [file 41598_2022_23431_MOESM1_ESM.zip › Sampling_Sequence_Figures/Rastrigin_Function/rastrigin2_Lolo_8.jpg]

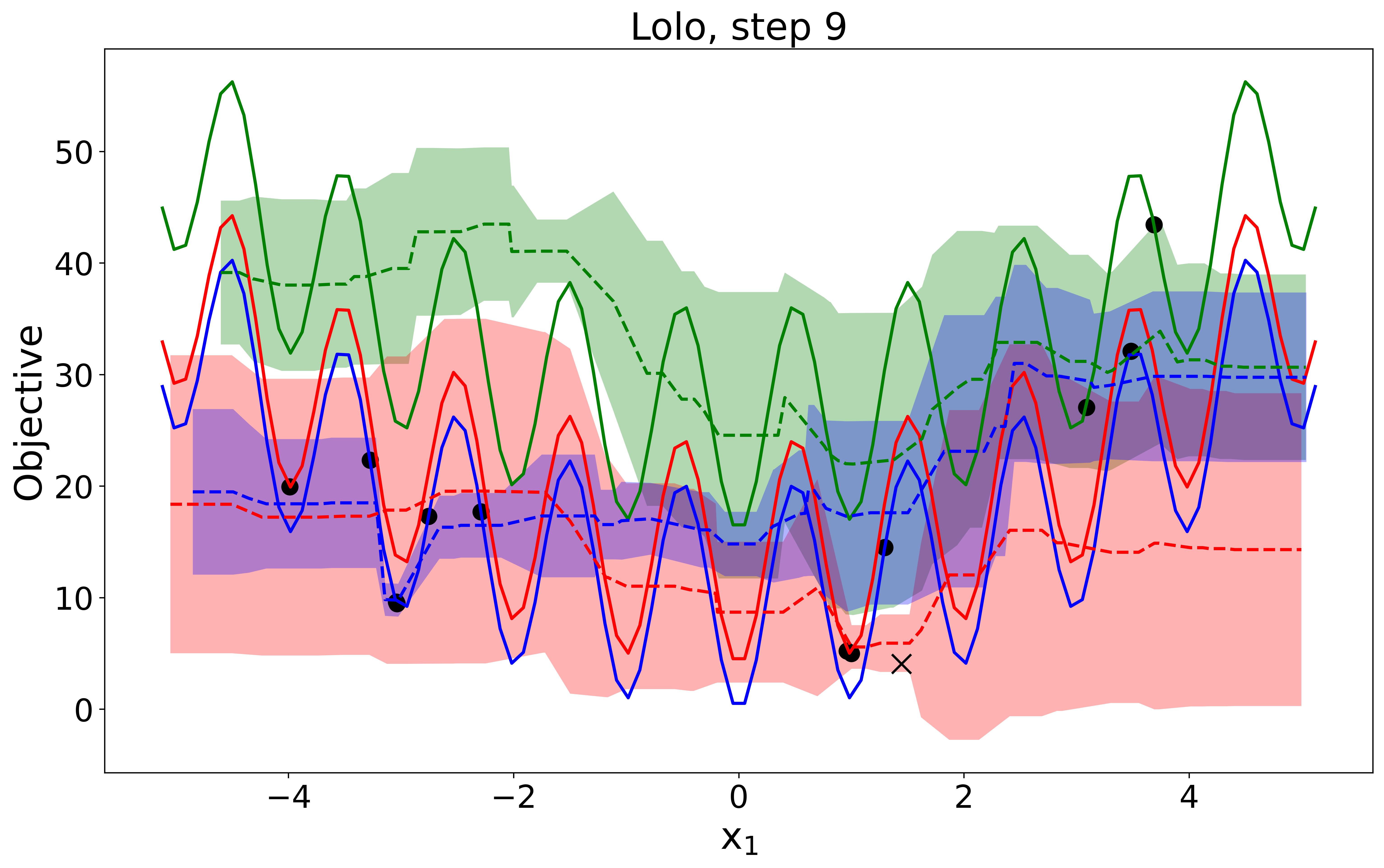

Supplement: Supplementary file 1 — Supplementary Information 1. [file 41598_2022_23431_MOESM1_ESM.zip › Sampling_Sequence_Figures/Rastrigin_Function/rastrigin2_Lolo_9.jpg]

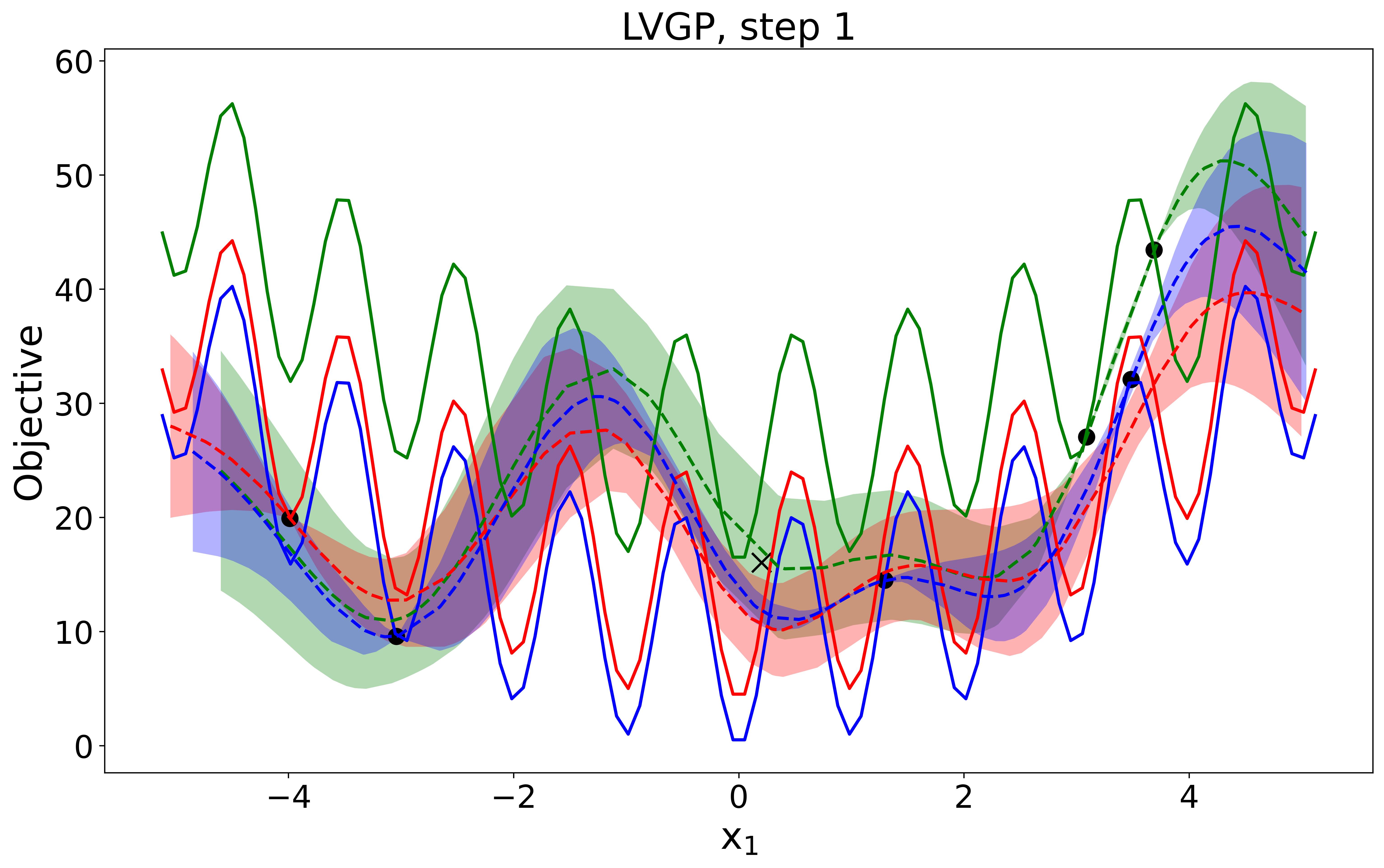

Supplement: Supplementary file 1 — Supplementary Information 1. [file 41598_2022_23431_MOESM1_ESM.zip › Sampling_Sequence_Figures/Rastrigin_Function/rastrigin2_LVGP_1.jpg]

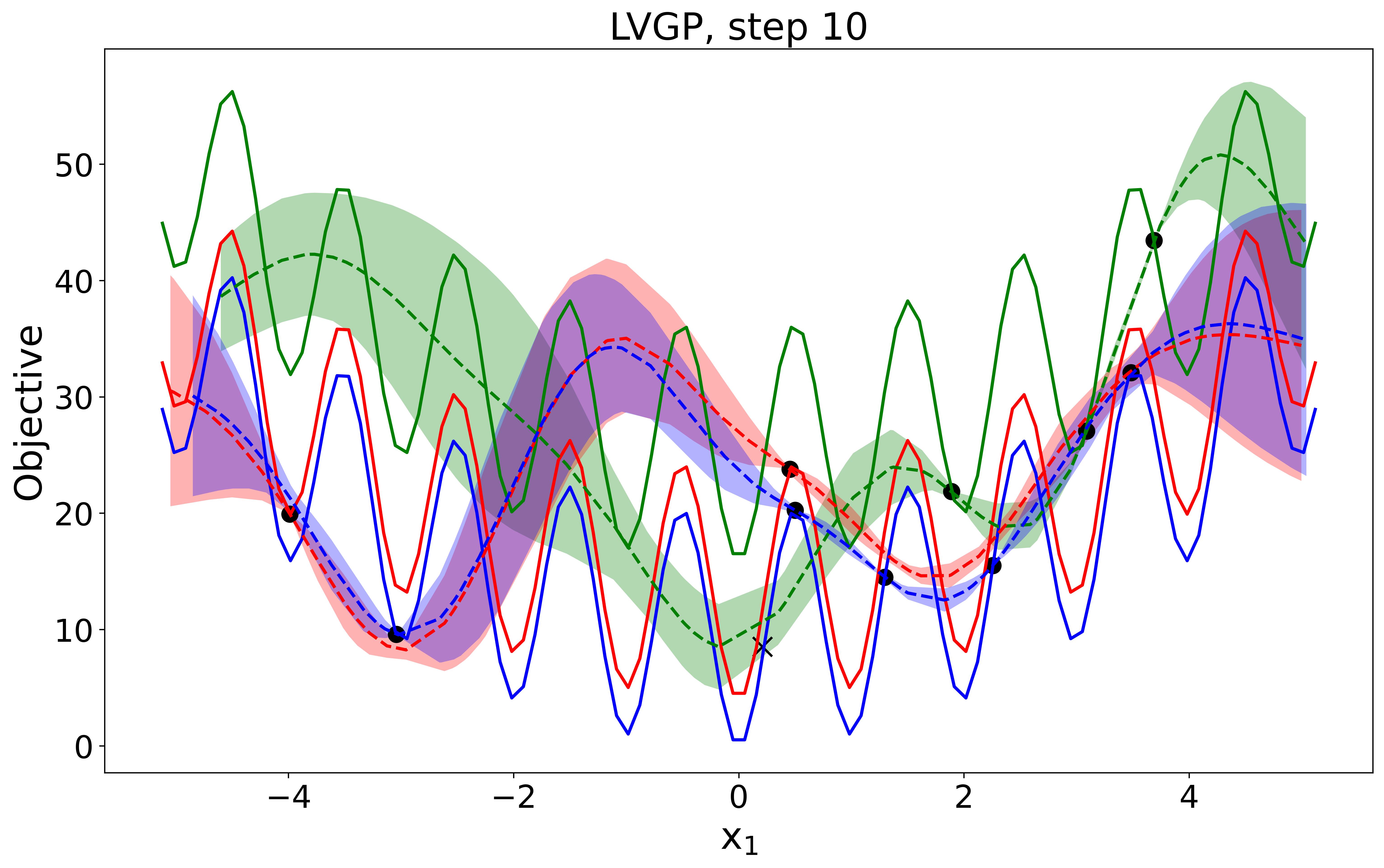

Supplement: Supplementary file 1 — Supplementary Information 1. [file 41598_2022_23431_MOESM1_ESM.zip › Sampling_Sequence_Figures/Rastrigin_Function/rastrigin2_LVGP_10.jpg]

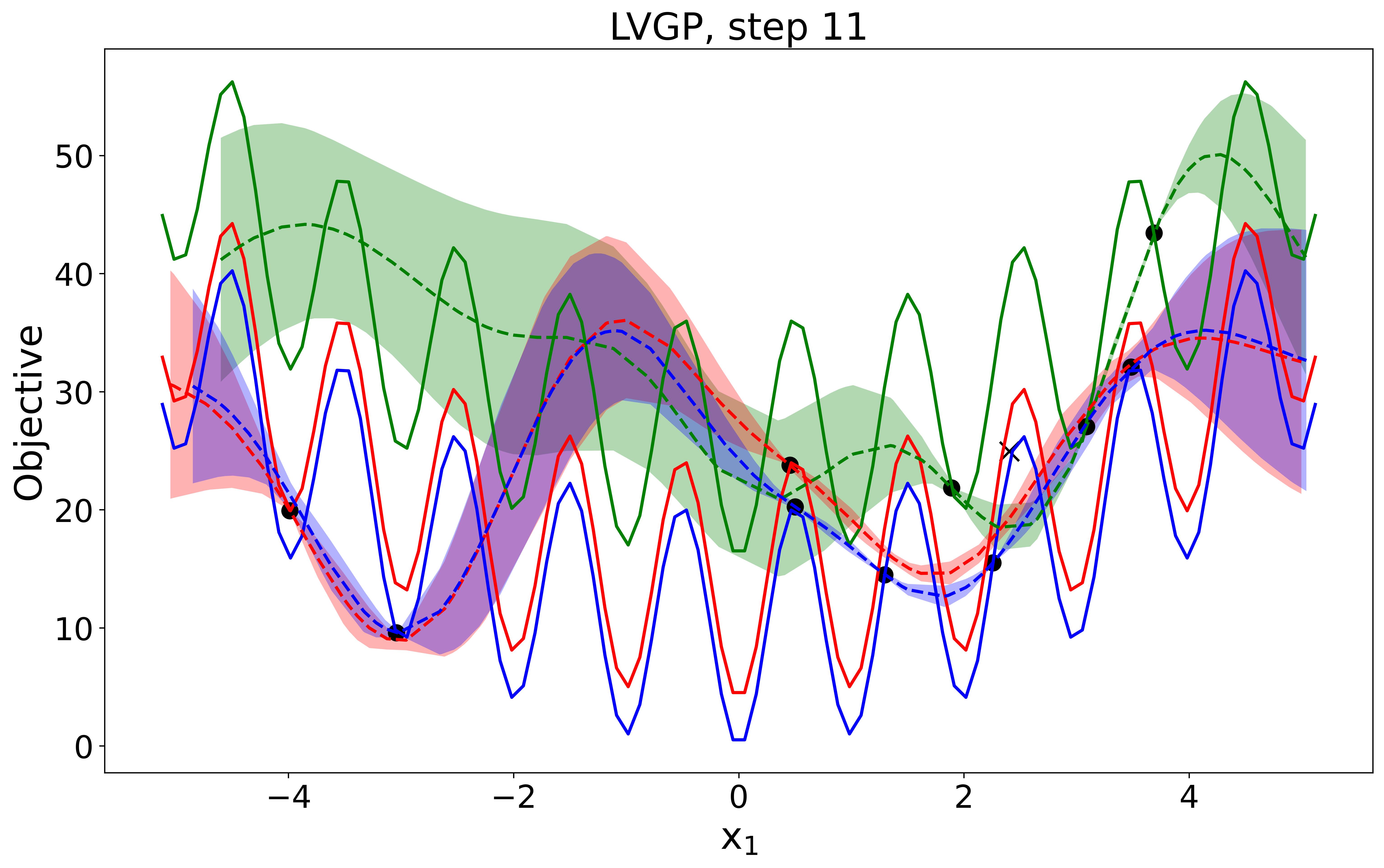

Supplement: Supplementary file 1 — Supplementary Information 1. [file 41598_2022_23431_MOESM1_ESM.zip › Sampling_Sequence_Figures/Rastrigin_Function/rastrigin2_LVGP_11.jpg]

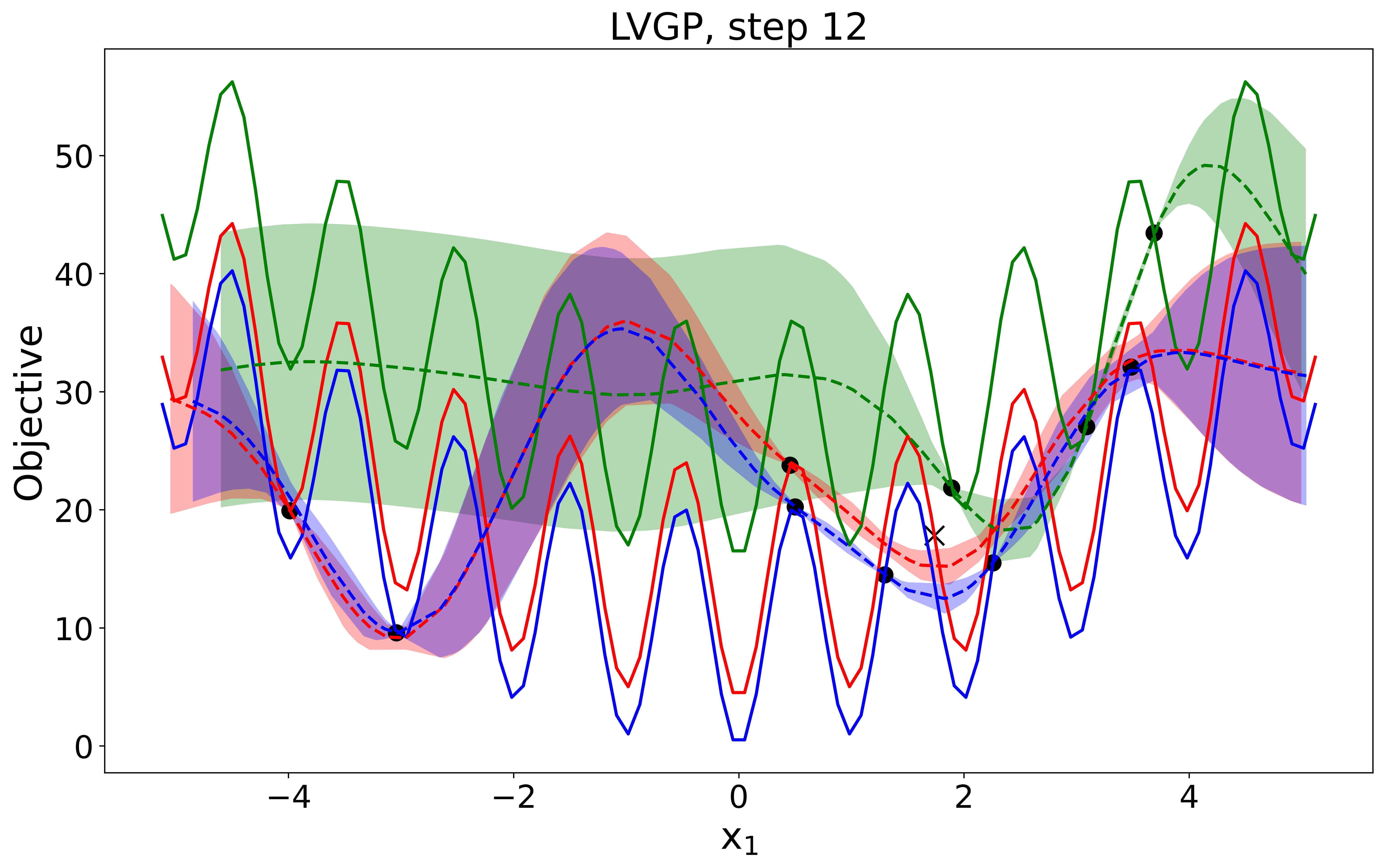

Supplement: Supplementary file 1 — Supplementary Information 1. [file 41598_2022_23431_MOESM1_ESM.zip › Sampling_Sequence_Figures/Rastrigin_Function/rastrigin2_LVGP_12.jpg]

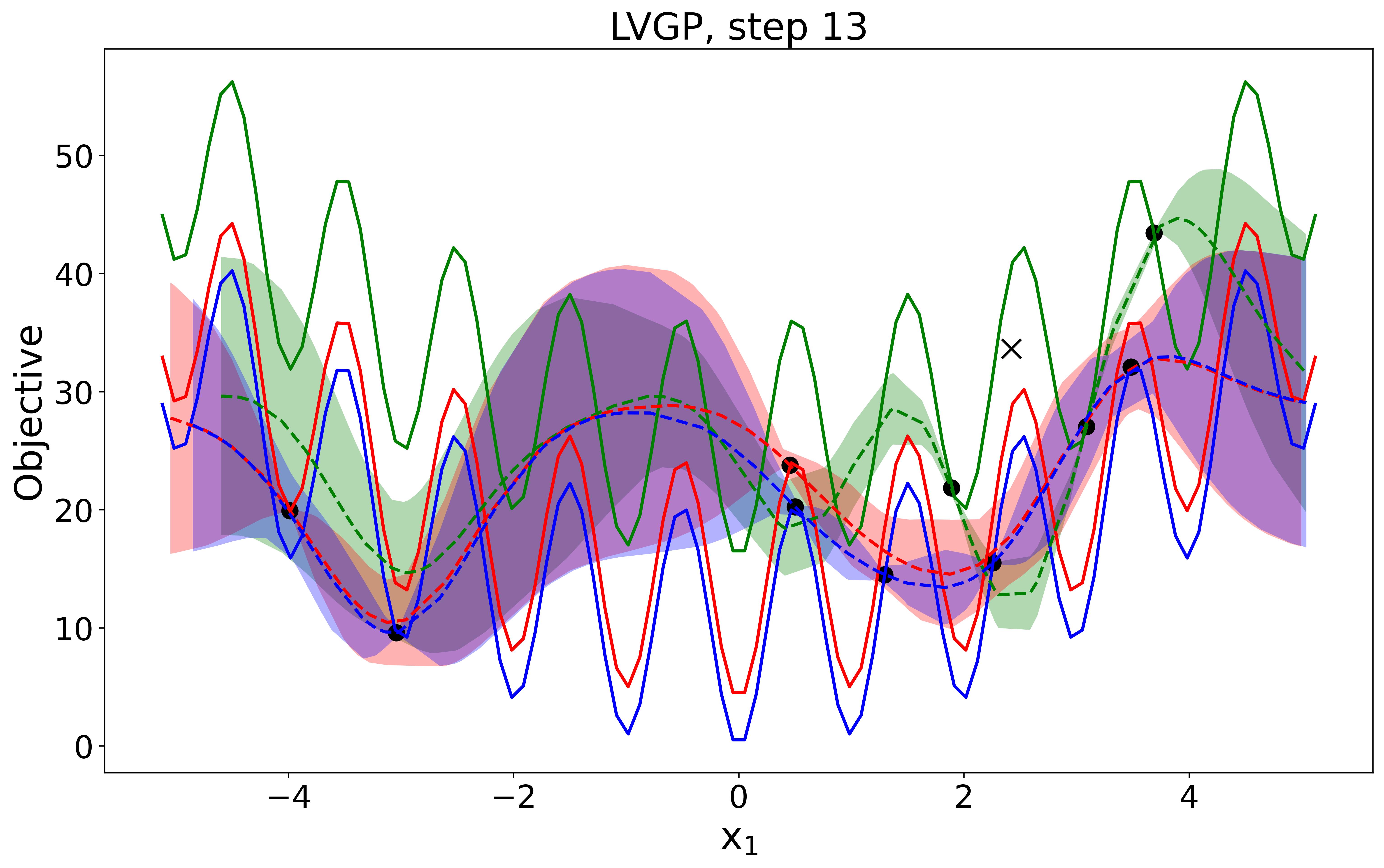

Supplement: Supplementary file 1 — Supplementary Information 1. [file 41598_2022_23431_MOESM1_ESM.zip › Sampling_Sequence_Figures/Rastrigin_Function/rastrigin2_LVGP_13.jpg]

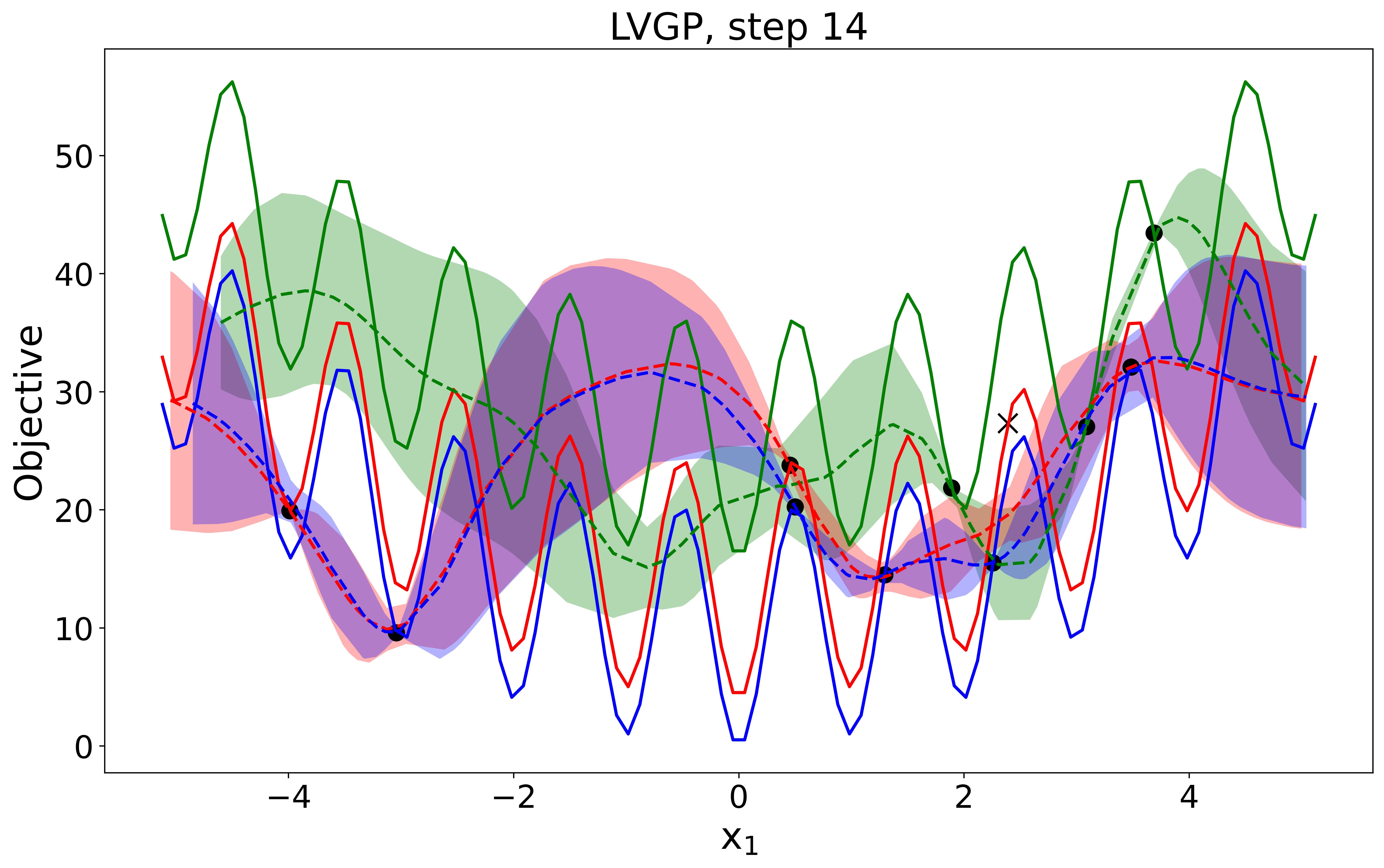

Supplement: Supplementary file 1 — Supplementary Information 1. [file 41598_2022_23431_MOESM1_ESM.zip › Sampling_Sequence_Figures/Rastrigin_Function/rastrigin2_LVGP_14.jpg]

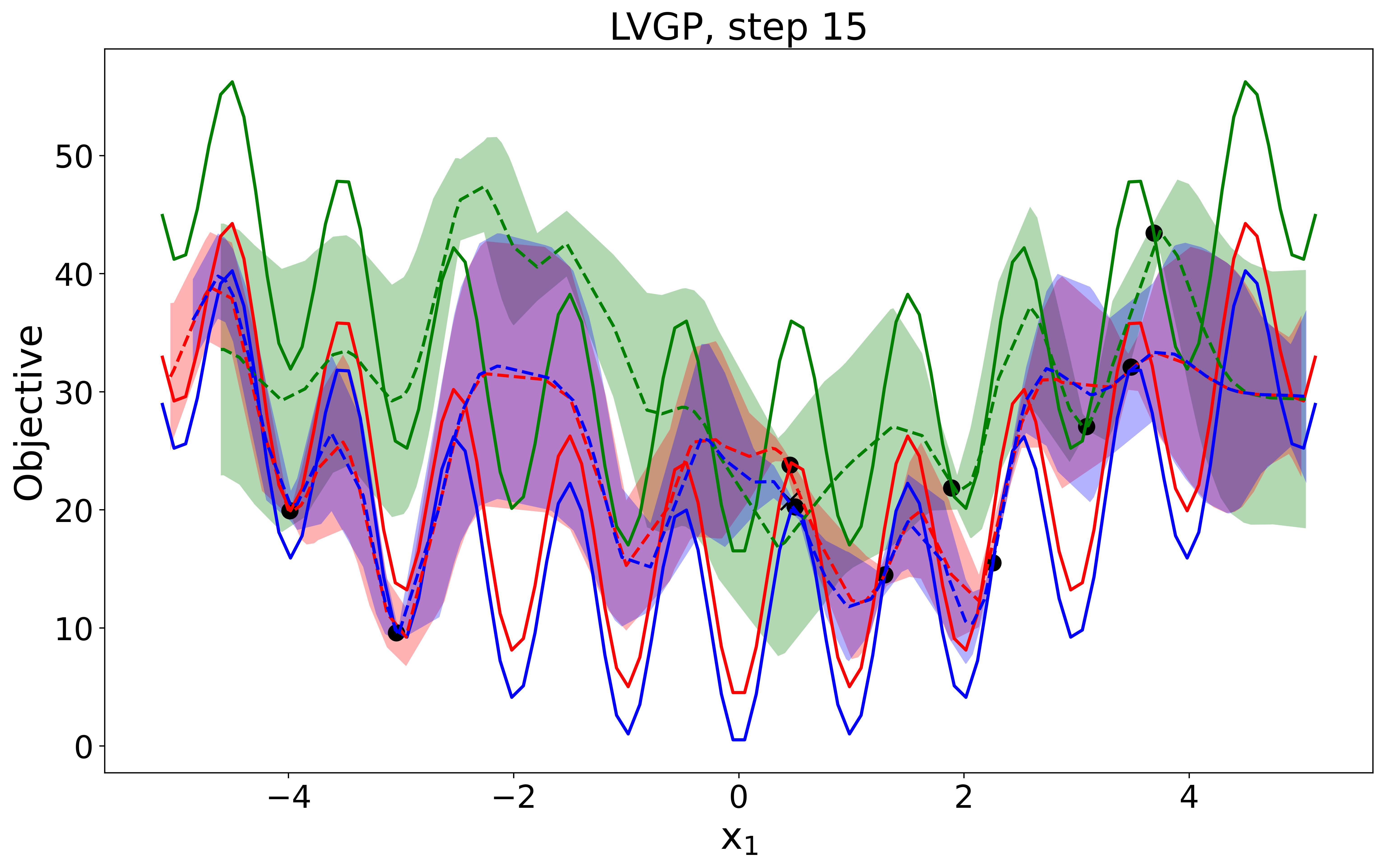

Supplement: Supplementary file 1 — Supplementary Information 1. [file 41598_2022_23431_MOESM1_ESM.zip › Sampling_Sequence_Figures/Rastrigin_Function/rastrigin2_LVGP_15.jpg]

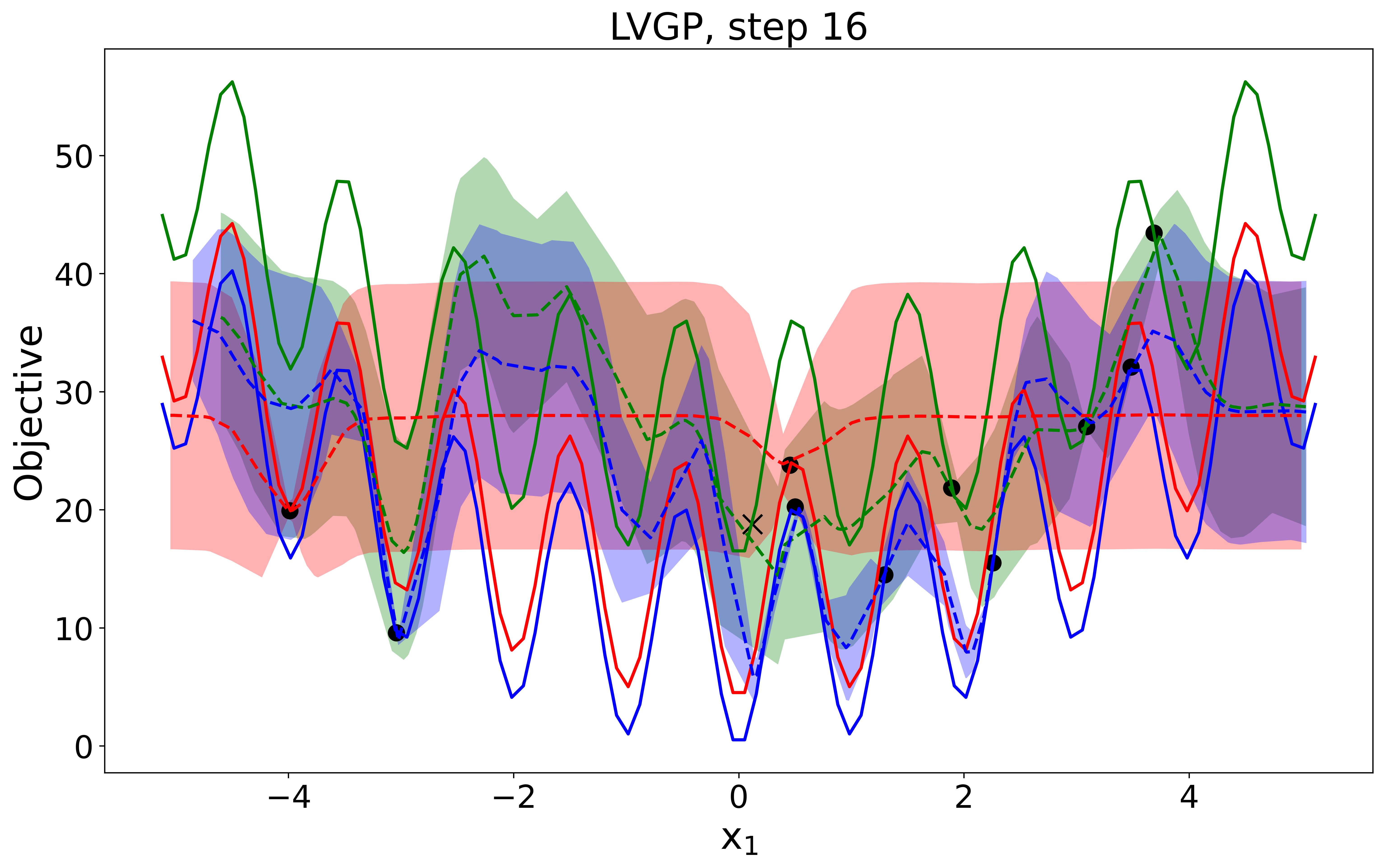

Supplement: Supplementary file 1 — Supplementary Information 1. [file 41598_2022_23431_MOESM1_ESM.zip › Sampling_Sequence_Figures/Rastrigin_Function/rastrigin2_LVGP_16.jpg]

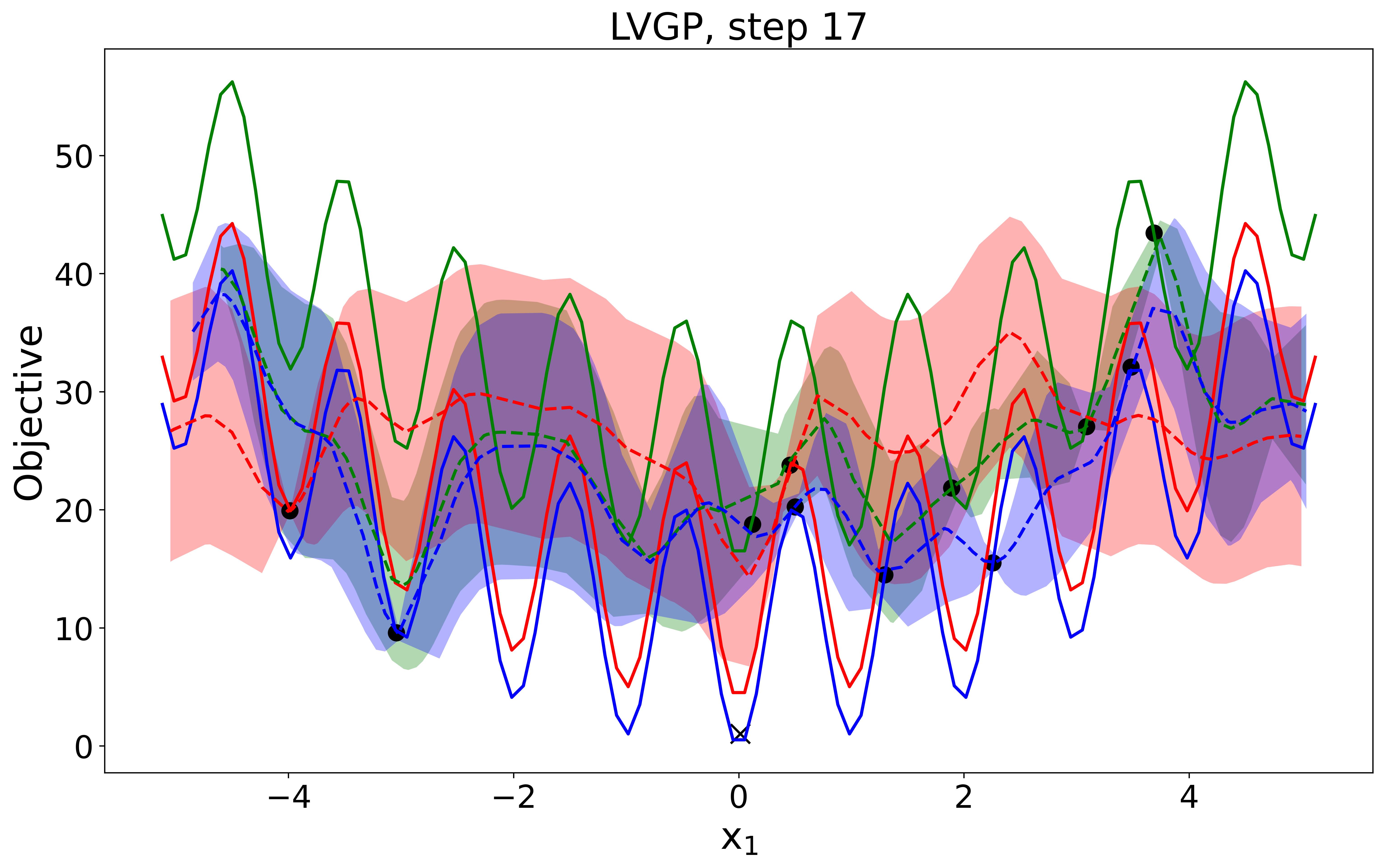

Supplement: Supplementary file 1 — Supplementary Information 1. [file 41598_2022_23431_MOESM1_ESM.zip › Sampling_Sequence_Figures/Rastrigin_Function/rastrigin2_LVGP_17.jpg]

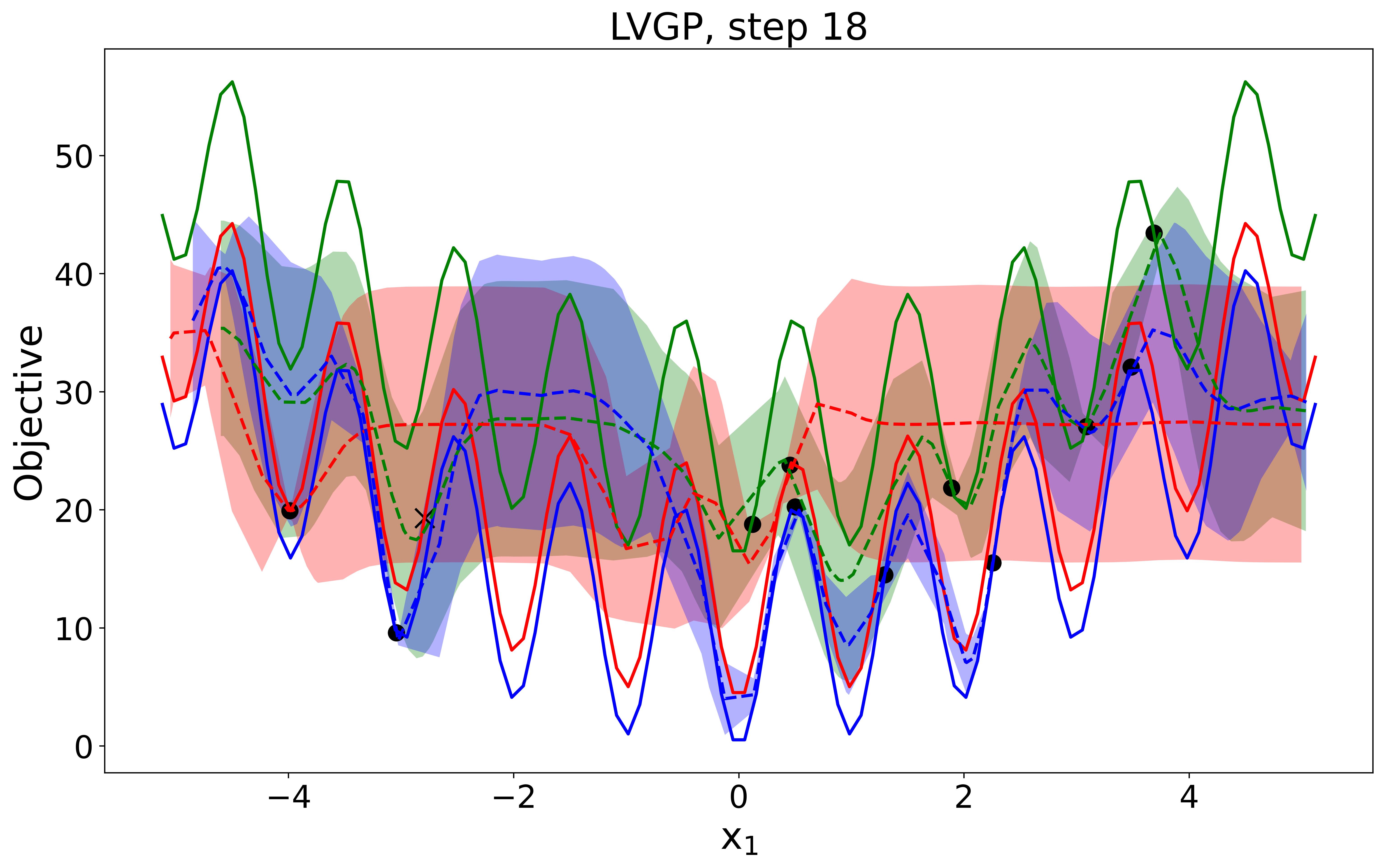

Supplement: Supplementary file 1 — Supplementary Information 1. [file 41598_2022_23431_MOESM1_ESM.zip › Sampling_Sequence_Figures/Rastrigin_Function/rastrigin2_LVGP_18.jpg]

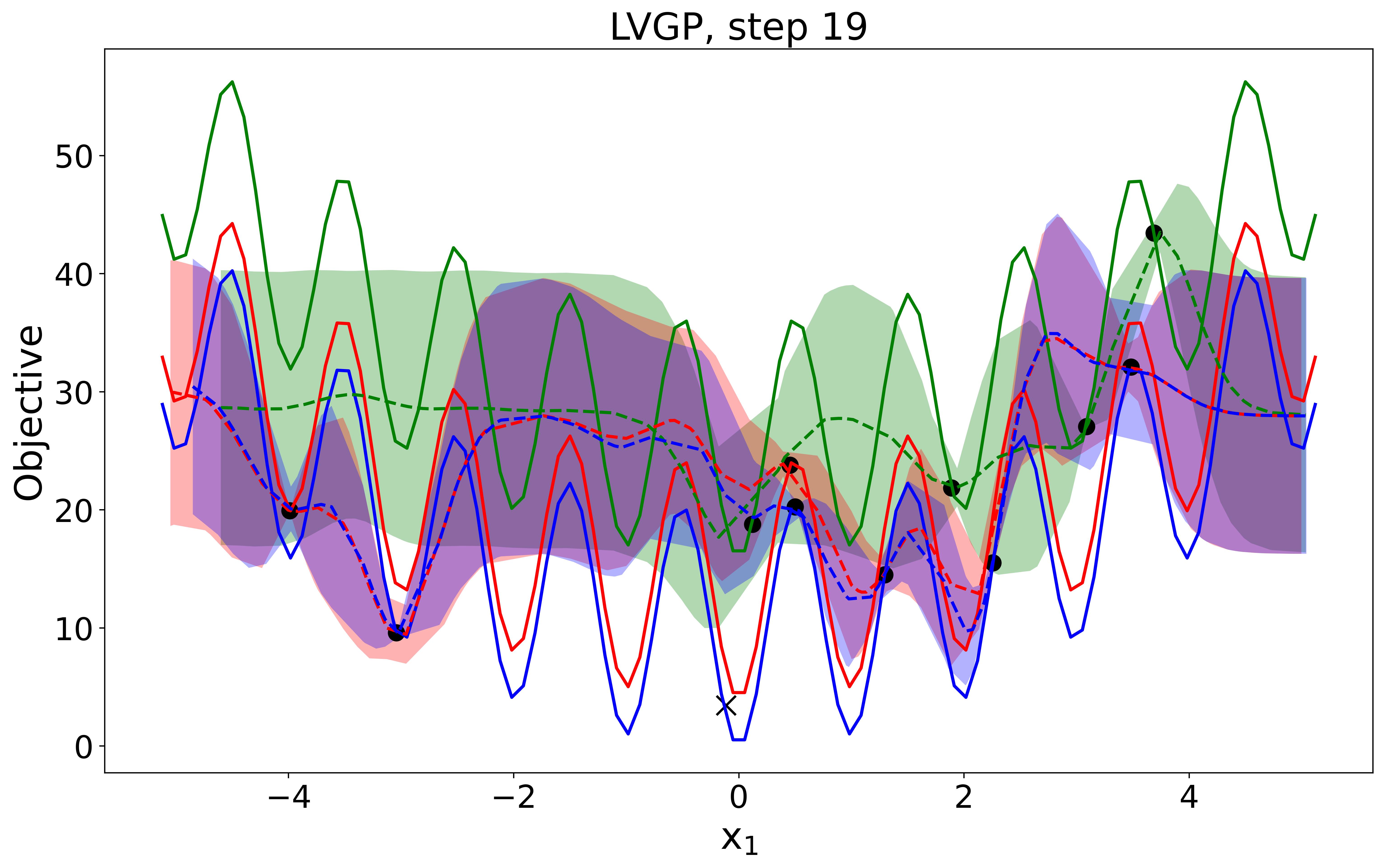

Supplement: Supplementary file 1 — Supplementary Information 1. [file 41598_2022_23431_MOESM1_ESM.zip › Sampling_Sequence_Figures/Rastrigin_Function/rastrigin2_LVGP_19.jpg]

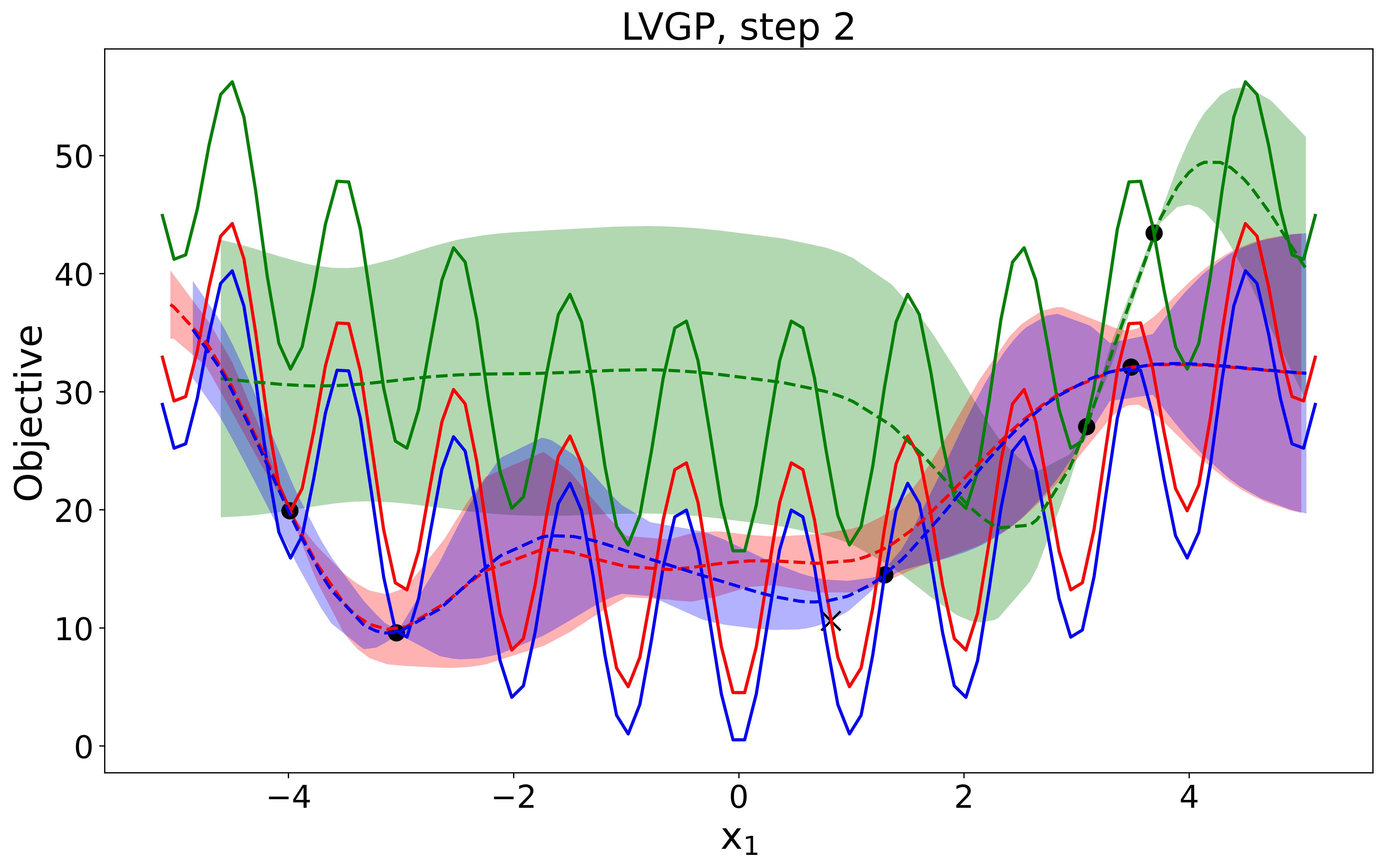

Supplement: Supplementary file 1 — Supplementary Information 1. [file 41598_2022_23431_MOESM1_ESM.zip › Sampling_Sequence_Figures/Rastrigin_Function/rastrigin2_LVGP_2.jpg]

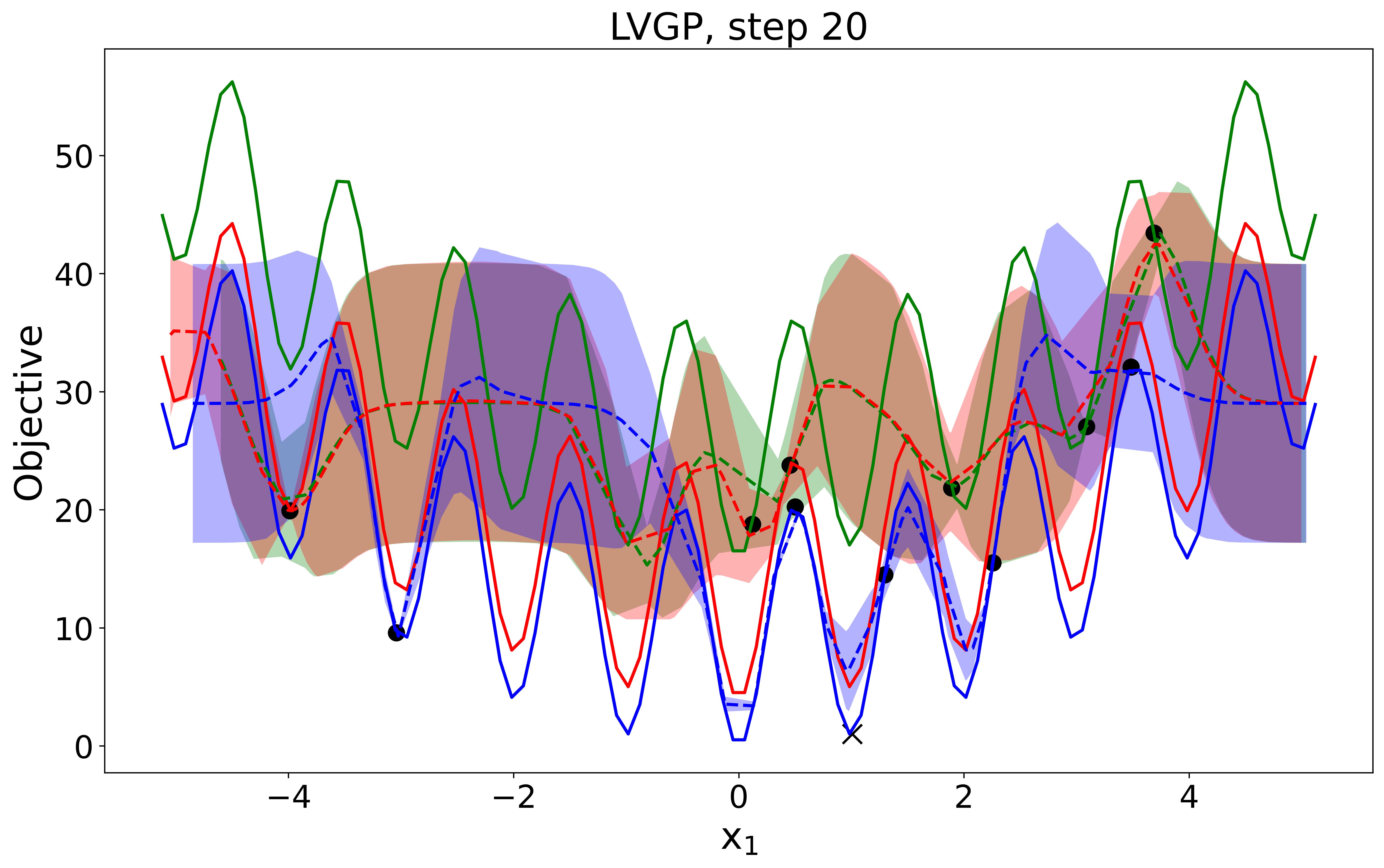

Supplement: Supplementary file 1 — Supplementary Information 1. [file 41598_2022_23431_MOESM1_ESM.zip › Sampling_Sequence_Figures/Rastrigin_Function/rastrigin2_LVGP_20.jpg]

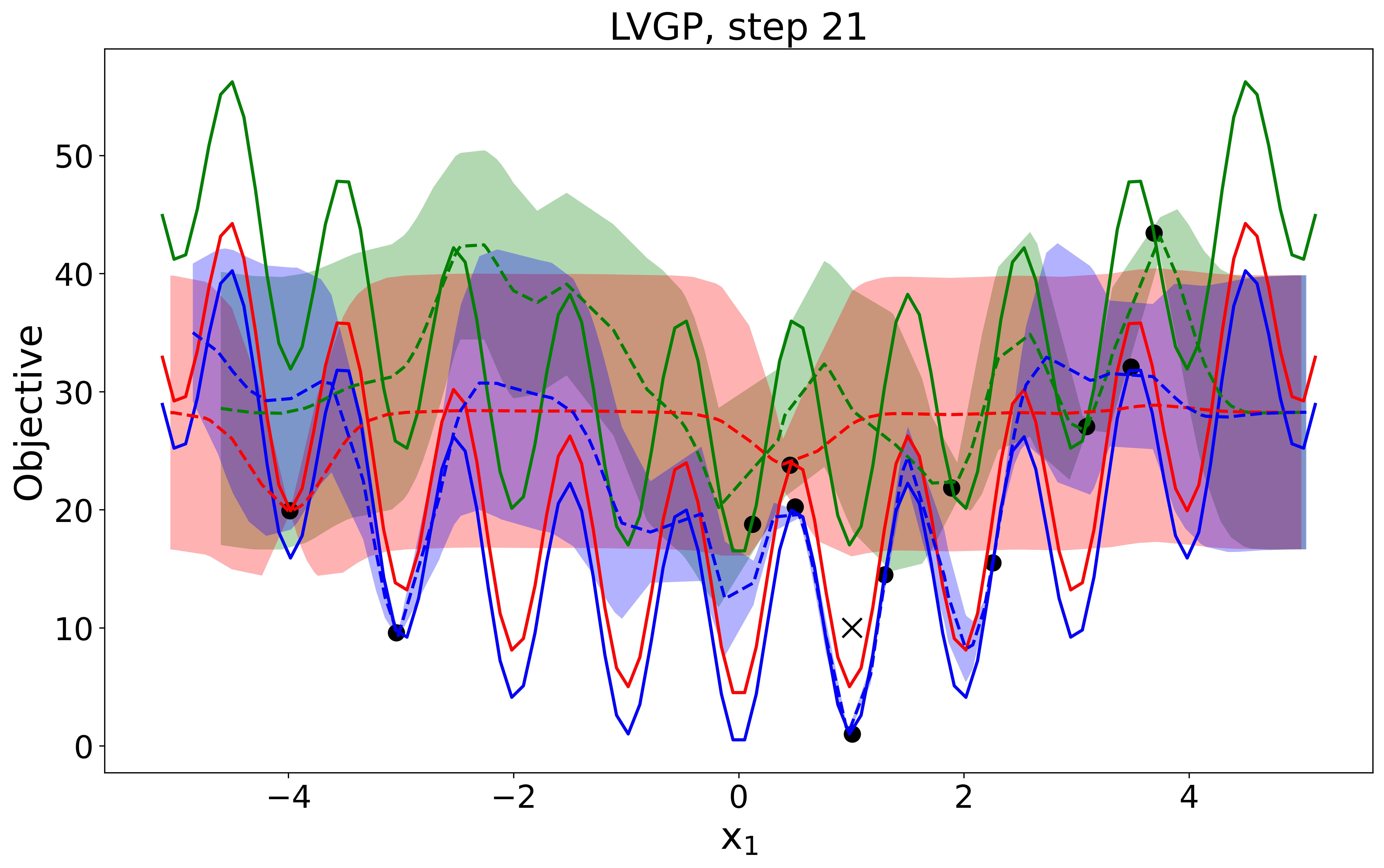

Supplement: Supplementary file 1 — Supplementary Information 1. [file 41598_2022_23431_MOESM1_ESM.zip › Sampling_Sequence_Figures/Rastrigin_Function/rastrigin2_LVGP_21.jpg]

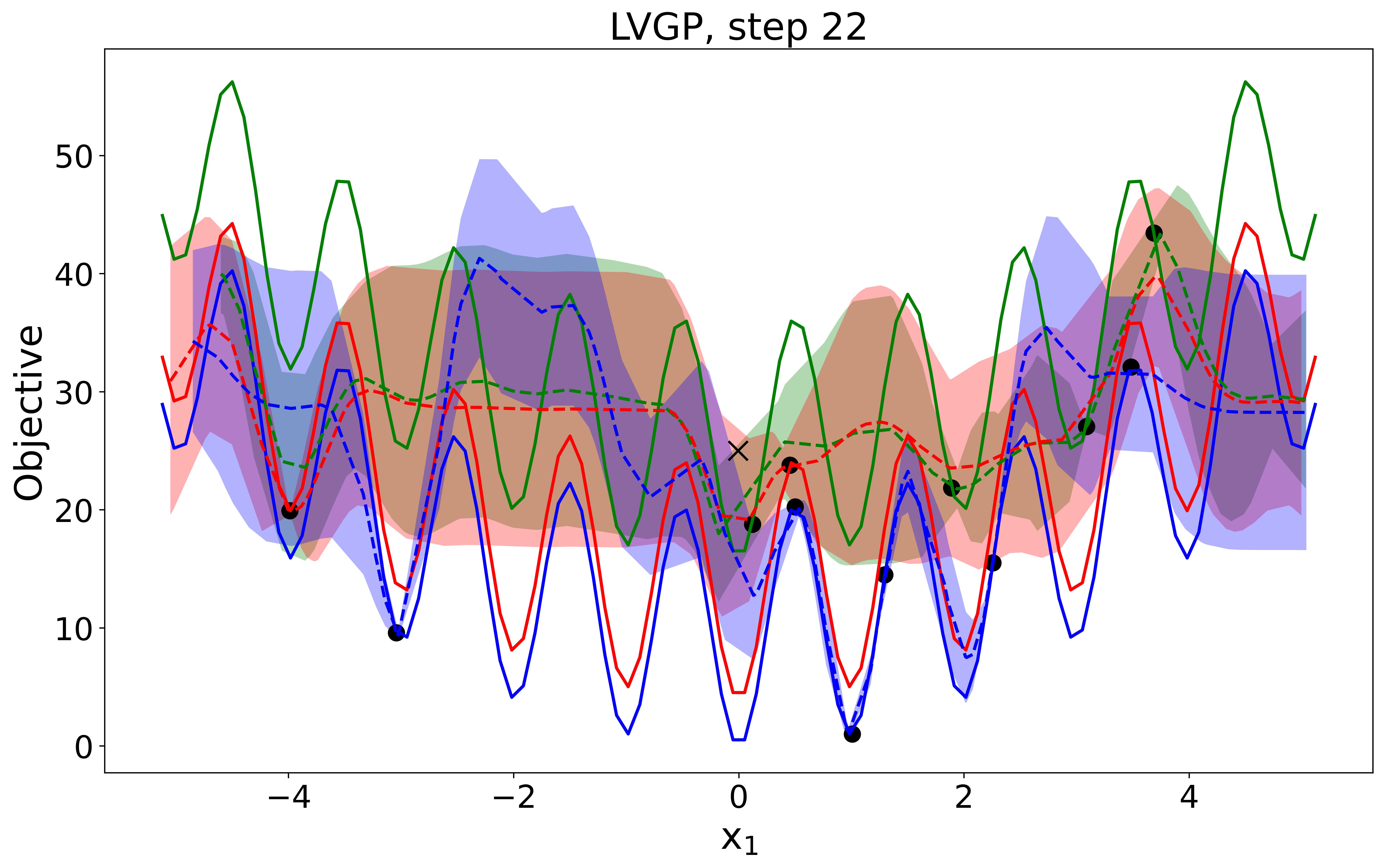

Supplement: Supplementary file 1 — Supplementary Information 1. [file 41598_2022_23431_MOESM1_ESM.zip › Sampling_Sequence_Figures/Rastrigin_Function/rastrigin2_LVGP_22.jpg]

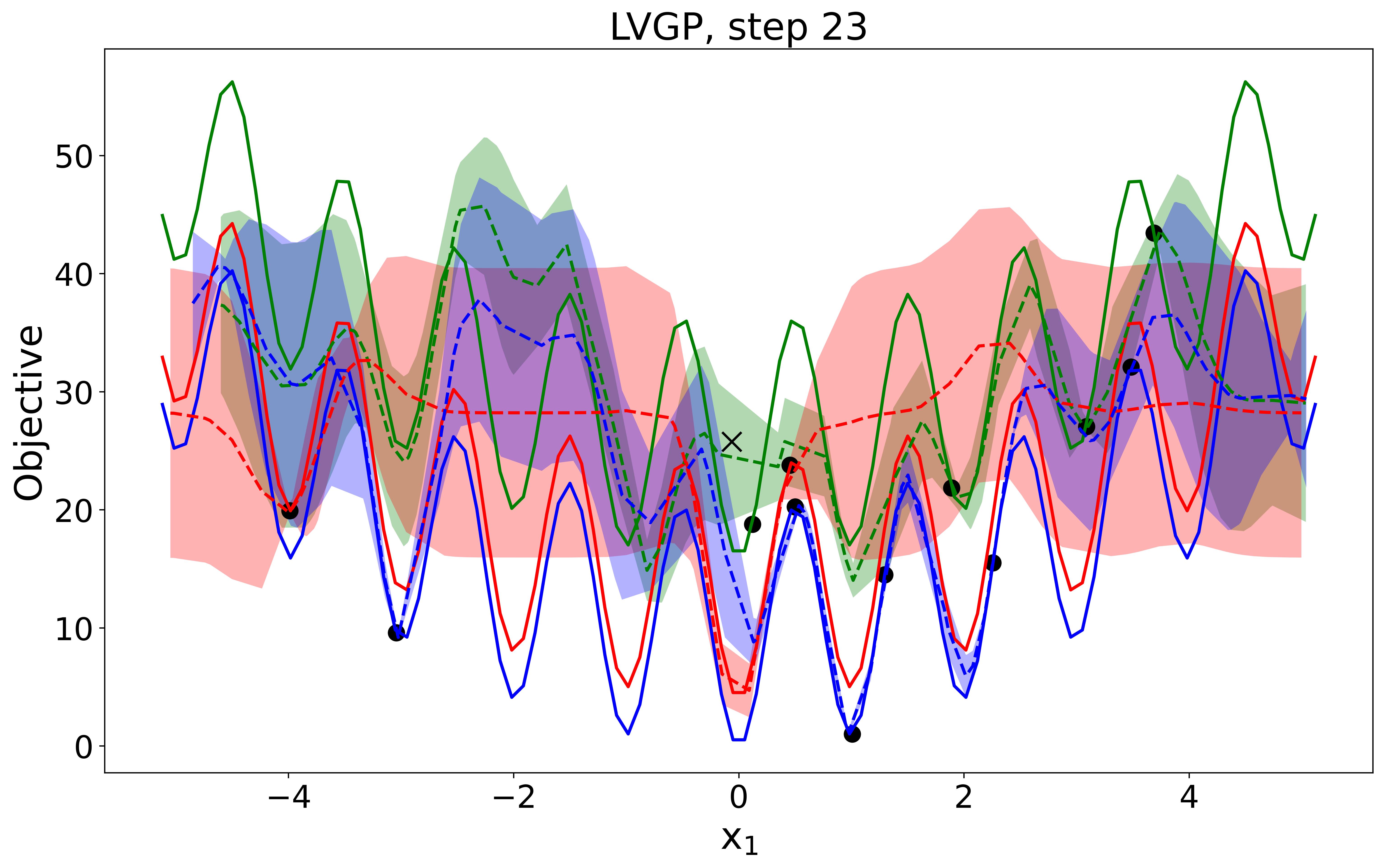

Supplement: Supplementary file 1 — Supplementary Information 1. [file 41598_2022_23431_MOESM1_ESM.zip › Sampling_Sequence_Figures/Rastrigin_Function/rastrigin2_LVGP_23.jpg]

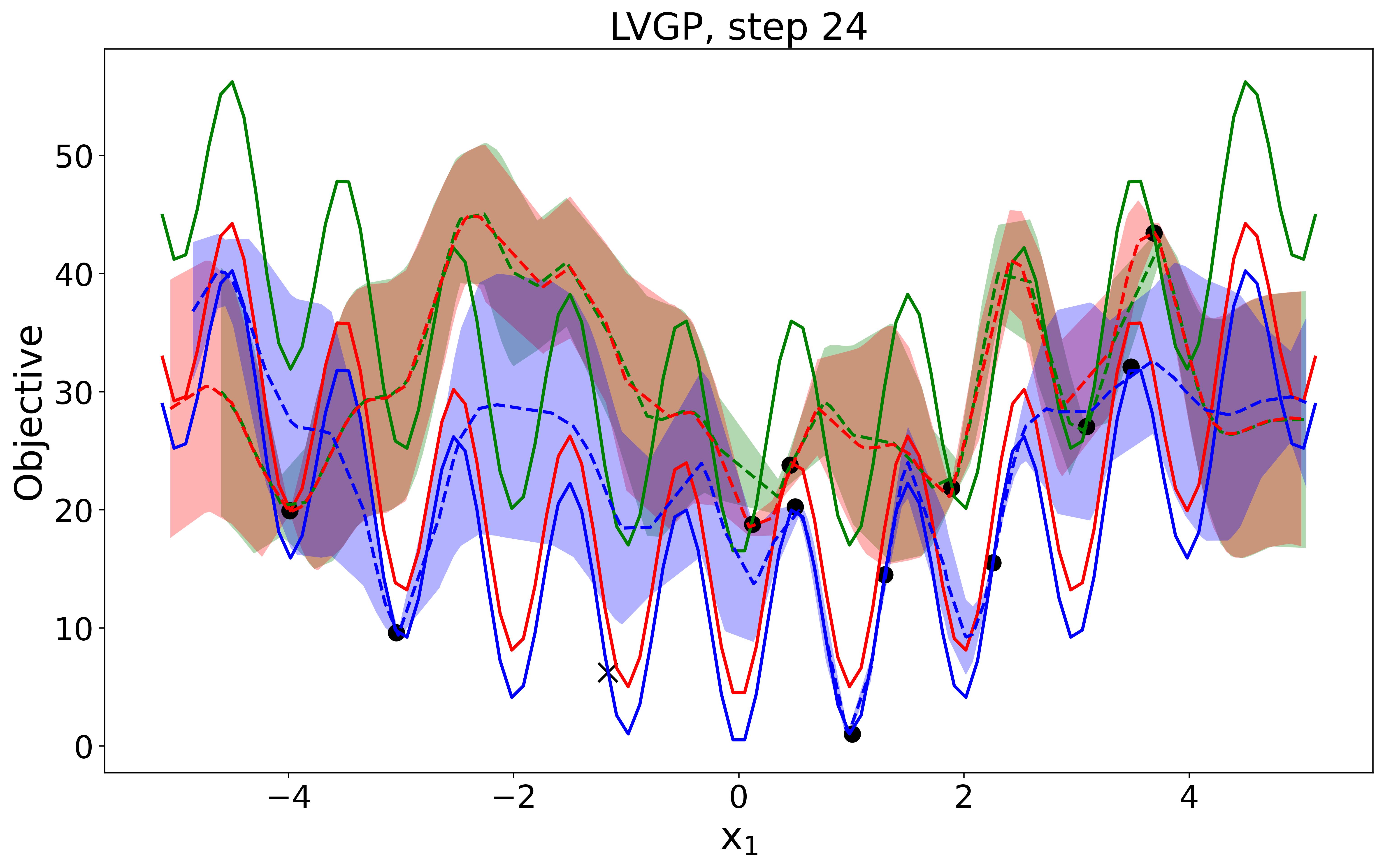

Supplement: Supplementary file 1 — Supplementary Information 1. [file 41598_2022_23431_MOESM1_ESM.zip › Sampling_Sequence_Figures/Rastrigin_Function/rastrigin2_LVGP_24.jpg]

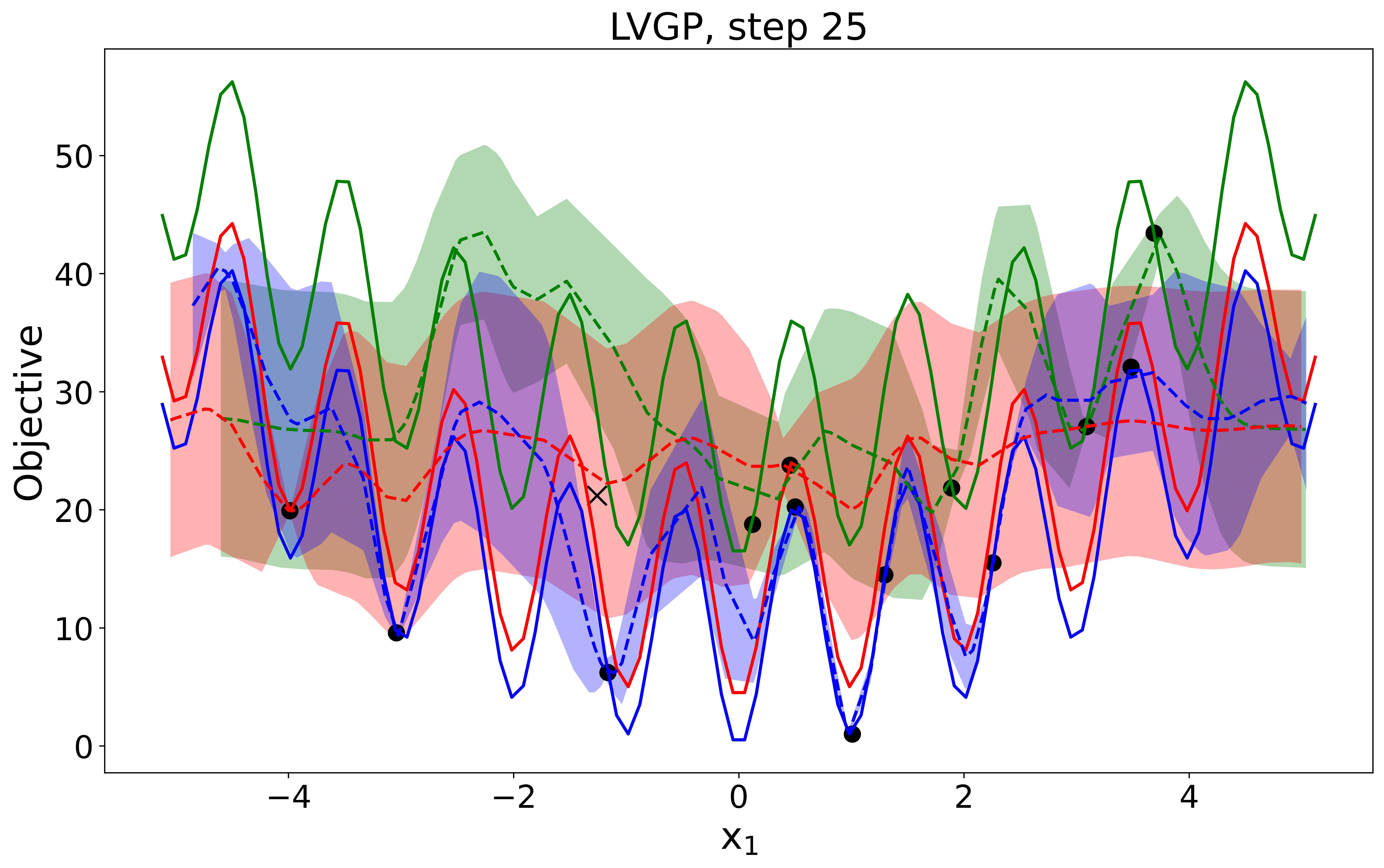

Supplement: Supplementary file 1 — Supplementary Information 1. [file 41598_2022_23431_MOESM1_ESM.zip › Sampling_Sequence_Figures/Rastrigin_Function/rastrigin2_LVGP_25.jpg]

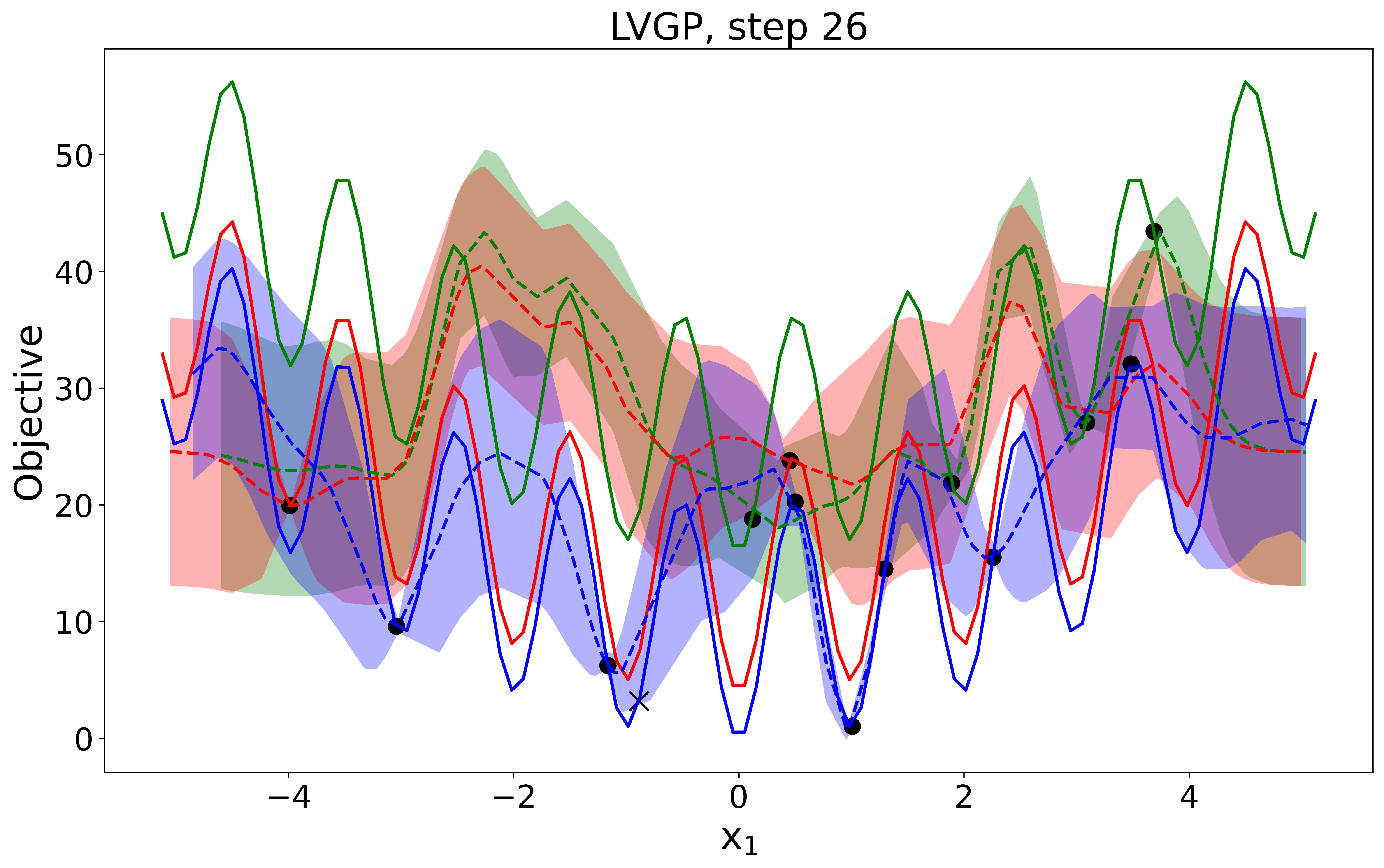

Supplement: Supplementary file 1 — Supplementary Information 1. [file 41598_2022_23431_MOESM1_ESM.zip › Sampling_Sequence_Figures/Rastrigin_Function/rastrigin2_LVGP_26.jpg]

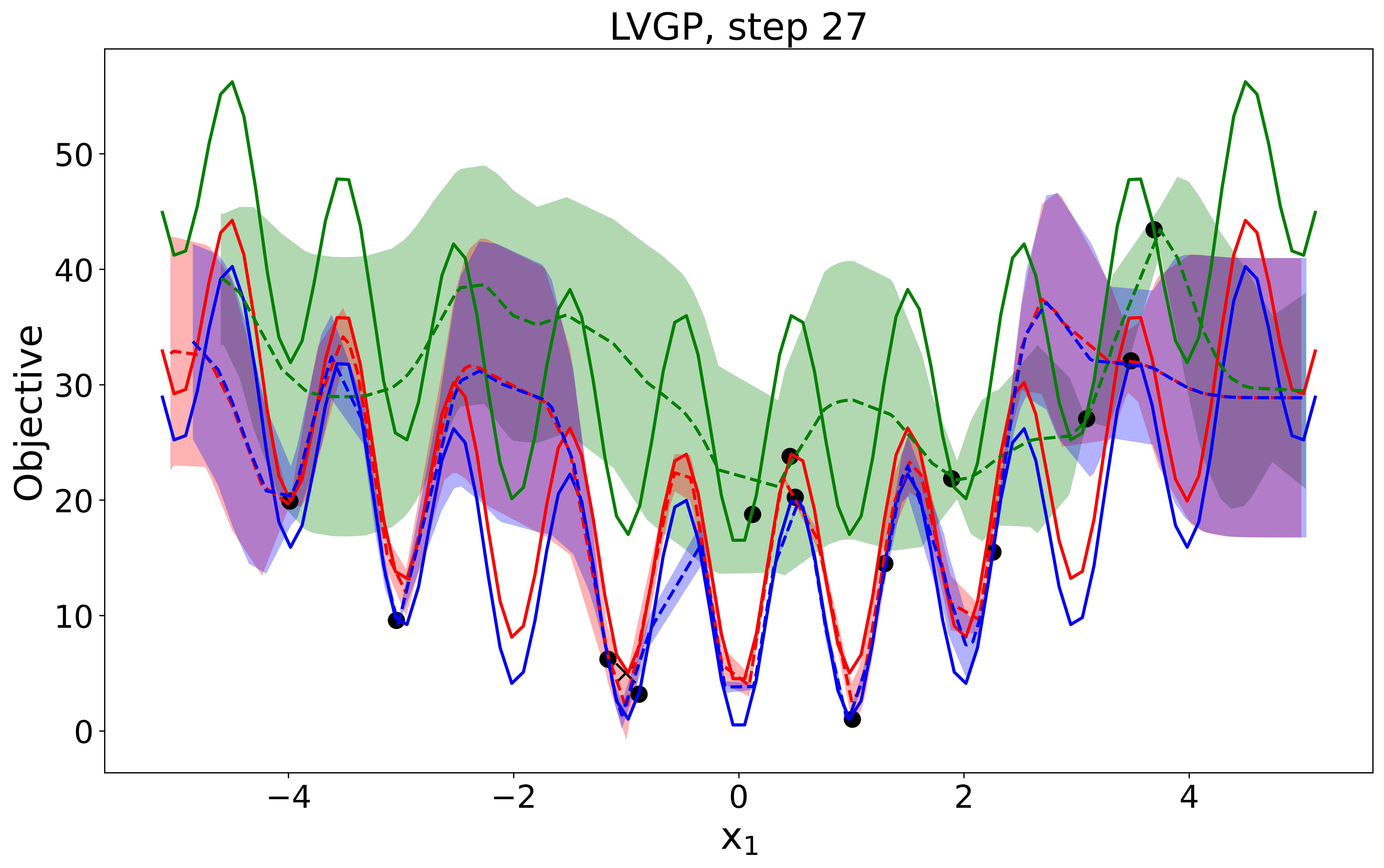

Supplement: Supplementary file 1 — Supplementary Information 1. [file 41598_2022_23431_MOESM1_ESM.zip › Sampling_Sequence_Figures/Rastrigin_Function/rastrigin2_LVGP_27.jpg]

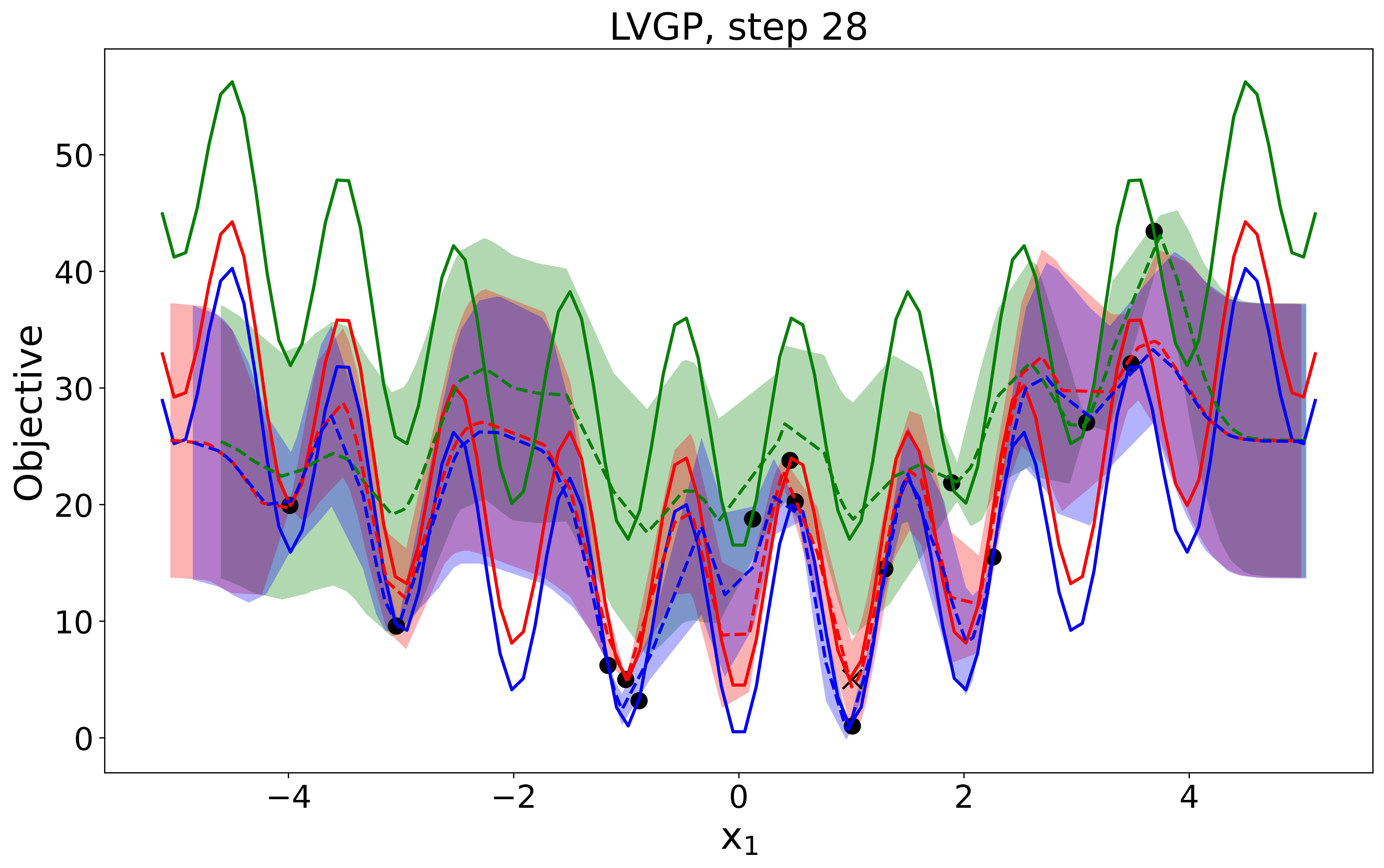

Supplement: Supplementary file 1 — Supplementary Information 1. [file 41598_2022_23431_MOESM1_ESM.zip › Sampling_Sequence_Figures/Rastrigin_Function/rastrigin2_LVGP_28.jpg]

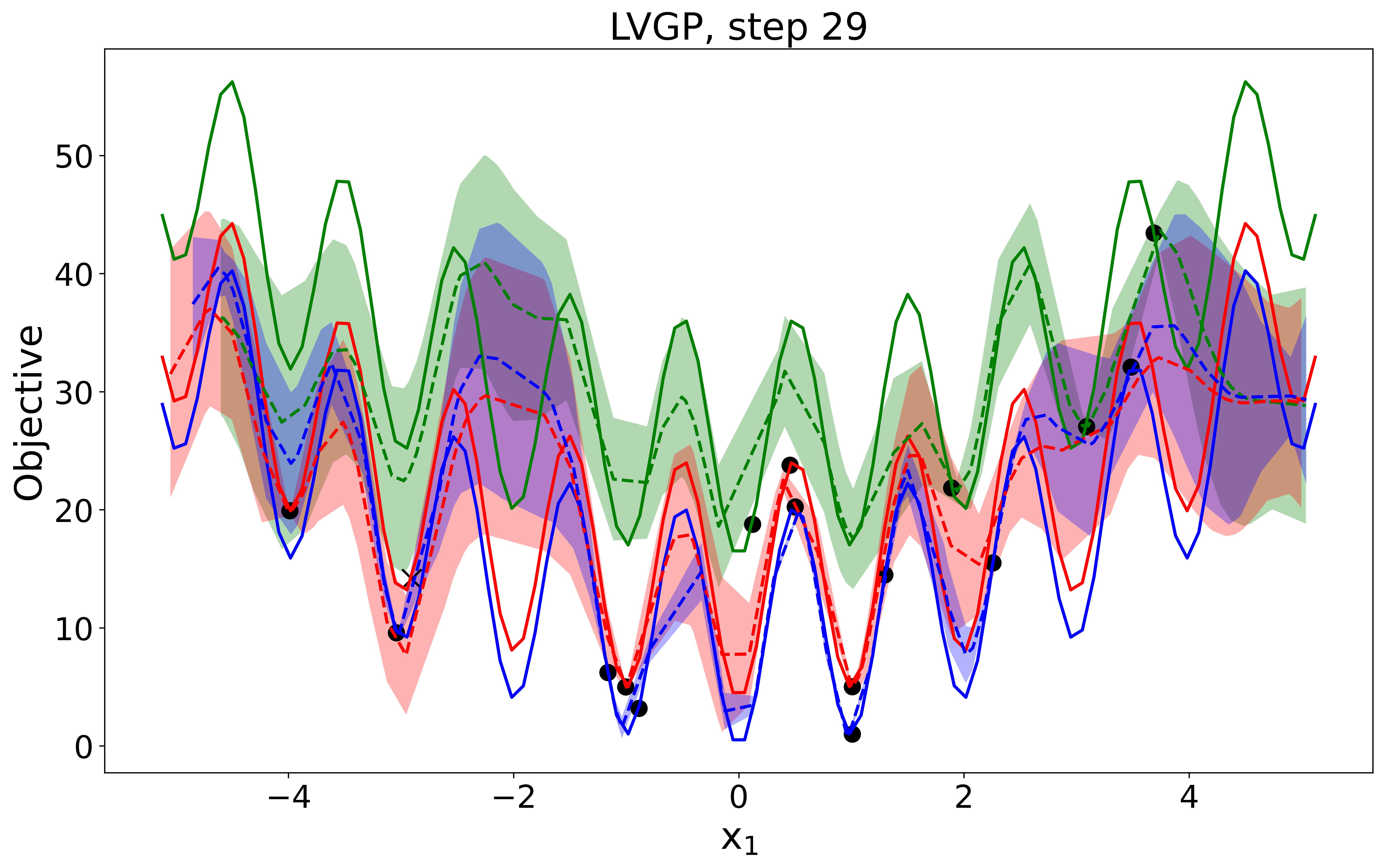

Supplement: Supplementary file 1 — Supplementary Information 1. [file 41598_2022_23431_MOESM1_ESM.zip › Sampling_Sequence_Figures/Rastrigin_Function/rastrigin2_LVGP_29.jpg]

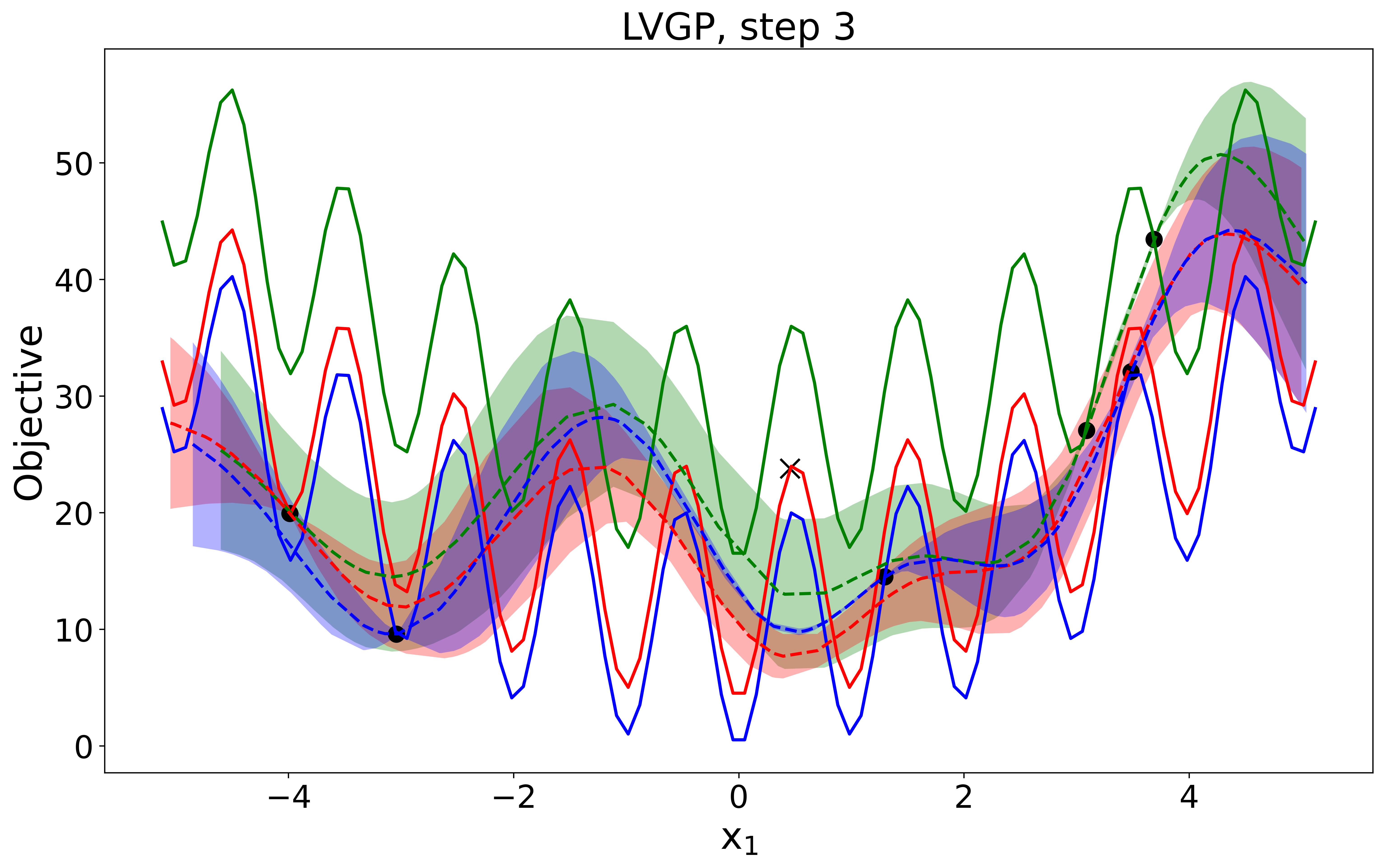

Supplement: Supplementary file 1 — Supplementary Information 1. [file 41598_2022_23431_MOESM1_ESM.zip › Sampling_Sequence_Figures/Rastrigin_Function/rastrigin2_LVGP_3.jpg]
