# Supplementary material for: Uncertainty-aware mixed-variable machine learning for materials design
Source: Sci Rep. 2022 Nov 17;12:19760. doi: 10.1038/s41598-022-23431-2 (PMC9672324; doi:10.1038/s41598-022-23431-2)
Supplement: Supplementary file 2 — Supplementary Information 2. [file 41598_2022_23431_MOESM2_ESM.pdf]

Uncertainty-Aware Mixed-Variable Machine Learning for Materials Design: Supplementary Information

Hengrui Zhang, Wei (Wayne) Chen, Akshay Iyer, Daniel W. Apley, and Wei Chen

October 4, 2022

1 Uncertainty Quantification in Mixed-Variable ML

Two uncertainty-aware mixed-variable ML models, Lolo and LVGP, are illustrated in Figures S1 and S2, respectively.

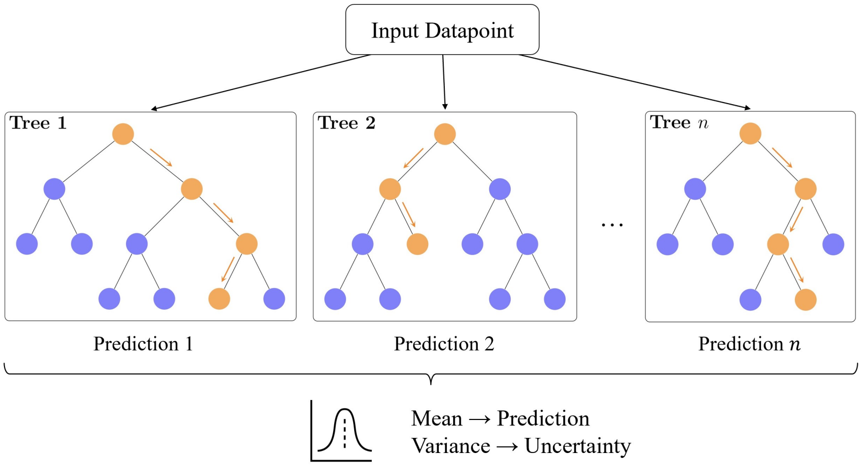

FIG. S1: Illustration of the Lolo model. In the training stage, n decision trees are constructed. In the prediction stage, for an input datapoint, each tree gives a prediction. Predicted mean and uncertainty are given by the mean value and variance estimation among n trees' predictions, respectively.

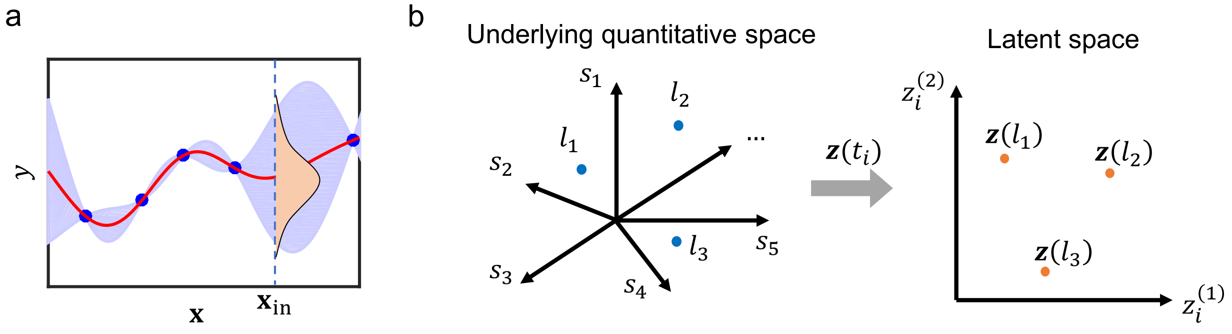

FIG. S2: Illustrations of Gaussian Process and LVGP models. (a) GP models assign prior distributions for y at various $\boldsymbol{x}$ locations. Given training data, the posterior distribution of y provides uncertainty quantification. (b) LVGP model assumes that levels of a categorical variable are characterized by underlying quantitative factors (represented as s). Represented as points in a low-dimensional latent space, the distances between levels reflect their similarities in terms of effect on the response.

2 Mathematical Test Cases

Bayesian Optimization (BO) results of mathematical test functions included in the main text, with different initial sample sizes: Branin function in Figure S3, and Camel function in Figure S4.

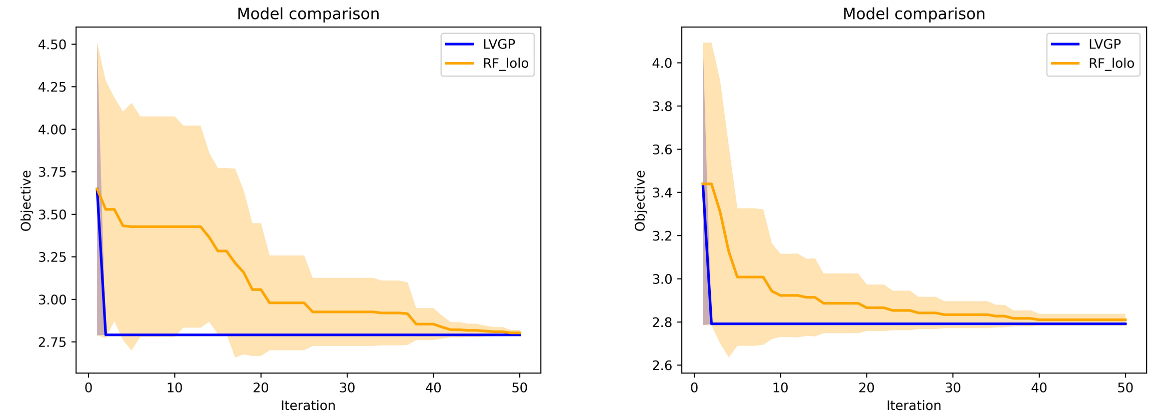

FIG. S3: Optimization history plots of the Branin function with initial sample sizes 50 (left) and 100 (right).

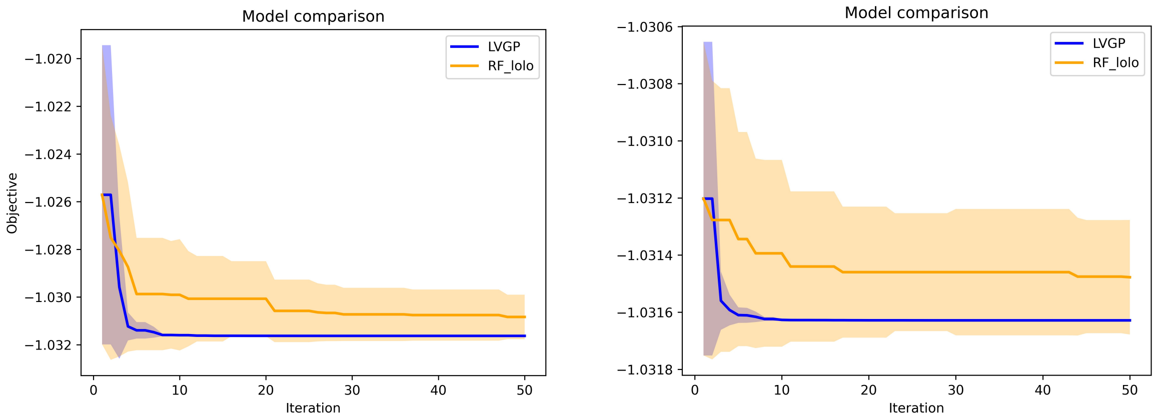

FIG. S4: Optimization history plots of the Camel function with initial sample sizes 50 (left) and 100 (right).

The Rastrigin function in 3-dimensional (1 categorical) and 4-dimensional (2 categorical) forms, with 30 initial samples. Plots (Figure S5) are created from results of 10 runs with different random initial samples.

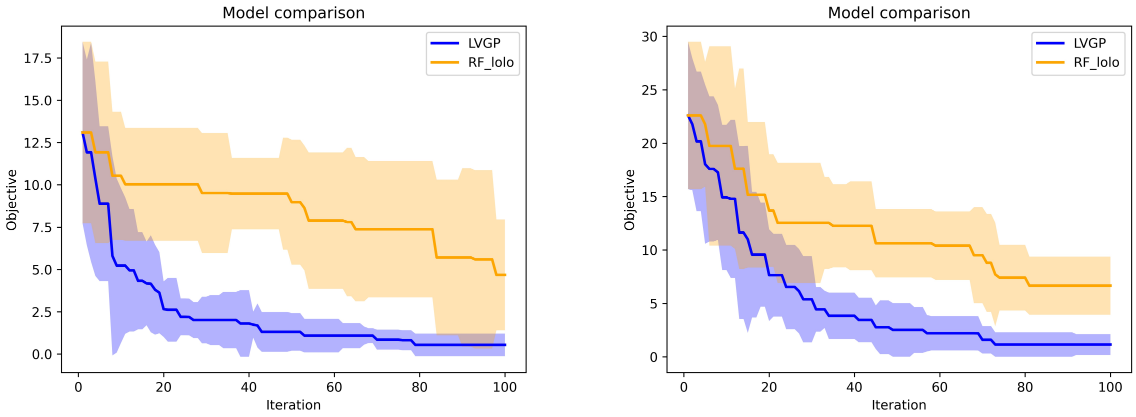

FIG. S5: Optimization history plots of the 3D (left) and 4D (right) Rastrigin functions.

The Perm function in 6-dimensional form (1 categorical variable with 3 levels), starting from 80 initial samples. Figure S6 is created from 10 runs.

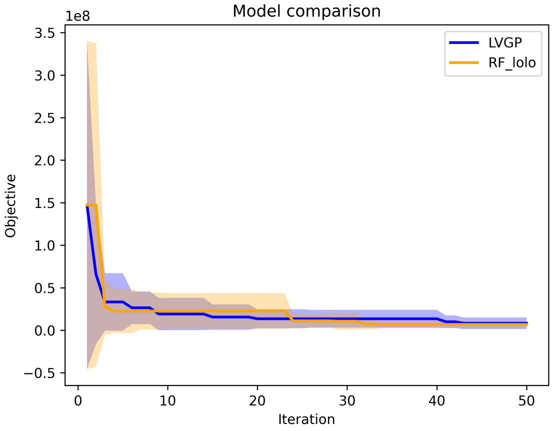

FIG. S6: Optimization history plot of the Perm function.

The Holder Table function

$$f(x,t) = -\left|\sin(x)\cos(t)\exp\left(\left|1 - \frac{\sqrt{x^2+t^2}}{\pi}\right|\right)\right|, \tag{1}$$

where $x \in [-10, 10]$, and $t \in \{\pm 10, \pm 9, \ldots, 0\}$. Plots (Figure S7) are created from 10 runs.

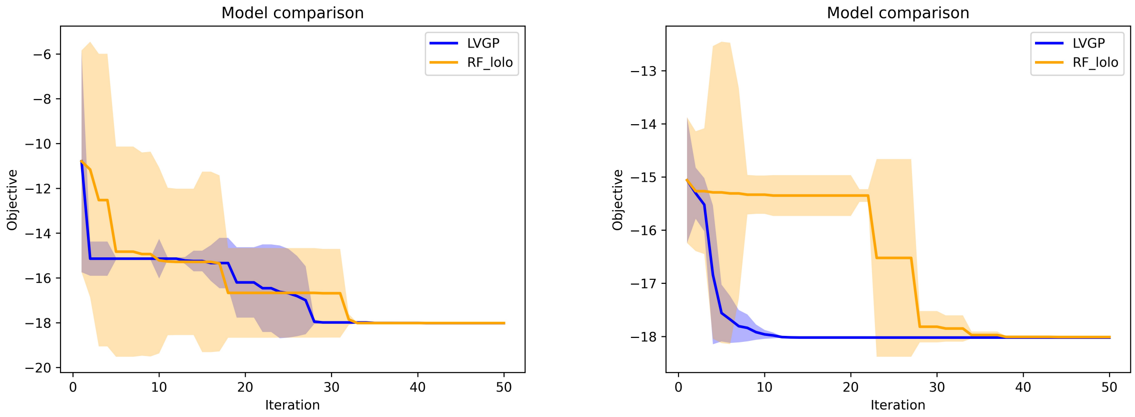

FIG. S7: Optimization history plots of the Holder Table function with initial sample sizes 21 (left) and 50 (right).

The Ackley function

$$f(\mathbf{v}) = -20 \exp\left(-b\sqrt{\frac{1}{d}\sum_{i=1}^{d} v_i^2}\right) - \exp\left(\frac{1}{d}\sum_{i=1}^{d}\cos\left(cv_i\right)\right) + 20 + e, \tag{2}$$

where $e = 2.71828...$; we set the dimensionality $d = 3$, with $v_{1,2} = x_{1,2} \in [-32.768, 32.768]$, and $v_3 = t \in \{\pm 32, \pm 31, ..., 0\}$. Figure S8 is created from 5 runs.

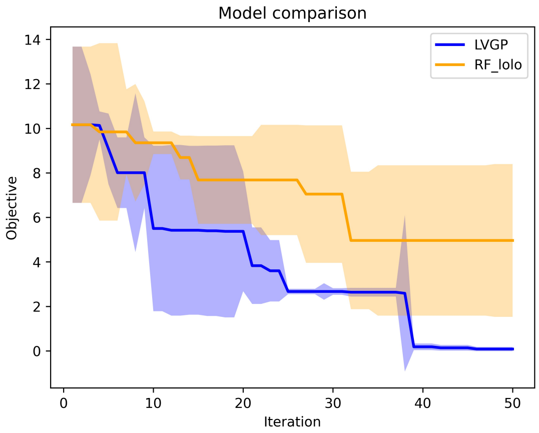

FIG. S8: Optimization history plot of the Ackley function with initial sample size 65.

The Cross-in-Tray function

$$f(x,t) = -0.0001\left(\left|\sin\left(x\right)\sin\left(t\right)\exp\left(\left|100 - \frac{\sqrt{x^2+t^2}}{\pi}\right|\right)\right| + 1\right)^{0.1}, \tag{3}$$

where $x \in [-10, 10]$, and $t \in \{\pm 10, \pm 9, ..., 0\}$. Figure S9 is created from 10 runs.

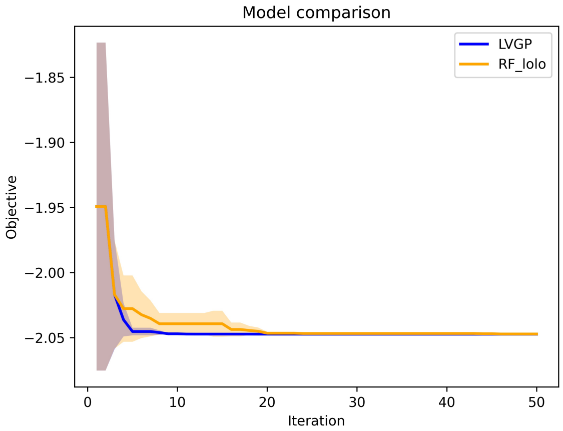

FIG. S9: Optimization history plot of the Cross-in-Tray function with initial sample size 21.

The Shubert function

$$f(x,t) = \left(\sum_{i=1}^{5} i\cos\left((i+1)x+i\right)\right)\left(\sum_{i=1}^{5} i\cos\left((i+1)t+i\right)\right), \tag{4}$$

where $x \in [-10, 10]$, and $t \in \{\pm 10, \pm 9, ..., 0\}$. Figure S10 is created from 10 runs.

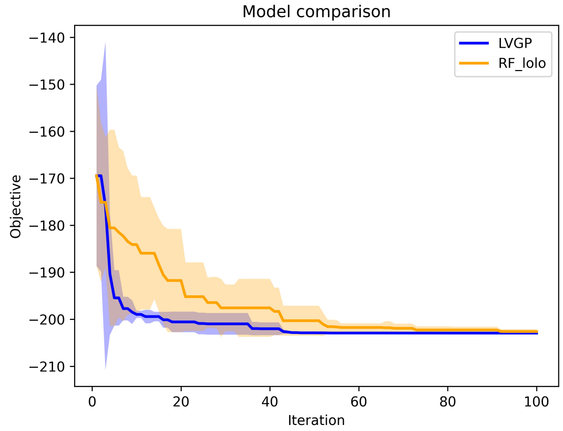

FIG. S10: Optimization history plot of the Shubert function with initial sample size 21.

Sampling path Figures that show the sampling sequences by LVGP-BO and Lolo-BO in optimizing the Branin function are provided in a separate compressed file.

3 Materials Property Test Cases

Figure S11 showing the relations between Young's, shear, and bulk modulus of M_2AX compounds, indicate that Young's and shear moduli are highly correlated, while bulk modulus is not with the others.

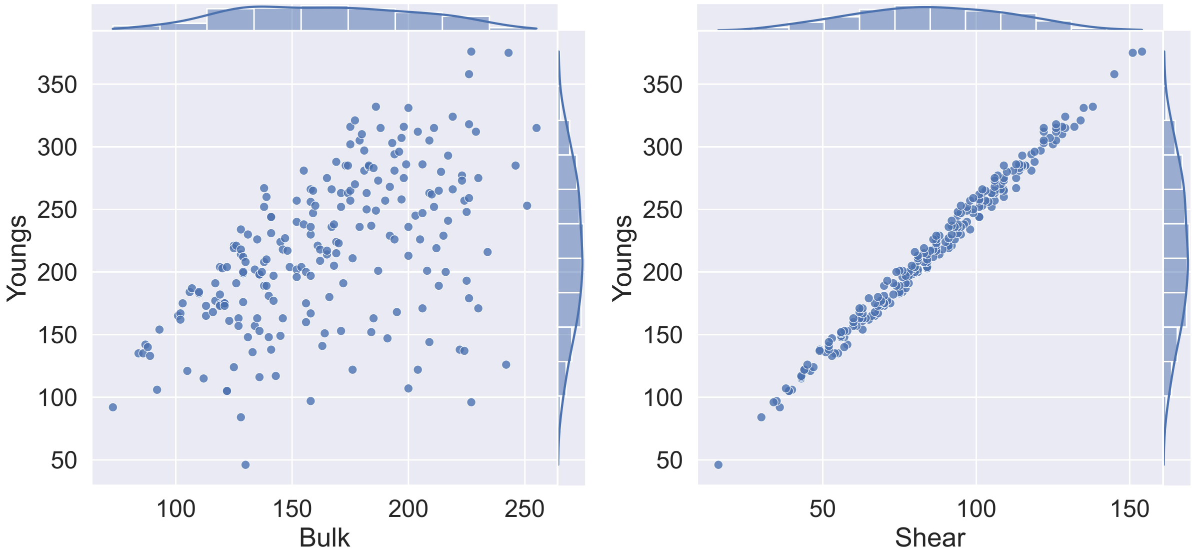

FIG. S11: Scatter plots of Young's–bulk modulus (E–B) and Young's–shear modulus(E–G).

Figure S12 shows the BO and ML fitting results for the shear modulus of $\mathrm{M_2AX}$ compounds.

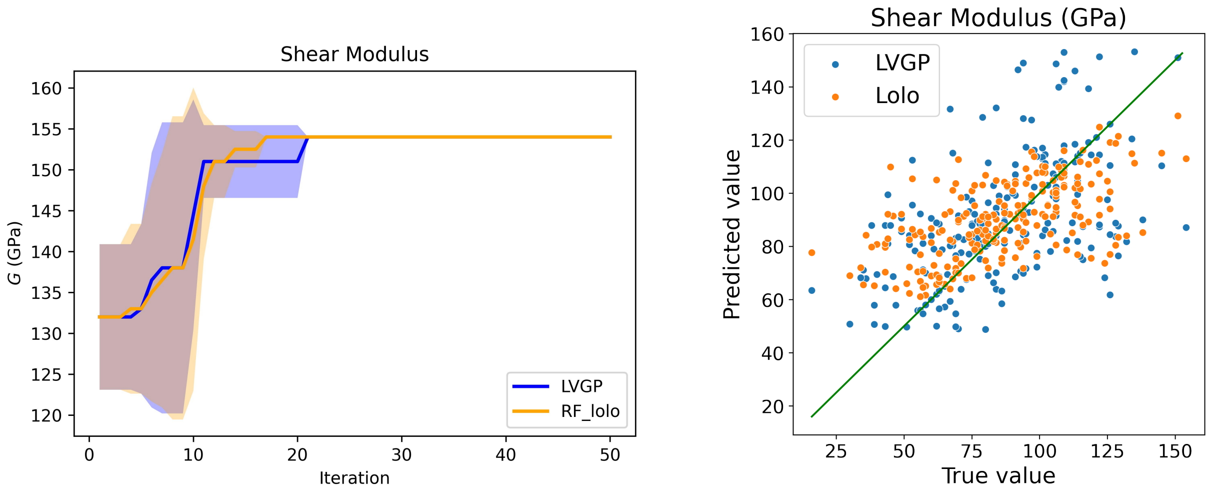

FIG. S12: Optimization history plot (left) and regression plot (right) of $\mathrm{M_2AX}$ shear modulus, with 30 initial samples.
